# Studies on the syntheses of β-carboline alkaloids brevicarine and brevicolline

**DOI:** 10.3762/bjoc.21.79

**Published:** 2025-05-20

**Authors:** Benedek Batizi, Patrik Pollák, András Dancsó, Péter Keglevich, Gyula Simig, Balázs Volk, Mátyás Milen

**Affiliations:** 1 Department of Organic Chemistry and Technology, Faculty of Chemical Technology and Biotechnology, Budapest University of Technology and Economics, Műegyetem rkp. 3, H-1111 Budapest, Hungary; 2 Egis Pharmaceuticals Plc., Directorate of Drug Substance Development, P. O. Box 100, Keresztúri út 30-38, H-1475 Budapest, Hungary; 3 Department of Organic Chemistry, Faculty of Pharmacy, Semmelweis University, Hőgyes Endre utca 7, H-1092 Budapest, Hungary; 4 Center for Pharmacology and Drug Research & Development, Semmelweis University, Üllői út 26, H-1085 Budapest, Hungary

**Keywords:** alkaloid, β-carboline, *Carex brevicollis DC*, cross-coupling reaction, trifluoroethylation

## Abstract

A new total synthesis of the β-carboline alkaloid brevicarine is disclosed. The synthesis was carried out starting from an aromatic triflate key intermediate, allowing the introduction of various substituents into position 4 of β-carboline by cross-coupling reactions. Thanks to its scalability, this novel approach ensures a broad accessibility to the target compound for potential pharmacological measurements. Using detailed NMR studies, the NMR signals have been assigned for both the base and its dihydrochloride salt for further confirming their structures. A new synthesis of the related alkaloid brevicolline was also attempted from the same intermediate. However, after successful coupling of β-carboline with *N*-methylpyrrole, the trials to saturate the pyrrole ring under various conditions led to unexpected reactions: reduction of ring A of the β-carboline skeleton or trifluoroethylation of the pyrrole moiety occurred, leading to interesting and potentially useful derivatives.

## Introduction

**Figure 1:**
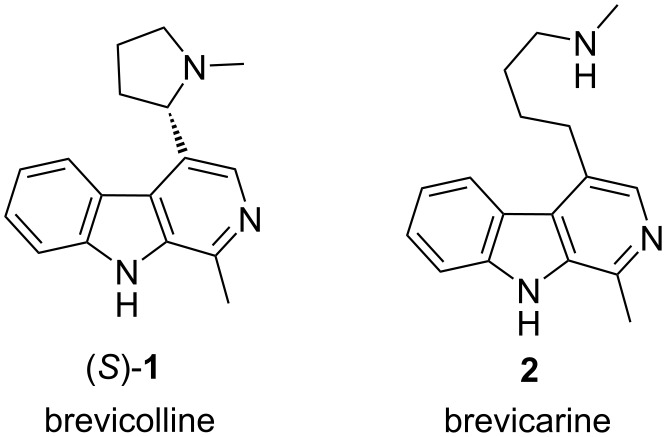
The structure of brevicolline ((*S*)-**1**) and brevicarine (**2**).

*Carex brevicollis DC* is a widely distributed sedge which can be mainly found in the Central and South-Eastern European region. It contains several alkaloids, including β-carboline alkaloids (*S*)-brevicolline (*S*-(**1**)) and brevicarine (**2**, Figure 1) as the two main components [1–3]. Our long-standing interest in the chemistry of β-carbolines [4–18] has now focused our attention on these two alkaloids.

**Scheme 1:**
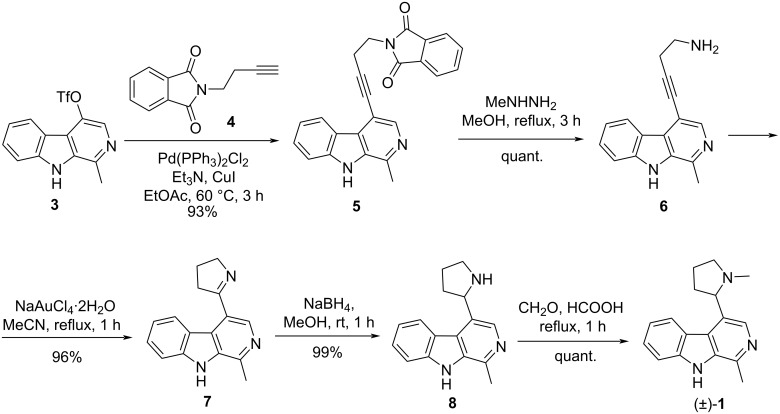
Synthesis of racemic brevicolline ((±)-**1**) starting from 1-methyl-9*H*-β-carbolin-4-yl trifluoromethanesulfonate (**3**).

In a recent publication we disclosed a new total synthesis of racemic brevicolline ((±)-**1**) (Scheme 1) [9]. A prerequisite for the synthesis was the development of a new, versatile key triflate intermediate **3**, which allowed the introduction of substituents attached by a C–C bond to position 4 of the β-carboline scaffold by cross-coupling reactions. Sonogashira reaction of compound **3** with *N*-(3-butynyl)phthalimide (**4**) led to coupled compound **5**. Cleavage of the phthalimide group with methylhydrazine afforded butynylamine derivative **6**. Cyclization of the latter to dihydropyrrole **7** and subsequent reduction resulted in compound **8**, which was *N*-methylated to give racemic brevicolline ((±)-**1**). The natural product (*S*)-brevicolline ((*S*)-**1**) was finally obtained by chiral chromatography of the corresponding racemate.

**Scheme 2:**
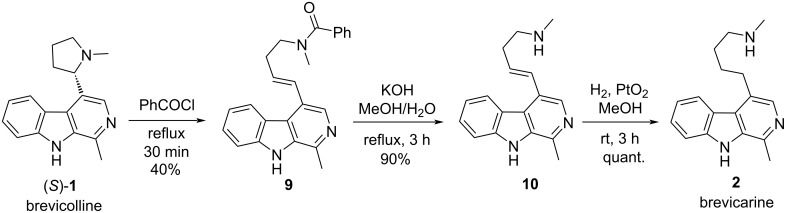
Synthesis of brevicarine (**2**) from brevicolline ((*S*)-**1**).

To continue this work, we decided to attempt the synthesis of brevicarine (**2**), a structurally related alkaloid, as well. There are some published examples for the synthesis of brevicarine in the literature. The first semi-synthetic access to brevicarine (**2**) was achieved by a few-step transformation starting from brevicolline ((*S*)-**1**) isolated from natural sources (Scheme 2) [19]. When heating (*S*)-**1** in benzoyl chloride, opening of the pyrrolidine ring and *N*-benzoylation occurred, resulting in compound **9**. Debenzoylation of the latter to **10**, followed by the catalytic hydrogenation of the C=C double bond in the side chain gave brevicarine (**2**).

**Scheme 3:**
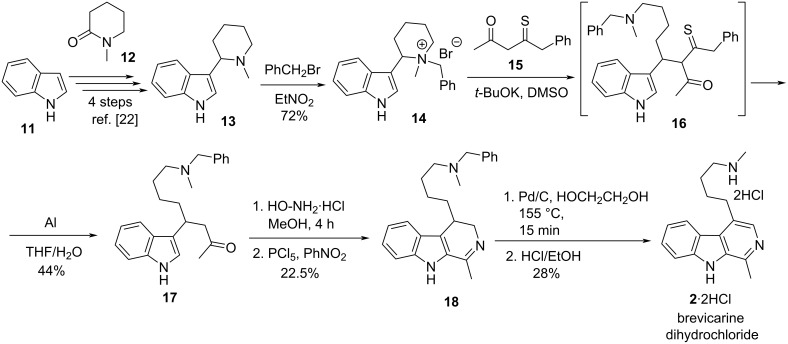
First total synthesis of brevicarine (**2**).

The first total synthesis of brevicarine is shown in Scheme 3 [2,20–21]. Condensation of indole (**11**) with 1-methylpiperidone (**12**) gave compound **13** [22]. *N*-Alkylation of **13** with benzyl bromide, followed by treatment of the quaternary ammonium derivative **14** with the potassium salt of compound **15** resulted in the ring-opened derivative **16**. Removal of the benzylthiocarbonyl moiety, then Beckmann rearrangement of the oxime obtained from ketone **17** and subsequent cyclization gave β-carboline derivative **18**, which was dehydrogenated and debenzylated to brevicarine (**2**), isolated as a dihydrochloride salt.

**Scheme 4:**
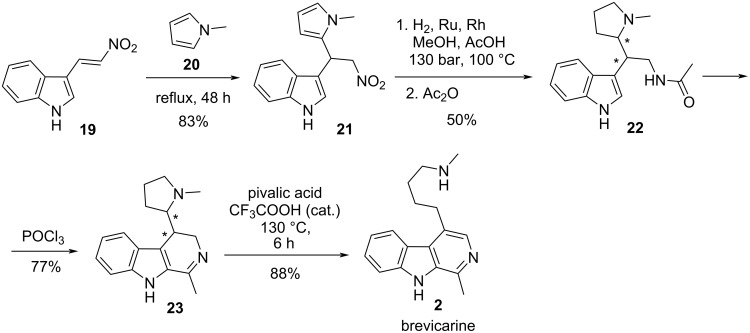
Multistep synthesis of brevicarine (**2**) starting from nitrovinylindole **19**.

Müller et al. accomplished an alternative synthesis of brevicarine (**2**, Scheme 4) [23]. Compound **21** was obtained by treatment of nitrovinylindole **19** with *N*-methylpyrrole (**20**). Catalytic hydrogenation of the pyrrole ring and the nitro group of **21** under extremely harsh conditions (100 °C, 130 bar), followed by *N*-acetylation gave a mixture of diastereomeric racemates **22**, which was cyclized to a diastereomeric mixture of β-carboline derivatives **23**. Heating of **23** in pivalic acid in the presence of a catalytic amount of trifluoroacetic acid gave brevicarine (**2**).

The sedge *Carex brevicollis DC* has long been recognized for its ability to stimulate the contraction of smooth muscles [2]. This effect may be linked to the oxytocic activity of (*S*)-brevicolline ((*S*)-**1**), which has been studied on pregnant mammals [24–25]. (*S*)-Brevicolline, first isolated in 1960 [3], was tested in vitro and demonstrated antibacterial and antifungal properties due to its photosensitizing ability [26]. It was used in medical practice as an obstetrical drug and also in veterinary practice for treating infertility [2]. Experiments investigating the synthesis of brevicarine (**2**) from (*S*)-brevicolline ((*S*)-**1**) (see Scheme 2) and their biogenetic relationship suggest that (*S*)-brevicolline could serve as a biosynthetic intermediate of brevicarine (**2**) in plants [19].

Brevicarine (**2**), isolated in 1967 from *Carex brevicollis DC* [27], has also been identified in various other natural sources, including *Tambourissa ficus* (mauritian endemic fruit) [28], *Asparagus racemosus* (linn seed) [29] and a mixture extract of *Phellinus linteus smilax corbularia* and *Phellinus linteus smilax glabra* [30]. Literature reports indicate that brevicarine exhibits several pharmacological activities: it acts as an antioxidant [28], shows antibacterial activity against *Mycobacterium tuberculosis* [31], has antiproliferative effects against triple-negative breast cancer [32], serves as an agent against Parkinson's disease [29], and possesses skin anti-inflammatory properties [33]. Notably, the dihydrochloride salt of the alkaloid has been tested in vivo in rats, cats, and rabbits as an antiarrhythmic agent, demonstrating superior efficacy compared to the commercially available drugs quinidine and novocainamide [34]. *N*-Methylbrevicarine, a semi-synthetic derivative of the alkaloid, has been screened in silico for non-peptide malignant brain tumor (MBT) antagonist activity, showing hits on three MBT-containing proteins [35]. Although *Carex brevicollis DC* has been observed to have a teratogenic effect on animals [1,36], which could be linked with the presence of the two mentioned β-carboline alkaloids, further investigation is needed to prove this observation. However, the mentioned alkaloids have high potential as medications. Based on the above, research on the total synthesis of these alkaloids and their closely related derivatives is crucial for further confirming their structure and for ensuring their accessibility for pharmacological measurements.

## Results and Discussion

**Scheme 5:**
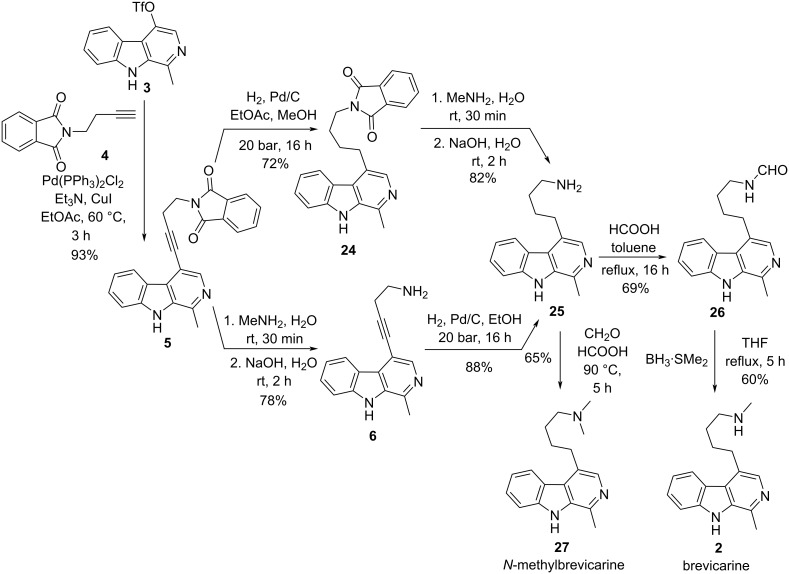
New synthesis variants for the preparation of brevicarine alkaloid (**2**) and its synthetic derivative *N*-methylbrevicarine (**27**).

In the present study, we aimed to develop a novel, scalable synthesis of brevicarine (**2**) and an alternative synthetic approach for the preparation of brevicolline (**1**), both based on the common key intermediate **3** [9]. The synthesis of brevicarine (**2**) (Scheme 5) started with the known synthesis of **5** from **3**, followed by catalytic reduction of the triple bond of phthaloyl intermediate **5** to give compound **24**. Removal of the phthalimide group with methylamine resulted in amine **25**. Alternatively, the removal of the phthaloyl moiety from compound **5** to amine **6** using methylamine instead of the highly toxic and environmentally harmful methylhydrazine, which was used earlier [9], has been developed as a greener approach, followed by catalytic reduction of the triple bond also leading to compound **25**.

Our experiments for the *N*-monomethylation of the primary amino group of compound **25** by alkylation with methyl iodide or by Eschweiler–Clarke reductive amination with formaldehyde and formic acid were unsuccessful, because the dimethylated byproduct was also formed, even when one equivalent alkylating agent was used. Finally, our efforts were crowned by success. In order to completely avoid the possibility of overmethylation [37], the methyl group was introduced by *N*-formylation of the primary amine group of **25** to give congener **26**, followed by reduction of the formyl group with borane–dimethyl sulfide complex [38] to result in brevicarine (**2**, isolated as its dihydrochloride salt). Based on the above results, Eschweiler–Clarke methylation of primary amine **25** was applied for the synthesis of *N*-methylbrevicarine (**27**) [39], a close structural analogue of alkaloid **2**.

The NMR data of our synthesized brevicarine (**2**) base and dihydrochloride salt are summarized in Table 1. Although a rudimentary ^1^H NMR spectrum of isolated brevicarine (**2**) base and some of the NMR signals were reported in 1969 [39], the signals were not fully assigned. However, the mentioned signals are identical to those of our synthetic product. All other publications [19,23–24] confirmed the structure with IR and MS data, or by reactivity. Herein, we report the full set of assigned ^1^H and ^13^C NMR data for both the base and the dihydrochloride salt.

**Table 1:** Assigned ^1^H and ^13^C NMR data of brevicarine (**2**) base and its dihydrochloride salt.

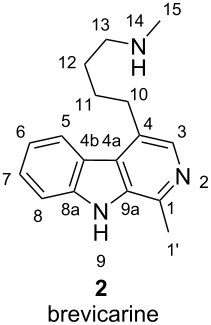				

Atom no.	Synthetic brevicarine		Synthetic brevicarine dihydrochloride salt	
	^1^H NMR (DMSO-*d*_6_, 600 MHz), δ_H_	^13^C NMR (DMSO-*d*_6_, 150 MHz), δ_C_	^1^H NMR (DMSO-*d*_6_, 600 MHz), δ_H_	^13^C NMR (DMSO-*d*_6_, 150 MHz), δ_C_

1	–	140.1	–	140.3
1’	2.72 (s, 3H)	20.4	2.74 (s, 3H)	20.2
2	–	–	–	–
3	7.99 (s, 1H)	137.7	8.03 (s, 1H)	137.4
4	–	129.0	–	128.4
4a	–	125.1	–	125.3
4b	–	121.1	–	121.0
5	8.13 (m, 1H)	123.3	8.13 (m, 1H)	123.4
6	7.25 (m, 1H)	119.6	7.27 (m, 1H)	119.7
7	7.53 (m, 1H)	127.3	7.55 (m, 1H)	127.6
8	7.61 (m, 1H)	112.1	7.64 (m, 1H)	112.2
8a	–	140.5	–	140.6
9	11.57 (br s, 1H)	-	11.70 (br s, 1H)	–
9a	–	134.5	–	134.5
10	3.11 (t, *J* = 7.6 Hz, 2H)	30.8	3.17 (t, *J* = 7.1 Hz, 2H)	30.2
11	1.75 (m, 2H)	27.5	1.79 (m, 2H)	26.3
12	1.55 (m, 2H)	29.3	1.72 (m, 2H)	25.4
13	2.50 (m, 2H)	51.5	2.91 (t, *J* = 7.4 Hz, 2H)	48.3
14	–	–	8.56 (br s, 2H)	–
15	2.25 (s, 3H)	36.4	2.50 (s, 3H)	32.6

**Scheme 6:**
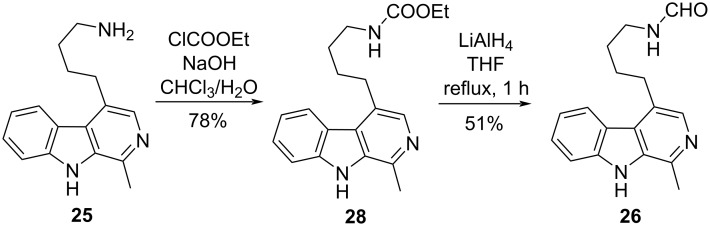
Preparation of carbamate **28** and subsequent reduction with LiAlH_4_.

In the course of our efforts devoted to elaborating a new and efficient synthesis of brevicarine (**2**), we encountered a surprising reaction (Scheme 6). Based on literature data, we expected to transform carbamate **28**, obtained from amine **25** by ethoxycarbonylation, into brevicarine (**2**) by reduction with LiAlH_4_ [40–41]. However, to our surprise, the reduction stopped at the *N*-formyl (**26**) stage, the formation of brevicarine (**2**) could not be detected by LC–MS.

**Scheme 7:**
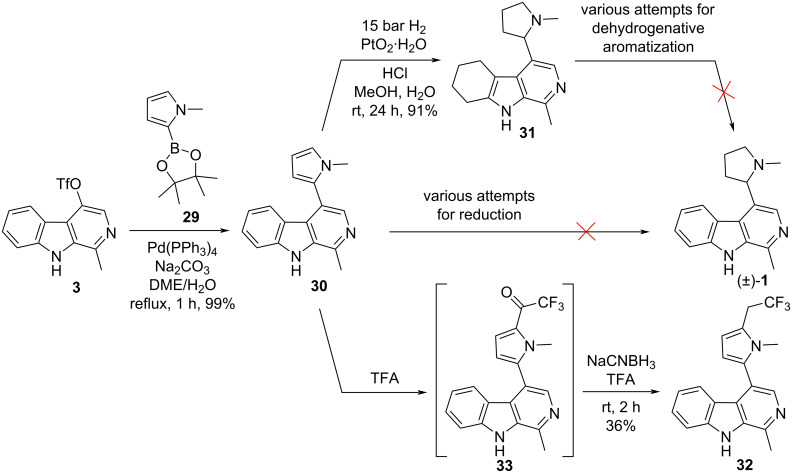
Experiments for the synthesis of racemic brevicolline ((±)-**1**), and formation of unexpected products.

As regards our plans for an alternative synthesis of racemic brevicolline ((±)-**1**), our primary goal was the direct coupling of the pyrrole ring to compound **3**, instead of its ring-closing construction shown in Scheme 1. Suzuki reaction of **3** with pyrrole boronic ester **29** gave pyrrolo-β-carboline **30** in excellent yield (Scheme 7). Our attempts for the selective saturation of the pyrrole ring of **30** by catalytic reduction were unsuccessful. When the hydrogenation was carried out under mild conditions (ambient temperature, 15 bar H_2_) in the presence of PtO_2_**^.^**H_2_O catalyst, overreduced product **31**, i.e., the tetrahydro derivative of racemic brevicolline ((±)-**1**) was obtained in 91% yield.

**Figure 2:**
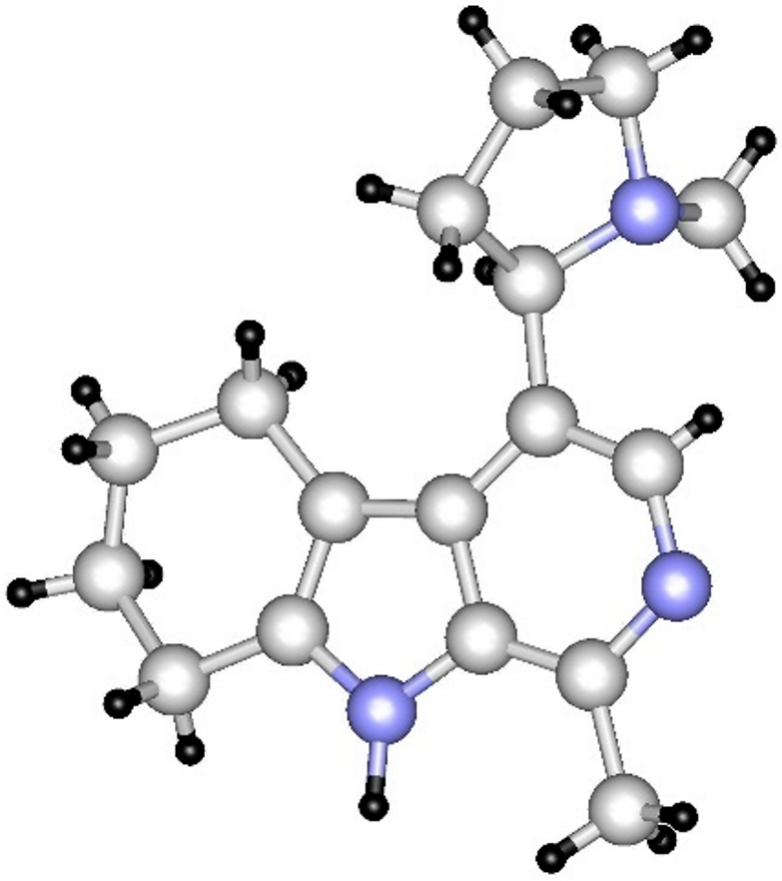
X-ray structure of compound **31**.

Structure determination of **31** was supported by single-crystal X-ray diffraction, as well (Figure 2). Changing the catalyst [Pd(OH)_2_, Ru, Rh], did not alter the course of the reaction: the formation of compound **31** was always observed, and brevicolline ((±)-**1**) was not formed. Interestingly, our attempts made for the transformation of compound **31** by dehydrogenative aromatization to brevicolline ((±)-**1**) by using several reagents (DDQ, Pd/C, MnO_2_, CuCl_2_, I_2_, elemental sulfur, KMnO_4_) were also ineffective. Based on literature data [42–43], we attempted the selective reduction of the pyrrole ring of compound **30** with NaCNBH_3_ in TFA as well. Surprisingly, trifluoroethylated product **32** was isolated. The formation of this compound can also be explained on the basis of analogies described in the literature for trifluoroacetylation of aromatic ring systems with TFA [44–45]. Nevertheless, in our case, trifluoroacetylation of the pyrrole moiety of **30** by TFA and reduction of the carbonyl group of **33** with NaCNBH_3_ took place in one pot, which is unprecedented in the literature. It is worth mentioning that in a similar reaction of **30** with NaCNBH_3_ in acetic acid (instead of TFA) we did not observe any reaction, however, with NaBH_4_ (instead of NaCNBH_3_) in TFA, the formation of **32** was detected.

## Conclusion

In conclusion, a new method for the synthesis of β-carboline alkaloid brevicarine has been elaborated rendering the preparation of larger amounts of the target compound possible. NMR data of brevicarine base and dihydrochloride salt were fully assigned for a further confirmation of their structure. In the course of the unsuccessful attempts for a new synthesis of brevicolline, we synthesized several new, potentially pharmacologically active β-carboline derivatives structurally close to the alkaloids brevicarine and brevicolline. These derivatives (**25**–**28**, **30**–**32**) can also serve as versatile starting materials for the synthesis of new alkaloid analogues and other C(4)-substituted β-carbolines. Some surprising reactions were also observed, such as the unexpected formation of racemic tetrahydrobrevicolline and the trifluoroethylation of the pyrrole moiety, which can also serve as favorable starting points for further research.

## Supporting Information

CCDC 2410549 (**31**) contain supplementary crystallographic data for this paper. These data are provided free of charge by The Cambridge Crystallographic Data Centre via http://www.ccdc.ac.uk/data request/cif.

File 1 ORTEP diagram of compound **31**, synthetic procedures, IR, ^1^H, ^13^C, ^19^F and 2D NMR spectra of compounds **2**, **5**, **6**, **24**–**28** and **30**–**32**.

File 2 Crystallographic information file of compound **31**.

File 3 Checkcif file for compound **31**.

## Data Availability

Additional research data generated and analyzed during this study is not shared.
