# Supplementary material for: Studies on the syntheses of β-carboline alkaloids brevicarine and brevicolline
Source: Beilstein J Org Chem. 2025 May 20;21:955–63. doi: 10.3762/bjoc.21.79 (PMC12117210; doi:10.3762/bjoc.21.79)
Supplement: File 1 — ORTEP diagram of compound 31, synthetic procedures, IR, 1H, 13C, 19F and 2D NMR spectra of compounds 2, 5, 6, 24–28 and 30–32. [file Beilstein_J_Org_Chem-21-955-s001.pdf]

BEILSTEIN JOURNAL OF ORGANIC CHEMISTRY

Supporting Information

for

Studies on the syntheses of β-carboline alkaloids brevicarine and brevicolline

Benedek Batizi, Patrik Pollák, András Dancsó, Péter Keglevich, Gyula Simig, Balázs Volk and Mátyás Milen

Beilstein J. Org. Chem. **2025,** *21,* 955–963. doi:10.3762/bjoc.21.79

ORTEP diagram of compound 31, synthetic procedures, IR, ^{1}H, ^{13}C, ^{19}F and 2D NMR spectra of compounds 2, 5, 6, 24–28 and 30–32

License and Terms: This is a supporting information file under the terms of the Creative Commons Attribution License (https://creativecommons.org/licenses/by/4.0). Please note that the reuse, redistribution and reproduction in particular requires that the author(s) and source are credited and that individual graphics may be subject to special legal provisions.
The license is subject to the *Beilstein Journal of Organic Chemistry* terms and conditions: (https://www.beilstein-journals.org/bjoc/terms)

Content:

Figure S1. X-ray structure (ORTEP diagram) of compound **31**.……………………….………S2

Experimental section of compounds **2**, **5**, **6**, **24**–**28** and **30**–**32**………………………………S3

References…………………………………………………………………………...S12

IR, ^{1}H, ^{13}C, ^{19}F and 2D NMR spectra of compounds **2**, **5**, **6**, **24**–**28** and **30**–**32**……………S13

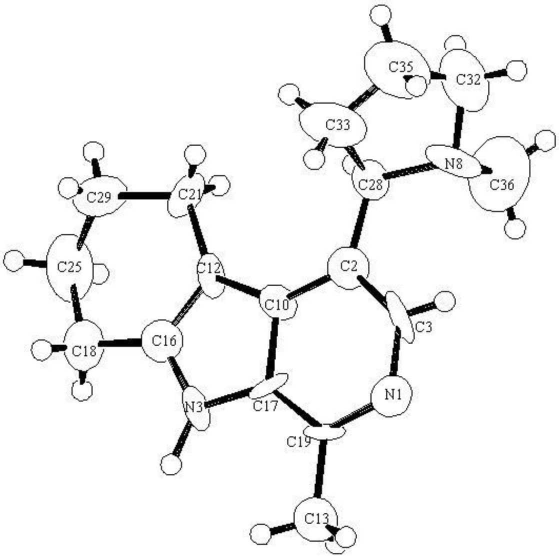

Figure S1. X-ray structure (ORTEP diagram) of compound **31**

Experimental

General

All melting points were determined on a Büchi B-540 capillary melting point apparatus and are uncorrected. IR spectra were obtained on a Bruker Alpha FT-IR spectrometer in transmission mode in KBr pellets, film or attenuated total reflection (ATR). ^{1}H NMR and ^{13}C NMR spectra were recorded in DMSO-d_6 solution in 5 mm tubes at room temperature, on a Bruker Avance III HD (600, 150 and 564 MHz for ^{1}H, ^{13}C and ^{19}F NMR spectra, respectively) spectrometer with the deuterium signal of the solvent as the lock and TMS as the internal standard. Chemical shifts (δ) and coupling constants (J) are given in ppm and in Hz, respectively. The following abbreviations are used to designate multiplicities: s = singlet, d = doublet, t = triplet, q = quartet, m = multiplet, br = broad. Mass spectra were recorded on a Bruker O-TOF MAXIS Impact mass spectrometer coupled to a Dionex Ultimate 3000 RS HPLC system with a diode array detector. High-resolution mass spectra (HRMS) were recorded on a Bruker Q-TOF MAXIS Impact mass spectrometer coupled with a Waters I-Class UPLC system with a diode array detector (for $[M+H]^+$) or on an Agilent 7250 Q-TOF mass spectrometer coupled to an Agilent 8890 gas chromatographic system (for $[M]^+$). The single crystal X-ray diffraction measurements were carried out on a Rigaku R-Axis Spider instrument with image plate detector. Single-crystal X-ray structure of compound **31** has been uploaded to the CCDC database (CCDC number: 2410549). Reactions were monitored by thin-layer chromatography (TLC) carried out on silica gel plates (60 F_{254}) using UV light as visualizing agent. Purifications by flash column chromatography were carried out using Merck 107736 silica gel 60 H using a hexane–EtOAc or DCM–MeOH solvent system. All reagents were purchased from commercial sources and used without further purification. Analytical samples of synthesized compounds were obtained by recrystallization from the solvents or solvent mixtures given below in

parentheses. Compounds **2**, **5**, **6** are described in the literature, while compounds **24**–**28**, **30**–**32** are new and characterized below.

Procedures

***N*-Methyl-4-(1-methyl-9*H*-β-carbolin-4-yl)butan-1-amine (2)**

Compound **26** (61 mg, 0.22 mmol) was dissolved in dry THF (5.0 mL) and $BH_3 \cdot SMe_2$ (66 µL, 0.87 mmol) reagent was added. The mixture was heated to reflux and reacted for 5 hours. After that, the mixture was cooled to room temperature and 37 wt % aqueous HCl solution (3.0 mL) was added. The mixture was heated to reflux and reacted for 1 hour. The mixture was poured onto ice and treated with 10 wt % aqueous NaOH until pH 7. It was extracted with DCM (3 × 15 mL). The combined organic layer was dried over Na_2SO_4 and concentrated in vacuo to give a colorless oil (44 mg, 60 %). IR (ATR): 3142, 3059, 1620, 1455, 1323, 1293, 735 cm^{-1}. ^1H NMR (DMSO-d_6, 600 MHz): δ 11.57 (br s, 1H), 8.13 (m, 1H), 7.99 (s, 1H), 7.61 (m, 1H), 7.53 (m, 1H), 7.25 (m, 1H), 3.11 (t, J = 7.6 Hz, 2H), 2.72 (s, 3H), 2.50 (m, 2H), 2.25 (s, 3H), 1.75 (m, 2H), 1.55 (m, 2H) ppm. ^{13}C NMR (DMSO-d_6, 150 MHz): δ 140.5, 140.1, 137.7, 134.5, 129.0, 127.3, 125.1, 123.3, 121.1, 119.6, 112.1, 51.5, 36.4, 30.8, 29.3, 27.5, 20.4 ppm. HRMS (ESI) calcd. for $C_{17}H_{21}N_3^+$ $[M]^+$ 267.1735; found 267.1728.

***N*-Methyl-4-(1-methyl-9*H*-β-carbolin-4-yl)butan-1-amine dihydrochloride (2 · 2 HCl)**

Brevicarine dihydrochloride salt was obtained by the addition of cc. HCl to an ethanolic solution of brevicarine (**2**) to give colorless crystals. Mp 192–195 °C (EtOH). (lit. S1 mp. 195–196 °C). IR (ATR): 3385, 3187, 2765, 2468, 1620, 1452, 1407, 1329, 1060, 765, 734 cm^{-1}. ^1H NMR (DMSO-d_6, 600 MHz): δ 11.70 (br s, 1H), 8.56 (br s, 2H), 8.13 (m, 1H), 8.03 (s, 1H), 7.64 (m, 1H), 7.55 (m, 1H), 7.27 (m, 1H), 3.17 (t, J = 7.1 Hz, 2H), 2.91 (t, J = 7.4 Hz, 2H), 2.74 (s, 3H), 2.50 (s, 3H), 1.79 (m, 2H), 1.72 (m, 2H) ppm. ^{13}C NMR (DMSO-d_6, 150 MHz): δ 140.6, 140.3, 137.4, 134.5, 128.4, 127.6, 125.3, 123.4, 121.0, 119.7, 112.2, 48.3, 32.6, 30.2, 26.3, 25.4, 20.2 ppm.

2-[4-(1-Methyl-9*H*-β-carbolin-4-yl)but-3-yn-1-yl]-1*H*-isoindole-1,3-(2*H*)-dione (5)[S2]

Compound **3** (422 mg, 1.28 mmol) was dissolved in EtOAc (25.0 mL). Reagent **4** (636 mg, 3.19 mmol), TEA (0.71 mL, 5.11 mmol), CuI (29.2 mg, 0.15 mmol) and $Pd(PPh_3)_2Cl_2$ (90 mg, 0.13 mmol) were added under argon atmosphere. The mixture was heated to 60 °C and reacted for 3 hours. After that, it was concentrated in vacuo and the residue was purified by silica gel column chromatography (hexane–EtOAc) to give yellow crystals (458 mg, 93%). Mp 245–248 °C (EtOAc) (lit. S2 mp. 245–248 °C (*n*-hexane–DCM)). IR (KBr): 3473, 3186, 2926, 2243, 1716, 1396, 1339, 1295, 1031, 721 cm^{-1} (newly measured and in agreement with lit. S2). ^1H NMR (DMSO-d_6, 600 MHz): δ 11.85 (s, 1H), 8.29 (d, J = 7.6 Hz, 1H), 8.24 (br s, 1H), 7.90 (m, 2H), 7.84 (m, 2H), 7.60 (d, J = 8.8 Hz, 1H), 7.53 (t, J = 7.6 Hz, 1H), 7.01 (t, J = 7.6 Hz, 1H), 3.97 (t, J = 6.6 Hz, 2H), 3.10 (t, J = 7.0 Hz, 2H), 2.77 (s, 3H). ^{13}C NMR (DMSO-d_6, 150 MHz): δ 168.3, 142.1, 141.1, 140.3, 135.0, 132.0, 128.9, 126.6, 123.7, 122.5, 122.2, 120.9, 120.1, 119.9, 112.5, 93.3, 78.7, 46.2, 36.9, 19.3, 9.1. HRMS (ESI) calcd. for $C_{24}H_{18}N_3O_2^+$ $[M+H]^+$ 380.1394; found: 380.1396.

4-(1-Methyl-9*H*-β-carbolin-4-yl)but-3-yn-1-amine (6) [S2]

Compound **5** (286 mg, 0.75 mmol) was dissolved in 40 wt % aqueous $MeNH_2$ (4.72 mL, 54.50 mmol) solution and stirred at room temperature for 30 minutes. After that, 20 wt % aqueous NaOH (1.65 mL) was added and reacted with stirring at room temperature for 2 hours. Afterwards, brine (10 mL) was added. The combined aqueous phase was washed with DCM (3 × 10 mL). The combined organic layer was dried over $NaSO_4$ and concentrated in vacuo. The crude product was purified by silica gel column chromatography (DCM–15 wt % NH_3 in MeOH) to give pale yellow crystals (147 mg, 78%). Mp 166–168 °C (EtOH–H_2O). (lit. S2 mp. 166–168 °C). IR (KBr): 3131, 2987, 1620, 1593, 1421, 1296, 905, 747 cm^{-1} (newly measured and in agreement with lit. S2). ^1H NMR (DMSO-d_6, 600 MHz): δ 11.78 (br s, 1H), 8.53 (d, J = 7.9 Hz, 1H), 8.26 (s, 1H), 7.64 (d, J = 8.2 Hz, 1H), 7.59 (m, 1H), 7.28 (t, J = 7.7 Hz, 1H), 2.92

(t, J = 6.9 Hz, 2H), 2.78 (s, 3H), 2.71 (t, J = 6.8 Hz, 2H) ppm. ^{13}C NMR (DMSO-d_6, 150 MHz): δ 141.8, 140.8, 140.7, 134.0, 128.4, 126.3, 122.5, 121.0, 119.7, 112.2, 110.2, 95.3, 78.0, 41.6, 24.5, 20.6 ppm. HRMS (ESI) calcd. for $C_{16}H_{16}N_3^+$ [M+H]$^+$ 250.1339; found: 250.1343.

2-[4-(1-Methyl-9*H*-β-carbolin-4-yl)butyl]-1*H*-isoindole-1,3(2*H*)-dione (24)

In a 22-mL autoclave, compound **5** (488 mg, 1.29 mmol) was dissolved in a mixture of EtOAc–MeOH (1:1, 15.0 mL), and 10 wt % Pd/C (150 mg, 0.141 mmol) was added. The autoclave was purged with argon gas. After that, it was washed with hydrogen gas three times and the starting pressure was adjusted to 20 bar. The mixture was reacted at room temperature while stirring for 16 hours. After the reaction was complete, the mixture was filtered through celite and the filter pad was washed with EtOAc–MeOH (1:1, 3 × 10 mL). It was concentrated in vacuo and the residue was purified by silica gel column chromatography (hexane–EtOAc) to give yellow crystals (353 mg, 72%). Mp 107–110 °C (EtOH). IR (KBr): 3377, 1706, 1620, 1396, 1030, 718 cm^{-1}. ^{1}H NMR (DMSO-d_6, 600 MHz): δ 11.56 (br s, 1H), 8.04 (m, 1H), 7.99 (s, 1H), 7.83 (m, 2H), 7.82 (m, 2H), 7.60 (m, 1H), 7.49 (m, 1H), 7.15 (m, 1H), 3.62 (t, J = 6.4 Hz, 2H), 3.13 (t, J = 6.9 Hz, 2H), 2.71 (s, 3H), 1.76 (m, 2H), 1.75 (m, 2H) ppm. ^{13}C NMR (DMSO-d_6, 150 MHz): δ 168.2, 140.5, 140.3, 137.7, 134.6, 131.7, 128.5, 127.3, 125.0, 123.2, 123.1, 121.0, 119.5, 112.2, 48.8, 37.5, 30.3, 28.0, 26.8, 20.4 ppm. HRMS (ESI) calcd. for $C_{24}H_{22}N_3O_2^+$ [M+H]$^+$ 384.1707; found: 384.1713.

4-(1-Methyl-9*H*-β-carbolin-4-yl)butan-1-amine (25)

Method A: Compound **24** (337 mg, 0.88 mmol) was dissolved in 40 wt % aqueous $MeNH_2$ (7.0 mL, 66.7 mmol) solution and stirred at room temperature for 30 minutes. After that, 20 wt % aqueous NaOH (7.0 mL) was added, and the mixture was reacted at room temperature for 2 hours. Afterwards, brine (100.0 mL) was added, and the combined aqueous phases were extracted with DCM (3 × 150 mL). The combined organic layer was dried over Na_2SO_4 and

concentrated in vacuo. The crude product was purified by silica gel column chromatography (DCM–15 wt % NH_3 in MeOH) to give pale yellow crystals (182 mg, 82%).

Method B: In a 22-mL autoclave, compound **6** (185 mg, 0.74 mmol) was dissolved in EtOH (15.0 mL) and 10 wt % Pd/C (77 mg, 0.07 mmol) was added. The autoclave was purged with argon gas. After that, it was washed with hydrogen gas three times and the starting pressure was adjusted to 20 bar. The mixture was reacted at room temperature for 16 hours. After the reaction was complete, the mixture was filtered through celite, and the filter pad was washed with EtOH (3 × 10 mL). It was concentrated in vacuo and the residue was purified by silica gel column chromatography (DCM–15 wt % NH_3 in MeOH) to give pale yellow crystals (165 mg, 88%). Mp 101–103 °C (MeCN). IR (ATR): 3058, 2923, 2859, 1620, 1570, 1507, 1455, 1394, 1323, 1293, 1030 cm^{-1}. ^{1}H NMR (DMSO-d_6, 600 MHz): δ 11.57 (s, 1H), 8.11 (d, J = 8.0 Hz, 1H), 7.99 (s, 1H), 7.61 (d, J = 8.2 Hz, 1H), 7.53 (t, J = 8.1 Hz, 1H), 7.25 (t, J = 7.5 Hz, 1H), 3.11 (t, J = 7.6 Hz, 2H), 2.72 (s, 3H), 2.58 (t, J = 7.0 Hz, 2H), 1.75 (m, 2H), 1.50 (m, 2H) ppm. ^{13}C NMR (DMSO-d_6, 150 MHz): δ 140.5, 140.1, 137.7, 134.5, 129.0, 127.3, 125.1, 123.3, 121.1, 119.6, 112.1, 41.6, 33.1, 30.7, 27.2, 20.4 ppm. HRMS (ESI) calcd. for $C_{16}H_{19}N_3^+$ $[M]^+$ 253.1573; found 253.1570.

***N*-[4-(1-Methyl-9*H*-β-carbolin-4-yl)butyl]formamide (26)**

Method A: Compound **25** (80 mg, 0.32 mmol) was dissolved in toluene (5.0 mL) and formic acid (15.0 µL, 0.38 mmol) was added. The mixture was heated to reflux and reacted for 16 hours. After concentration in vacuo, the residue was dissolved in EtOAc (10.0 mL), washed with 10 wt % aqueous $NaHCO_3$ solution (10.0 mL). The aqueous phase was extracted with EtOAc (2 × 15 mL). The combined organic layer was dried over Na_2SO_4 and concentrated in vacuo to give colorless crystals. The crude product was purified by preparative TLC (DCM–MeOH) to give colorless crystals (61 mg, 69%).

Method B: Compound **28** (10.0 mg, 0.03 mmol) was dissolved in dry THF (10.0 mL) and cooled to 0 °C. Under argon atmosphere, $LiAlH_4$ (3.50 mg, 0.09 mmol) was added slowly. After that, the mixture was heated to reflux and stirred for 1 hour. The reaction cooled to 0 °C and the excess reagent was hydrolyzed by carefully adding water (4 × 10 mL) and 15 wt % NaOH solution (10.0 mL). The mixture was stirred for 20 minutes and additional THF (30.0 mL) was added and filtered through celite, and the filter pad was washed with THF (50.0 mL). The organic layer was dried over Na_2SO_4 and concentrated in vacuo to give a yellow oil. The crude product was purified by preparative TLC (DCM–MeOH) to give colorless crystals (4.4 mg, 51%). Mp 121–123 °C (MeCN). IR (KBr): 3472, 1665, 1564, 1508, 1381, 907, 731 cm^{-1}. ^1H NMR (DMSO-d_6, 600 MHz): δ 11.58 (br s, 1H), 8.10 (d, J = 8.0 Hz, 1H), 8.01 (br s, 1H), 8.00 (s, 1H), 7.97 (s, 1H), 7.62 (m, 1H), 7.53 (t, J = 7.7 Hz, 1H), 7.26 (t, J = 7.6 Hz, 1H), 3.13 (m, 2H), 3.12 (m, 2H) 2.72 (s, 3H), 1.73 (m, 2H), 1.56 (m, 2H) ppm. ^{13}C NMR (DMSO-d_6, 150 MHz): δ 161.1, 140.5, 140.2, 137.7, 134.5, 128.8, 127.4, 125.1, 123.3, 121.1, 119.6, 112.1, 37.1, 30.4, 29.1, 27.0, 20.4 ppm. HRMS (ESI) calcd. for $C_{17}H_{19}N_3O^+$ $[M]^+$ 281.1528; found 281.1523.

***N*,*N*-Dimethyl-4-(1-methyl-9*H*-β-carbolin-4-yl)butan-1-amine (27)**

Compound **25** (165 mg, 0.65 mmol) was dissolved in formic acid (2.33 mL, 61.7 mmol) and 35 wt % aqueous CH_2O solution (102 µL, 1.30 mmol) was added. The mixture was heated to 90 °C and reacted for 5 hours. The mixture was added to 10 wt % aqueous $NaHCO_3$ (47.0 mL) and extracted with DCM (3 × 25 mL). The combined organic layer was dried over Na_2SO_4 and concentrated in vacuo. The residue was purified by silica gel column chromatography (DCM–MeOH) to give pale yellow crystals (118 mg, 65%). Mp 189–192 °C (EtOH). (lit. S1 mp. 128–129 °C (benzene)). IR (film): 3422, 2940, 1664, 1622, 1458, 1325, 736 cm^{-1}. ^1H NMR (DMSO-d_6, 600 MHz): δ 11.62 (br s, 1H), 8.16 (m, 1H), 8.01 (s, 1H), 7.62 (m, 1H), 7.53 (m, 1H), 7.26 (m, 1H), 3.13 (t, J = 7.6 Hz, 2H), 2.73 (s, 3H), 2.51 (m, 2H), 2.30 (s, 6H), 1.75 (m, 2H), 1.64

(m, 2H) ppm. ^{13}C NMR (DMSO-d_6, 150 MHz): δ 140.5, 140.2, 137.7, 134.5, 128.8, 127.4, 125.1, 123.4, 121.1, 119.6, 112.1, 58.1, 48.8, 44.4, 30.5, 27.1, 26.0, 20.4 ppm. HRMS (ESI) calcd. for $C_{18}H_{23}N_3^+$ $[M]^+$ 281.1892; found: 281.1891.

Ethyl [4-(1-methyl-9*H*-β-carbolin-4-yl)butyl]carbamate (28)

Compound **25** (20.0 mg, 0.08 mmol) was dissolved in $CHCl_3$ (1.0 mL) and cooled to 0 °C. While stirring, ethyl chloroformate (7.50 µL, 0.08 mmol) and 4.0 M aqueous solution of NaOH (20.0 µL, 0.08 mmol) was added. The mixture was reacted for 3 hours at room temperature, then washed with water (5.0 mL). The aqueous phase was extracted with DCM (3 × 15 mL). The combined organic layers were dried over Na_2SO_4 and concentrated in vacuo to give brown oil. The crude product was purified by silica gel column chromatography (DCM–MeOH) to give yellow crystals (20 mg, 78%). Mp 155–157 °C (MeCN). IR (KBr): 3420, 1694, 1662, 1540, 1457, 1411, 1325, 739 cm^{-1}. ^{1}H NMR (DMSO-d_6, 600 MHz): δ 11.58 (br s, 1H), 8.09 (m, 1H), 8.00 (s, 1H), 7.62 (m, 1H), 7.53 (m, 1H), 7.25 (m, 1H), 7.09 (br t, J = 5.4 Hz, 1H), 3.94 (q, J = 7.1 Hz, 2H), 3.11 (t, J = 7.5 Hz, 2H) 3.02 (q, J = 6.4 Hz, 2H), 2.73 (s, 3H), 1.72 (m, 2H), 1.55 (m, 2H), 1.12 (t, J = 7.1 Hz, 3H) ppm. ^{13}C NMR (DMSO-d_6, 150 MHz): δ 156.5, 140.5, 140.1, 137.7, 134.5, 128.8, 127.4, 125.1, 123.3, 121.1, 119.6, 112.1, 59.6, 40.2, 30.5, 29.6, 26.9, 20.4, 14.9 ppm. HRMS (ESI) calcd. for $C_{19}H_{23}N_3O_2^+$ $[M]^+$ 325.1782; found 325.1777.

1-Methyl-4-(1-methyl-1*H*-pyrrol-2-yl)-9*H*-β-carboline (30)

Compound **3** (500 mg, 1.50 mmol) was dissolved in 1,2-dimethoxyethane solvent (150.0 mL). 2.0 M aqueous solution of Na_2CO_3 (80.0 mL) and reagent **29** (625 mg, 3.0 mmol) was added. The mixture was heated under argon atmosphere to 50 °C and stirred at this temperature for 5 minutes. $Pd(PPh_3)_4$ (15 mg, 0.01 mmol) was added, the mixture was heated to reflux and reacted for 1 hour. After the reaction was complete, it was concentrated in vacuo and extracted with DCM (3 × 200 mL). The combined organic layer was dried over Na_2SO_4 and concentrated in

vacuo. The crude product was purified by silica gel column chromatography (DCM–MeOH) to give pale yellow crystals (396 mg, 99%). Mp 216–218 °C (EtOH). IR (KBr): 3158, 1618, 1381, 1318, 711 cm^{-1}. ^{1}H NMR (DMSO-d_6, 600 MHz): δ 11.75 (br s, 1H), 8.09 (s, 1H), 7.62 (d, J = 8.2 Hz, 1H), 7.50 (t, J = 7.4 Hz, 1H), 7.15 (d, J = 7.9 Hz, 1H), 7.09 (t, J = 7.3 Hz, 1H), 7.04 (s, 1H), 6.24 (m, 1H), 6.19 (m, 1H), 3.32 (s, 3H), 2.82 (s, 3H) ppm. ^{13}C NMR (DMSO-d_6, 150 MHz): δ 142.0, 140.7, 138.8, 134.2, 129.5, 128.0, 126.5, 123.5, 122.0, 120.9, 120.1, 119.6, 112.3, 109.2, 107.8, 34.0, 20.6 ppm. HRMS (ESI) calcd. for $C_{17}H_{16}N_3^+$ [M+H]$^+$ 262.1339; found 262.1343.

1-Methyl-4-(1-methylpyrrolidin-2-yl)-6,7,8,9-tetrahydro-5*H*-β-carboline (31)

In a 22-mL autoclave, compound **30** (48.0 mg, 0.18 mmol) was dissolved in a mixture of MeOH–H_2O (4:1, 10 mL) and 37 wt % aqueous HCl solution (46 µL, 0.55 mmol), $PtO_2 \cdot H_2O$ (90 mg, 0.29 mmol) was added at room temperature. The autoclave was purged with argon gas. After that, it was washed with hydrogen gas three times and the starting pressure was adjusted to 15 bar. The mixture was reacted at room temperature for 24 hours. After the reaction was complete, the mixture was filtered through celite and the filter pad was washed with MeOH (4 × 15 mL). It was concentrated in vacuo, the residue was dissolved in DCM (50 mL), washed with 10 wt % aqueous $NaHCO_3$ (10 mL) and extracted with DCM (2 × 20 mL). The combined organic layer was dried over Na_2SO_4 and concentrated in vacuo to give yellow crystals (45 mg, 91%). An analytical sample was recrystallized from MeCN. Mp 242–245 °C (MeCN). (lit. S3 mp. 234–237 °C (acetone)). IR (KBr): 3429, 3045, 2930, 2843, 1559, 1444, 1312, 1049 cm^{-1}. ^{1}H NMR (DMSO-d_6, 600 MHz): δ 11.03 (br s, 1H), 7.96 (s, 1H), 3.52 (t, J = 8.3 Hz, 1H), 3.18 (t, J = 8.0 Hz, 1H), 2.90 (m, 1H), 2.79 (m, 1H), 2.74 (m, 2H), 2.54 (s, 3H), 2.22 (m, 1H), 2.20 (m, 1H), 2.11 (s, 3H), 1.83 (m, 1H), 1.83 (m, 1H), 1.82 (m, 1H), 1.78 (m, 1H), 1.76 (m, 1H), 1.76 (m, 1H), 1.60 (m, 1H) ppm. ^{13}C NMR (DMSO-d_6, 150 MHz): δ 139.7, 138.2, 135.2, 131.1,

129.6, 127.5, 108.2, 66.6, 56.7, 40.8, 35.8, 23.9, 23.6, 23.4, 22.4, 22.2, 20.0 ppm. HRMS (ESI) calcd. for $C_{17}H_{23}N_3^+$ $[M]^+$ 269.1885; found 269.1885.

1-Methyl-4-[1-methyl-5-(2,2,2-trifluoroethyl)-1*H*-pyrrol-2-yl]-9*H*-β-carboline (32)

Compound **30** (196 mg, 0.75 mmol) was dissolved in TFA (10.0 mL, 130.0 mmol) and $NaCNBH_3$ (800 mg, 12.7 mmol) was added. The mixture was stirred at room temperature for 2 hours, it was poured onto ice and treated with 10 wt % aqueous $NaHCO_3$ solution (100.0 mL) until pH 7. It was extracted with DCM (3 × 200 mL). The combined organic layer was dried over Na_2SO_4 and concentrated in vacuo. The crude product was purified by silica gel column chromatography (DCM–MeOH) to give yellow crystals (68 mg, 36%). Mp 246–248 °C (MeCN). IR (KBr): 1621, 1457, 1254, 1137, 1071, 739 cm^{-1}. ^{1}H NMR (DMSO-d_6, 600 MHz): δ 11.78 (br s, 1H), 8.09 (s, 1H), 7.62 (m, 1H), 7.50 (m, 1H), 7.08 (m, 1H), 7.05 (m, 1H), 6.31 (d, J = 3.5 Hz, 1H), 6.19 (d, J = 3.5 Hz, 1H), 3.90 (d, J = 11.1 Hz, 1H), 3.87 (d, J = 11.1 Hz, 1H), 3.24 (s, 3H), 2.82 (s, 3H) ppm. ^{13}C NMR (DMSO-d_6, 150 MHz): δ 142.2, 140.8, 138.7, 134.1, 130.9, 128.1, 126.5, 126.2, 122.8, 121.9, 120.8, 120.1, 119.6, 112.3, 110.2, 108.4, 31.3, 30.9, 20.6 ppm. ^{19}F NMR (DMSO-d_6, 564 MHz): −64.5 ppm. HRMS (ESI) calcd. for $C_{19}H_{17}F_3N_3^+$ $[M+H]^+$ 344.1369; found: 344.1374.

References

S1. Terent'eva, I. V.; Lazur'evskii, G. V.; Shirshova, T. I. *Chem. Nat. Compd.*, **1969**, *5*, 330–334. doi: 10.1007/BF00595071

S2. Szabó, T.; Görür, F. L.; Horváth, S.; Volk, B.; Milen, M. *Synthesis*, **2022**, *54*, 3867–3873. doi: 10.1055/s-0041-1737830

S3. Lazurjevski, G.; Terentjeva, I. *Heterocycles*, **1976**, *4*, 1783–1816.

2

Batizi Benedek	KP
Gyemant ATR	10/3/2024

BRUKER Alpha
Resolution: 2 cm-1
Number of Scans: 32

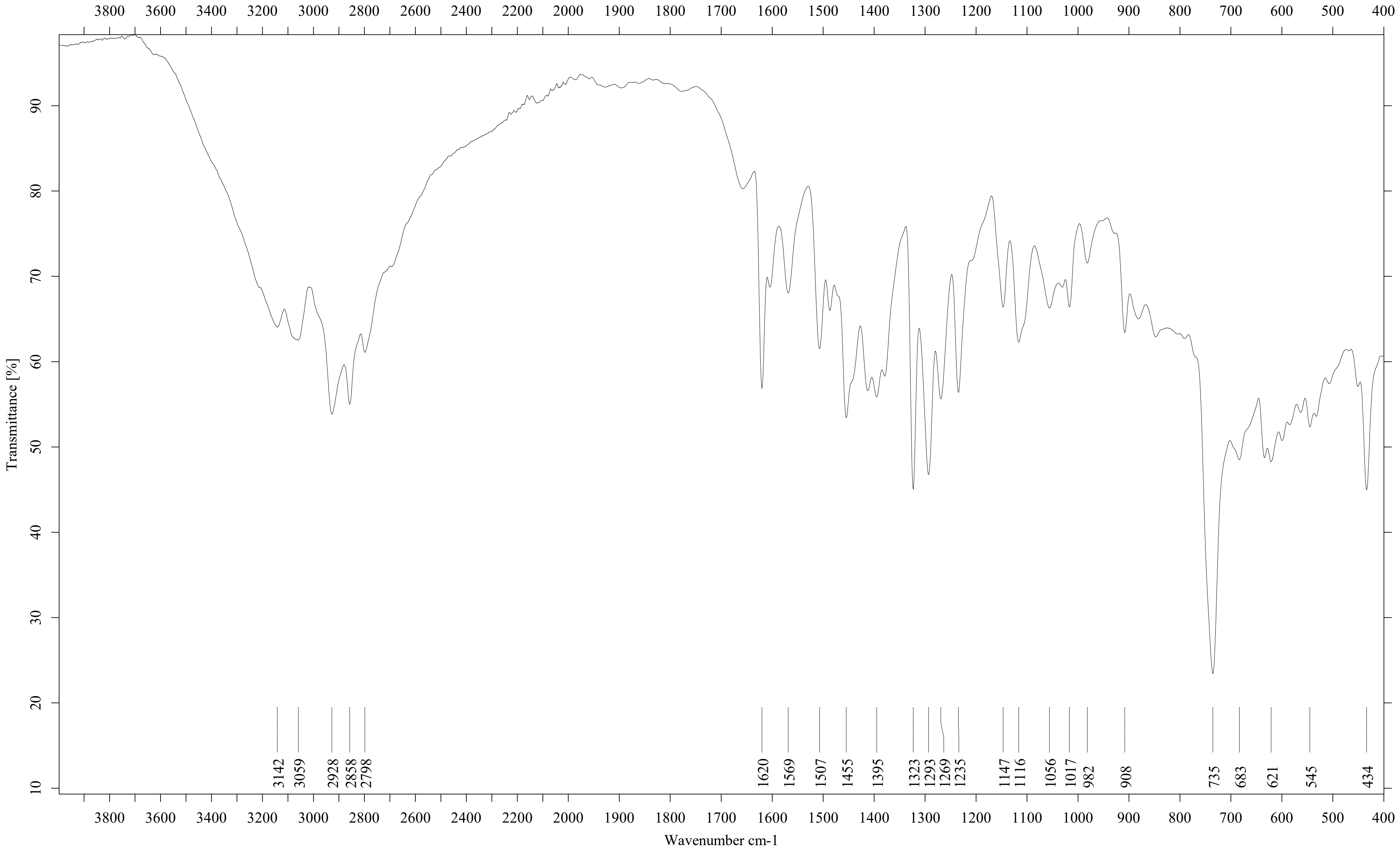

2

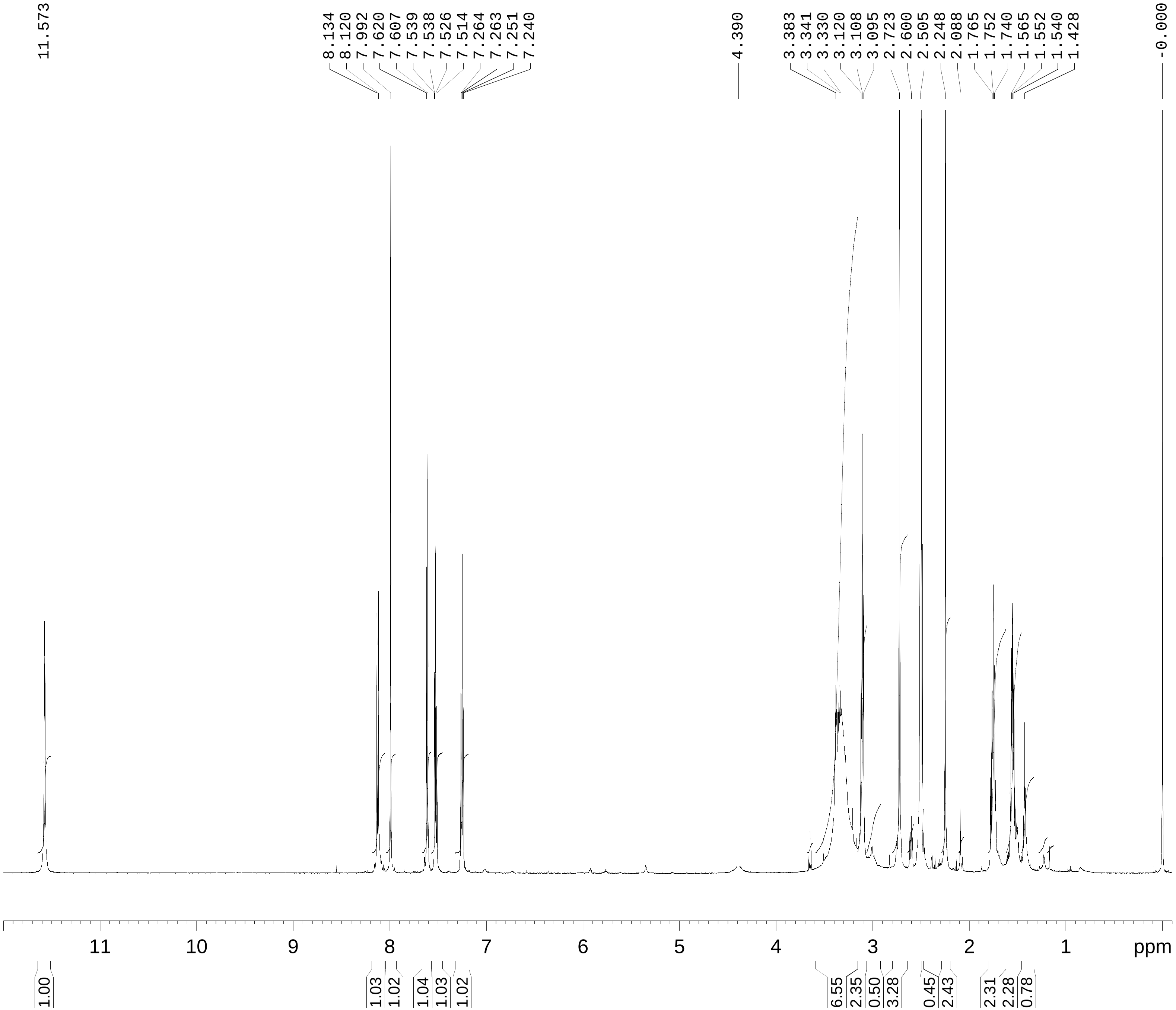

Standard 1H
143648
BAB0325_1A
Batizi Benedek
2024.10.03. (KP)

Current Data Parameters	
NAME	143648
EXPNO	11
PROCNO	1

F2 - Acquisition Parameters	
Date_	20241003
Time	15.48 h
INSTRUM	spect
PROBHD	Z145856_0002 (
PULPROG	zg30
TD	65536
SOLVENT	DMSO
NS	16
DS	2
SWH	12019.230 Hz
FIDRES	0.366798 Hz
AQ	2.7262976 sec
RG	196.07
DW	41.600 usec
DE	25.00 usec
TE	295.0 K
D1	1.00000000 sec
TD0	1
SFO1	600.0037050 MHz
NUC1	1H
P1	11.50 usec
PLW1	28.00000000 W

F2 - Processing parameters	
SI	65536
SF	600.0000020 MHz
WDW	EM
SSB	0
LB	0.30 Hz
GB	0
PC	1.00

2

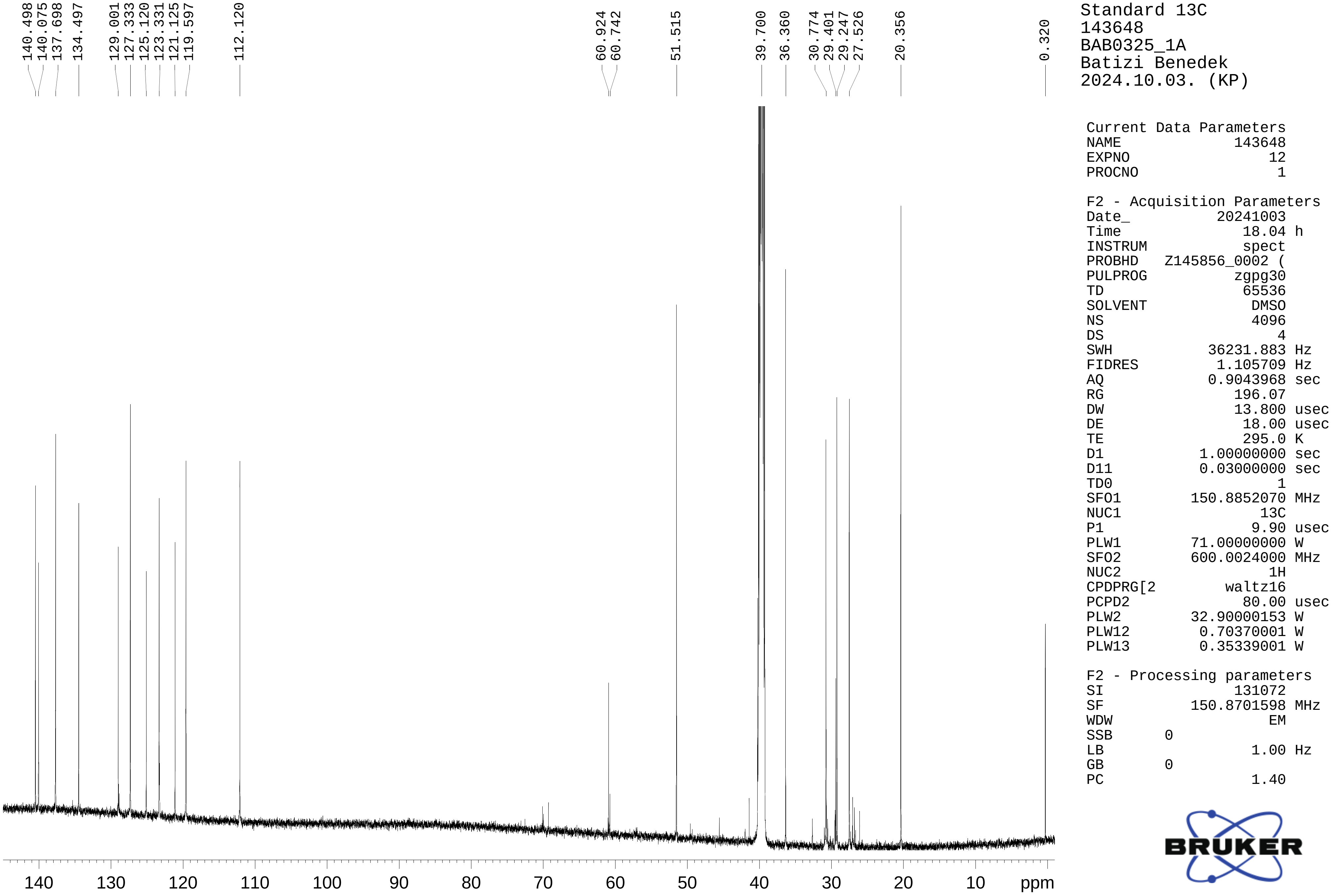
140.498
140.075
137.698
134.497
129.001
127.333
125.120
123.331
121.125
119.597
112.120
60.924
60.742
51.515
39.700
36.360
30.774
29.401
29.247
27.526
20.356
0.320
Standard 13C
143648
BAB0325_1A
Batizi Benedek
2024.10.03. (KP)
Current Data Parameters
NAME 143648
EXPNO 12
PROCNO 1
F2 - Acquisition Parameters
Date_ 20241003
Time 18.04 h
INSTRUM spect
PROBHD Z145856_0002 (
PULPROG zgpg30
TD 65536
SOLVENT DMSO
NS 4096
DS 4
SWH 36231.883 Hz
FIDRES 1.105709 Hz
AQ 0.9043968 sec
RG 196.07
DW 13.800 usec
DE 18.00 usec
TE 295.0 K
D1 1.00000000 sec
D11 0.03000000 sec
TD0 1
SFO1 150.8852070 MHz
NUC1 13C
P1 9.90 usec
PLW1 71.00000000 W
SFO2 600.0024000 MHz
NUC2 1H
CPDPRG[2 waltz16
PCPD2 80.00 usec
PLW2 32.90000153 W
PLW12 0.70370001 W
PLW13 0.35339001 W
F2 - Processing parameters
SI 131072
SF 150.8701598 MHz
WDW EM
SSB 0
LB 1.00 Hz
GB 0
PC 1.40
140
130
120
110
100
90
80
70
60
50
40
30
20
10
ppm

2

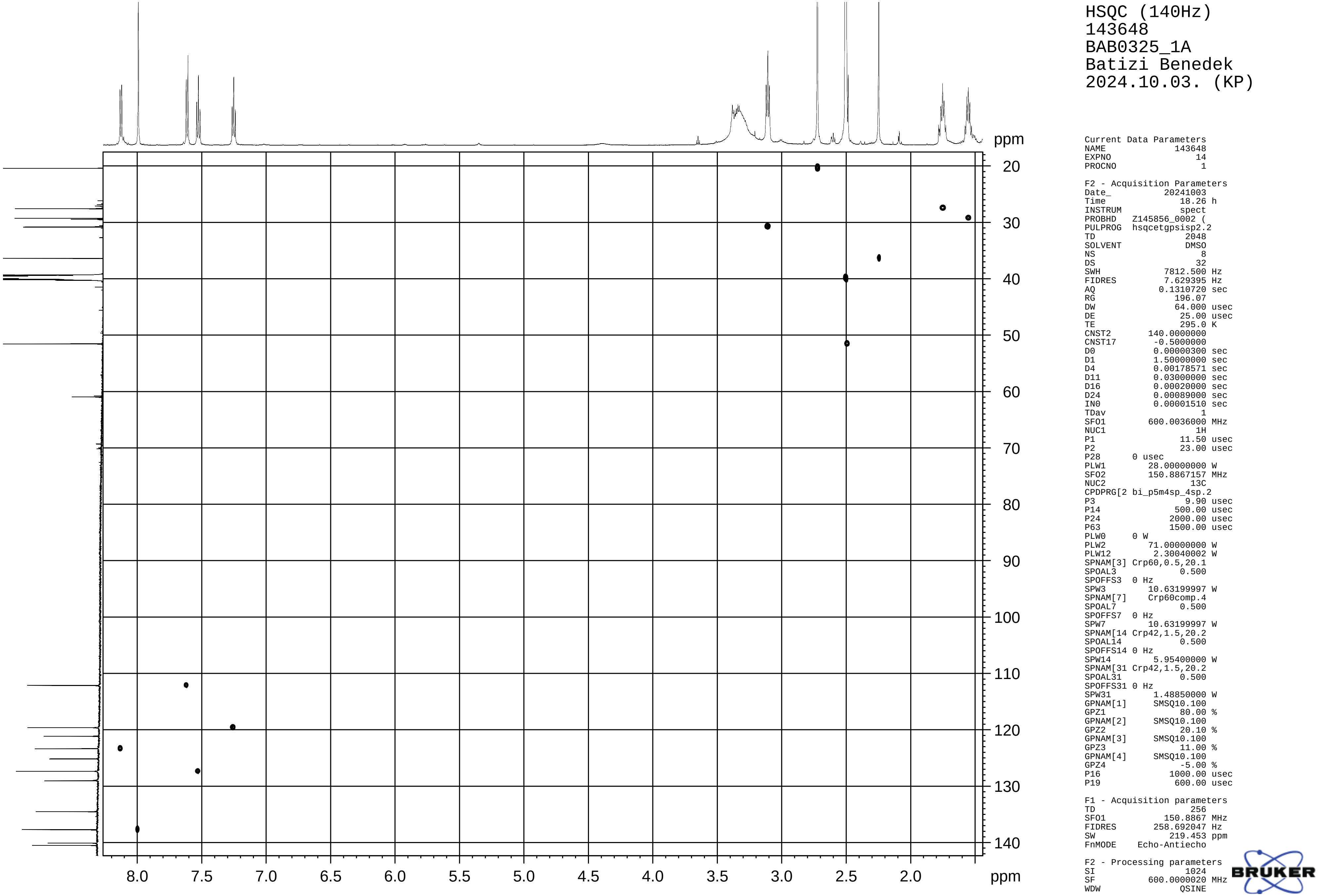

HSQC (140Hz)
143648
BAB0325_1A
Batizi Benedek
2024.10.03. (KP)
Current Data Parameters
NAME 143648
EXPNO 14
PROCNO 1
F2 - Acquisition Parameters
Date_ 20241003
Time 18.26 h
INSTRUM spect
PROBHD Z145856_0002 (
PULPROG hsqcetgpsisp2.2
TD 2048
SOLVENT DMSO
NS 8
DS 32
SWH 7812.500 Hz
FIDRES 7.629395 Hz
AQ 0.1310720 sec
RG 196.07
DW 64.000 usec
DE 25.00 usec
TE 295.0 K
CNST2 140.0000000
CNST17 -0.5000000
D0 0.00000300 sec
D1 1.50000000 sec
D4 0.00178571 sec
D11 0.03000000 sec
D16 0.00020000 sec
D24 0.00089000 sec
IN0 0.00001510 sec
TDav 1
SFO1 600.0036000 MHz
NUC1 1H
P1 11.50 usec
P2 23.00 usec
P28 0 usec
PLW1 28.00000000 W
SFO2 150.8867157 MHz
NUC2 13C
CPDPRG[2 bi_p5m4sp_4sp.2
P3 9.90 usec
P14 500.00 usec
P24 2000.00 usec
P63 1500.00 usec
PLW0 0 W
PLW2 71.00000000 W
PLW12 2.30040002 W
SPNAM[3] Crp60,0.5,20.1
SPOAL3 0.500
SPOFFS3 0 Hz
SPW3 10.63199997 W
SPNAM[7] Crp60comp.4
SPOAL7 0.500
SPOFFS7 0 Hz
SPW7 10.63199997 W
SPNAM[14 Crp42,1.5,20.2
SPOAL14 0.500
SPOFFS14 0 Hz
SPW14 5.95400000 W
SPNAM[31 Crp42,1.5,20.2
SPOAL31 0.500
SPOFFS31 0 Hz
SPW31 1.48850000 W
GPNAM[1] SMSQ10.100
GPZ1 80.00 %
GPNAM[2] SMSQ10.100
GPZ2 20.10 %
GPNAM[3] SMSQ10.100
GPZ3 11.00 %
GPNAM[4] SMSQ10.100
GPZ4 -5.00 %
P16 1000.00 usec
P19 600.00 usec
F1 - Acquisition parameters
TD 256
SFO1 150.8867 MHz
FIDRES 258.692047 Hz
SW 219.453 ppm
FnMODE Echo-Antiecho
F2 - Processing parameters
SI 1024
SF 600.0000020 MHz
WDW QSINE
ppm
20
30
40
50
60
70
80
90
100
110
120
130
140
8.0
7.5
7.0
6.5
6.0
5.5
5.0
4.5
4.0
3.5
3.0
2.5
2.0
ppm

2

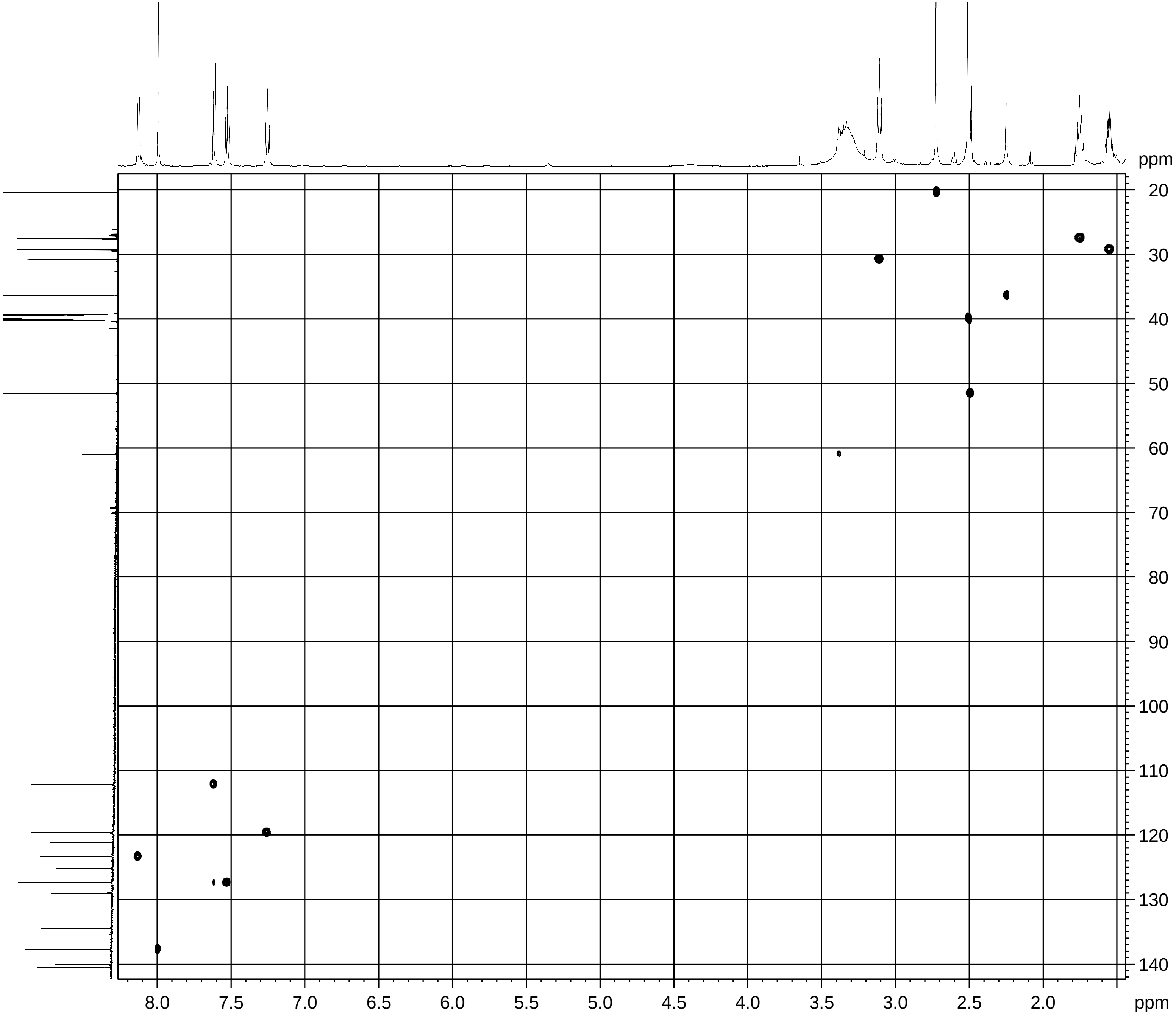

HSQC (140Hz)
143648
BAB0325_1A
Batizi Benedek
2024.10.03. (KP)

```
Current Data Parameters
NAME            143648
EXPNO               14
PROCNO               1

F2 - Acquisition Parameters
Date_         20241003
Time             18.26 h
INSTRUM          spect
PROBHD   Z145856_0002 (
PULPROG  hsqcetgpsisp2.2
TD                2048
SOLVENT           DMSO
NS                   8
DS                  32
SWH           7812.500 Hz
FIDRES        7.629395 Hz
AQ           0.1310720 sec
RG              196.07
DW              64.000 usec
DE               25.00 usec
TE               295.0 K
CNST2      140.0000000
CNST17      -0.5000000
D0          0.00000300 sec
D1          1.50000000 sec
D4          0.00178571 sec
D11         0.03000000 sec
D16         0.00020000 sec
D24         0.00089000 sec
IN0         0.00001510 sec
TDav                 1
SFO1       600.0036000 MHz
NUC1                1H
P1               11.50 usec
P2               23.00 usec
P28      0 usec
PLW1       28.00000000 W
SFO2       150.8867157 MHz
NUC2               13C
CPDPRG[2 bi_p5m4sp_4sp.2
P3                9.90 usec
P14             500.00 usec
P24            2000.00 usec
P63            1500.00 usec
PLW0     0 W
PLW2       71.00000000 W
PLW12       2.30040002 W
SPNAM[3] Crp60,0.5,20.1
SPOAL3           0.500
SPOFFS3  0 Hz
SPW3       10.63199997 W
SPNAM[7]    Crp60comp.4
SPOAL7           0.500
SPOFFS7  0 Hz
SPW7       10.63199997 W
SPNAM[14 Crp42,1.5,20.2
SPOAL14          0.500
SPOFFS14 0 Hz
SPW14       5.95400000 W
SPNAM[31 Crp42,1.5,20.2
SPOAL31          0.500
SPOFFS31 0 Hz
SPW31       1.48850000 W
GPNAM[1]     SMSQ10.100
GPZ1             80.00 %
GPNAM[2]     SMSQ10.100
GPZ2             20.10 %
GPNAM[3]     SMSQ10.100
GPZ3             11.00 %
GPNAM[4]     SMSQ10.100
GPZ4             -5.00 %
P16            1000.00 usec
P19             600.00 usec

F1 - Acquisition parameters
TD                 256
SFO1          150.8867 MHz
FIDRES      258.692047 Hz
SW             219.453 ppm
FnMODE    Echo-Antiecho

F2 - Processing parameters
SI                1024
SF         600.0000020 MHz
WDW              QSINE
```

BRUKER

2

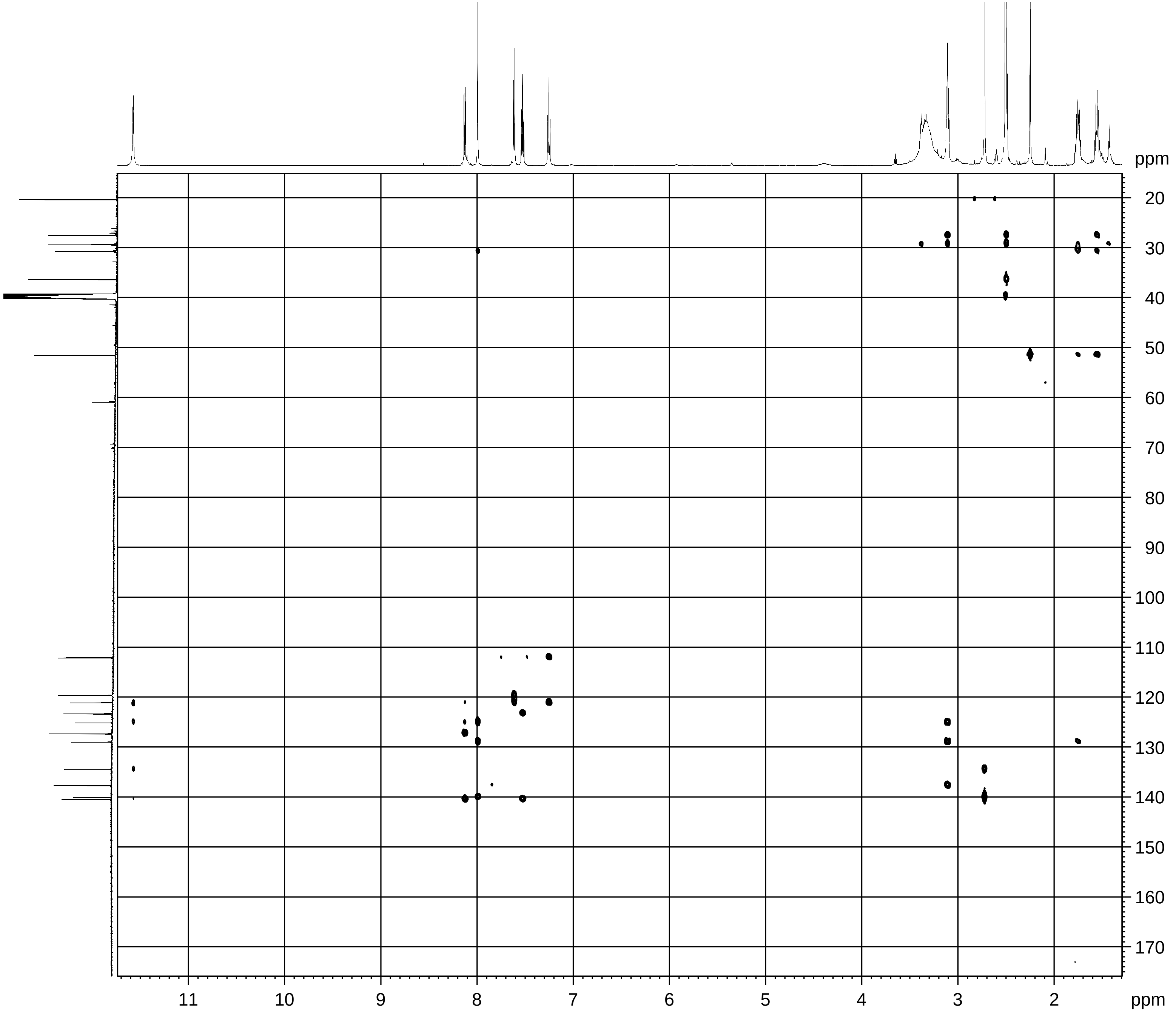

HMBC (8Hz, 140Hz)
143648
BAB0325_1A
Batizi Benedek
2024.10.03. (KP)

```
Current Data Parameters
NAME              143648
EXPNO                 15
PROCNO                 1

F2 - Acquisition Parameters
Date_          20241003
Time              19.24 h
INSTRUM           spect
PROBHD   Z145856_0002 (
PULPROG    hmbcgplpndqf
TD                 2048
SOLVENT            DMSO
NS                    8
DS                   16
SWH            7812.500 Hz
FIDRES         7.629395 Hz
AQ            0.1310720 sec
RG               196.07
DW               64.000 usec
DE                25.00 usec
TE                295.0 K
CNST2       140.0000000
CNST13        8.0000000
D0           0.00000300 sec
D1           1.50000000 sec
D2           0.00357143 sec
D6           0.06250000 sec
D16          0.00020000 sec
IN0          0.00001510 sec
TDav                  1
SFO1        600.0037800 MHz
NUC1                 1H
P1                11.50 usec
P2                23.00 usec
PLW1        28.00000000 W
SFO2        150.8867157 MHz
NUC2                13C
P3                 9.90 usec
PLW2        71.00000000 W
GPNAM[1]     SMSQ10.100
GPZ1              50.00 %
GPNAM[2]     SMSQ10.100
GPZ2              30.00 %
GPNAM[3]     SMSQ10.100
GPZ3              40.10 %
P16             1000.00 usec

F1 - Acquisition parameters
TD                  256
SFO1           150.8867 MHz
FIDRES       258.692047 Hz
SW              219.453 ppm
FnMODE               QF

F2 - Processing parameters
SI                 2048
SF          600.0000020 MHz
WDW                SINE
SSB      0
LB       0 Hz
GB       0
PC                 1.40

F1 - Processing parameters
SI                 1024
MC2                  QF
SF          150.8701598 MHz
WDW                SINE
SSB      0
LB       0 Hz
GB       0
```

2 · 2 HCl

Batizi Benedek	KP
Gyemant ATR	11/8/2024

BRUKER Alpha
Resolution: 2 cm-1
Number of Scans: 32

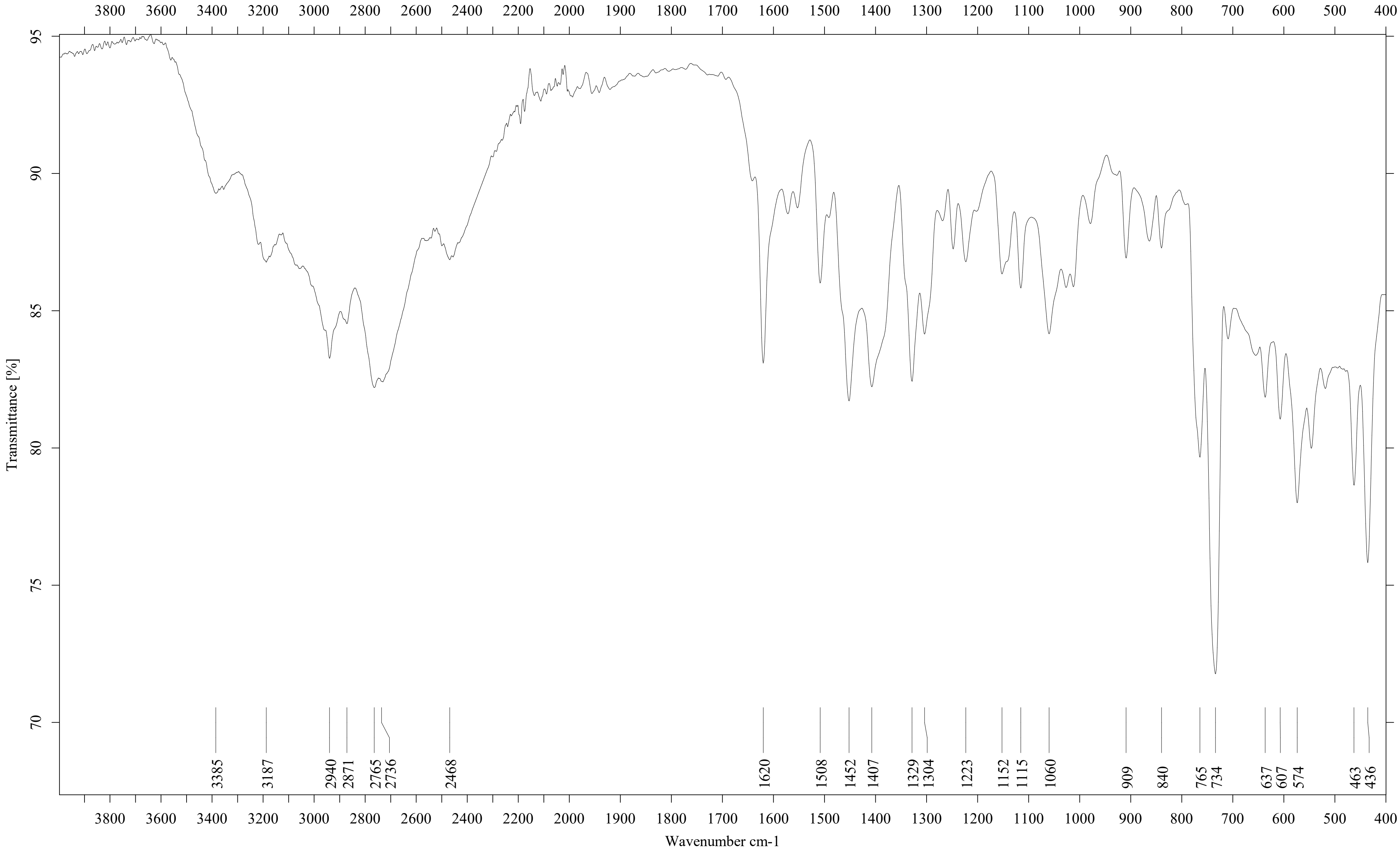

2 · 2 HCl

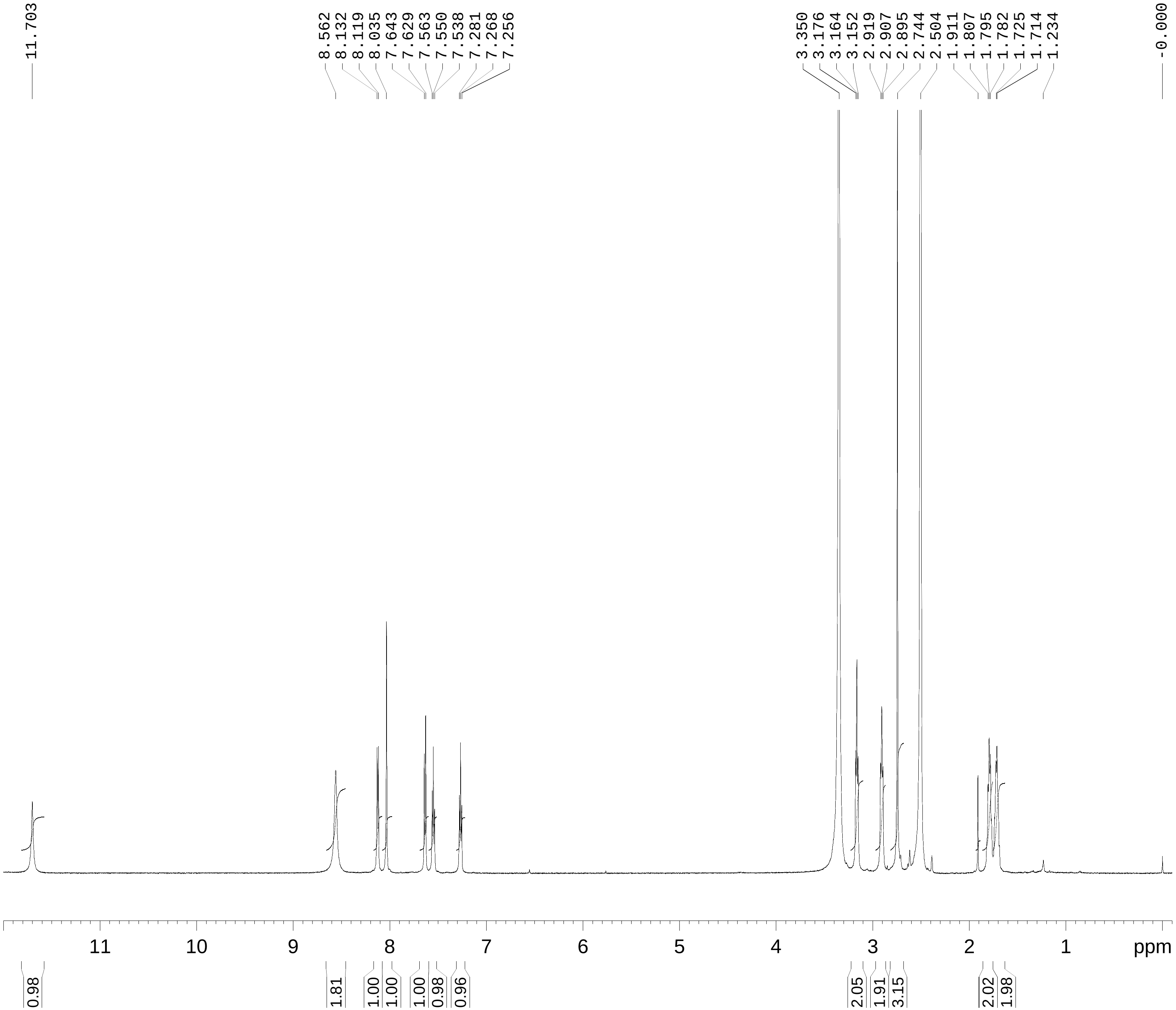

Standard 1H
143819
BAB0352_1
Batizi Benedek
2024.11.08. (KP)

Current Data Parameters	
NAME	143819
EXPNO	11
PROCNO	1

F2 - Acquisition Parameters	
Date_	20241109
Time	2.50 h
INSTRUM	spect
PROBHD	Z145856_0002 (
PULPROG	zg30
TD	65536
SOLVENT	DMSO
NS	16
DS	2
SWH	12019.230 Hz
FIDRES	0.366798 Hz
AQ	2.7262976 sec
RG	196.07
DW	41.600 usec
DE	25.00 usec
TE	295.0 K
D1	1.00000000 sec
TD0	1
SFO1	600.0037050 MHz
NUC1	1H
P1	11.50 usec
PLW1	28.00000000 W

F2 - Processing parameters	
SI	65536
SF	600.0000023 MHz
WDW	EM
SSB	0
LB	0.30 Hz
GB	0
PC	1.00

2 · 2 HCl

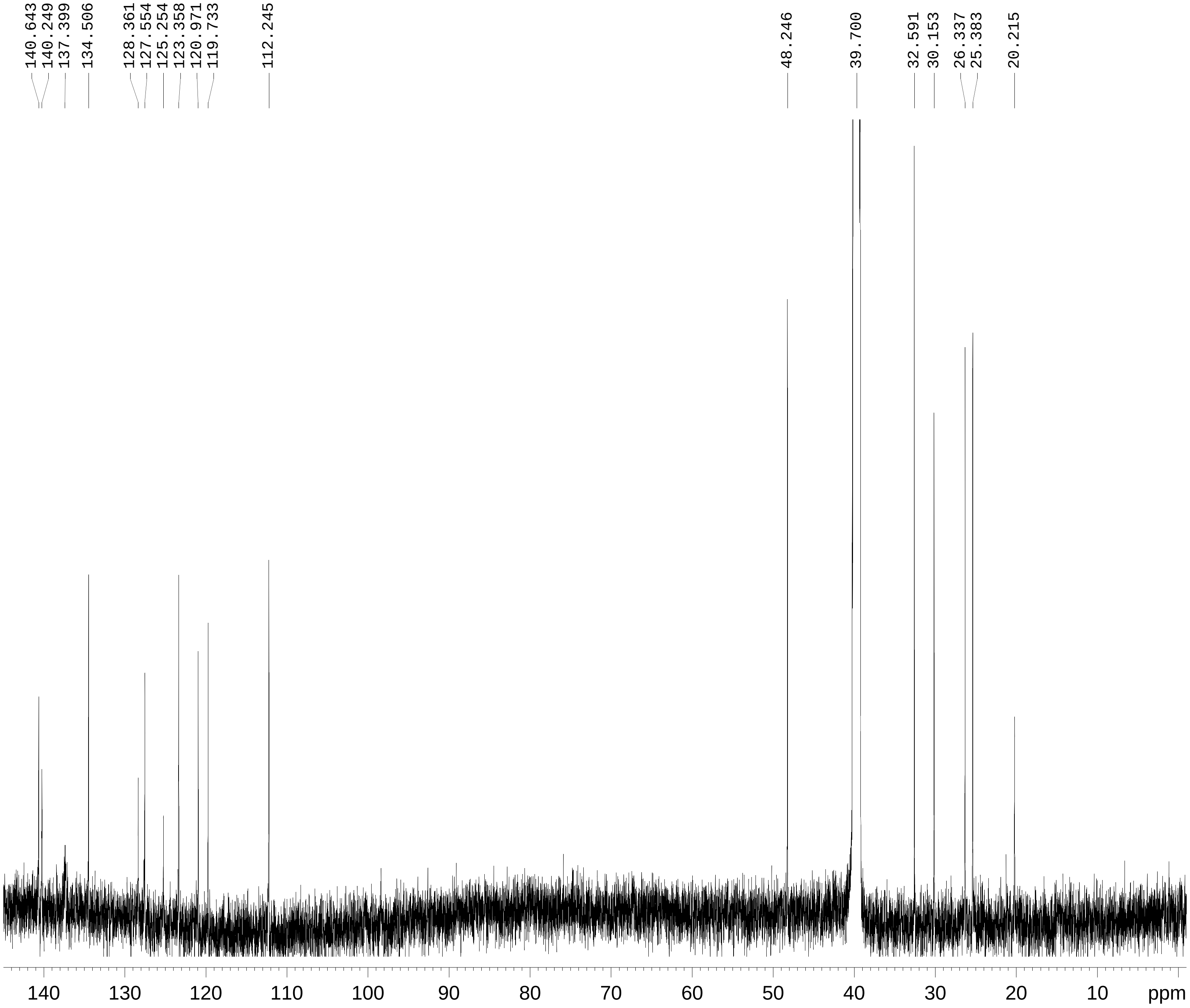

Standard 13C
143819
BAB0352_1
Batizi Benedek
2024.11.08. (KP)

Current Data Parameters

NAME	143819
EXPNO	12
PROCNO	1

F2 - Acquisition Parameters

Date_	20241109
Time	3.24 h
INSTRUM	spect
PROBHD	Z145856_0002 (
PULPROG	zgpg30
TD	65536
SOLVENT	DMSO
NS	1024
DS	4
SWH	36231.883 Hz
FIDRES	1.105709 Hz
AQ	0.9043968 sec
RG	196.07
DW	13.800 usec
DE	18.00 usec
TE	295.0 K
D1	1.00000000 sec
D11	0.03000000 sec
TD0	1
SFO1	150.8852070 MHz
NUC1	13C
P1	9.90 usec
PLW1	71.00000000 W
SFO2	600.0024000 MHz
NUC2	1H
CPDPRG[2	waltz16
PCPD2	80.00 usec
PLW2	32.90000153 W
PLW12	0.70370001 W
PLW13	0.35339001 W

F2 - Processing parameters

SI	131072
SF	150.8701599 MHz
WDW	EM
SSB	0
LB	1.00 Hz
GB	0
PC	1.40

2 · 2 HCl

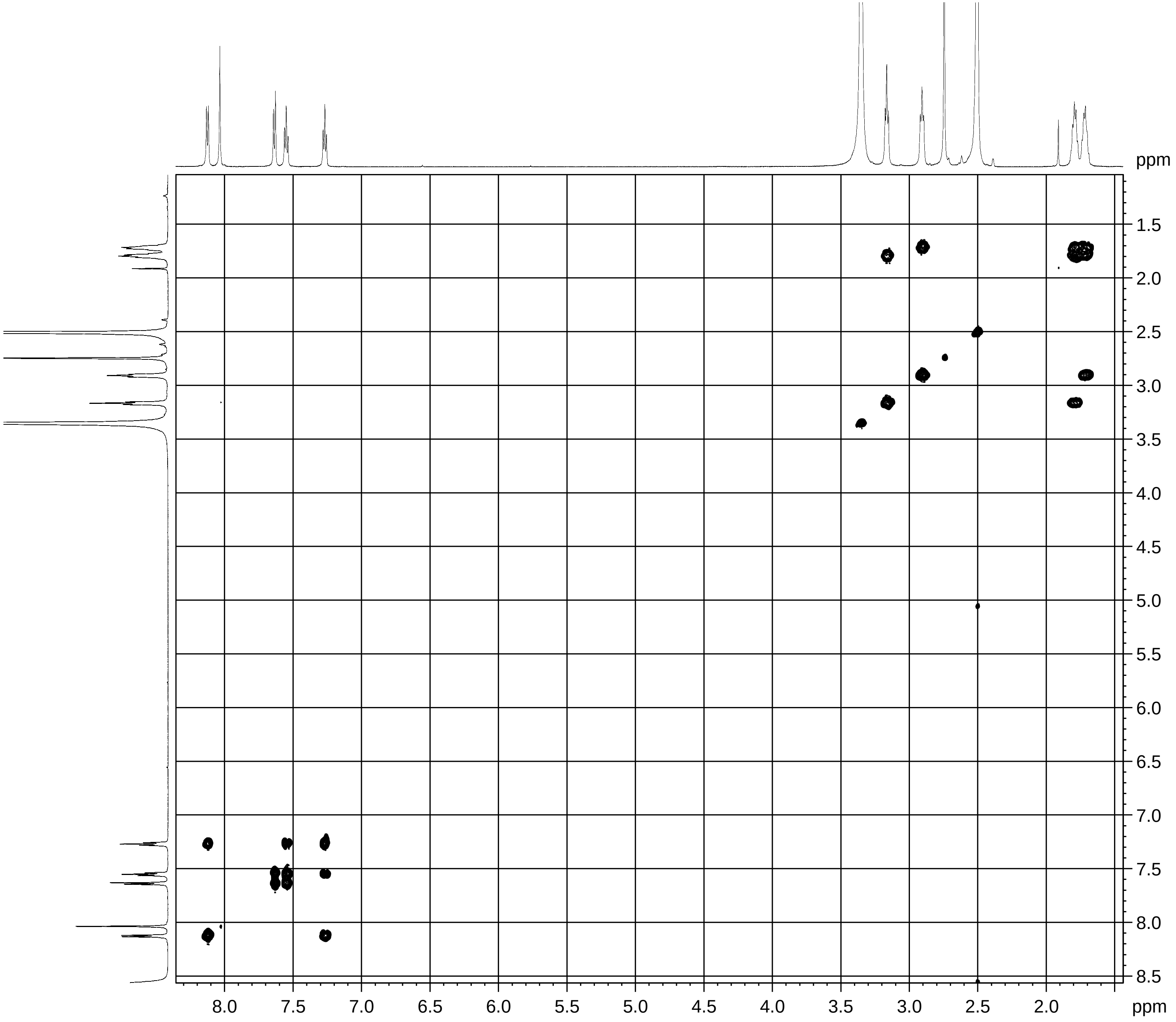

COSY
143819
BAB0352_1
Batizi Benedek
2024.11.08. (KP)

```
Current Data Parameters
NAME              143819
EXPNO                 13
PROCNO                 1

F2 - Acquisition Parameters
Date_           20241109
Time                3.26 h
INSTRUM            spect
PROBHD   Z145856_0002 (
PULPROG       cosygpmfqf
TD                  2048
SOLVENT             DMSO
NS                     2
DS                    16
SWH             7812.500 Hz
FIDRES          7.629395 Hz
AQ             0.1310720 sec
RG                196.07
DW                64.000 usec
DE                 25.00 usec
TE                 295.0 K
D0            0.00000300 sec
D1            2.00000000 sec
D13           0.00000400 sec
D16           0.00020000 sec
IN0           0.00012800 sec
TDav                   1
SFO1         600.0036000 MHz
NUC1                  1H
P1                 11.50 usec
PLW1         28.00000000 W
GPNAM[1]      SMSQ10.100
GPZ1               16.00 %
GPNAM[2]      SMSQ10.100
GPZ2               12.00 %
GPNAM[3]      SMSQ10.100
GPZ3               40.00 %
P16              1000.00 usec

F1 - Acquisition parameters
TD                   256
SFO1         600.0036 MHz
FIDRES      61.035156 Hz
SW             13.021 ppm
FnMODE                QF

F2 - Processing parameters
SI                  1024
SF          600.0000023 MHz
WDW                 SINE
SSB      0
LB       0 Hz
GB       0
PC                  1.40

F1 - Processing parameters
SI                  1024
MC2                   QF
SF          600.0000023 MHz
WDW                 SINE
SSB      0
LB       0 Hz
GB       0
```

2 · 2 HCl

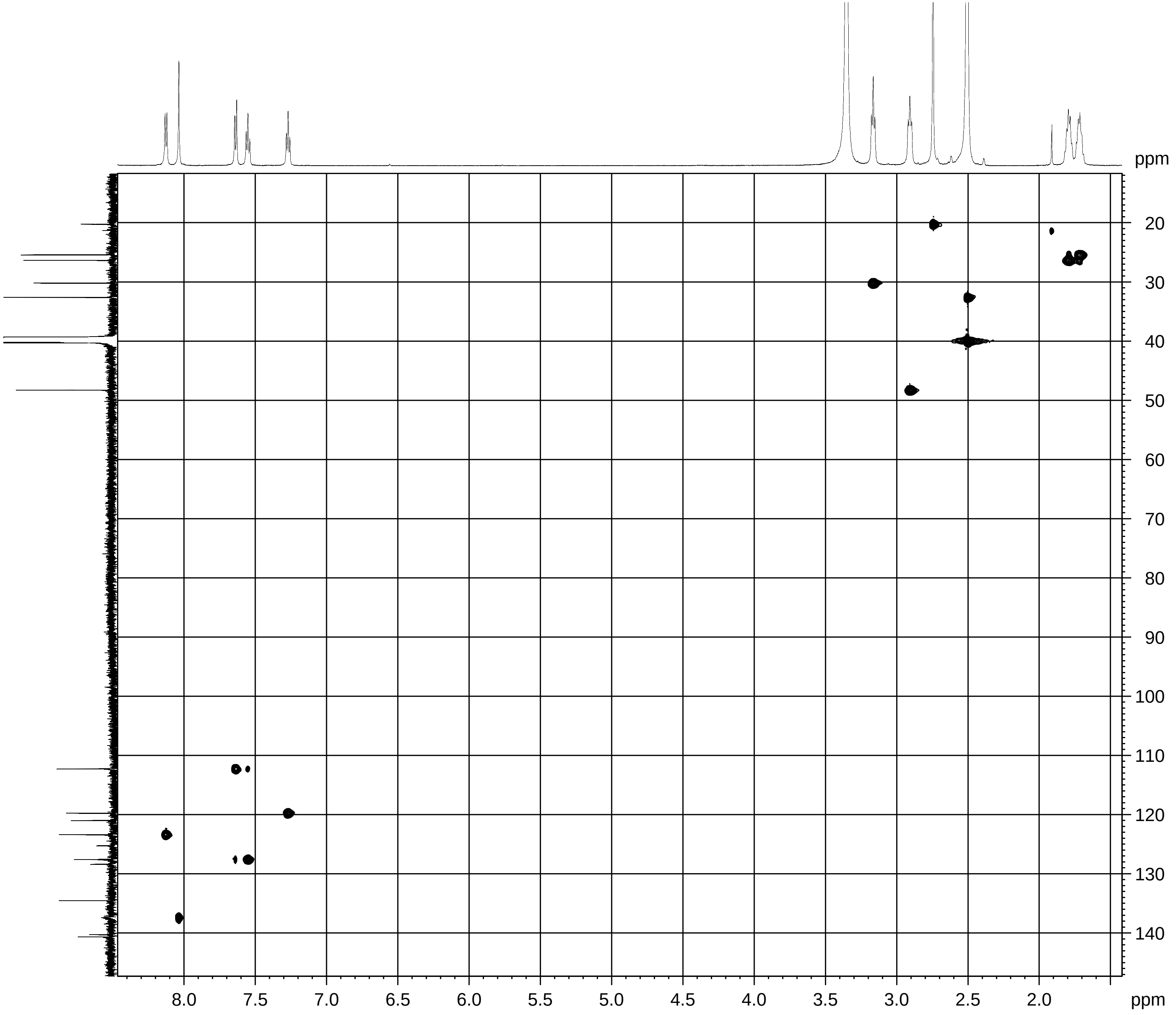

HSQC (140Hz)
143819
BAB0352_1
Batizi Benedek
2024.11.08. (KP)

```
Current Data Parameters
NAME             143819
EXPNO                14
PROCNO                1

F2 - Acquisition Parameters
Date_          20241109
Time               3.46 h
INSTRUM           spect
PROBHD   Z145856_0002 (
PULPROG  hsqcetgpsisp2.2
TD                 2048
SOLVENT            DMSO
NS                    4
DS                   32
SWH            7812.500 Hz
FIDRES         7.629395 Hz
AQ            0.1310720 sec
RG               196.07
DW               64.000 usec
DE                25.00 usec
TE                295.0 K
CNST2       140.0000000
CNST17       -0.5000000
D0           0.00000300 sec
D1           1.50000000 sec
D4           0.00178571 sec
D11          0.03000000 sec
D16          0.00020000 sec
D24          0.00089000 sec
IN0          0.00001510 sec
TDav                  1
SFO1        600.0036000 MHz
NUC1                 1H
P1                11.50 usec
P2                23.00 usec
P28      0 usec
PLW1        28.00000000 W
SFO2        150.8867157 MHz
NUC2                13C
CPDPRG[2 bi_p5m4sp_4sp.2
P3                 9.90 usec
P14              500.00 usec
P24             2000.00 usec
P63             1500.00 usec
PLW0     0 W
PLW2        71.00000000 W
PLW12        2.30040002 W
SPNAM[3] Crp60,0.5,20.1
SPOAL3            0.500
SPOFFS3  0 Hz
SPW3        10.63199997 W
SPNAM[7]    Crp60comp.4
SPOAL7            0.500
SPOFFS7  0 Hz
SPW7        10.63199997 W
SPNAM[14 Crp42,1.5,20.2
SPOAL14           0.500
SPOFFS14 0 Hz
SPW14        5.95400000 W
SPNAM[31 Crp42,1.5,20.2
SPOAL31           0.500
SPOFFS31 0 Hz
SPW31        1.48850000 W
GPNAM[1]     SMSQ10.100
GPZ1              80.00 %
GPNAM[2]     SMSQ10.100
GPZ2              20.10 %
GPNAM[3]     SMSQ10.100
GPZ3              11.00 %
GPNAM[4]     SMSQ10.100
GPZ4              -5.00 %
P16             1000.00 usec
P19              600.00 usec

F1 - Acquisition parameters
TD                  256
SFO1           150.8867 MHz
FIDRES       258.692047 Hz
SW              219.453 ppm
FnMODE    Echo-Antiecho

F2 - Processing parameters
SI                 1024
SF          600.0000023 MHz
WDW               QSINE
```

BRUKER

2 · 2 HCl

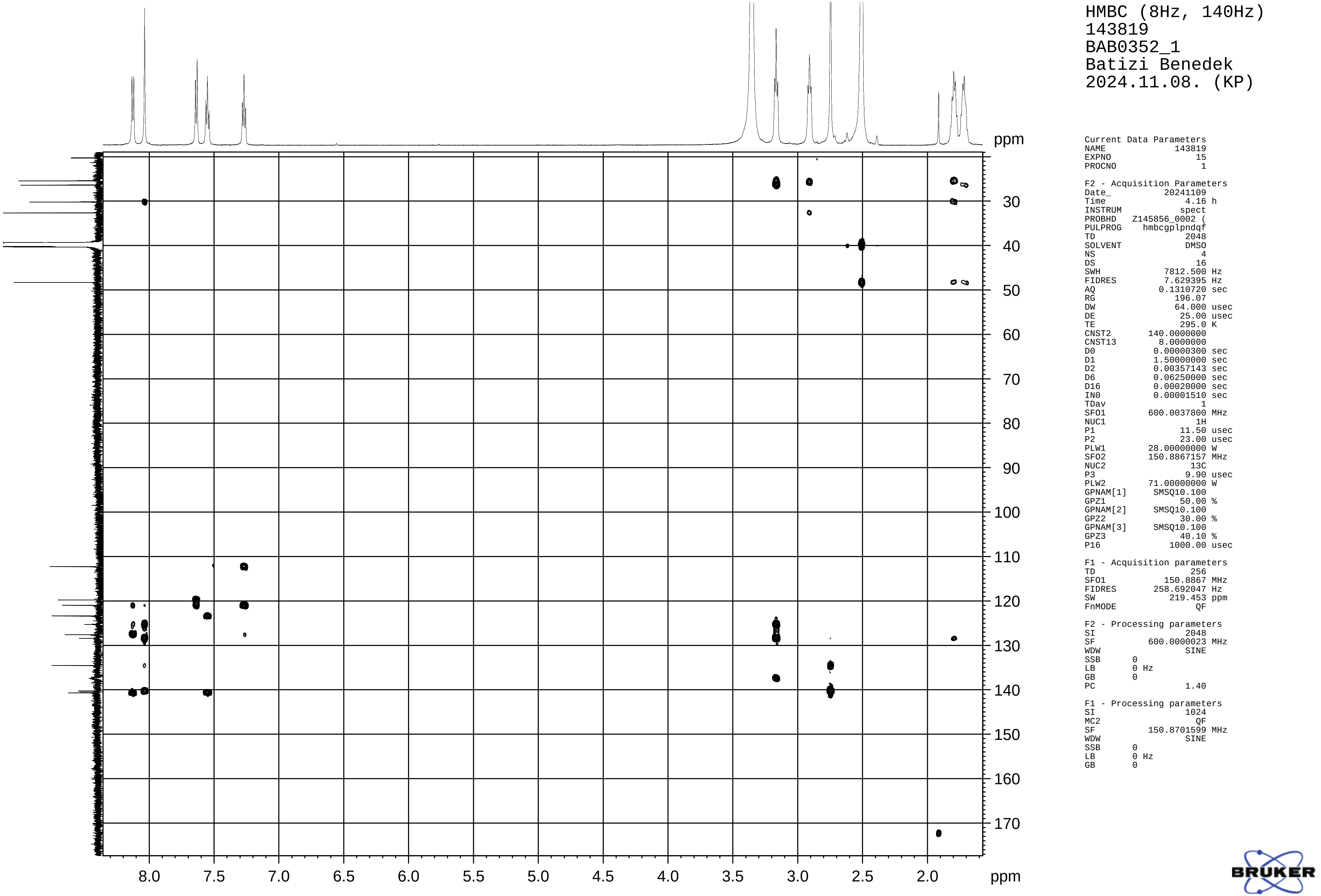

HMBC (8Hz, 140Hz)
143819
BAB0352_1
Batizi Benedek
2024.11.08. (KP)
ppm
30
40
50
60
70
80
90
100
110
120
130
140
150
160
170
8.0
7.5
7.0
6.5
6.0
5.5
5.0
4.5
4.0
3.5
3.0
2.5
2.0
ppm
Current Data Parameters
NAME 143819
EXPNO 15
PROCNO 1
F2 - Acquisition Parameters
Date_ 20241109
Time 4.16 h
INSTRUM spect
PROBHD Z145856_0002 (
PULPROG hmbcgplpndqf
TD 2048
SOLVENT DMSO
NS 4
DS 16
SWH 7812.500 Hz
FIDRES 7.629395 Hz
AQ 0.1310720 sec
RG 196.07
DW 64.000 usec
DE 25.00 usec
TE 295.0 K
CNST2 140.0000000
CNST13 8.0000000
D0 0.00000300 sec
D1 1.50000000 sec
D2 0.00357143 sec
D6 0.06250000 sec
D16 0.00020000 sec
IN0 0.00001510 sec
TDav 1
SFO1 600.0037800 MHz
NUC1 1H
P1 11.50 usec
P2 23.00 usec
PLW1 28.00000000 W
SFO2 150.8867157 MHz
NUC2 13C
P3 9.90 usec
PLW2 71.00000000 W
GPNAM[1] SMSQ10.100
GPZ1 50.00 %
GPNAM[2] SMSQ10.100
GPZ2 30.00 %
GPNAM[3] SMSQ10.100
GPZ3 40.10 %
P16 1000.00 usec
F1 - Acquisition parameters
TD 256
SFO1 150.8867 MHz
FIDRES 258.692047 Hz
SW 219.453 ppm
FnMODE QF
F2 - Processing parameters
SI 2048
SF 600.0000023 MHz
WDW SINE
SSB 0
LB 0 Hz
GB 0
PC 1.40
F1 - Processing parameters
SI 1024
MC2 QF
SF 150.8701599 MHz
WDW SINE
SSB 0
LB 0 Hz
GB 0

5

Batizi Benedek	MM
KBr	2023.01.26.

BRUKER Alpha
Resolution: 2 cm-1
Number of Scans: 16

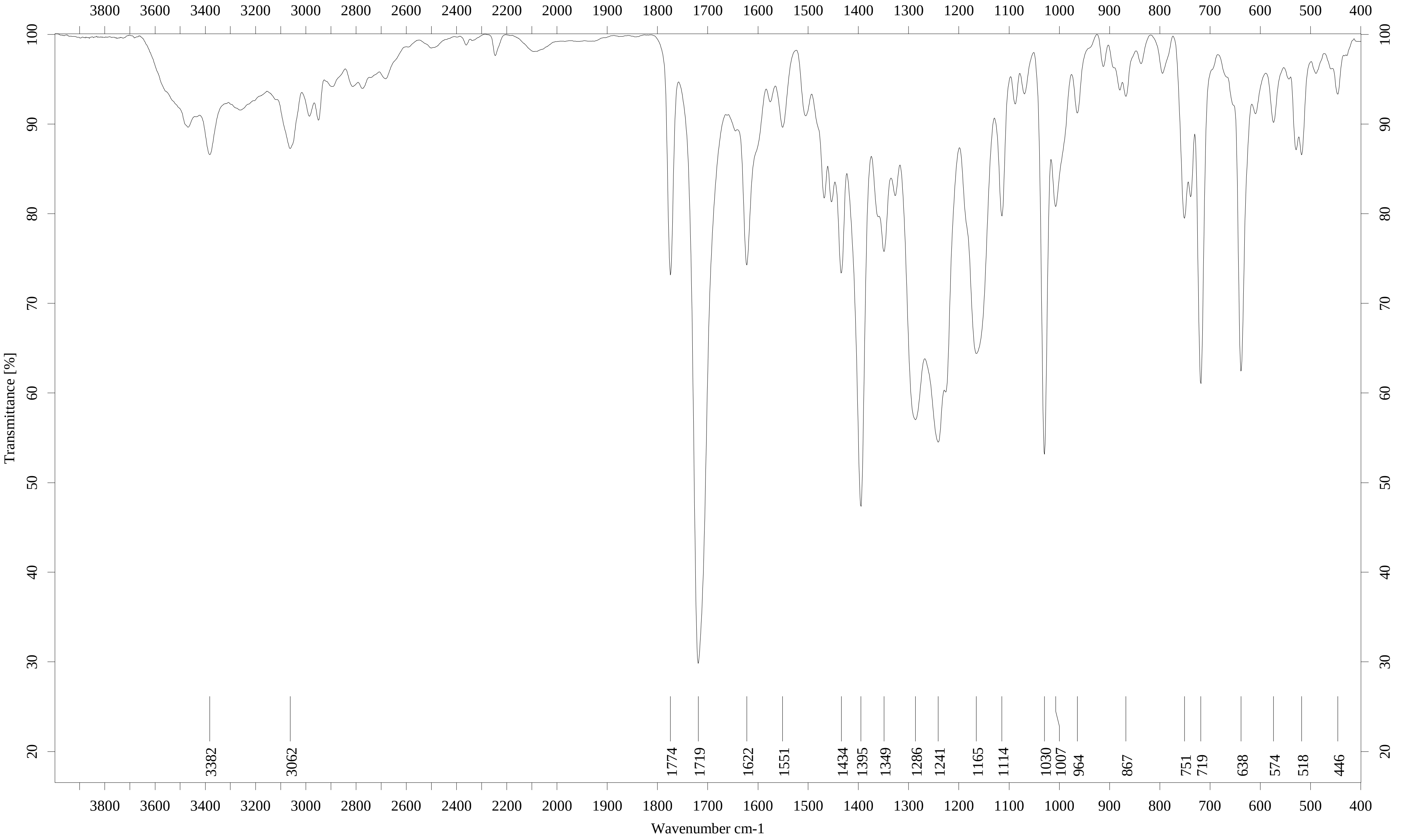

5

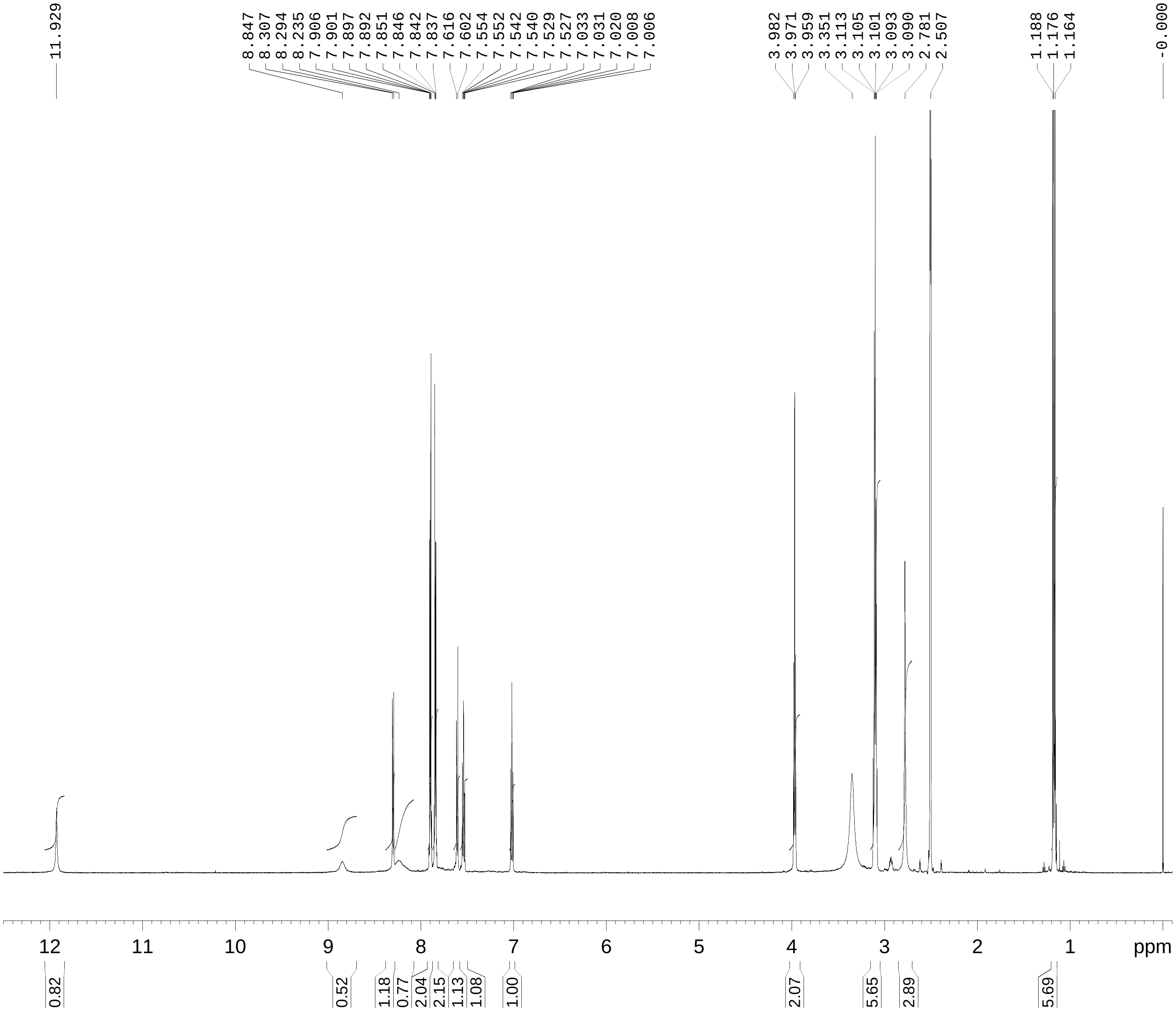

Standard 1H
140379
BAB0025_1
Batizi Benedek
2023.01.25. (KP)

Current Data Parameters

NAME	140379
EXPNO	11
PROCNO	1

F2 - Acquisition Parameters

Date_	20230125
Time	21.23 h
INSTRUM	spect
PROBHD	Z145856_0002 (
PULPROG	zg30
TD	65536
SOLVENT	DMSO
NS	16
DS	2
SWH	12019.230 Hz
FIDRES	0.366798 Hz
AQ	2.7262976 sec
RG	196.07
DW	41.600 usec
DE	25.00 usec
TE	295.0 K
D1	1.00000000 sec
TD0	1
SFO1	600.0537053 MHz
NUC1	1H
P1	11.50 usec
PLW1	28.00000000 W

F2 - Processing parameters

SI	65536
SF	600.0500008 MHz
WDW	no
SSB	0
LB	0 Hz
GB	0
PC	1.00

5

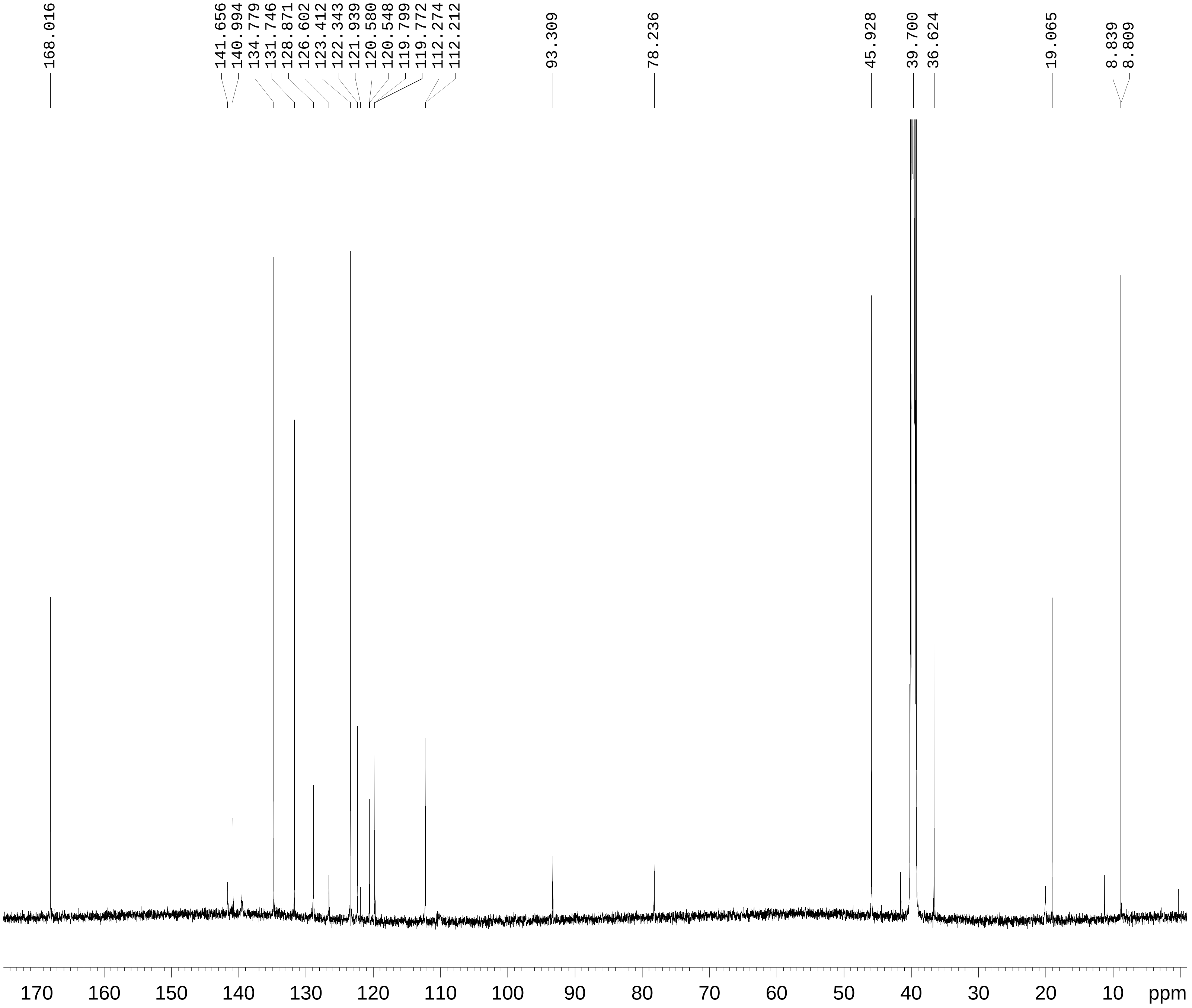

Standard 13C
140379
BAB0025_1
Batizi Benedek
2023.01.25. (KP)

Current Data Parameters	
NAME	140379
EXPNO	12
PROCNO	1

F2 - Acquisition Parameters	
Date_	20230125
Time	22.32 h
INSTRUM	spect
PROBHD	Z145856_0002 (
PULPROG	zgpg30
TD	65536
SOLVENT	DMSO
NS	2048
DS	4
SWH	36231.883 Hz
FIDRES	1.105709 Hz
AQ	0.9043968 sec
RG	196.07
DW	13.800 usec
DE	18.00 usec
TE	295.0 K
D1	1.00000000 sec
D11	0.03000000 sec
TD0	1
SFO1	150.8977808 MHz
NUC1	13C
P1	10.00 usec
PLW1	70.48699951 W
SFO2	600.0524002 MHz
NUC2	1H
CPDPRG[2	waltz16
PCPD2	80.00 usec
PLW2	29.46299934 W
PLW12	0.66293001 W
PLW13	0.33292001 W

F2 - Processing parameters	
SI	32768
SF	150.8827330 MHz
WDW	EM
SSB	0
LB	1.00 Hz
GB	0
PC	1.40

6

Batizi Benedek	KP
KBr	2023.10.31.

BRUKER Alpha
Resolution: 2 cm-1
Number of Scans: 16

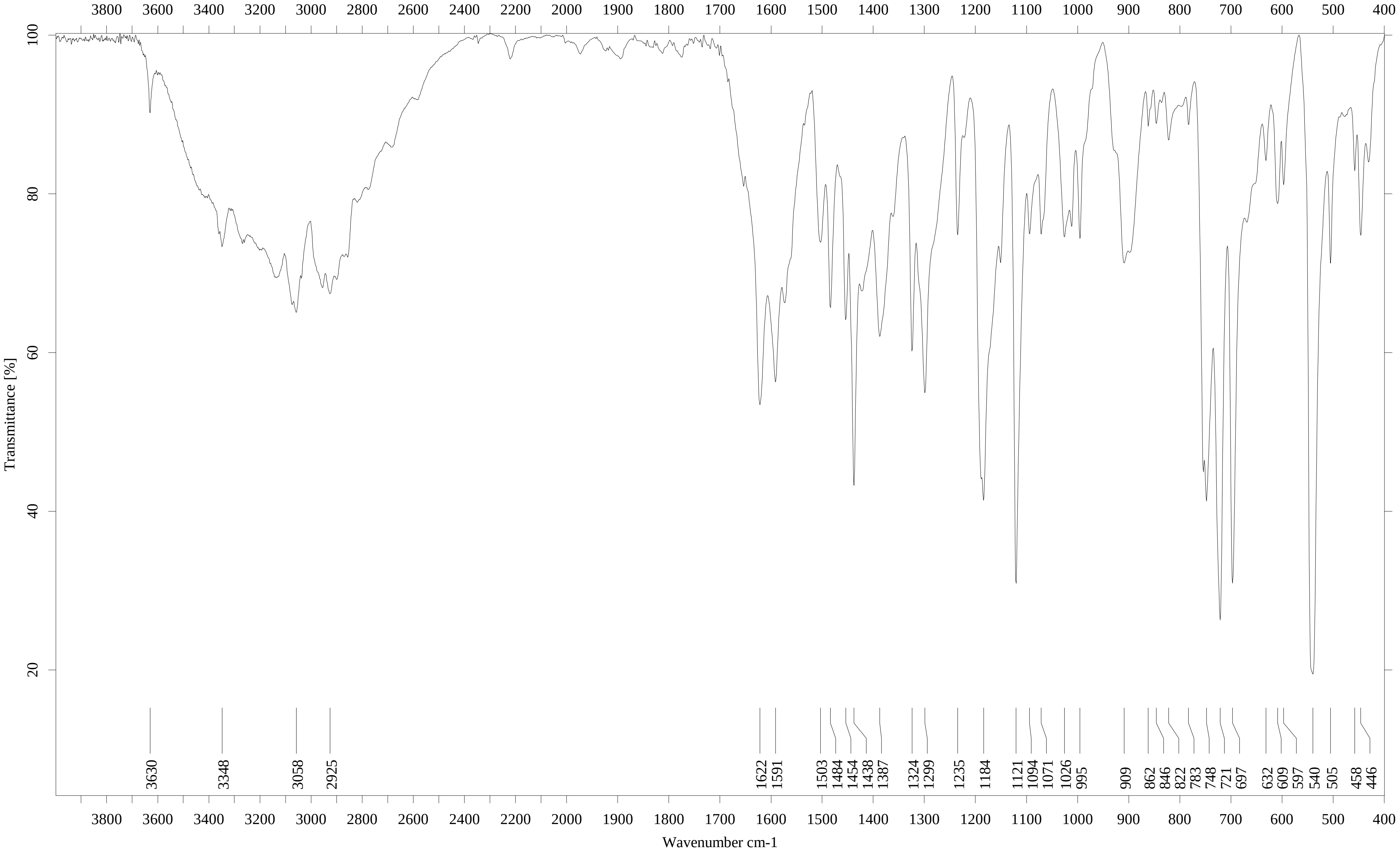

6

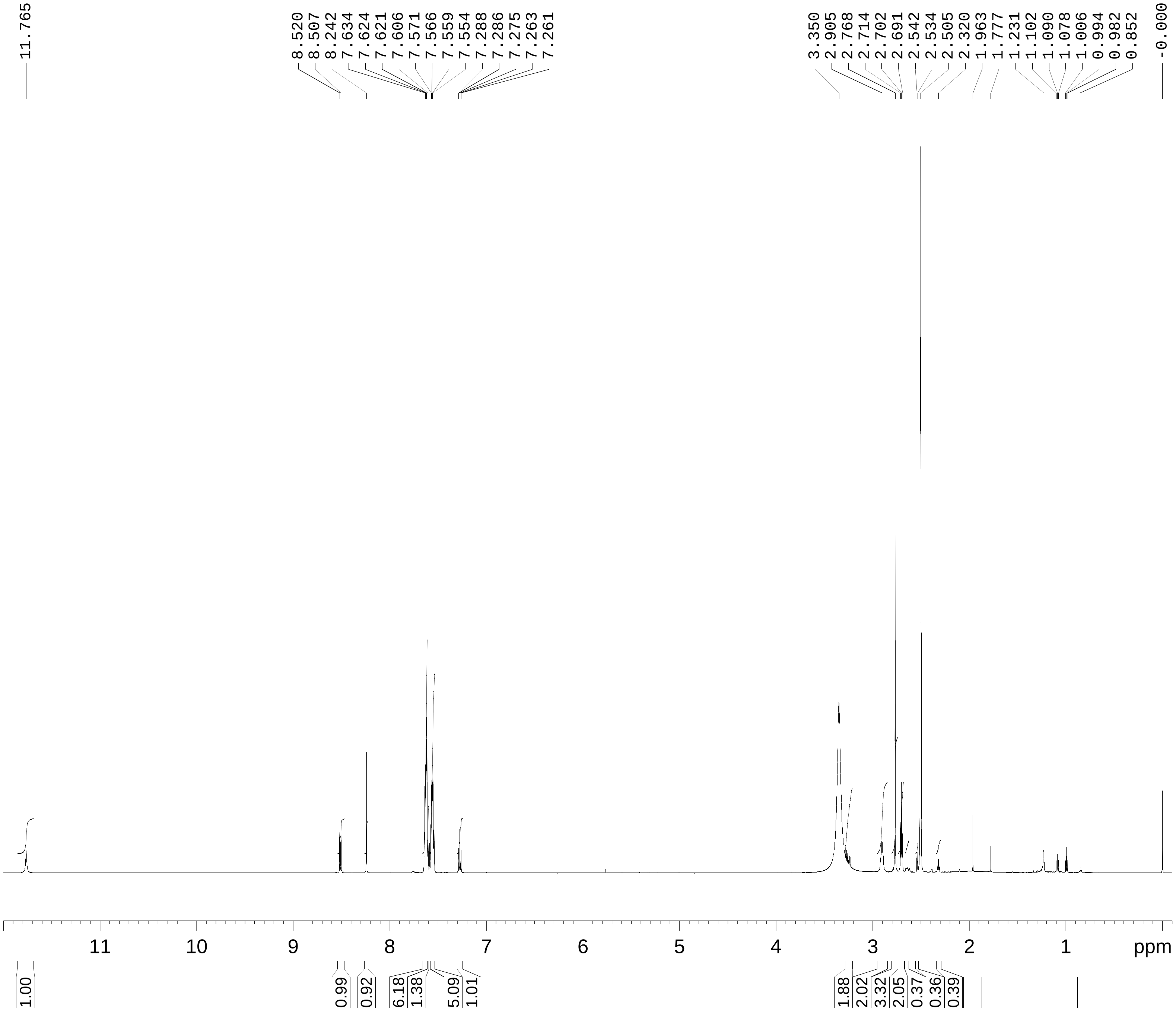

Standard 1H
142090
BAB0103_1
Batizi Benedek
2023.10.31. (DA)

Current Data Parameters
NAME 142090
EXPNO 11
PROCNO 1

F2 - Acquisition Parameters
Date_ 20231031
Time 18.18 h
INSTRUM spect
PROBHD Z145856_0002 (
PULPROG zg30
TD 65536
SOLVENT DMSO
NS 16
DS 2
SWH 12019.230 Hz
FIDRES 0.366798 Hz
AQ 2.7262976 sec
RG 196.07
DW 41.600 usec
DE 25.00 usec
TE 295.0 K
D1 1.00000000 sec
TD0 1
SFO1 600.0037050 MHz
NUC1 1H
P1 11.50 usec
PLW1 28.00000000 W

F2 - Processing parameters
SI 65536
SF 600.0000029 MHz
WDW EM
SSB 0
LB 0.30 Hz
GB 0
PC 1.00

6

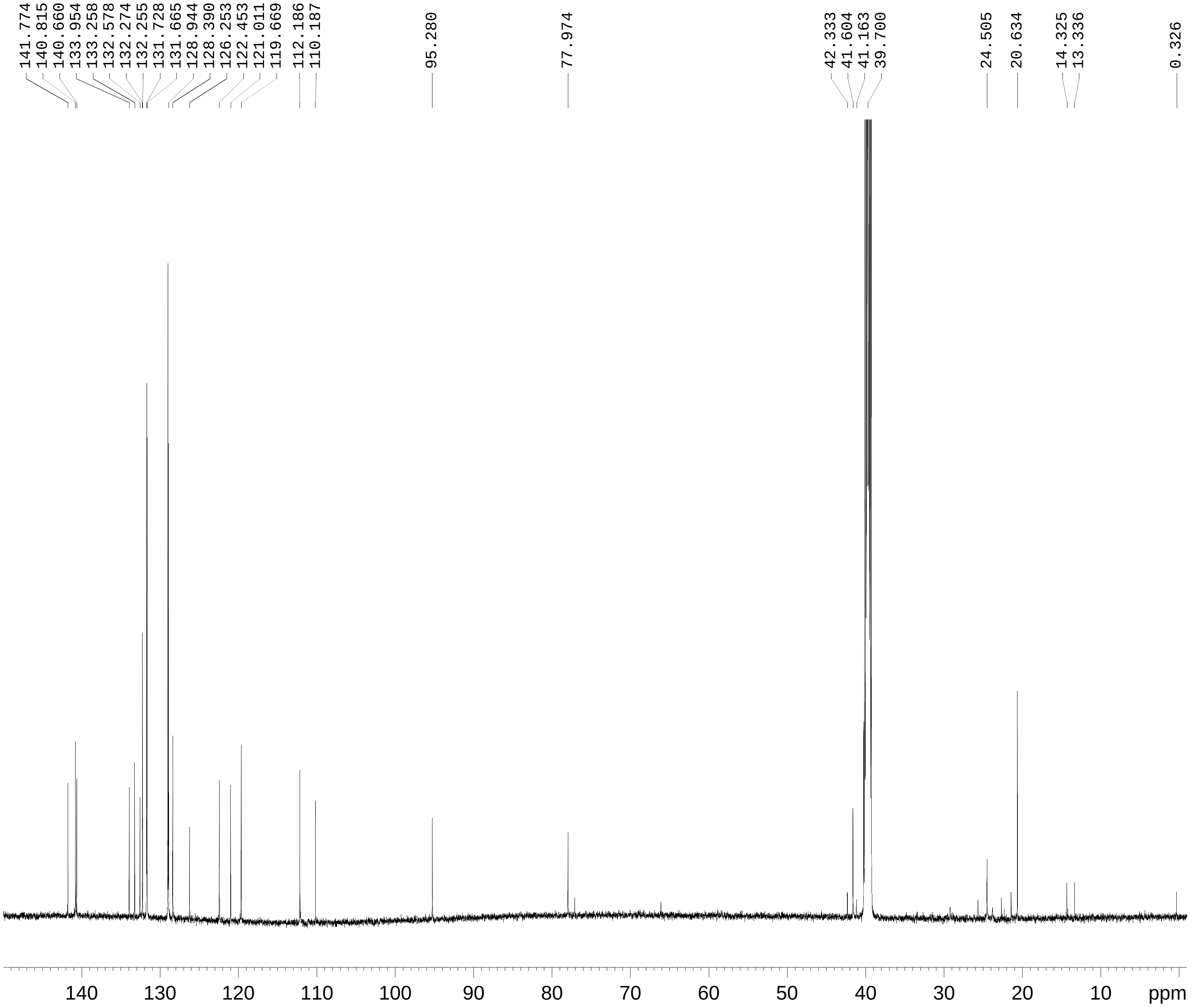

Standard 13C
142090
BAB0103_1
Batizi Benedek
2023.10.31. (DA)

Current Data Parameters

NAME	142090	
EXPNO	12	
PROCNO	1	

F2 - Acquisition Parameters

Date_	20231031	
Time	20.33	h
INSTRUM	spect	
PROBHD	Z145856_0002 (	
PULPROG	zgpg30	
TD	65536	
SOLVENT	DMSO	
NS	4096	
DS	4	
SWH	36231.883	Hz
FIDRES	1.105709	Hz
AQ	0.9043968	sec
RG	196.07	
DW	13.800	usec
DE	18.00	usec
TE	295.0	K
D1	1.00000000	sec
D11	0.03000000	sec
TD0	1	
SFO1	150.8852070	MHz
NUC1	13C	
P1	9.90	usec
PLW1	80.09999847	W
SFO2	600.0024000	MHz
NUC2	1H	
CPDPRG[2	waltz16	
PCPD2	80.00	usec
PLW2	35.00000000	W
PLW12	0.74861997	W
PLW13	0.37595001	W

F2 - Processing parameters

SI	32768	
SF	150.8701593	MHz
WDW	EM	
SSB	0	
LB	1.00	Hz
GB	0	
PC	1.40	

6

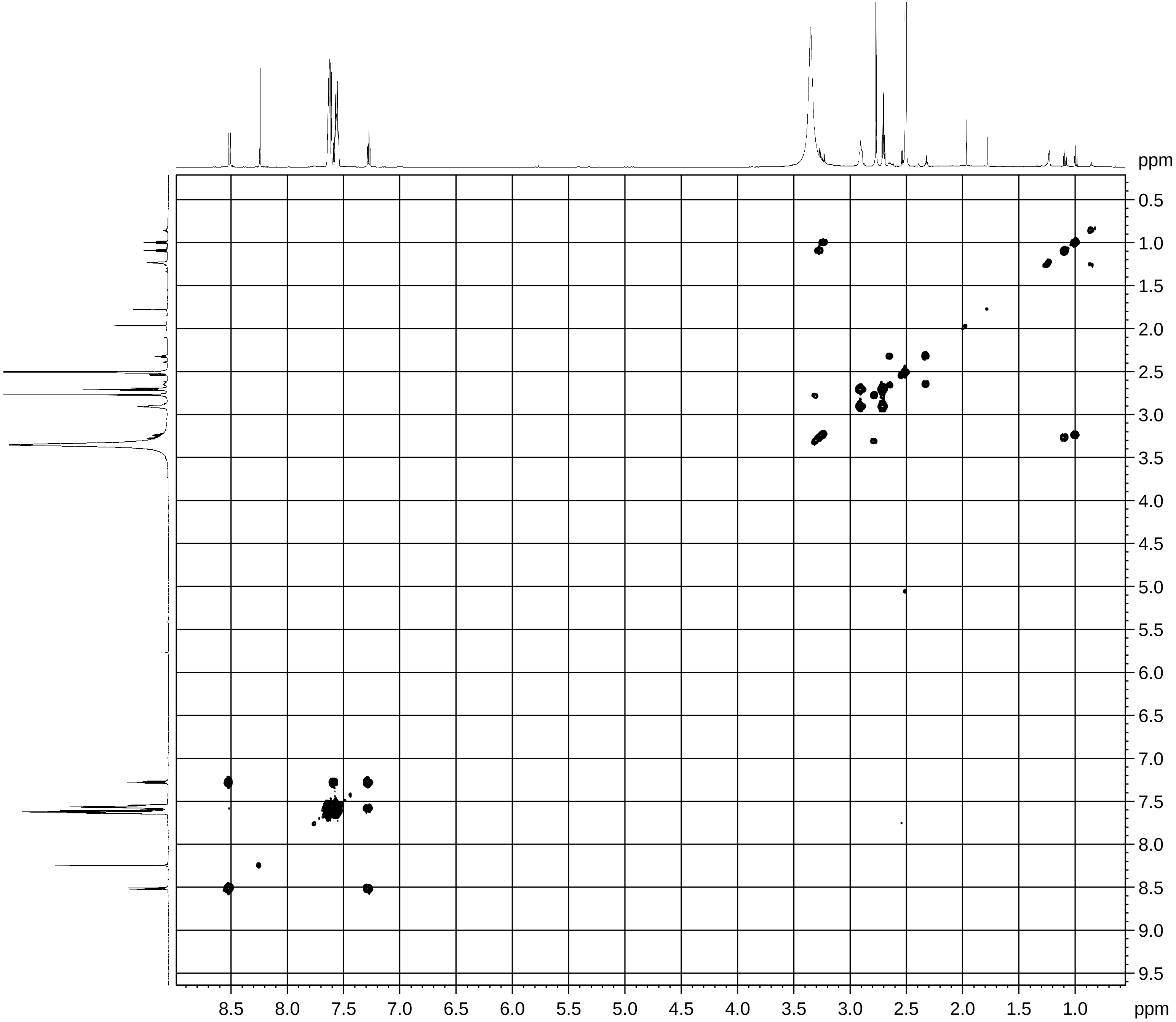

COSY
142090
BAB0103_1
Batizi Benedek
2023.10.31. (DA)

```
Current Data Parameters
NAME               142090
EXPNO                  13
PROCNO                  1

F2 - Acquisition Parameters
Date_            20231031
Time                20.35 h
INSTRUM             spect
PROBHD   Z145856_0002 (
PULPROG       cosygpmfqf
TD                   2048
SOLVENT              DMSO
NS                      2
DS                     16
SWH              7812.500 Hz
FIDRES           7.629395 Hz
AQ              0.1310720 sec
RG                 196.07
DW                 64.000 usec
DE                  25.00 usec
TE                  295.0 K
D0             0.00000300 sec
D1             2.00000000 sec
D13            0.00000400 sec
D16            0.00020000 sec
IN0            0.00012800 sec
TDav                    1
SFO1          600.0036000 MHz
NUC1                   1H
P1                  11.50 usec
PLW1          28.00000000 W
GPNAM[1]      SMSQ10.100
GPZ1                16.00 %
GPNAM[2]      SMSQ10.100
GPZ2                12.00 %
GPNAM[3]      SMSQ10.100
GPZ3                40.00 %
P16               1000.00 usec

F1 - Acquisition parameters
TD                    256
SFO1             600.0036 MHz
FIDRES          61.035156 Hz
SW                 13.021 ppm
FnMODE                 QF

F2 - Processing parameters
SI                   1024
SF            600.0000029 MHz
WDW                  SINE
SSB      0
LB       0 Hz
GB       0
PC                   1.40

F1 - Processing parameters
SI                   1024
MC2                    QF
SF            600.0000029 MHz
WDW                  SINE
SSB      0
LB       0 Hz
GB       0
```

6

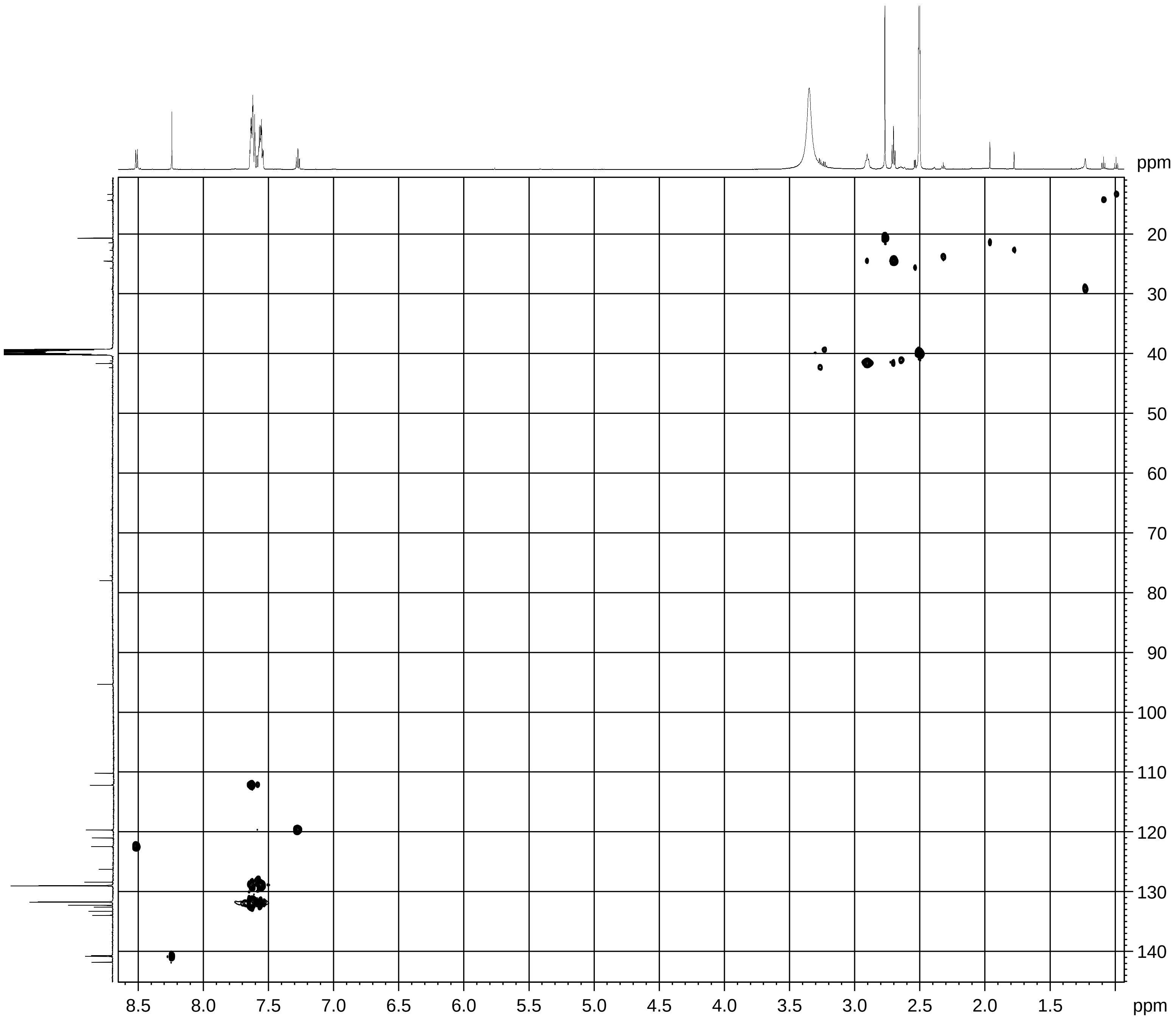

HSQC (140 Hz)
142090
BAB0103_1
Batizi Benedek
2023.10.31. (DA)

Current Data Parameters
NAME 142090
EXPNO 14
PROCNO 1

F2 - Acquisition Parameters
Date_ 20231031
Time 20.56 h
INSTRUM spect
PROBHD Z145856_0002 (
PULPROG hsqcetgpsisp2.2
TD 2048
SOLVENT DMSO
NS 4
DS 32
SWH 7812.500 Hz
FIDRES 7.629395 Hz
AQ 0.1310720 sec
RG 196.07
DW 64.000 usec
DE 25.00 usec
TE 295.0 K
CNST2 140.0000000
CNST17 -0.5000000
D0 0.00000300 sec
D1 1.50000000 sec
D4 0.00178571 sec
D11 0.03000000 sec
D16 0.00020000 sec
D24 0.00089000 sec
IN0 0.00001510 sec
TDav 1
SFO1 600.0036000 MHz
NUC1 1H
P1 11.50 usec
P2 23.00 usec
P28 0 usec
PLW1 28.00000000 W
SFO2 150.8867157 MHz
NUC2 13C
CPDPRG[2 bi_p5m4sp_4sp.2
P3 9.90 usec
P14 500.00 usec
P24 2000.00 usec
P63 1500.00 usec
PLW0 0 W
PLW2 80.09999847 W
PLW12 2.59520006 W
SPNAM[3] Crp60,0.5,20.1
SPOAL3 0.500
SPOFFS3 0 Hz
SPW3 11.99499989 W
SPNAM[7] Crp60comp.4
SPOAL7 0.500
SPOFFS7 0 Hz
SPW7 11.99499989 W
SPNAM[14 Crp42,1.5,20.2
SPOAL14 0.500
SPOFFS14 0 Hz
SPW14 6.71710014 W
SPNAM[31 Crp42,1.5,20.2
SPOAL31 0.500
SPOFFS31 0 Hz
SPW31 1.67929995 W
GPNAM[1] SMSQ10.100
GPZ1 80.00 %
GPNAM[2] SMSQ10.100
GPZ2 20.10 %
GPNAM[3] SMSQ10.100
GPZ3 11.00 %
GPNAM[4] SMSQ10.100
GPZ4 -5.00 %
P16 1000.00 usec
P19 600.00 usec

F1 - Acquisition parameters
TD 256
SFO1 150.8867 MHz
FIDRES 258.692047 Hz
SW 219.453 ppm
FnMODE Echo-Antiecho

F2 - Processing parameters
SI 1024
SF 600.0000029 MHz
WDW QSINE

6

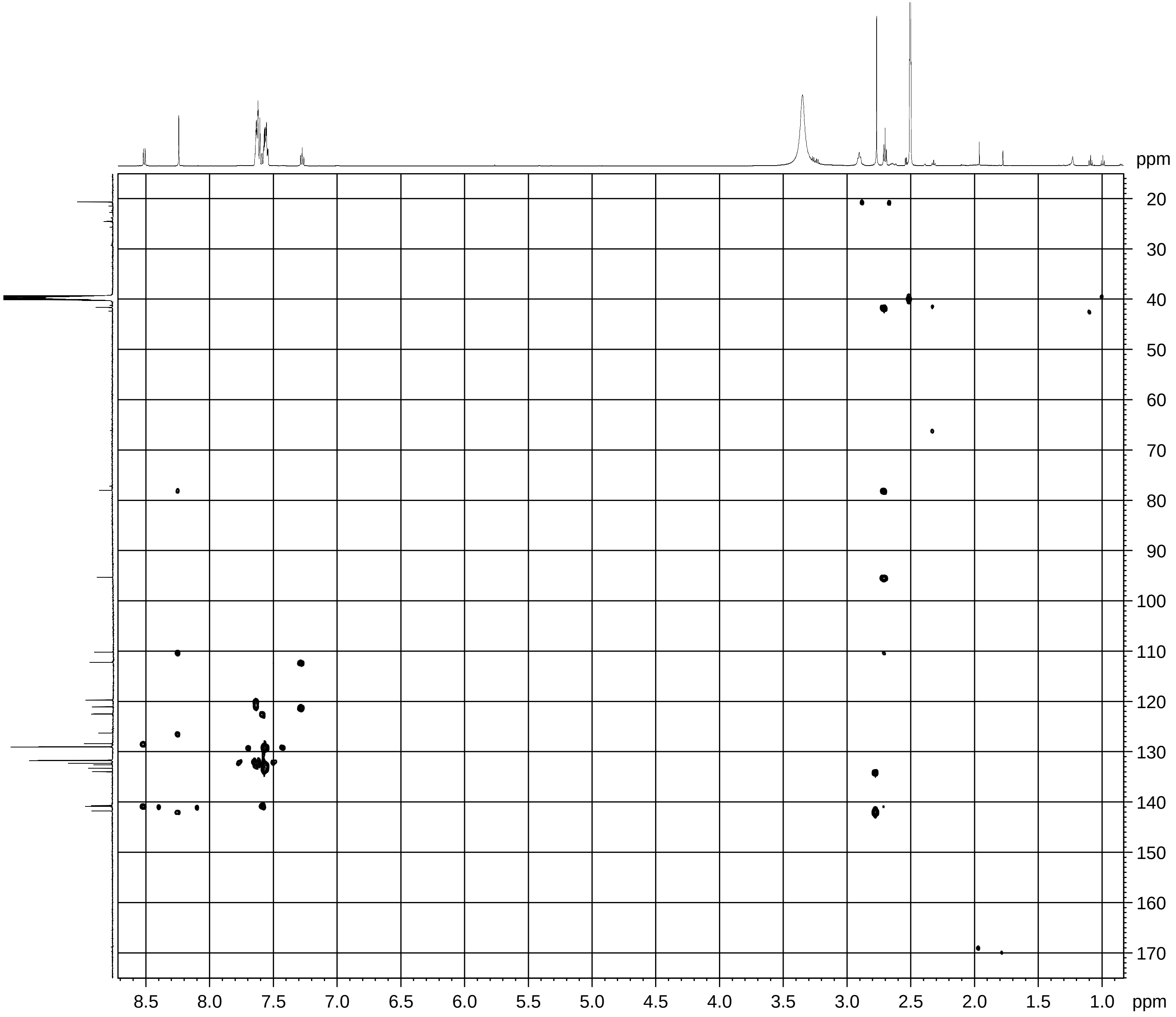
HMBC (8 Hz, 140 Hz)
142090
BAB0103_1
Batizi Benedek
2023.10.31. (DA)
ppm
ppm
Current Data Parameters
NAME 142090
EXPNO 15
PROCNO 1
F2 - Acquisition Parameters
Date_ 20231031
Time 21.25 h
INSTRUM spect
PROBHD Z145856_0002 (
PULPROG hmbcgplpndqf
TD 2048
SOLVENT DMSO
NS 4
DS 16
SWH 7812.500 Hz
FIDRES 7.629395 Hz
AQ 0.1310720 sec
RG 196.07
DW 64.000 usec
DE 25.00 usec
TE 295.0 K
CNST2 140.0000000
CNST13 8.0000000
D0 0.00000300 sec
D1 1.50000000 sec
D2 0.00357143 sec
D6 0.06250000 sec
D16 0.00020000 sec
IN0 0.00001510 sec
TDav 1
SFO1 600.0037800 MHz
NUC1 1H
P1 11.50 usec
P2 23.00 usec
PLW1 28.00000000 W
SFO2 150.8867157 MHz
NUC2 13C
P3 9.90 usec
PLW2 80.09999847 W
GPNAM[1] SMSQ10.100
GPZ1 50.00 %
GPNAM[2] SMSQ10.100
GPZ2 30.00 %
GPNAM[3] SMSQ10.100
GPZ3 40.10 %
P16 1000.00 usec
F1 - Acquisition parameters
TD 256
SFO1 150.8867 MHz
FIDRES 258.692047 Hz
SW 219.453 ppm
FnMODE QF
F2 - Processing parameters
SI 2048
SF 600.0000000 MHz
WDW SINE
SSB 0
LB 0 Hz
GB 0
PC 1.40
F1 - Processing parameters
SI 1024
MC2 QF
SF 150.8701200 MHz
WDW SINE
SSB 0
LB 0 Hz
GB 0

24

Batizi Benedek	KP
KBr	8/2/2024

BRUKER Alpha
Resolution: 2 cm-1
Number of Scans: 16

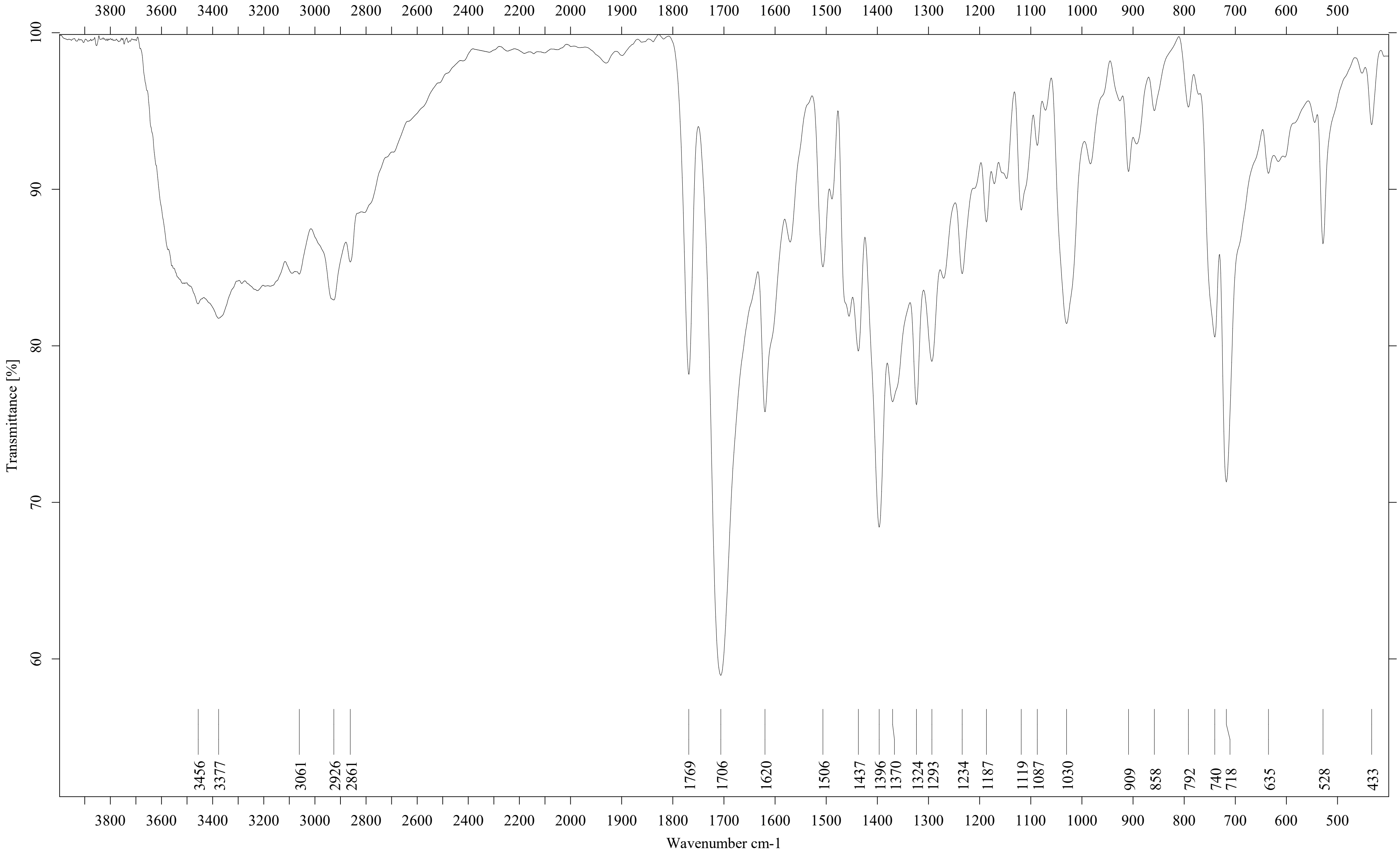

24

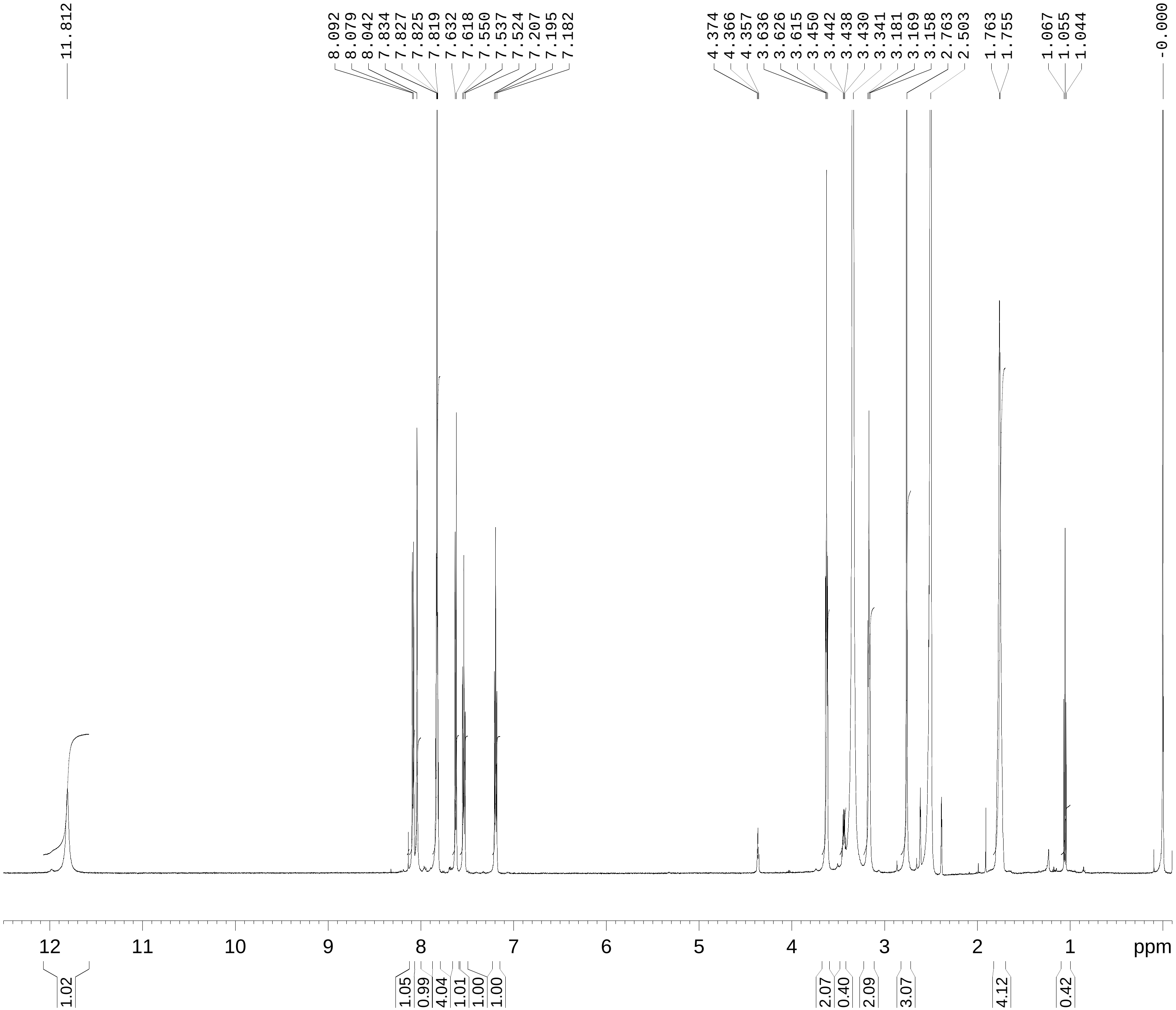

Standard 1H
143668
BAB0334_1
Batizi Benedek
2024.10.07. (KP)

Current Data Parameters
NAME 143668
EXPNO 11
PROCNO 1

F2 - Acquisition Parameters
Date_ 20241007
Time 15.44 h
INSTRUM spect
PROBHD Z145856_0002 (
PULPROG zg30
TD 65536
SOLVENT DMSO
NS 128
DS 2
SWH 12019.230 Hz
FIDRES 0.366798 Hz
AQ 2.7262976 sec
RG 196.07
DW 41.600 usec
DE 25.00 usec
TE 295.0 K
D1 1.00000000 sec
TD0 1
SFO1 600.0037050 MHz
NUC1 1H
P1 11.50 usec
PLW1 28.00000000 W

F2 - Processing parameters
SI 65536
SF 600.0000027 MHz
WDW EM
SSB 0
LB 0.30 Hz
GB 0
PC 1.00

24

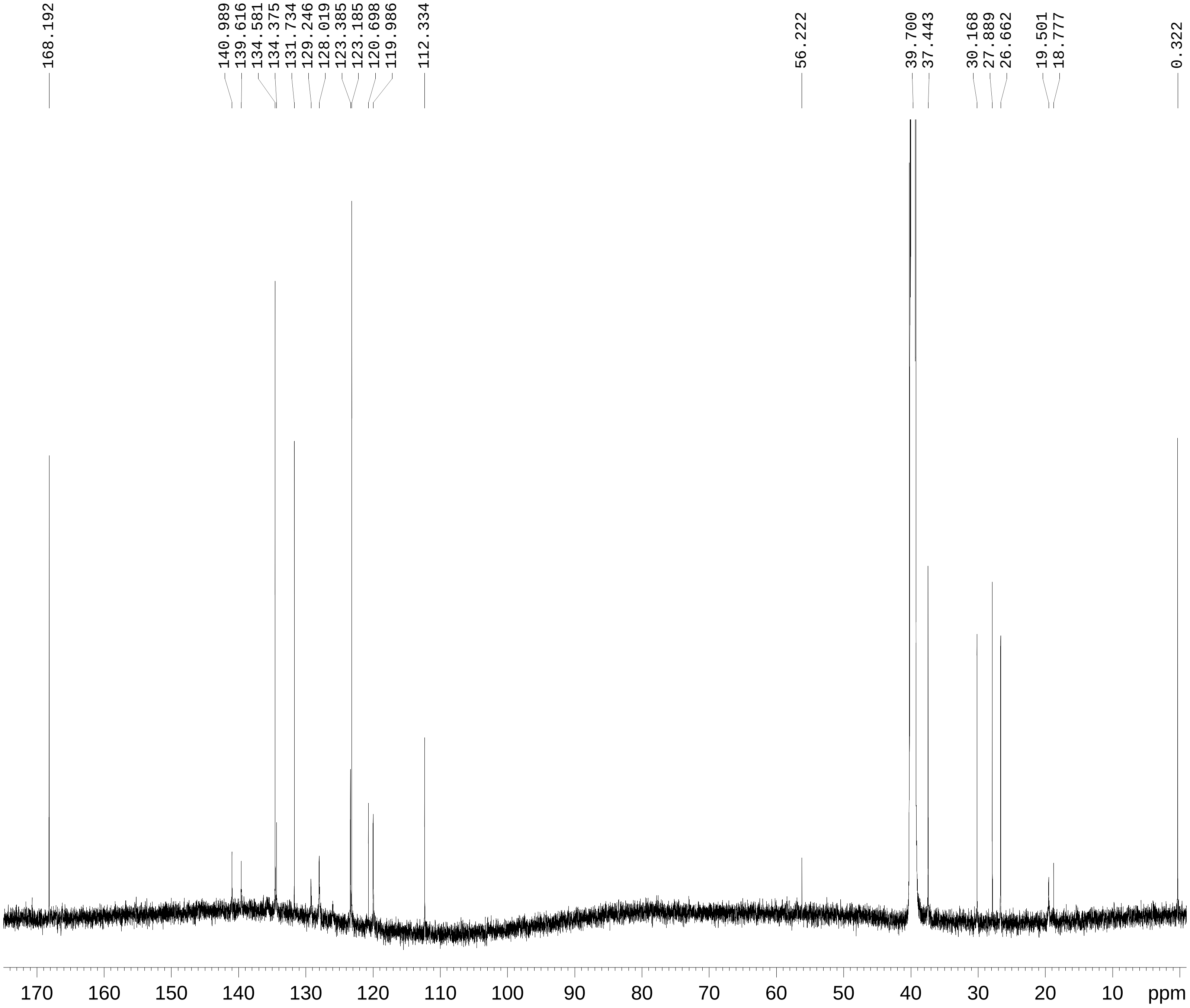

Standard 13C
143668
BAB0334_1
Batizi Benedek
2024.10.07. (KP)

Current Data Parameters
NAME 143668
EXPNO 12
PROCNO 1

F2 - Acquisition Parameters
Date_ 20241007
Time 20.14 h
INSTRUM spect
PROBHD Z145856_0002 (
PULPROG zgpg30
TD 65536
SOLVENT DMSO
NS 8192
DS 4
SWH 36231.883 Hz
FIDRES 1.105709 Hz
AQ 0.9043968 sec
RG 196.07
DW 13.800 usec
DE 18.00 usec
TE 295.0 K
D1 1.00000000 sec
D11 0.03000000 sec
TD0 1
SFO1 150.8852070 MHz
NUC1 13C
P1 9.90 usec
PLW1 71.00000000 W
SFO2 600.0024000 MHz
NUC2 1H
CPDPRG[2 waltz16
PCPD2 80.00 usec
PLW2 32.90000153 W
PLW12 0.70370001 W
PLW13 0.35339001 W

F2 - Processing parameters
SI 131072
SF 150.8701602 MHz
WDW EM
SSB 0
LB 1.00 Hz
GB 0
PC 1.40

24

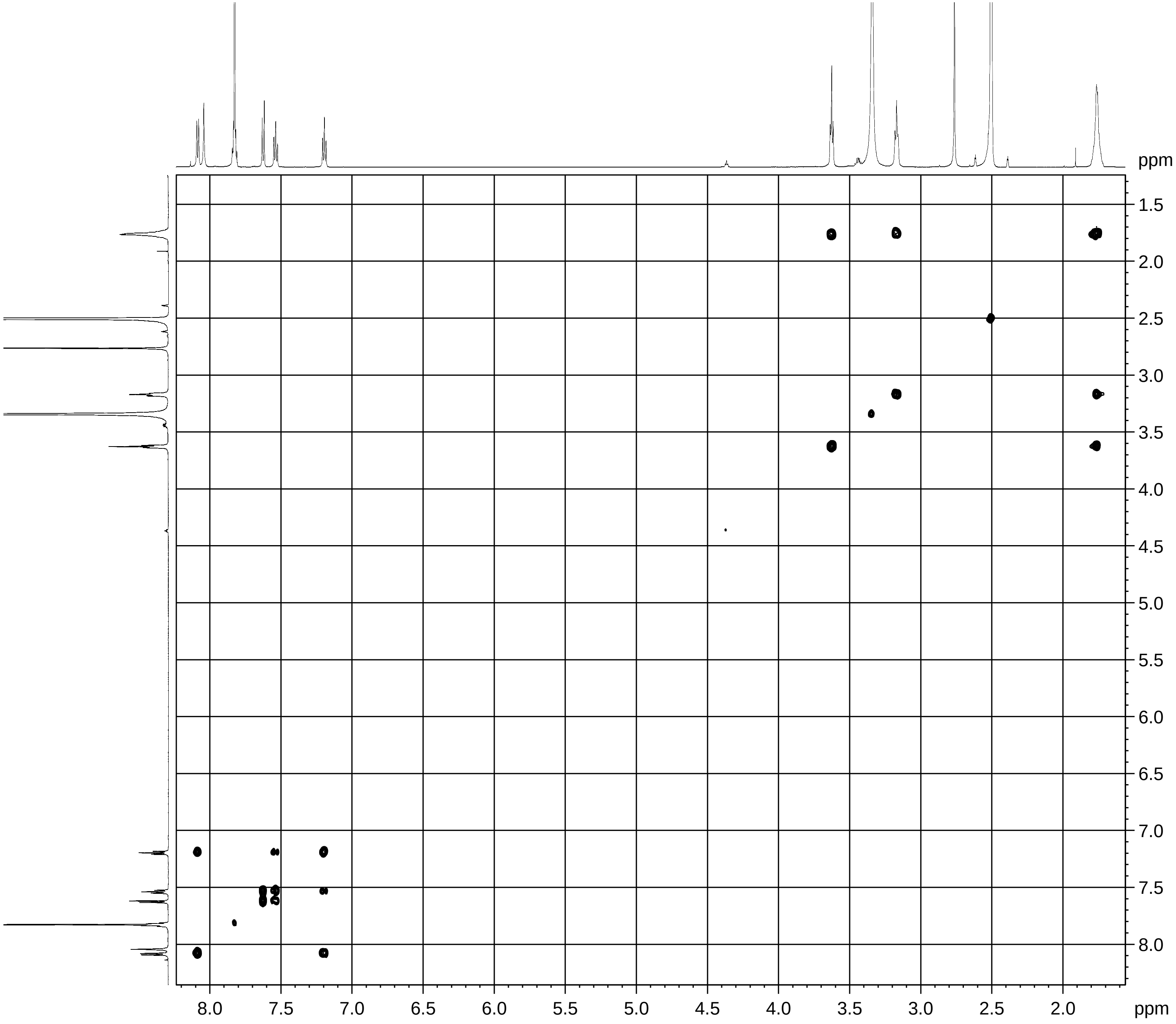

COSY
143668
BAB0334_1
Batizi Benedek
2024.10.07. (KP)

```
Current Data Parameters
NAME              143668
EXPNO                 13
PROCNO                 1

F2 - Acquisition Parameters
Date_           20241007
Time               20.15 h
INSTRUM            spect
PROBHD   Z145856_0002 (
PULPROG      cosygpmfqf
TD                  2048
SOLVENT             DMSO
NS                     8
DS                    16
SWH             7812.500 Hz
FIDRES          7.629395 Hz
AQ             0.1310720 sec
RG                196.07
DW                64.000 usec
DE                 25.00 usec
TE                 295.0 K
D0            0.00000300 sec
D1            2.00000000 sec
D13           0.00000400 sec
D16           0.00020000 sec
IN0           0.00012800 sec
TDav                   1
SFO1        600.0036000 MHz
NUC1                  1H
P1                 11.50 usec
PLW1         28.00000000 W
GPNAM[1]      SMSQ10.100
GPZ1               16.00 %
GPNAM[2]      SMSQ10.100
GPZ2               12.00 %
GPNAM[3]      SMSQ10.100
GPZ3               40.00 %
P16              1000.00 usec

F1 - Acquisition parameters
TD                   256
SFO1         600.0036 MHz
FIDRES       61.035156 Hz
SW              13.021 ppm
FnMODE                QF

F2 - Processing parameters
SI                  1024
SF           600.0000027 MHz
WDW                 SINE
SSB       0
LB        0 Hz
GB        0
PC                  1.40

F1 - Processing parameters
SI                  1024
MC2                   QF
SF           600.0000027 MHz
WDW                 SINE
SSB       0
LB        0 Hz
GB        0
```

24

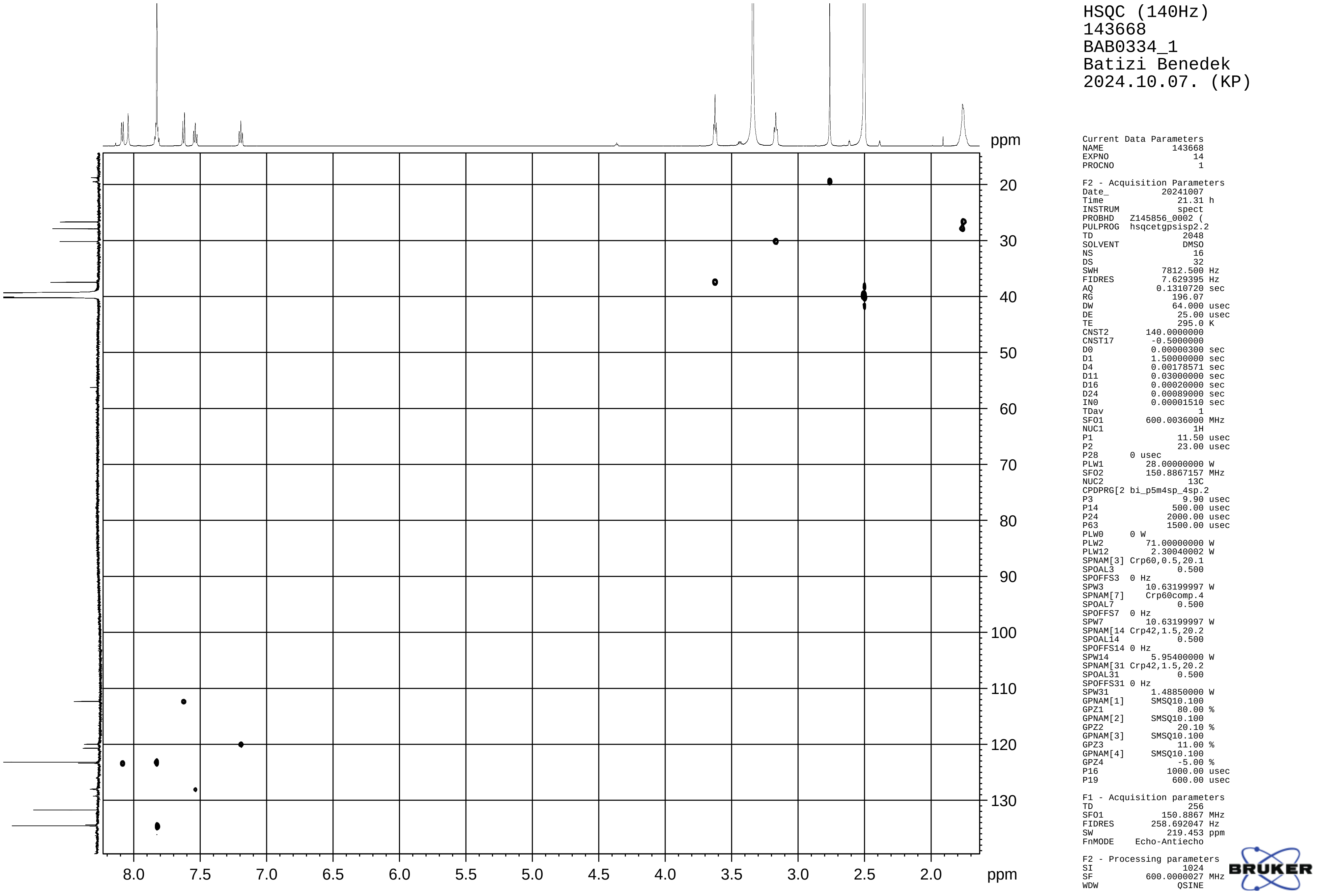
HSQC (140Hz)
143668
BAB0334_1
Batizi Benedek
2024.10.07. (KP)
ppm
20
30
40
50
60
70
80
90
100
110
120
130
8.0
7.5
7.0
6.5
6.0
5.5
5.0
4.5
4.0
3.5
3.0
2.5
2.0
ppm
Current Data Parameters
NAME 143668
EXPNO 14
PROCNO 1
F2 - Acquisition Parameters
Date_ 20241007
Time 21.31 h
INSTRUM spect
PROBHD Z145856_0002 (
PULPROG hsqcetgpsisp2.2
TD 2048
SOLVENT DMSO
NS 16
DS 32
SWH 7812.500 Hz
FIDRES 7.629395 Hz
AQ 0.1310720 sec
RG 196.07
DW 64.000 usec
DE 25.00 usec
TE 295.0 K
CNST2 140.0000000
CNST17 -0.5000000
D0 0.00000300 sec
D1 1.50000000 sec
D4 0.00178571 sec
D11 0.03000000 sec
D16 0.00020000 sec
D24 0.00089000 sec
IN0 0.00001510 sec
TDav 1
SFO1 600.0036000 MHz
NUC1 1H
P1 11.50 usec
P2 23.00 usec
P28 0 usec
PLW1 28.00000000 W
SFO2 150.8867157 MHz
NUC2 13C
CPDPRG[2 bi_p5m4sp_4sp.2
P3 9.90 usec
P14 500.00 usec
P24 2000.00 usec
P63 1500.00 usec
PLW0 0 W
PLW2 71.00000000 W
PLW12 2.30040002 W
SPNAM[3] Crp60,0.5,20.1
SPOAL3 0.500
SPOFFS3 0 Hz
SPW3 10.63199997 W
SPNAM[7] Crp60comp.4
SPOAL7 0.500
SPOFFS7 0 Hz
SPW7 10.63199997 W
SPNAM[14 Crp42,1.5,20.2
SPOAL14 0.500
SPOFFS14 0 Hz
SPW14 5.95400000 W
SPNAM[31 Crp42,1.5,20.2
SPOAL31 0.500
SPOFFS31 0 Hz
SPW31 1.48850000 W
GPNAM[1] SMSQ10.100
GPZ1 80.00 %
GPNAM[2] SMSQ10.100
GPZ2 20.10 %
GPNAM[3] SMSQ10.100
GPZ3 11.00 %
GPNAM[4] SMSQ10.100
GPZ4 -5.00 %
P16 1000.00 usec
P19 600.00 usec
F1 - Acquisition parameters
TD 256
SFO1 150.8867 MHz
FIDRES 258.692047 Hz
SW 219.453 ppm
FnMODE Echo-Antiecho
F2 - Processing parameters
SI 1024
SF 600.0000027 MHz
WDW QSINE
BRUKER

24

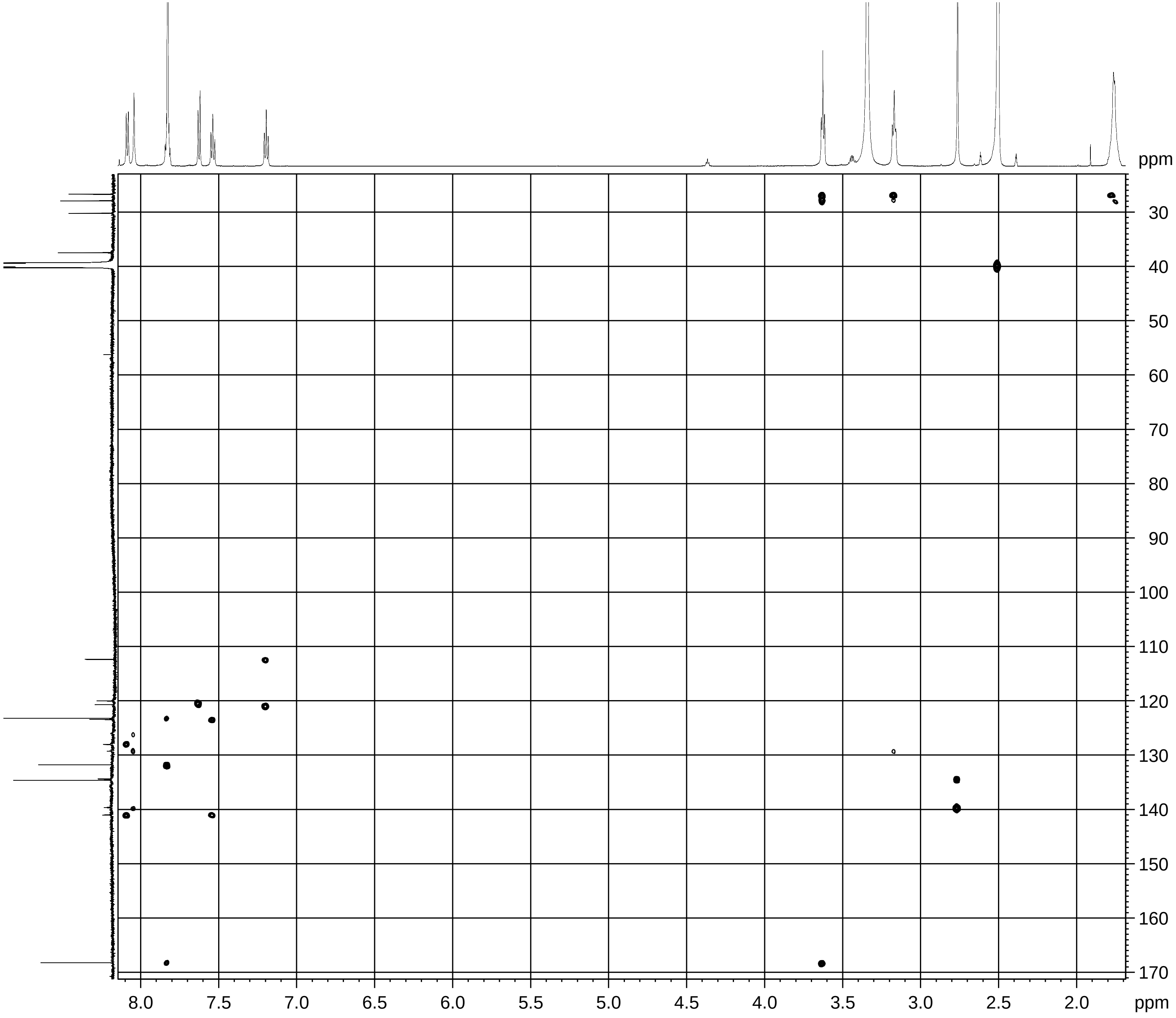

HMBC (8Hz, 140Hz)
143668
BAB0334_1
Batizi Benedek
2024.10.07. (KP)

```
Current Data Parameters
NAME              143668
EXPNO                 15
PROCNO                 1

F2 - Acquisition Parameters
Date_          20241007
Time              23.26 h
INSTRUM           spect
PROBHD   Z145856_0002 (
PULPROG    hmbcgplpndqf
TD                 2048
SOLVENT            DMSO
NS                   16
DS                   16
SWH            7812.500 Hz
FIDRES         7.629395 Hz
AQ            0.1310720 sec
RG               196.07
DW               64.000 usec
DE                25.00 usec
TE                295.0 K
CNST2       140.0000000
CNST13        8.0000000
D0           0.00000300 sec
D1           1.50000000 sec
D2           0.00357143 sec
D6           0.06250000 sec
D16          0.00020000 sec
IN0          0.00001510 sec
TDav                  1
SFO1        600.0037800 MHz
NUC1                 1H
P1                11.50 usec
P2                23.00 usec
PLW1        28.00000000 W
SFO2        150.8867157 MHz
NUC2                13C
P3                 9.90 usec
PLW2        71.00000000 W
GPNAM[1]     SMSQ10.100
GPZ1              50.00 %
GPNAM[2]     SMSQ10.100
GPZ2              30.00 %
GPNAM[3]     SMSQ10.100
GPZ3              40.10 %
P16             1000.00 usec

F1 - Acquisition parameters
TD                  256
SFO1           150.8867 MHz
FIDRES       258.692047 Hz
SW              219.453 ppm
FnMODE               QF

F2 - Processing parameters
SI                 2048
SF          600.0000000 MHz
WDW                SINE
SSB      0
LB       0 Hz
GB       0
PC                 1.40

F1 - Processing parameters
SI                 1024
MC2                  QF
SF          150.8701200 MHz
WDW                SINE
SSB      0
LB       0 Hz
GB       0
```

25

Batizi Benedek	KP
Gyemant ATR	2023.10.17.

BRUKER Alpha
Resolution: 2 cm-1
Number of Scans: 32

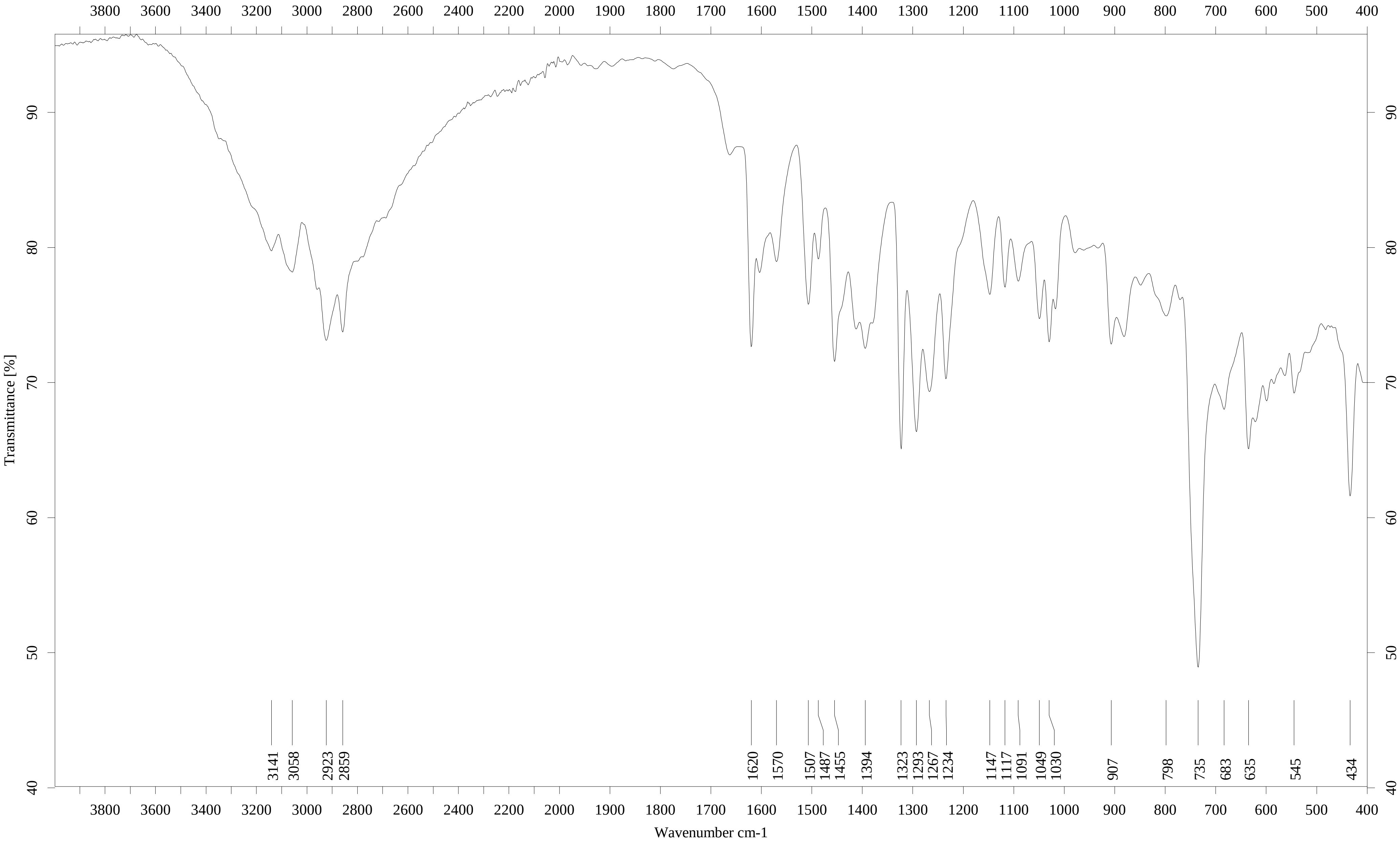

25

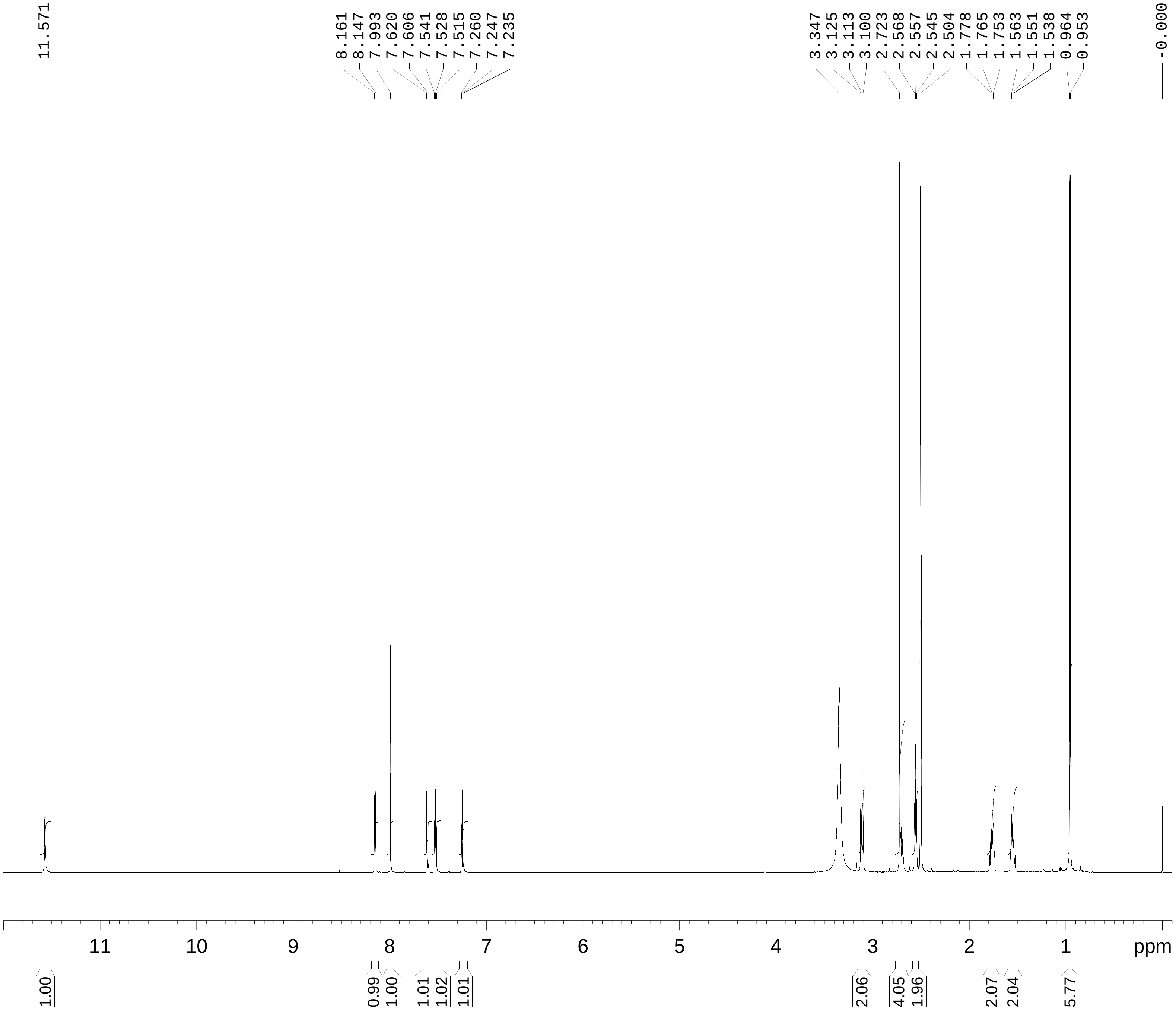

Standard 1H
142472
BAB0140_1
Batizi Benedek
2024.02.21. (KP)

Current Data Parameters

NAME	142472
EXPNO	11
PROCNO	1

F2 - Acquisition Parameters

Date_	20240221
Time	23.04 h
INSTRUM	spect
PROBHD	Z145856_0002 (
PULPROG	zg30
TD	65536
SOLVENT	DMSO
NS	16
DS	2
SWH	12019.230 Hz
FIDRES	0.366798 Hz
AQ	2.7262976 sec
RG	196.07
DW	41.600 usec
DE	25.00 usec
TE	295.0 K
D1	1.00000000 sec
TD0	1
SFO1	600.0037050 MHz
NUC1	1H
P1	11.50 usec
PLW1	28.00000000 W

F2 - Processing parameters

SI	65536
SF	600.0000022 MHz
WDW	no
SSB	0
LB	0 Hz
GB	0
PC	1.00

25

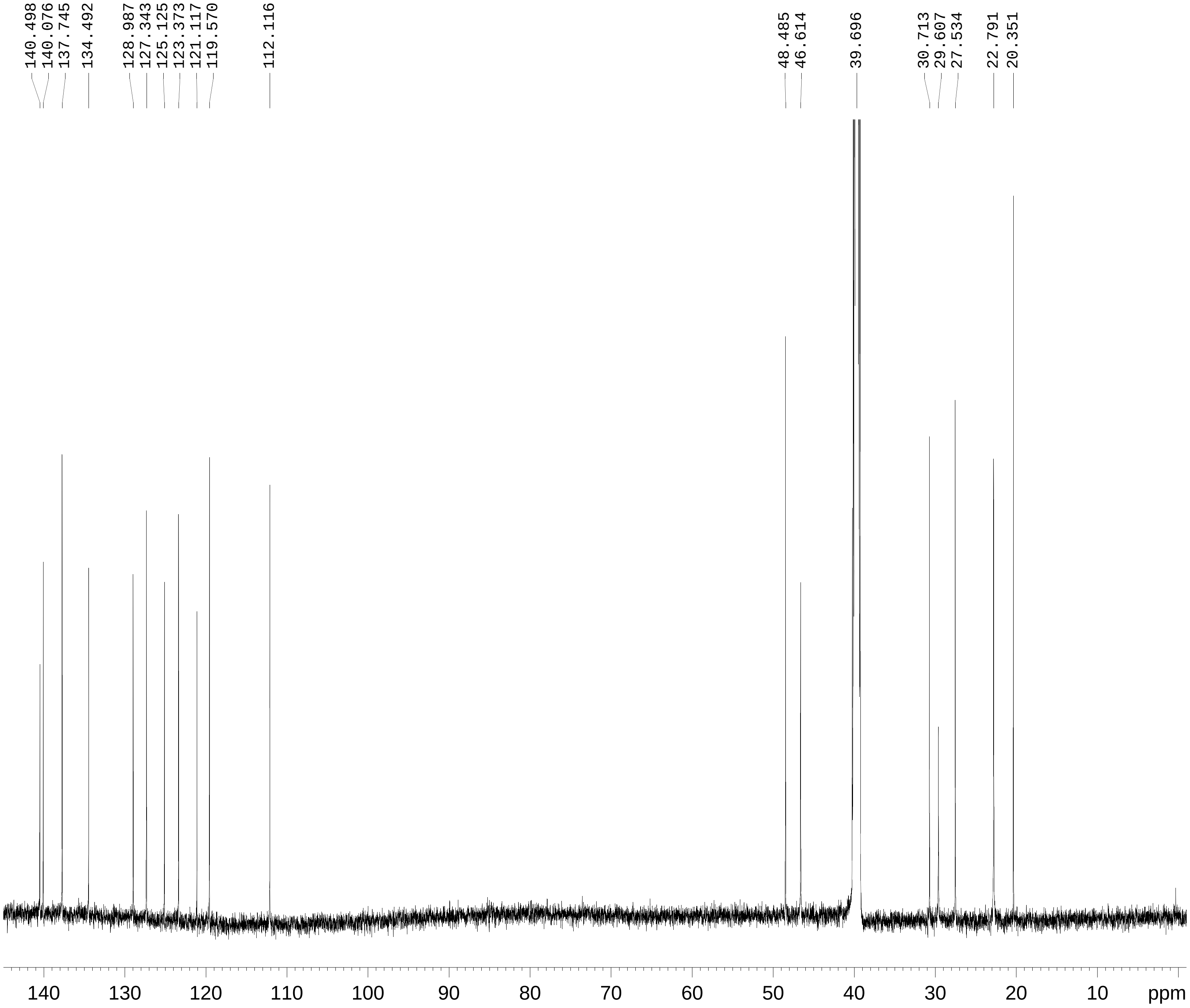

Standard 13C
142472
BAB0140_1
Batizi Benedek
2024.02.21. (KP)

Current Data Parameters

NAME	142472	
EXPNO	12	
PROCNO	1	

F2 - Acquisition Parameters

Date_	20240222	
Time	0.13	h
INSTRUM	spect	
PROBHD	Z145856_0002 (	
PULPROG	zgpg30	
TD	65536	
SOLVENT	DMSO	
NS	2048	
DS	4	
SWH	36231.883	Hz
FIDRES	1.105709	Hz
AQ	0.9043968	sec
RG	196.07	
DW	13.800	usec
DE	18.00	usec
TE	295.0	K
D1	1.00000000	sec
D11	0.03000000	sec
TD0	1	
SFO1	150.8852070	MHz
NUC1	13C	
P1	9.90	usec
PLW1	71.00000000	W
SFO2	600.0024000	MHz
NUC2	1H	
CPDPRG[2	waltz16	
PCPD2	80.00	usec
PLW2	32.90000153	W
PLW12	0.70370001	W
PLW13	0.35339001	W

F2 - Processing parameters

SI	32768	
SF	150.8701596	MHz
WDW	EM	
SSB	0	
LB	1.00	Hz
GB	0	
PC	1.40	

BRUKER

25

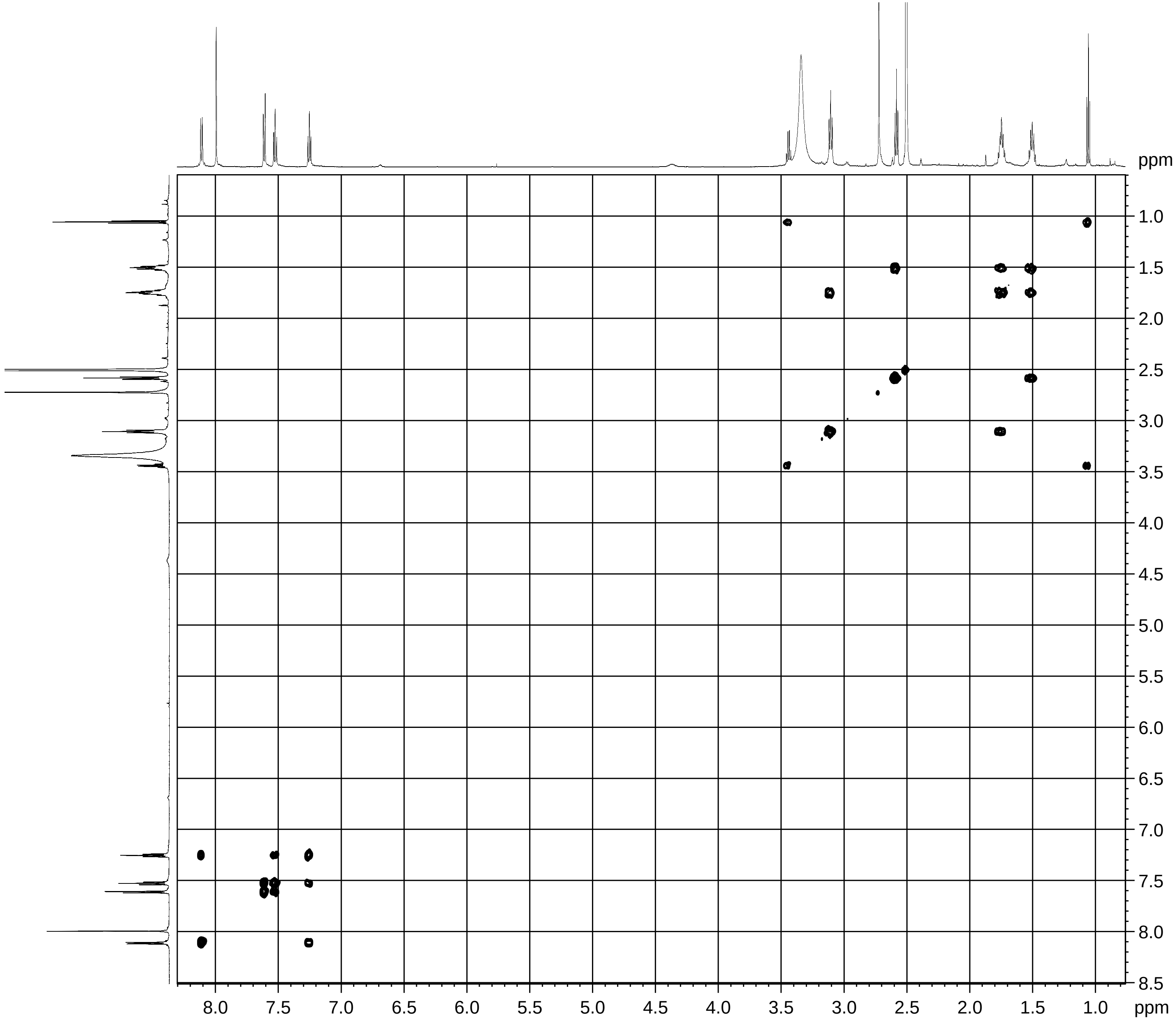

COSY
142050
BAB0085_1
Batizi Benedek
2023.10.17. (KP)

Current Data Parameters
NAME 142050
EXPNO 13
PROCNO 1

F2 - Acquisition Parameters
Date_ 20231017
Time 17.42 h
INSTRUM spect
PROBHD Z145856_0002 (
PULPROG cosygpmfqf
TD 2048
SOLVENT DMSO
NS 8
DS 16
SWH 7812.500 Hz
FIDRES 7.629395 Hz
AQ 0.1310720 sec
RG 196.07
DW 64.000 usec
DE 25.00 usec
TE 295.0 K
D0 0.00000300 sec
D1 2.00000000 sec
D13 0.00000400 sec
D16 0.00020000 sec
IN0 0.00012800 sec
TDav 1
SFO1 600.0036000 MHz
NUC1 1H
P1 11.50 usec
PLW1 28.00000000 W
GPNAM[1] SMSQ10.100
GPZ1 16.00 %
GPNAM[2] SMSQ10.100
GPZ2 12.00 %
GPNAM[3] SMSQ10.100
GPZ3 40.00 %
P16 1000.00 usec

F1 - Acquisition parameters
TD 256
SFO1 600.0036 MHz
FIDRES 61.035156 Hz
SW 13.021 ppm
FnMODE QF

F2 - Processing parameters
SI 1024
SF 600.0000037 MHz
WDW SINE
SSB 0
LB 0 Hz
GB 0
PC 1.40

F1 - Processing parameters
SI 1024
MC2 QF
SF 600.0000037 MHz
WDW SINE
SSB 0
LB 0 Hz
GB 0

25

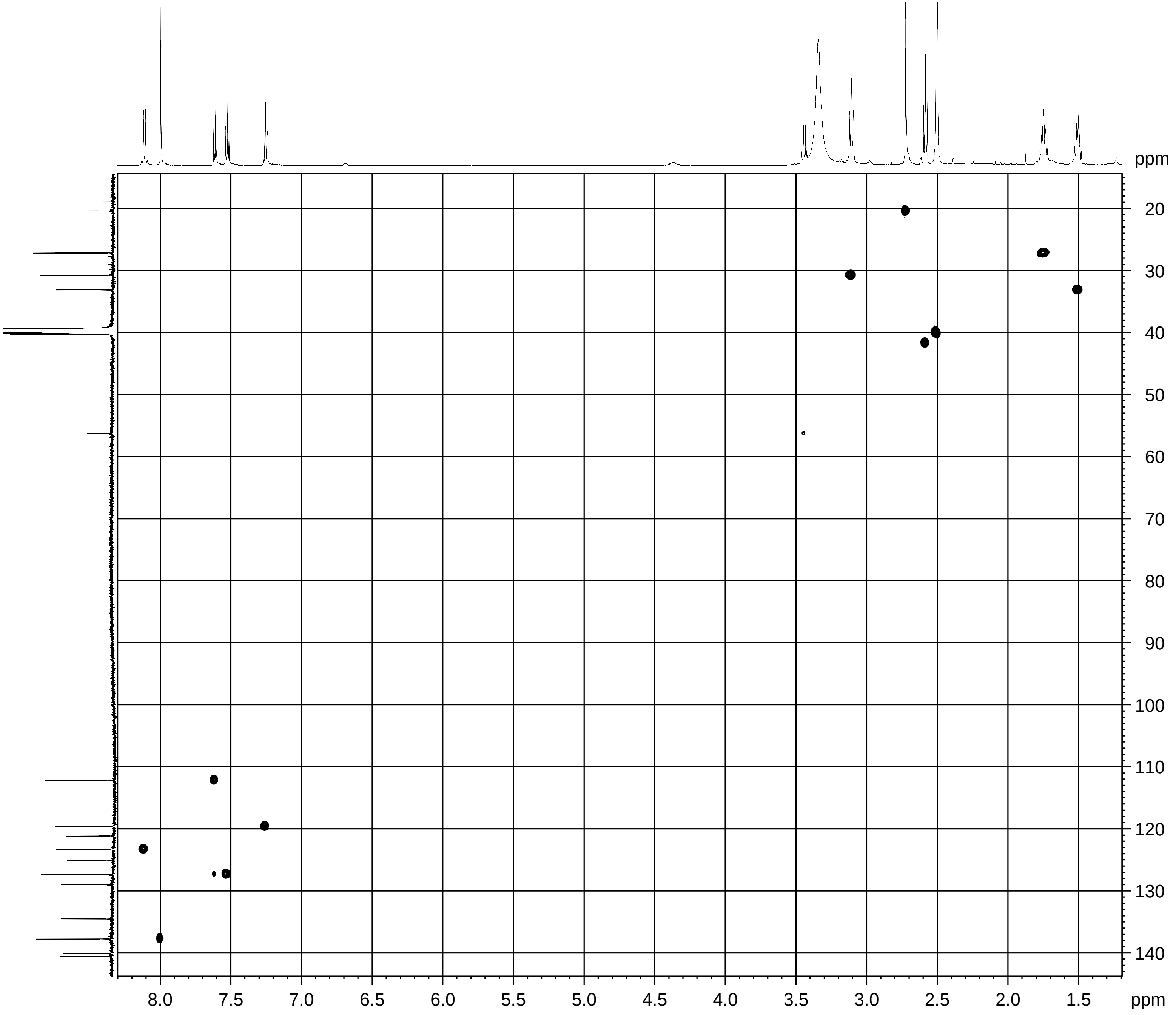

HSQC (140Hz)
142050
BAB0085_1
Batizi Benedek
2023.10.17. (KP)

```
Current Data Parameters
NAME              142050
EXPNO                 14
PROCNO                 1

F2 - Acquisition Parameters
Date_           20231017
Time               18.57 h
INSTRUM            spect
PROBHD   Z145856_0002 (
PULPROG  hsqcetgpsisp2.2
TD                  2048
SOLVENT             DMSO
NS                    16
DS                    32
SWH             7812.500 Hz
FIDRES          7.629395 Hz
AQ             0.1310720 sec
RG                196.07
DW                64.000 usec
DE                 25.00 usec
TE                 295.0 K
CNST2        140.0000000
CNST17        -0.5000000
D0            0.00000300 sec
D1            1.50000000 sec
D4            0.00178571 sec
D11           0.03000000 sec
D16           0.00020000 sec
D24           0.00089000 sec
IN0           0.00001510 sec
TDav                   1
SFO1        600.0036000 MHz
NUC1                  1H
P1                 11.50 usec
P2                 23.00 usec
P28      0 usec
PLW1         28.00000000 W
SFO2        150.8867157 MHz
NUC2                 13C
CPDPRG[2 bi_p5m4sp_4sp.2
P3                  9.90 usec
P14               500.00 usec
P24              2000.00 usec
P63              1500.00 usec
PLW0     0 W
PLW2         80.09999847 W
PLW12         2.59520006 W
SPNAM[3] Crp60,0.5,20.1
SPOAL3             0.500
SPOFFS3  0 Hz
SPW3         11.99499989 W
SPNAM[7]    Crp60comp.4
SPOAL7             0.500
SPOFFS7  0 Hz
SPW7         11.99499989 W
SPNAM[14 Crp42,1.5,20.2
SPOAL14            0.500
SPOFFS14 0 Hz
SPW14         6.71710014 W
SPNAM[31 Crp42,1.5,20.2
SPOAL31            0.500
SPOFFS31 0 Hz
SPW31         1.67929995 W
GPNAM[1]      SMSQ10.100
GPZ1               80.00 %
GPNAM[2]      SMSQ10.100
GPZ2               20.10 %
GPNAM[3]      SMSQ10.100
GPZ3               11.00 %
GPNAM[4]      SMSQ10.100
GPZ4               -5.00 %
P16              1000.00 usec
P19               600.00 usec

F1 - Acquisition parameters
TD                   256
SFO1        150.8867 MHz
FIDRES    258.692047 Hz
SW           219.453 ppm
FnMODE    Echo-Antiecho

F2 - Processing parameters
SI                  1024
SF        600.0000037 MHz
WDW                QSINE
```

25

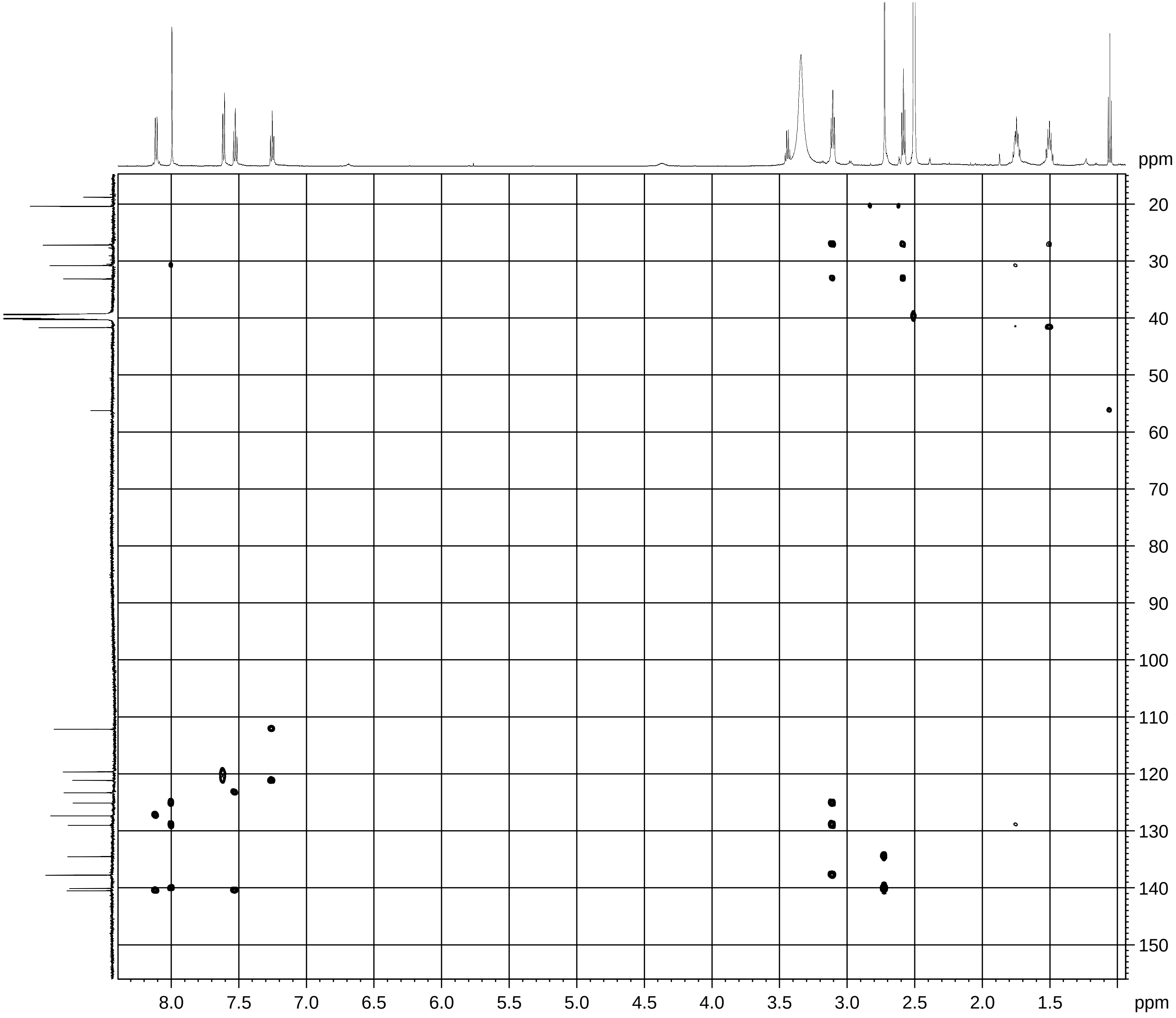

HMBC (8Hz, 140Hz)
142050
BAB0085_1
Batizi Benedek
2023.10.17. (KP)

Current Data Parameters
NAME 142050
EXPNO 15
PROCNO 1

F2 - Acquisition Parameters
Date_ 20231017
Time 20.52 h
INSTRUM spect
PROBHD Z145856_0002 (
PULPROG hmbcgplpndqf
TD 2048
SOLVENT DMSO
NS 16
DS 16
SWH 7812.500 Hz
FIDRES 7.629395 Hz
AQ 0.1310720 sec
RG 196.07
DW 64.000 usec
DE 25.00 usec
TE 295.0 K
CNST2 140.0000000
CNST13 8.0000000
D0 0.00000300 sec
D1 1.50000000 sec
D2 0.00357143 sec
D6 0.06250000 sec
D16 0.00020000 sec
IN0 0.00001510 sec
TDav 1
SFO1 600.0037800 MHz
NUC1 1H
P1 11.50 usec
P2 23.00 usec
PLW1 28.00000000 W
SFO2 150.8867157 MHz
NUC2 13C
P3 9.90 usec
PLW2 80.09999847 W
GPNAM[1] SMSQ10.100
GPZ1 50.00 %
GPNAM[2] SMSQ10.100
GPZ2 30.00 %
GPNAM[3] SMSQ10.100
GPZ3 40.10 %
P16 1000.00 usec

F1 - Acquisition parameters
TD 256
SFO1 150.8867 MHz
FIDRES 258.692047 Hz
SW 219.453 ppm
FnMODE QF

F2 - Processing parameters
SI 2048
SF 600.0000037 MHz
WDW SINE
SSB 0
LB 0 Hz
GB 0
PC 1.40

F1 - Processing parameters
SI 1024
MC2 QF
SF 150.8701601 MHz
WDW SINE
SSB 0
LB 0 Hz
GB 0

25

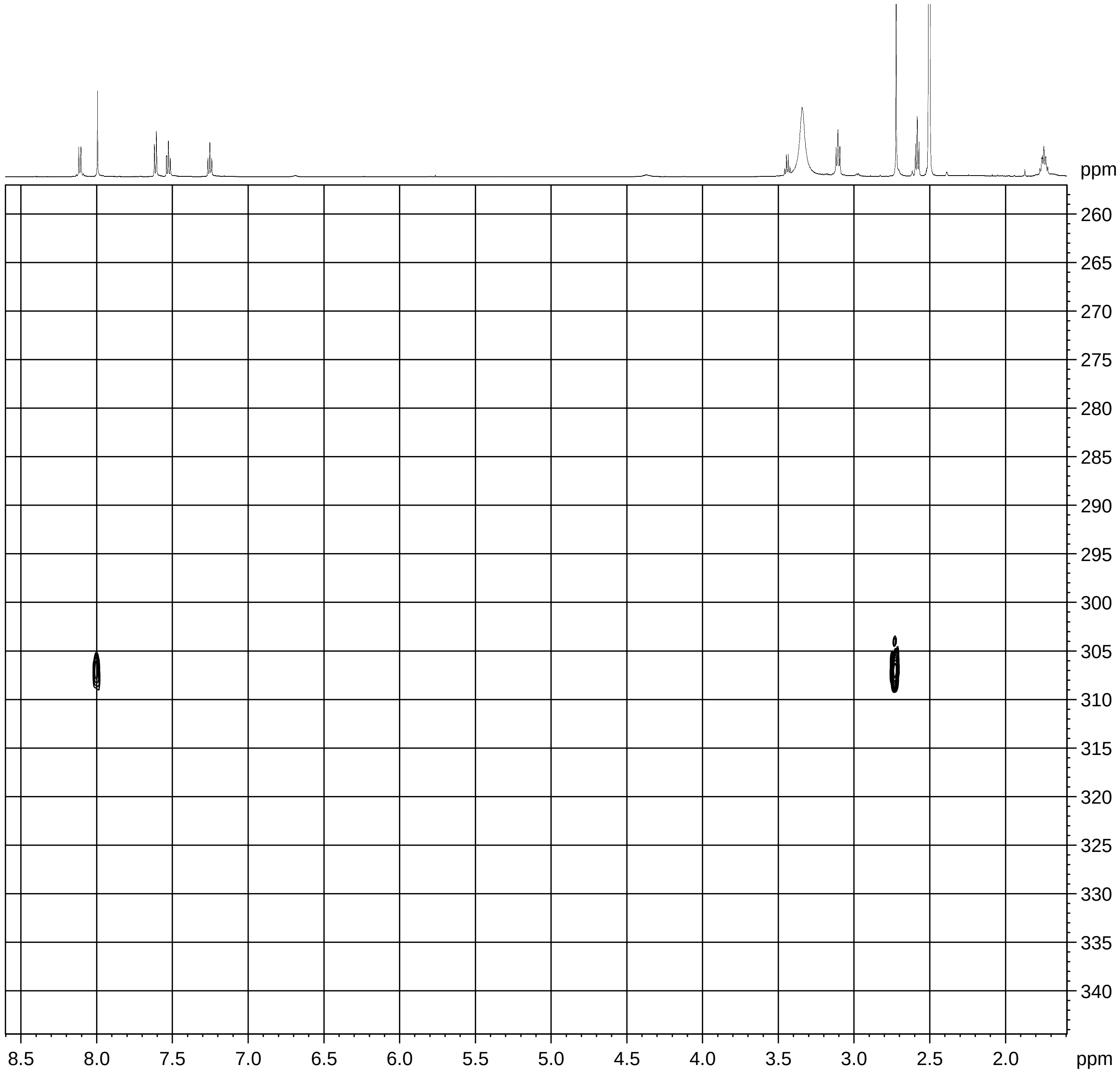

NHMBC
142050
BAB0085_1
Batizi Benedek
2023.10.17. (KP)

```
Current Data Parameters
NAME              142050
EXPNO                 16
PROCNO                 1

F2 - Acquisition Parameters
Date_           20231017
Time               22.51 h
INSTRUM            spect
PROBHD   Z145856_0002 (
PULPROG       hmbcgpndqf
TD                  2048
SOLVENT             DMSO
NS                    16
DS                    16
SWH             9615.385 Hz
FIDRES          9.390024 Hz
AQ             0.1064960 sec
RG                196.07
DW                52.000 usec
DE                 25.00 usec
TE                 295.0 K
CNST13         5.0000000
D0            0.00000300 sec
D1            2.00000000 sec
D6            0.10000000 sec
D16           0.00020000 sec
IN0           0.00002060 sec
TDav                   1
SFO1         600.0045600 MHz
NUC1                  1H
P1                 11.50 usec
P2                 23.00 usec
PLW1         28.00000000 W
SFO2          60.8096315 MHz
NUC2                 15N
P3                 14.60 usec
PLW2        236.00000000 W
GPNAM[1]      SMSQ10.100
GPZ1               70.00 %
GPNAM[2]      SMSQ10.100
GPZ2               30.00 %
GPNAM[3]      SMSQ10.100
GPZ3               50.10 %
P16              1000.00 usec

F1 - Acquisition parameters
TD                   128
SFO1            60.80963 MHz
FIDRES        379.247559 Hz
SW               399.145 ppm
FnMODE                QF

F2 - Processing parameters
SI                  2048
SF           600.0000037 MHz
WDW                 SINE
SSB      0
LB       0 Hz
GB       0
PC                  1.40

F1 - Processing parameters
SI                  1024
MC2                   QF
SF            60.7974720 MHz
WDW                 SINE
SSB      0
LB       0 Hz
GB       0
```

26

Batizi Benedek	KP
KBr	10/25/2024

BRUKER Alpha
Resolution: 2 cm-1
Number of Scans: 16

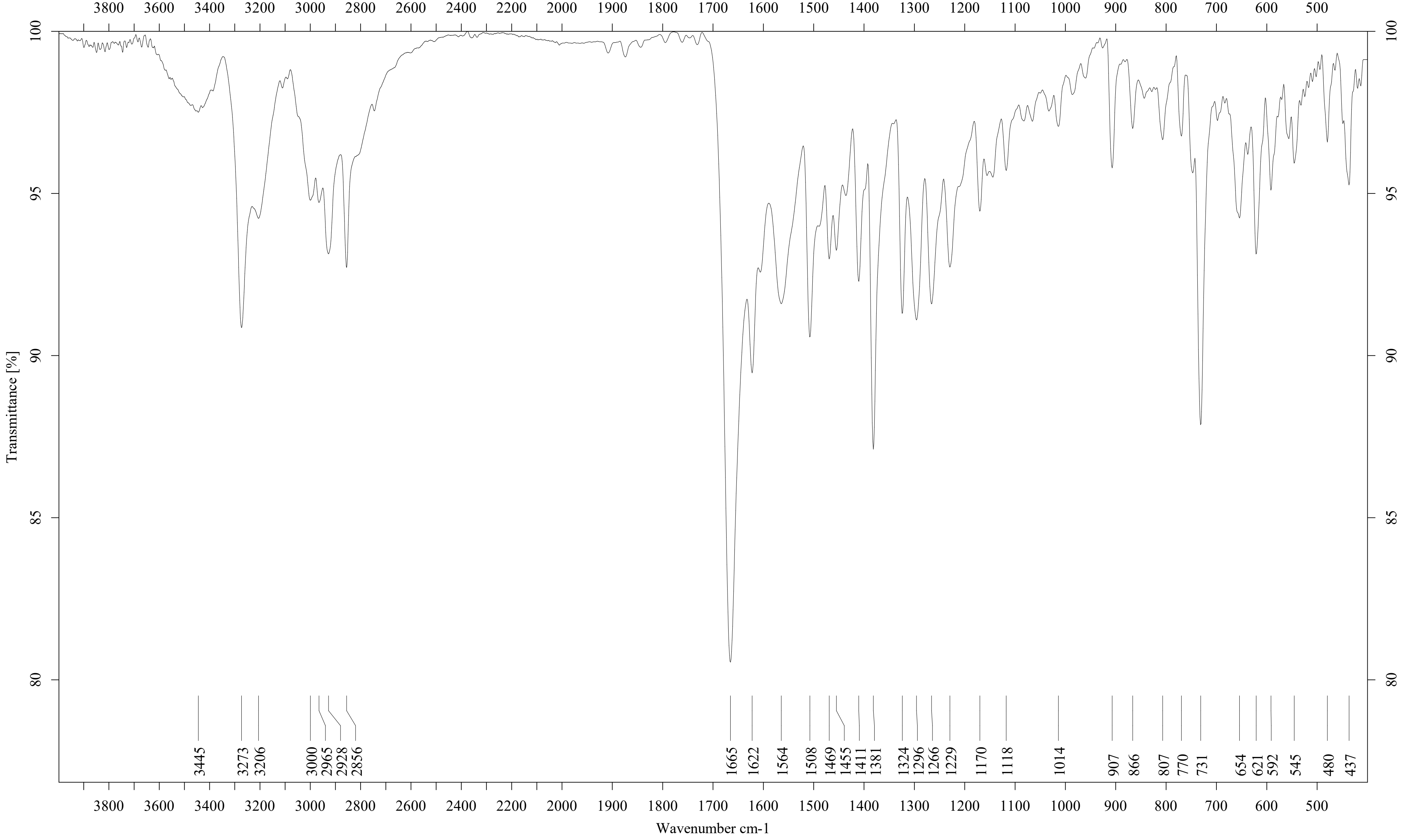

26

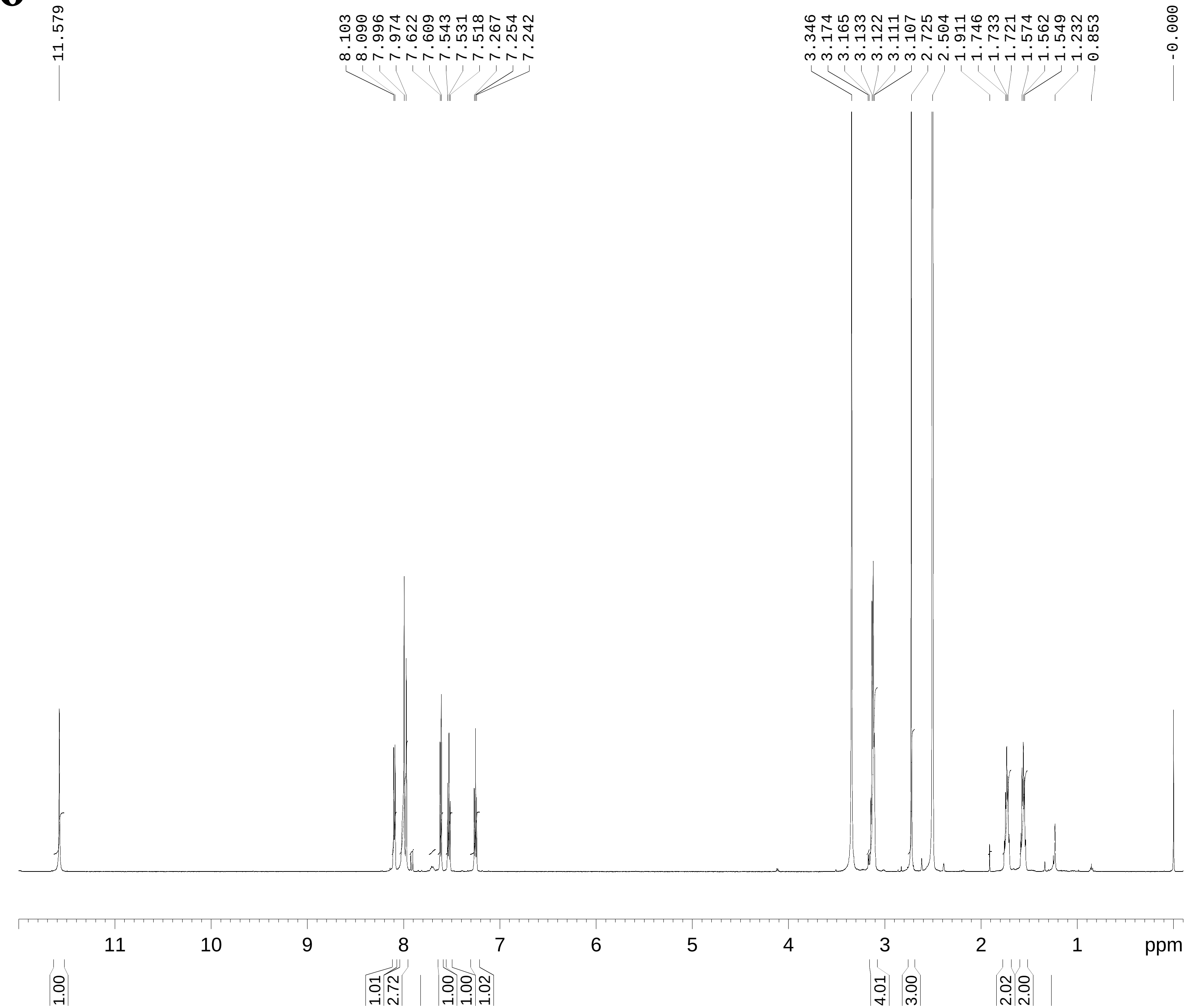

Standard 1H
143759
BAB0345_1
Batizi Benedek
2024.10.25. (KP)

Current Data Parameters
NAME 143759
EXPNO 11
PROCNO 1

F2 - Acquisition Parameters
Date_ 20241025
Time 20.40 h
INSTRUM spect
PROBHD Z145856_0002 (
PULPROG zg30
TD 65536
SOLVENT DMSO
NS 64
DS 2
SWH 12019.230 Hz
FIDRES 0.366798 Hz
AQ 2.7262976 sec
RG 196.07
DW 41.600 usec
DE 25.00 usec
TE 295.0 K
D1 1.00000000 sec
TD0 1
SFO1 600.0037050 MHz
NUC1 1H
P1 11.50 usec
PLW1 28.00000000 W

F2 - Processing parameters
SI 65536
SF 600.0000025 MHz
WDW EM
SSB 0
LB 0.30 Hz
GB 0
PC 1.00

26

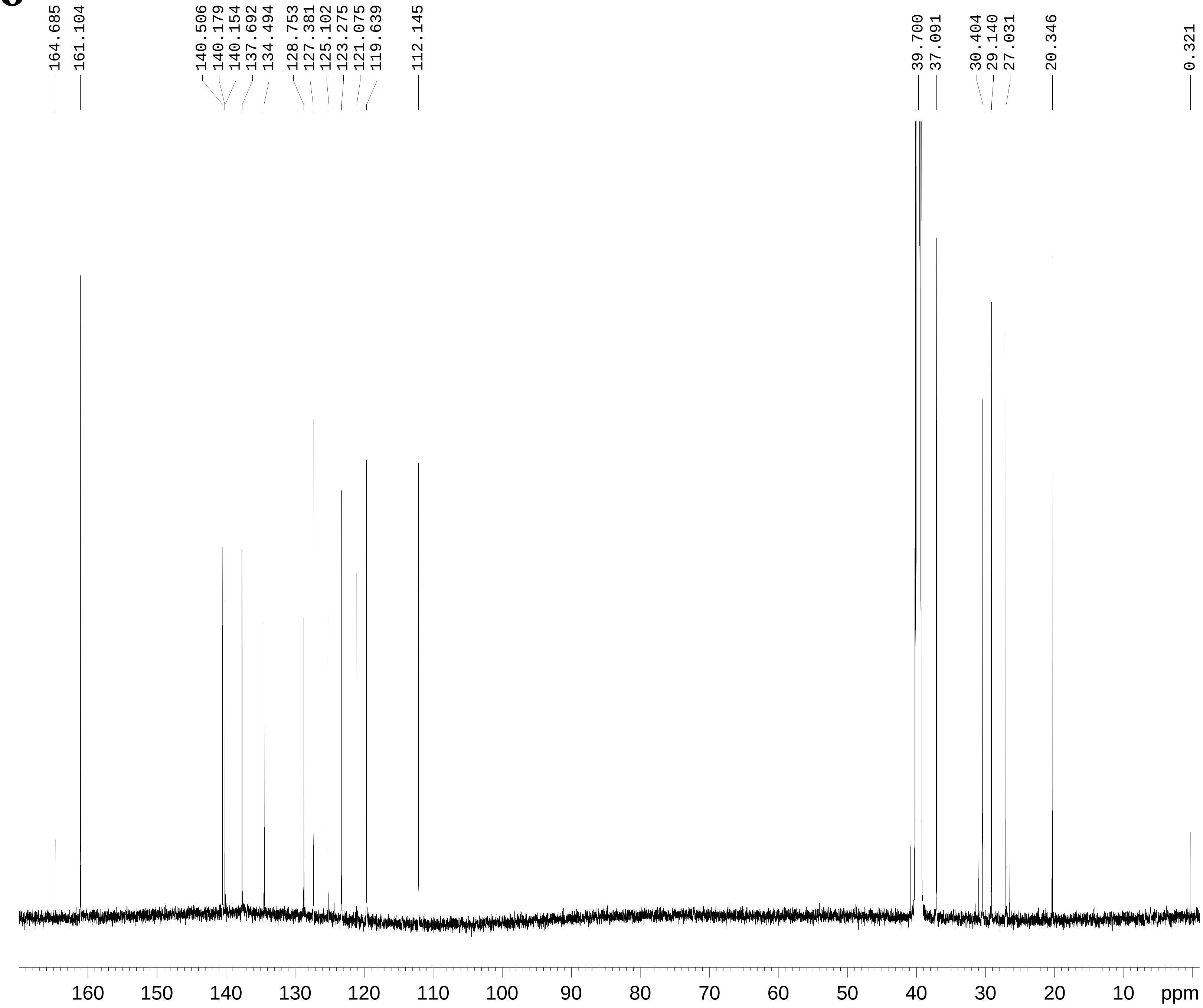

Standard 13C
143759
BAB0345_1
Batizi Benedek
2024.10.25. (KP)

Current Data Parameters

NAME	143759
EXPNO	12
PROCNO	1

F2 - Acquisition Parameters

Date_	20241025
Time	22.57 h
INSTRUM	spect
PROBHD	Z145856_0002 (
PULPROG	zgpg30
TD	65536
SOLVENT	DMSO
NS	4096
DS	4
SWH	36231.883 Hz
FIDRES	1.105709 Hz
AQ	0.9043968 sec
RG	196.07
DW	13.800 usec
DE	18.00 usec
TE	295.0 K
D1	1.00000000 sec
D11	0.03000000 sec
TD0	1
SFO1	150.8852070 MHz
NUC1	13C
P1	9.90 usec
PLW1	71.00000000 W
SFO2	600.0024000 MHz
NUC2	1H
CPDPRG[2	waltz16
PCPD2	80.00 usec
PLW2	32.90000153 W
PLW12	0.70370001 W
PLW13	0.35339001 W

F2 - Processing parameters

SI	131072
SF	150.8701601 MHz
WDW	EM
SSB	0
LB	1.00 Hz
GB	0
PC	1.40

26

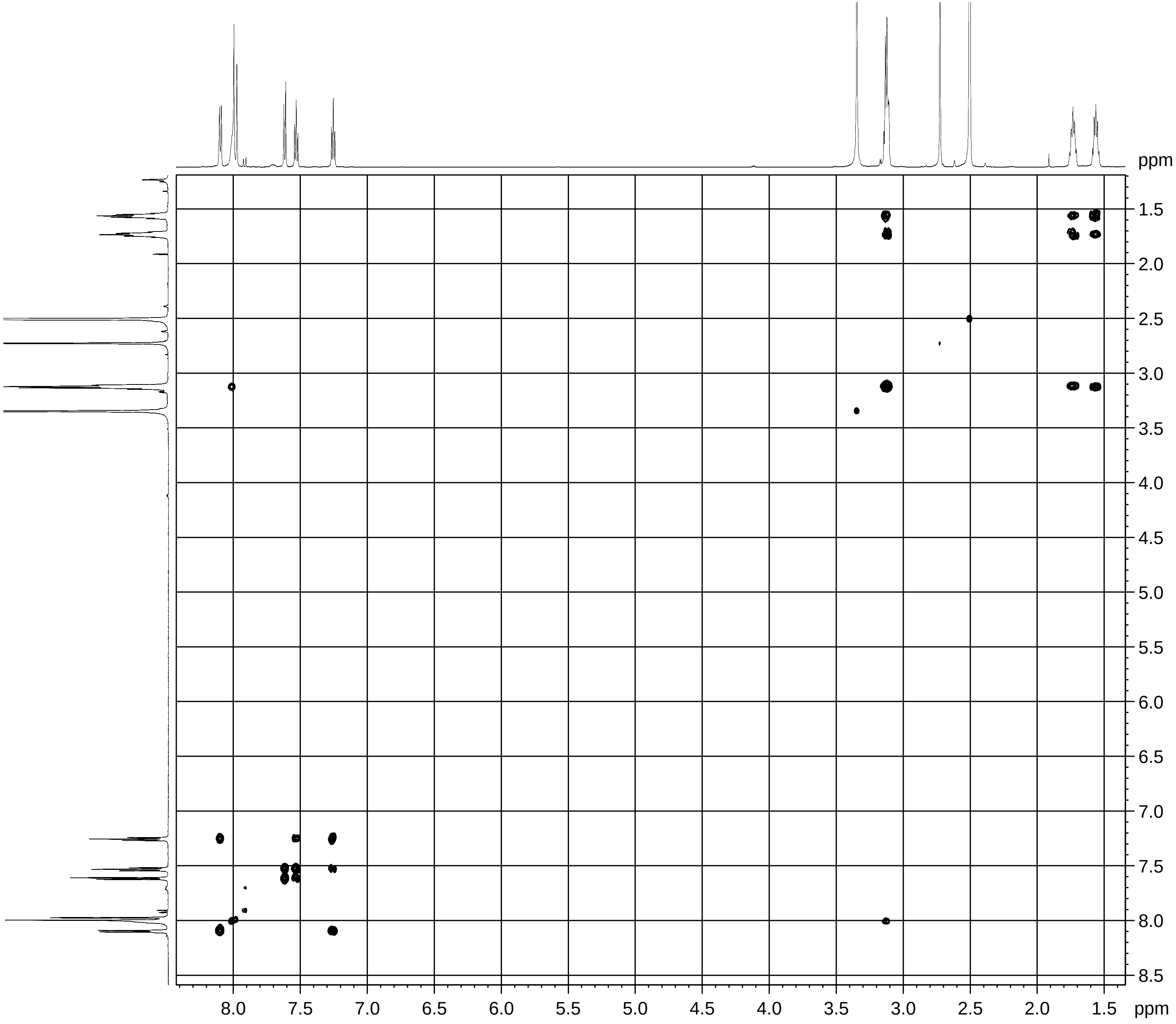

COSY
143759
BAB0345_1
Batizi Benedek
2024.10.25. (KP)

Current Data Parameters
NAME 143759
EXPNO 13
PROCNO 1

F2 - Acquisition Parameters
Date_ 20241025
Time 22.59 h
INSTRUM spect
PROBHD Z145856_0002 (
PULPROG cosygpmfqf
TD 2048
SOLVENT DMSO
NS 4
DS 16
SWH 7812.500 Hz
FIDRES 7.629395 Hz
AQ 0.1310720 sec
RG 196.07
DW 64.000 usec
DE 25.00 usec
TE 295.0 K
D0 0.00000300 sec
D1 2.00000000 sec
D13 0.00000400 sec
D16 0.00020000 sec
IN0 0.00012800 sec
TDav 1
SFO1 600.0036000 MHz
NUC1 1H
P1 11.50 usec
PLW1 28.00000000 W
GPNAM[1] SMSQ10.100
GPZ1 16.00 %
GPNAM[2] SMSQ10.100
GPZ2 12.00 %
GPNAM[3] SMSQ10.100
GPZ3 40.00 %
P16 1000.00 usec

F1 - Acquisition parameters
TD 256
SFO1 600.0036 MHz
FIDRES 61.035156 Hz
SW 13.021 ppm
FnMODE QF

F2 - Processing parameters
SI 1024
SF 600.0000025 MHz
WDW SINE
SSB 0
LB 0 Hz
GB 0
PC 1.40

F1 - Processing parameters
SI 1024
MC2 QF
SF 600.0000025 MHz
WDW SINE
SSB 0
LB 0 Hz
GB 0

26

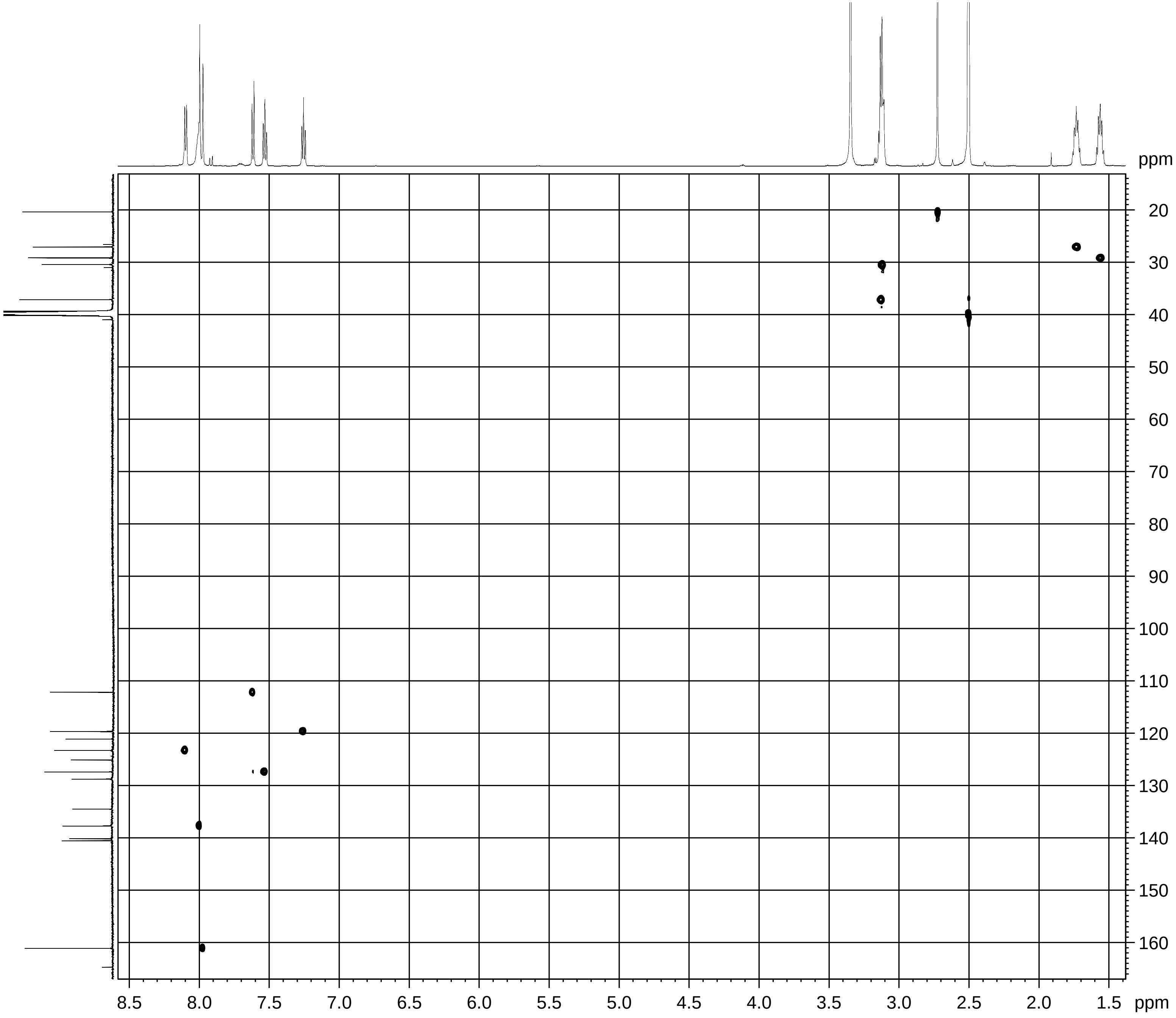

HSQC (140Hz)
143759
BAB0345_1
Batizi Benedek
2024.10.25. (KP)

```
Current Data Parameters
NAME             143759
EXPNO                14
PROCNO                1

F2 - Acquisition Parameters
Date_          20241025
Time              23.37 h
INSTRUM           spect
PROBHD   Z145856_0002 (
PULPROG  hsqcetgpsisp2.2
TD                 2048
SOLVENT            DMSO
NS                    8
DS                   32
SWH            7812.500 Hz
FIDRES         7.629395 Hz
AQ            0.1310720 sec
RG               196.07
DW               64.000 usec
DE                25.00 usec
TE                295.0 K
CNST2       140.0000000
CNST17       -0.5000000
D0           0.00000300 sec
D1           1.50000000 sec
D4           0.00178571 sec
D11          0.03000000 sec
D16          0.00020000 sec
D24          0.00089000 sec
IN0          0.00001510 sec
TDav                  1
SFO1        600.0036000 MHz
NUC1                 1H
P1                11.50 usec
P2                23.00 usec
P28      0 usec
PLW1        28.00000000 W
SFO2        150.8867157 MHz
NUC2                13C
CPDPRG[2 bi_p5m4sp_4sp.2
P3                 9.90 usec
P14              500.00 usec
P24             2000.00 usec
P63             1500.00 usec
PLW0     0 W
PLW2        71.00000000 W
PLW12        2.30040002 W
SPNAM[3] Crp60,0.5,20.1
SPOAL3            0.500
SPOFFS3  0 Hz
SPW3        10.63199997 W
SPNAM[7]    Crp60comp.4
SPOAL7            0.500
SPOFFS7  0 Hz
SPW7        10.63199997 W
SPNAM[14 Crp42,1.5,20.2
SPOAL14           0.500
SPOFFS14 0 Hz
SPW14        5.95400000 W
SPNAM[31 Crp42,1.5,20.2
SPOAL31           0.500
SPOFFS31 0 Hz
SPW31        1.48850000 W
GPNAM[1]     SMSQ10.100
GPZ1              80.00 %
GPNAM[2]     SMSQ10.100
GPZ2              20.10 %
GPNAM[3]     SMSQ10.100
GPZ3              11.00 %
GPNAM[4]     SMSQ10.100
GPZ4              -5.00 %
P16             1000.00 usec
P19              600.00 usec

F1 - Acquisition parameters
TD                  256
SFO1           150.8867 MHz
FIDRES       258.692047 Hz
SW              219.453 ppm
FnMODE    Echo-Antiecho

F2 - Processing parameters
SI                 1024
SF          600.0000025 MHz
WDW               QSINE
```

26

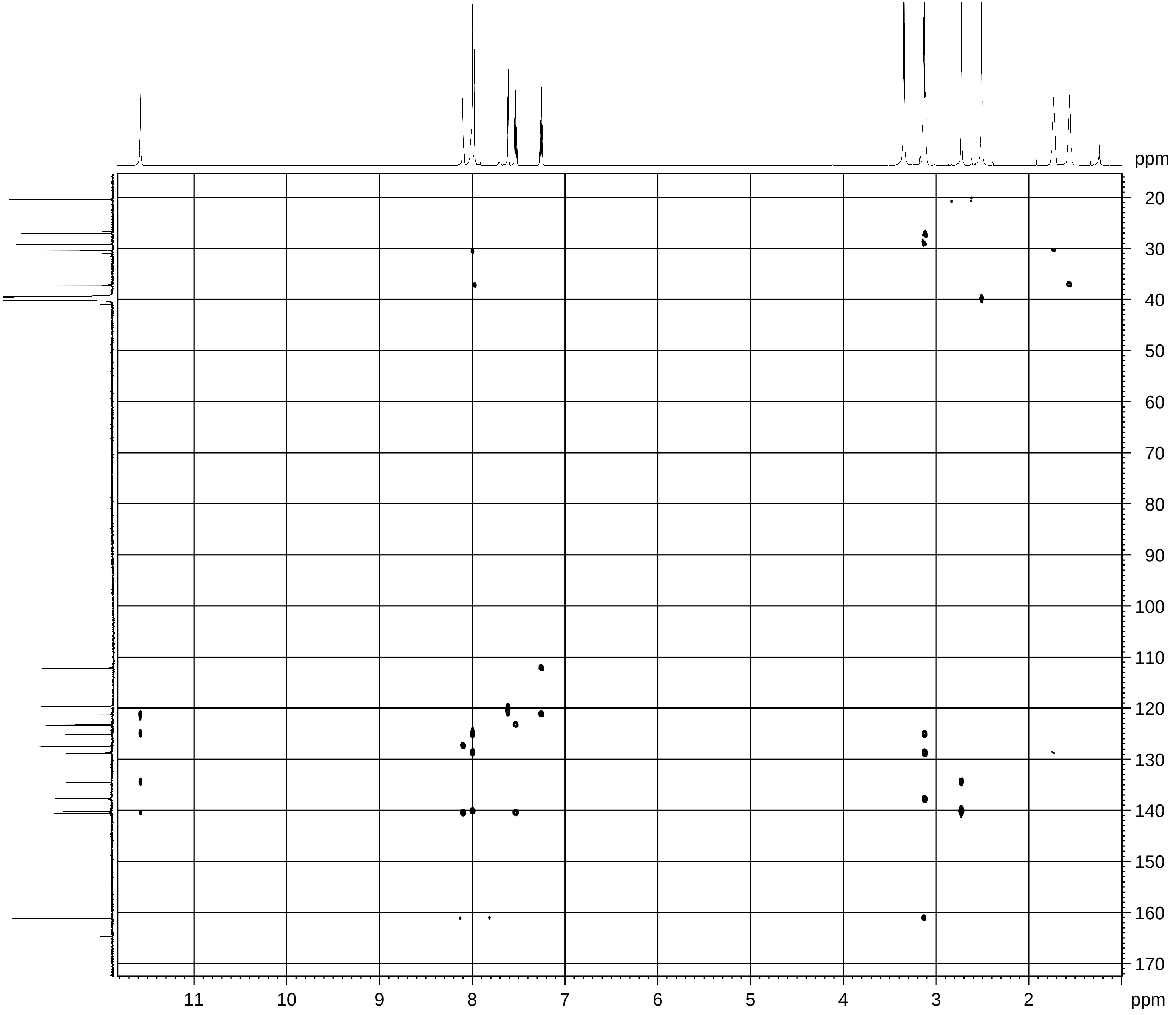

HMBC (8Hz, 140Hz)
143759
BAB0345_1
Batizi Benedek
2024.10.25. (KP)

```
Current Data Parameters
NAME              143759
EXPNO                 15
PROCNO                 1

F2 - Acquisition Parameters
Date_           20241026
Time              0.36 h
INSTRUM            spect
PROBHD   Z145856_0002 (
PULPROG    hmbcgplpndqf
TD                  2048
SOLVENT             DMSO
NS                     8
DS                    16
SWH             7812.500 Hz
FIDRES          7.629395 Hz
AQ             0.1310720 sec
RG                196.07
DW                64.000 usec
DE                 25.00 usec
TE                 295.0 K
CNST2        140.0000000
CNST13         8.0000000
D0            0.00000300 sec
D1            1.50000000 sec
D2            0.00357143 sec
D6            0.06250000 sec
D16           0.00020000 sec
IN0           0.00001510 sec
TDav                   1
SFO1        600.0037800 MHz
NUC1                  1H
P1                 11.50 usec
P2                 23.00 usec
PLW1         28.00000000 W
SFO2        150.8867157 MHz
NUC2                 13C
P3                  9.90 usec
PLW2         71.00000000 W
GPNAM[1]      SMSQ10.100
GPZ1               50.00 %
GPNAM[2]      SMSQ10.100
GPZ2               30.00 %
GPNAM[3]      SMSQ10.100
GPZ3               40.10 %
P16              1000.00 usec

F1 - Acquisition parameters
TD                   256
SFO1            150.8867 MHz
FIDRES        258.692047 Hz
SW               219.453 ppm
FnMODE                QF

F2 - Processing parameters
SI                  2048
SF           600.0000025 MHz
WDW                 SINE
SSB       0
LB        0 Hz
GB        0
PC                  1.40

F1 - Processing parameters
SI                  1024
MC2                   QF
SF           150.8701601 MHz
WDW                 SINE
SSB       0
LB        0 Hz
GB        0
```

27

Batizi Benedek	KP
Film	2023.11.27.

BRUKER Alpha
Resolution: 2 cm-1
Number of Scans: 16

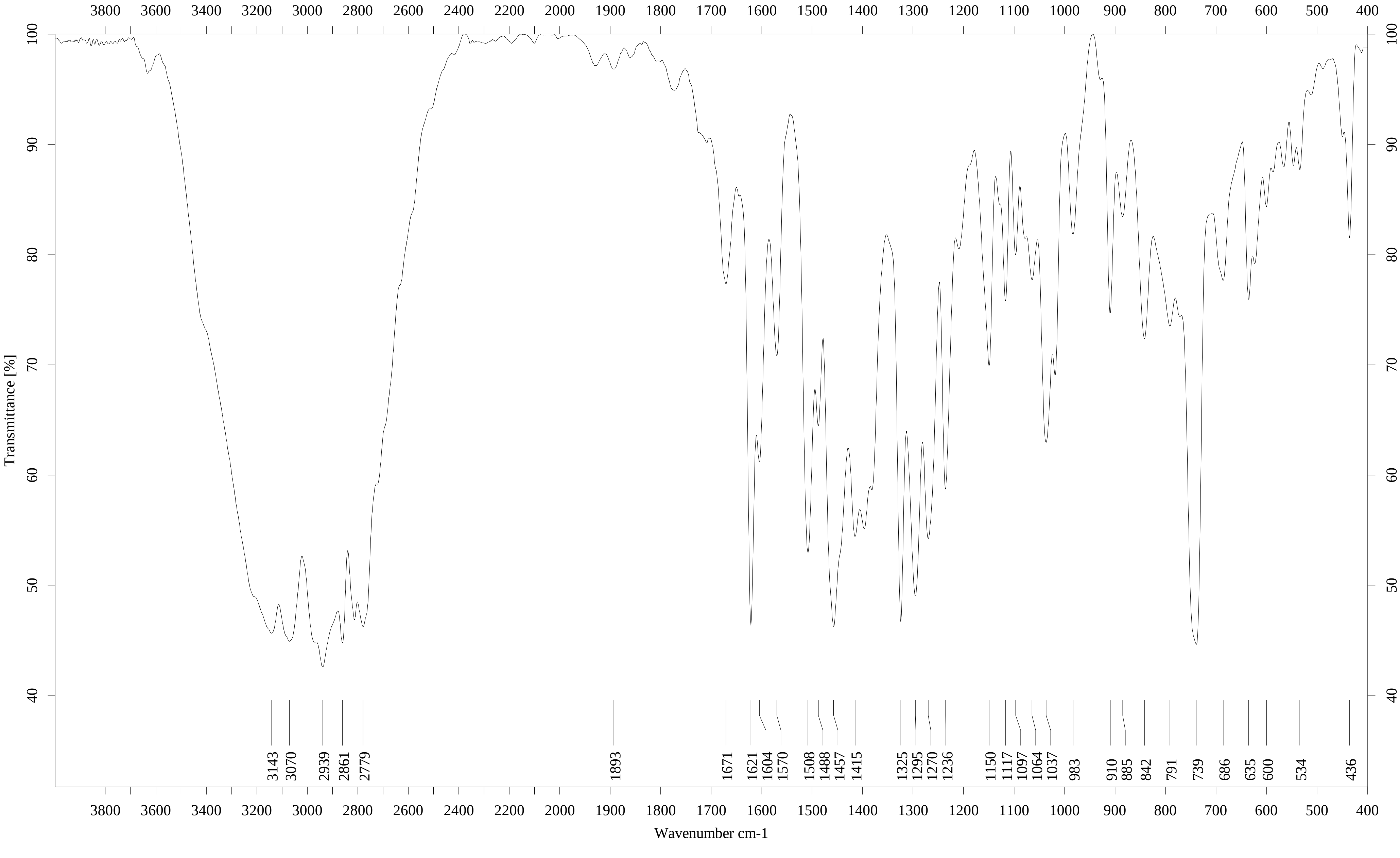

27

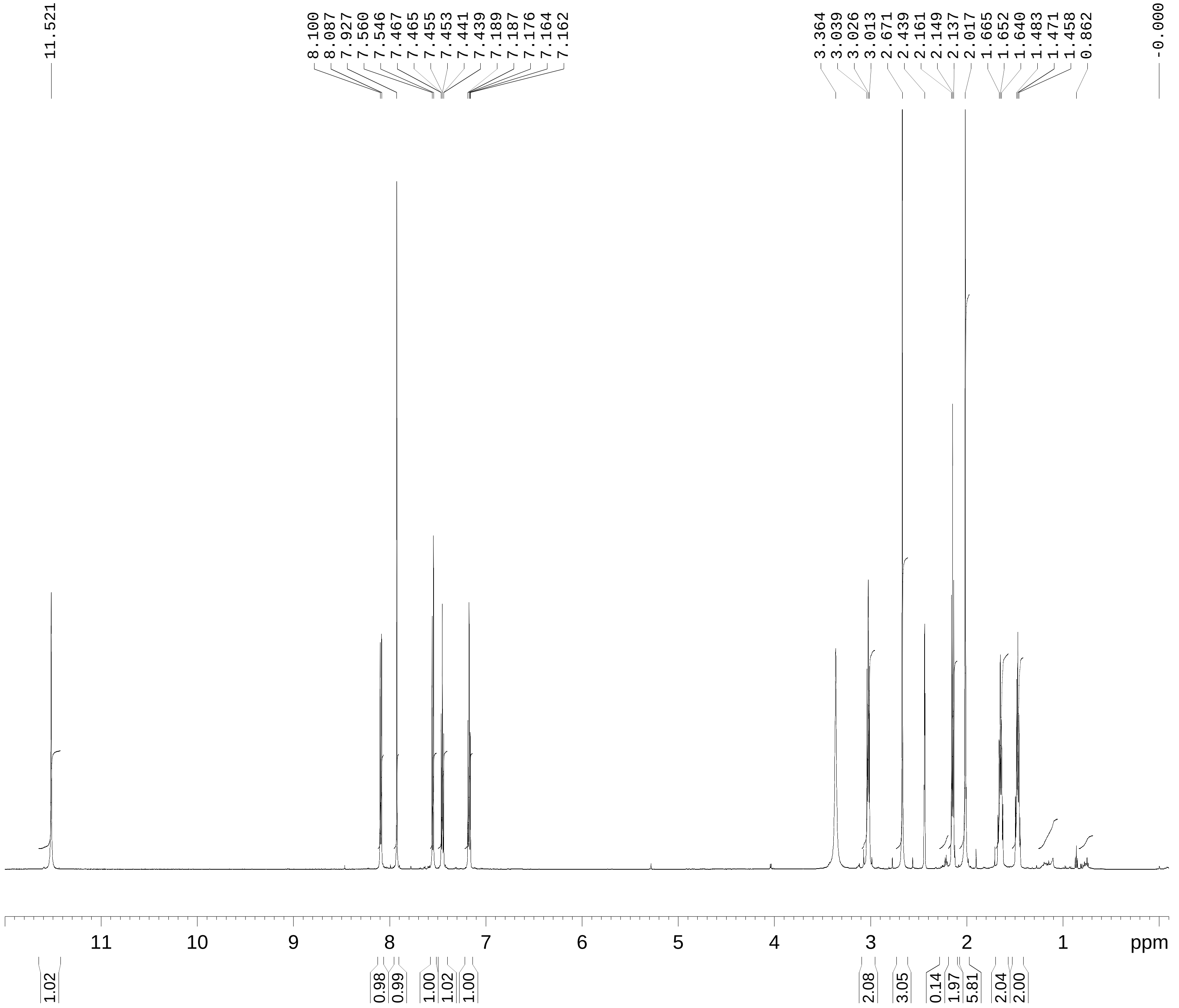

Standard 1H
142221
BAB0113_1
Batizi Benedek
2023.11.27. (KP)

Current Data Parameters
NAME 142221
EXPNO 11
PROCNO 1

F2 - Acquisition Parameters
Date_ 20231127
Time 16.11 h
INSTRUM spect
PROBHD Z145856_0002 (
PULPROG zg30
TD 65536
SOLVENT DMSO
NS 16
DS 2
SWH 12019.230 Hz
FIDRES 0.366798 Hz
AQ 2.7262976 sec
RG 56.03
DW 41.600 usec
DE 25.00 usec
TE 295.0 K
D1 1.00000000 sec
TD0 1
SFO1 600.0037050 MHz
NUC1 1H
P1 11.50 usec
PLW1 28.00000000 W

F2 - Processing parameters
SI 65536
SF 600.0000420 MHz
WDW EM
SSB 0
LB 0.30 Hz
GB 0
PC 1.00

27

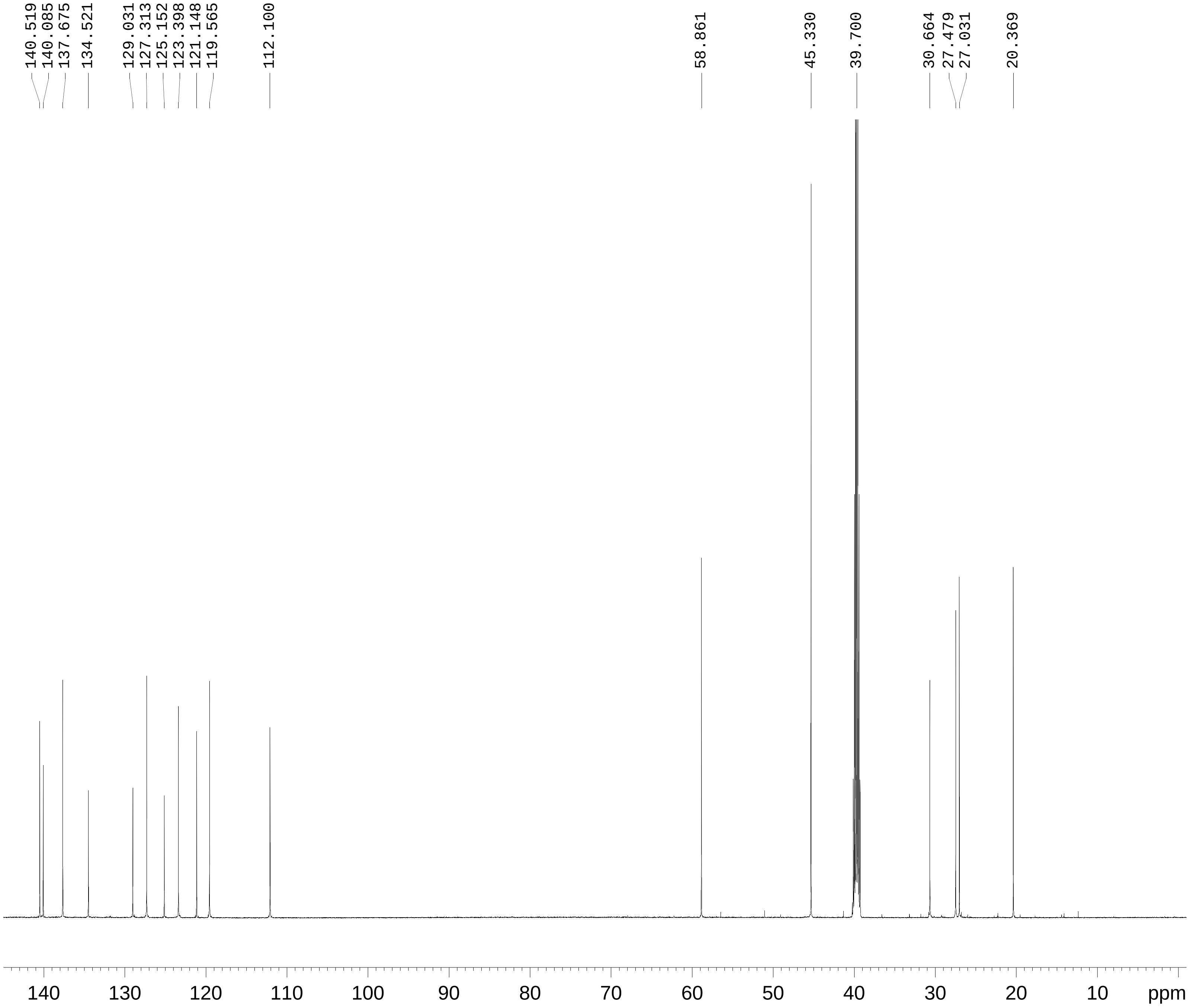

Standard 13C
142221
BAB0113_1
Batizi Benedek
2023.11.27. (KP)

Current Data Parameters

NAME	142221
EXPNO	12
PROCNO	1

F2 - Acquisition Parameters

Date_	20231127
Time	18.07 h
INSTRUM	spect
PROBHD	Z145856_0002 (
PULPROG	zgpg30
TD	65536
SOLVENT	DMSO
NS	2048
DS	4
SWH	36231.883 Hz
FIDRES	1.105709 Hz
AQ	0.9043968 sec
RG	196.07
DW	13.800 usec
DE	18.00 usec
TE	295.0 K
D1	1.00000000 sec
D11	0.03000000 sec
TD0	1
SFO1	150.8852070 MHz
NUC1	13C
P1	9.90 usec
PLW1	80.09999847 W
SFO2	600.0024000 MHz
NUC2	1H
CPDPRG[2	waltz16
PCPD2	80.00 usec
PLW2	35.00000000 W
PLW12	0.74861997 W
PLW13	0.37595001 W

F2 - Processing parameters

SI	32768
SF	150.8701586 MHz
WDW	EM
SSB	0
LB	1.00 Hz
GB	0
PC	1.40

27

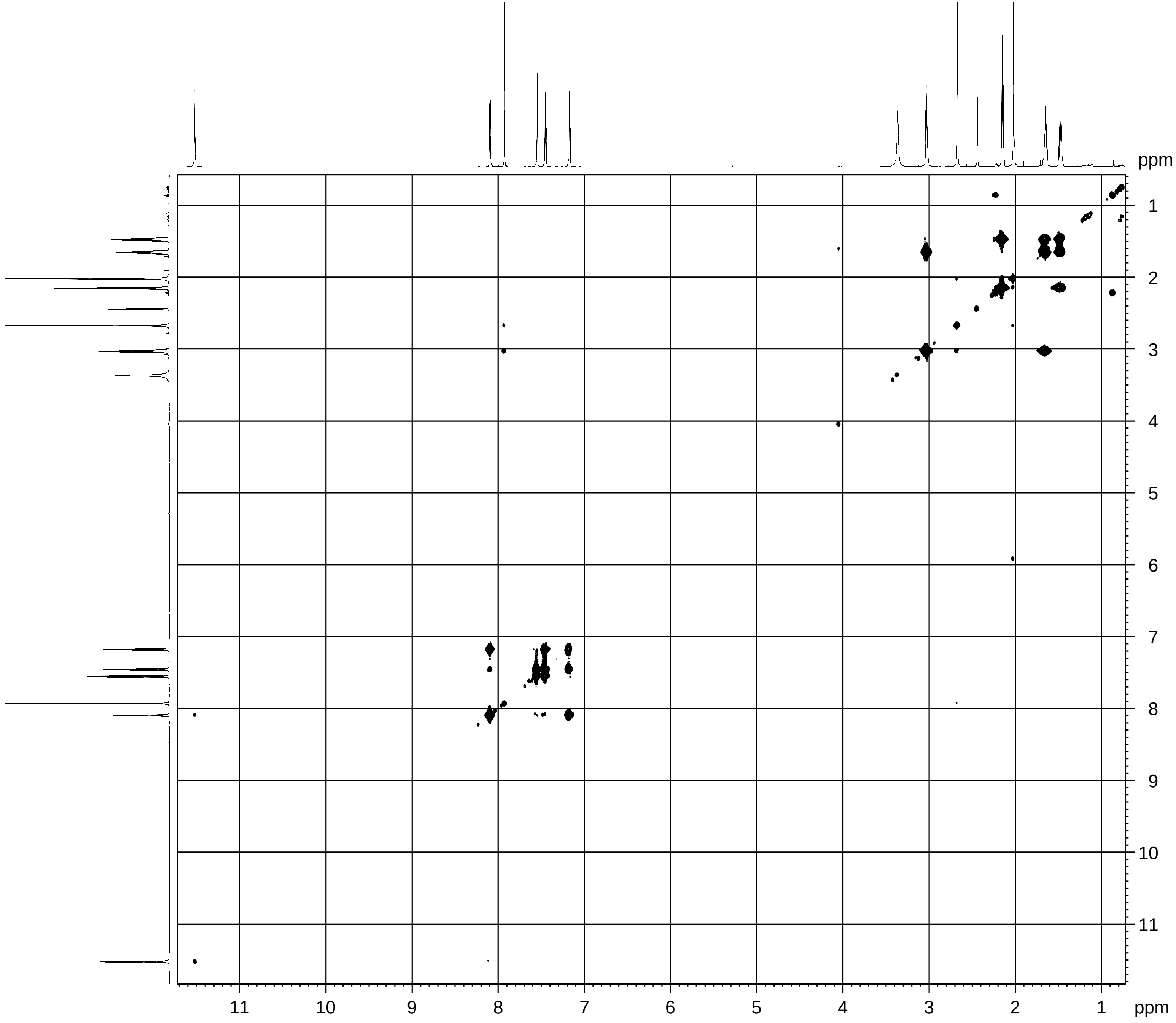

COSY
142221
BAB0113_1
Batizi Benedek
2023.11.27. (KP)

```
Current Data Parameters
NAME              142221
EXPNO                 13
PROCNO                 1

F2 - Acquisition Parameters
Date_           20231127
Time               18.08 h
INSTRUM            spect
PROBHD   Z145856_0002 (
PULPROG      cosygpmfqf
TD                  2048
SOLVENT             DMSO
NS                     2
DS                    16
SWH             7812.500 Hz
FIDRES          7.629395 Hz
AQ             0.1310720 sec
RG                196.07
DW                64.000 usec
DE                 25.00 usec
TE                 295.0 K
D0            0.00000300 sec
D1            2.00000000 sec
D13           0.00000400 sec
D16           0.00020000 sec
IN0           0.00012800 sec
TDav                   1
SFO1        600.0036000 MHz
NUC1                  1H
P1                 11.50 usec
PLW1         28.00000000 W
GPNAM[1]     SMSQ10.100
GPZ1               16.00 %
GPNAM[2]     SMSQ10.100
GPZ2               12.00 %
GPNAM[3]     SMSQ10.100
GPZ3               40.00 %
P16              1000.00 usec

F1 - Acquisition parameters
TD                   256
SFO1         600.0036 MHz
FIDRES      61.035156 Hz
SW             13.021 ppm
FnMODE                QF

F2 - Processing parameters
SI                  1024
SF           600.0000420 MHz
WDW                 SINE
SSB      0
LB       0 Hz
GB       0
PC                  1.40

F1 - Processing parameters
SI                  1024
MC2                   QF
SF           600.0000420 MHz
WDW                 SINE
SSB      0
LB       0 Hz
GB       0
```

27

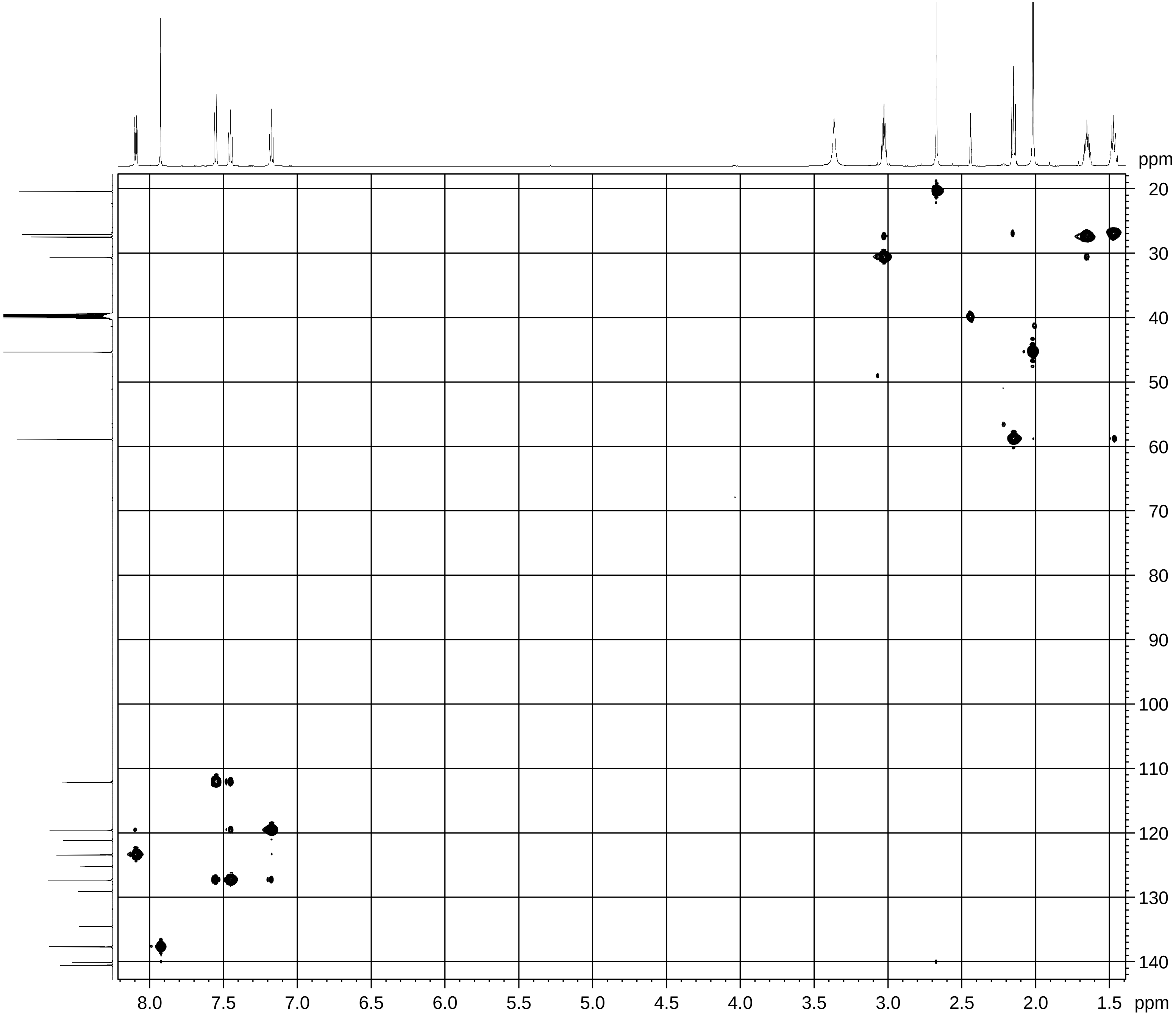

HSQC (140Hz)
142221
BAB0113_1
Batizi Benedek
2023.11.27. (KP)

```
Current Data Parameters
NAME             142221
EXPNO                14
PROCNO                1

F2 - Acquisition Parameters
Date_          20231127
Time              18.29 h
INSTRUM           spect
PROBHD   Z145856_0002 (
PULPROG  hsqcetgpsisp2.2
TD                 2048
SOLVENT            DMSO
NS                    4
DS                   32
SWH            7812.500 Hz
FIDRES         7.629395 Hz
AQ            0.1310720 sec
RG               196.07
DW               64.000 usec
DE                25.00 usec
TE                295.0 K
CNST2       140.0000000
CNST17       -0.5000000
D0           0.00000300 sec
D1           1.50000000 sec
D4           0.00178571 sec
D11          0.03000000 sec
D16          0.00020000 sec
D24          0.00089000 sec
IN0          0.00001510 sec
TDav                  1
SFO1        600.0036000 MHz
NUC1                 1H
P1                11.50 usec
P2                23.00 usec
P28      0 usec
PLW1        28.00000000 W
SFO2        150.8867157 MHz
NUC2                13C
CPDPRG[2 bi_p5m4sp_4sp.2
P3                 9.90 usec
P14              500.00 usec
P24             2000.00 usec
P63             1500.00 usec
PLW0     0 W
PLW2        80.09999847 W
PLW12        2.59520006 W
SPNAM[3] Crp60,0.5,20.1
SPOAL3            0.500
SPOFFS3  0 Hz
SPW3        11.99499989 W
SPNAM[7]    Crp60comp.4
SPOAL7            0.500
SPOFFS7  0 Hz
SPW7        11.99499989 W
SPNAM[14 Crp42,1.5,20.2
SPOAL14           0.500
SPOFFS14 0 Hz
SPW14        6.71710014 W
SPNAM[31 Crp42,1.5,20.2
SPOAL31           0.500
SPOFFS31 0 Hz
SPW31        1.67929995 W
GPNAM[1]     SMSQ10.100
GPZ1              80.00 %
GPNAM[2]     SMSQ10.100
GPZ2              20.10 %
GPNAM[3]     SMSQ10.100
GPZ3              11.00 %
GPNAM[4]     SMSQ10.100
GPZ4              -5.00 %
P16             1000.00 usec
P19              600.00 usec

F1 - Acquisition parameters
TD                  256
SFO1           150.8867 MHz
FIDRES       258.692047 Hz
SW              219.453 ppm
FnMODE    Echo-Antiecho

F2 - Processing parameters
SI                 1024
SF          600.0000420 MHz
WDW               QSINE
```

27

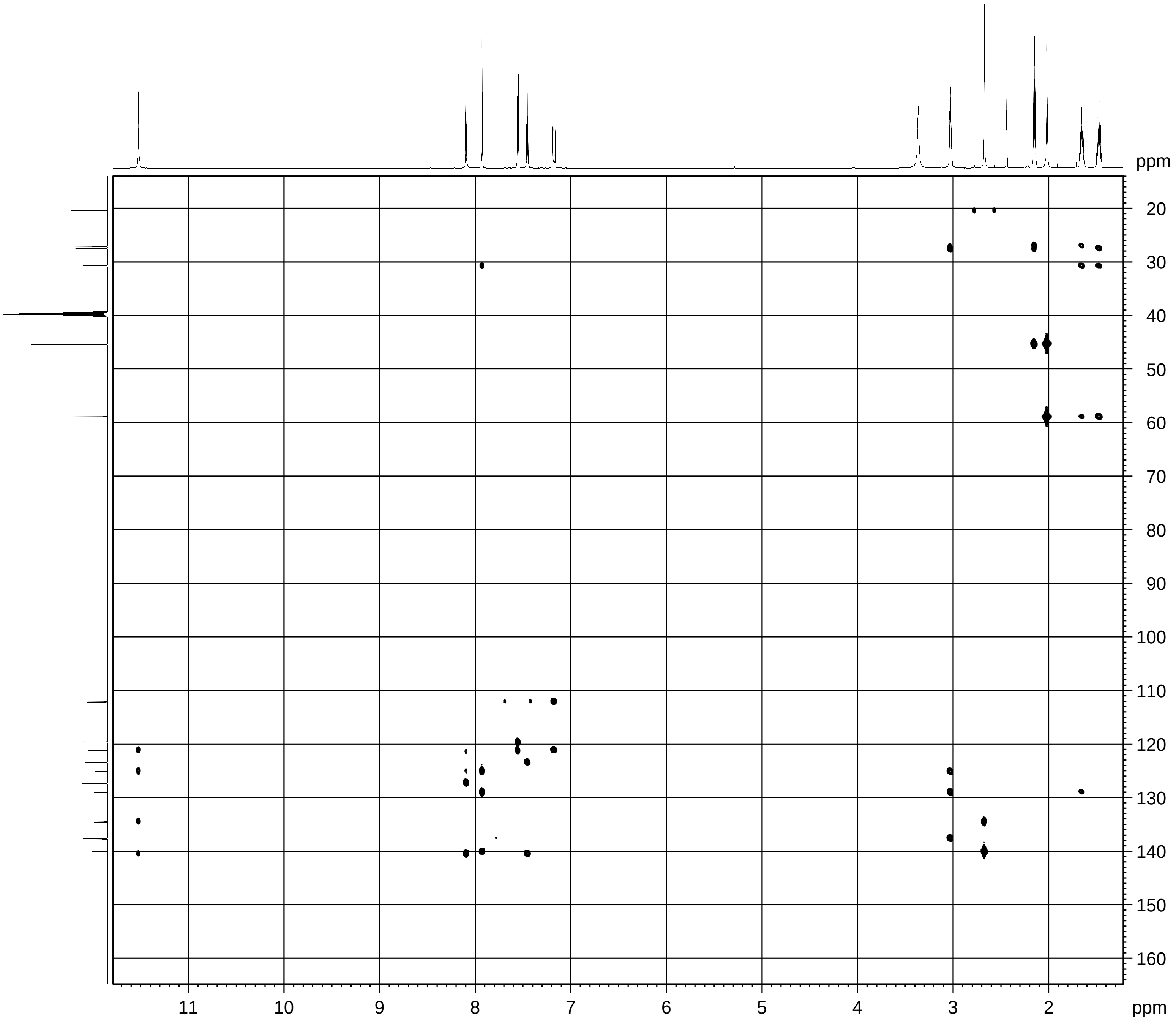

HMBC (8Hz, 140Hz)
142221
BAB0113_1
Batizi Benedek
2023.11.27. (KP)

```
Current Data Parameters
NAME              142221
EXPNO                 15
PROCNO                 1

F2 - Acquisition Parameters
Date_           20231127
Time               18.58 h
INSTRUM            spect
PROBHD    Z145856_0002 (
PULPROG    hmbcgplpndqf
TD                  2048
SOLVENT             DMSO
NS                     4
DS                    16
SWH             7812.500 Hz
FIDRES          7.629395 Hz
AQ             0.1310720 sec
RG                196.07
DW                64.000 usec
DE                 25.00 usec
TE                 295.0 K
CNST2        140.0000000
CNST13         8.0000000
D0            0.00000300 sec
D1            1.50000000 sec
D2            0.00357143 sec
D6            0.06250000 sec
D16           0.00020000 sec
IN0           0.00001510 sec
TDav                   1
SFO1         600.0037800 MHz
NUC1                  1H
P1                 11.50 usec
P2                 23.00 usec
PLW1         28.00000000 W
SFO2         150.8867157 MHz
NUC2                 13C
P3                  9.90 usec
PLW2         80.09999847 W
GPNAM[1]      SMSQ10.100
GPZ1               50.00 %
GPNAM[2]      SMSQ10.100
GPZ2               30.00 %
GPNAM[3]      SMSQ10.100
GPZ3               40.10 %
P16              1000.00 usec

F1 - Acquisition parameters
TD                   256
SFO1            150.8867 MHz
FIDRES        258.692047 Hz
SW               219.453 ppm
FnMODE                QF

F2 - Processing parameters
SI                  2048
SF           600.0000420 MHz
WDW                 SINE
SSB      0
LB       0 Hz
GB       0
PC                  1.40

F1 - Processing parameters
SI                  1024
MC2                   QF
SF           150.8701586 MHz
WDW                 SINE
SSB      0
LB       0 Hz
GB       0
```

27

NHMBC
142221
BAB0113_1
Batizi Benedek
2023.11.27. (KP)

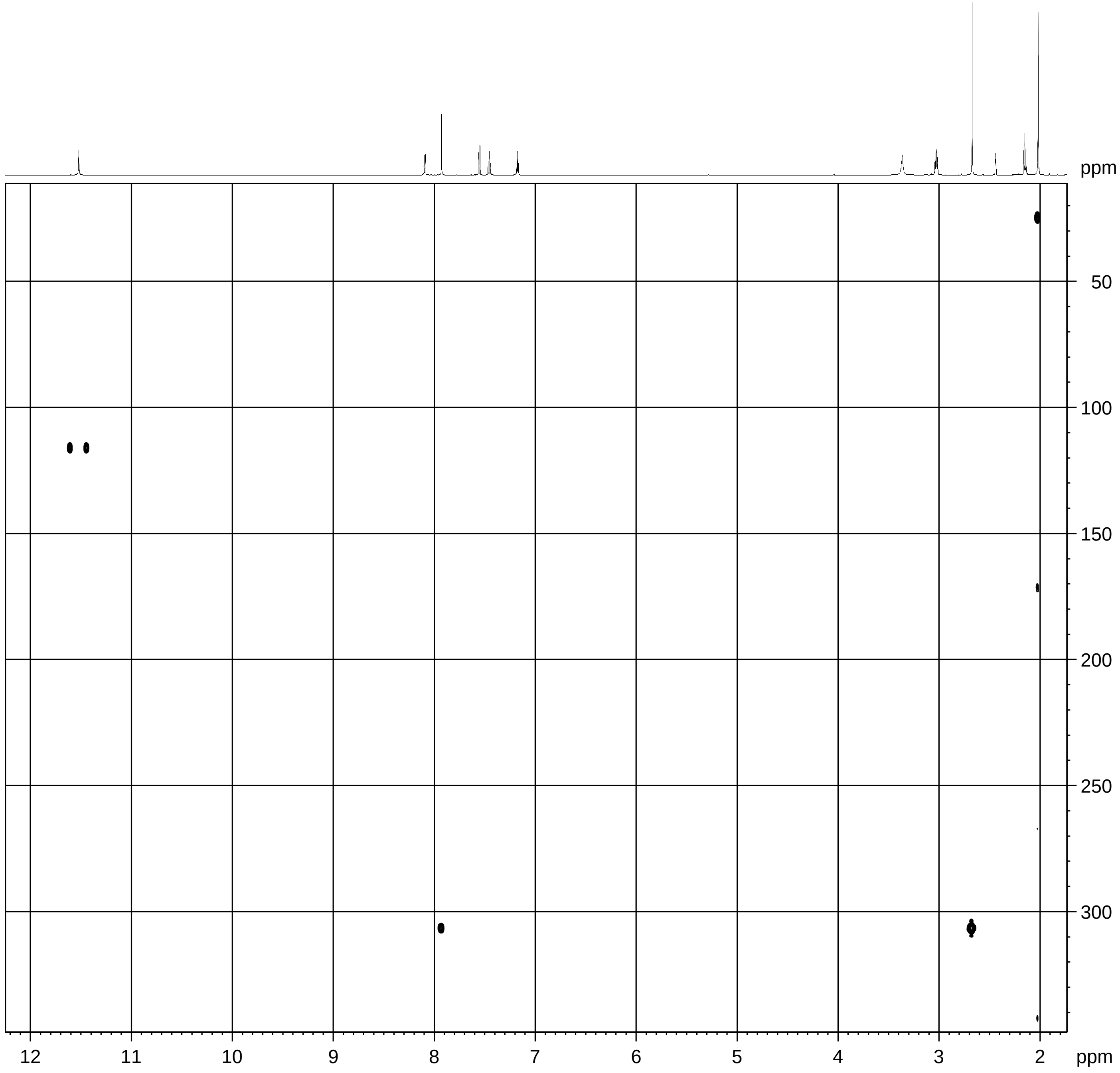


```
Current Data Parameters
NAME              142221
EXPNO                 16
PROCNO                 1

F2 - Acquisition Parameters
Date_           20231127
Time               19.31 h
INSTRUM            spect
PROBHD   Z145856_0002 (
PULPROG       hmbcgpndqf
TD                  2048
SOLVENT             DMSO
NS                    16
DS                    16
SWH             9615.385 Hz
FIDRES          9.390024 Hz
AQ             0.1064960 sec
RG                196.07
DW                52.000 usec
DE                 25.00 usec
TE                 295.0 K
CNST13         5.0000000
D0            0.00000300 sec
D1            2.00000000 sec
D6            0.10000000 sec
D16           0.00020000 sec
IN0           0.00002060 sec
TDav                   1
SFO1         600.0045600 MHz
NUC1                  1H
P1                 11.50 usec
P2                 23.00 usec
PLW1         28.00000000 W
SFO2          60.8096315 MHz
NUC2                 15N
P3                 14.60 usec
PLW2        236.00000000 W
GPNAM[1]      SMSQ10.100
GPZ1               70.00 %
GPNAM[2]      SMSQ10.100
GPZ2               30.00 %
GPNAM[3]      SMSQ10.100
GPZ3               50.10 %
P16              1000.00 usec

F1 - Acquisition parameters
TD                   128
SFO1            60.80963 MHz
FIDRES        379.247559 Hz
SW               399.145 ppm
FnMODE                QF

F2 - Processing parameters
SI                  2048
SF           600.0000420 MHz
WDW                 SINE
SSB      0
LB       0 Hz
GB       0
PC                  1.40

F1 - Processing parameters
SI                  1024
MC2                   QF
SF            60.7974720 MHz
WDW                 SINE
SSB      0
LB       0 Hz
GB       0
```

28

Pollak Patrik	KP
KBr	1/3/2024

BRUKER Alpha
Resolution: 2 cm-1
Number of Scans: 16

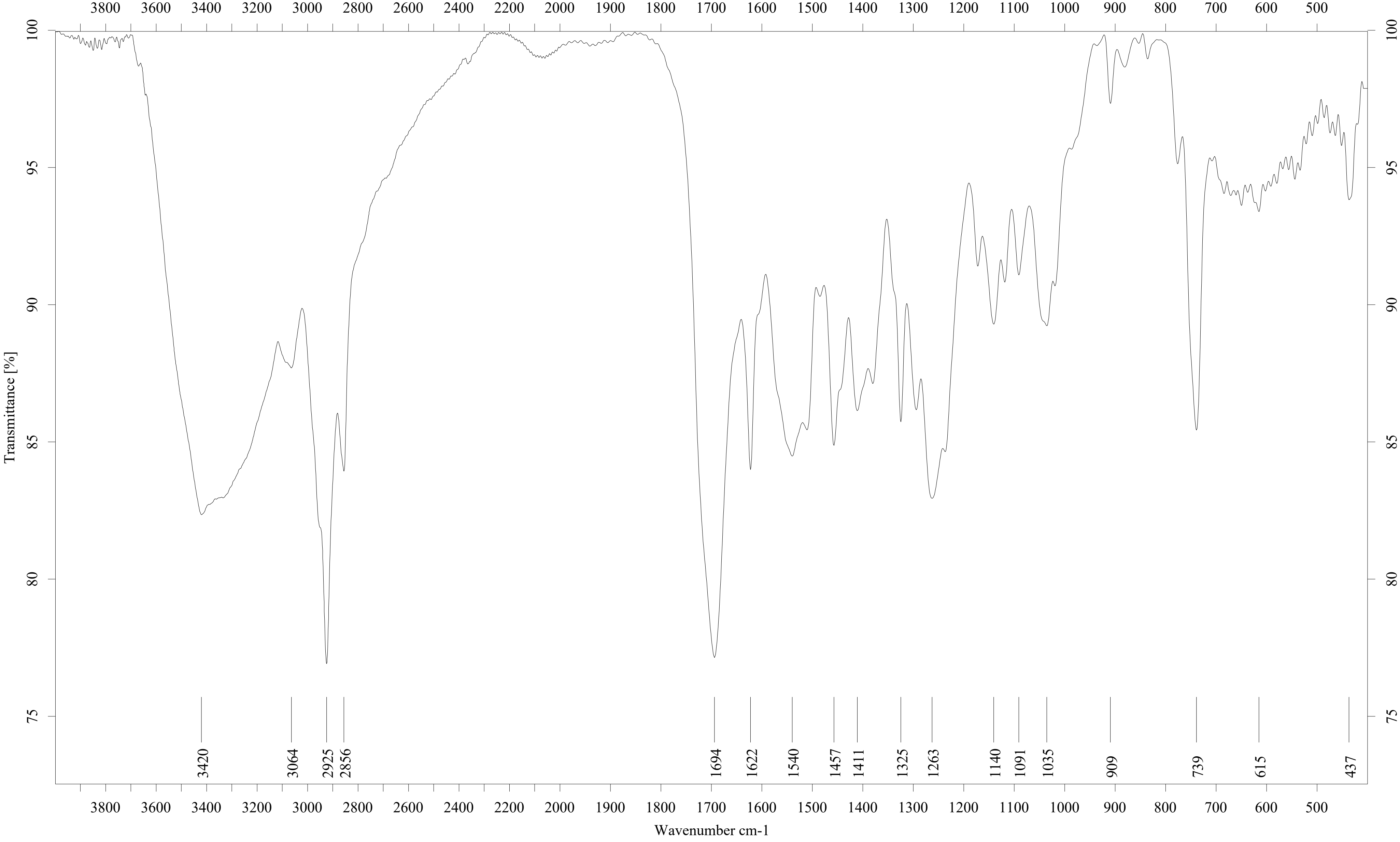

S60

28

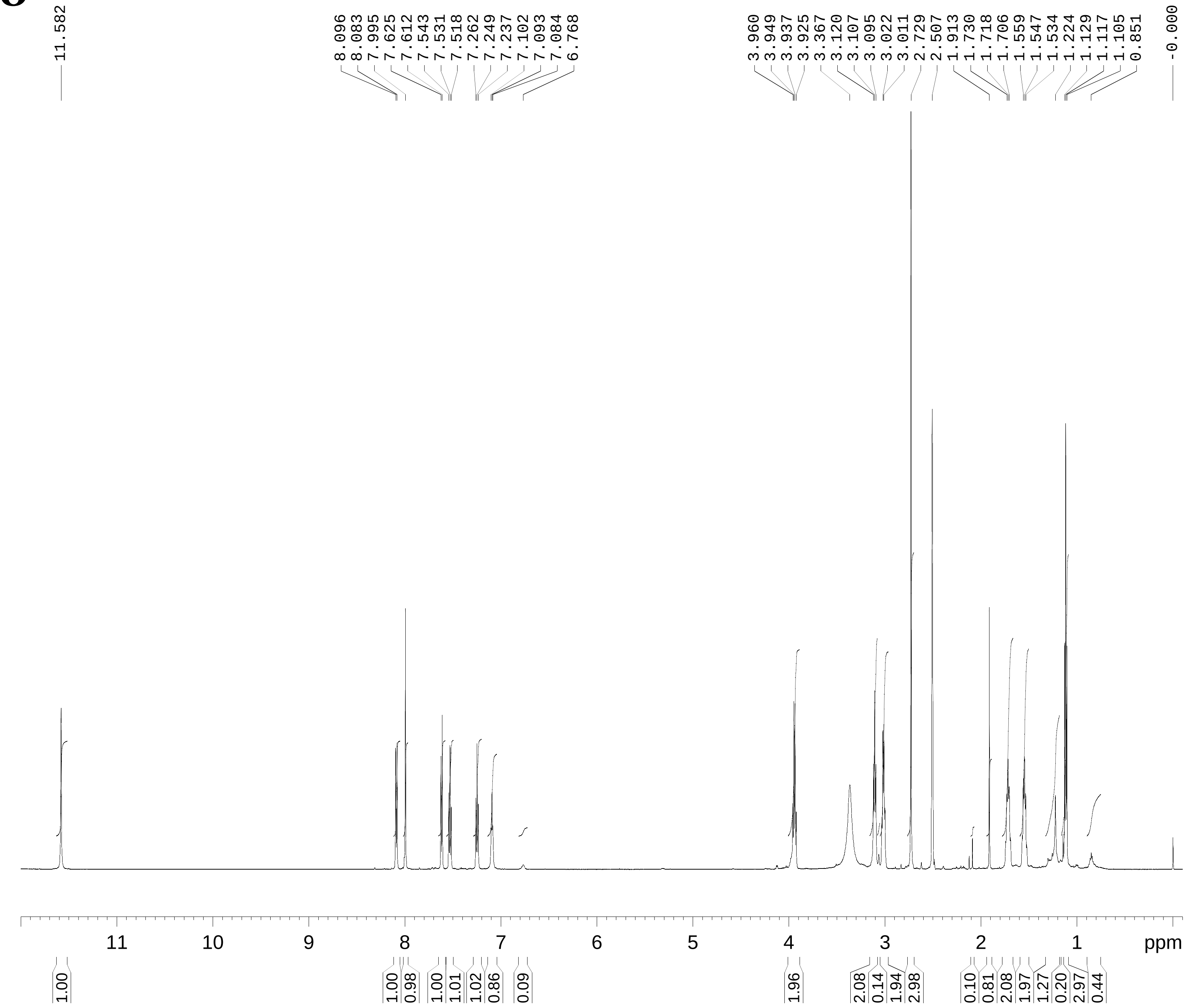

Standard 1H
142318
BAB0122_1
Pollak Patrik
2024.01.03. (KP)

Current Data Parameters
NAME 142318
EXPNO 11
PROCNO 1

F2 - Acquisition Parameters
Date_ 20240103
Time 19.41 h
INSTRUM spect
PROBHD Z145856_0002 (
PULPROG zg30
TD 65536
SOLVENT DMSO
NS 16
DS 2
SWH 12019.230 Hz
FIDRES 0.366798 Hz
AQ 2.7262976 sec
RG 196.07
DW 41.600 usec
DE 25.00 usec
TE 295.0 K
D1 1.00000000 sec
TD0 1
SFO1 600.0037050 MHz
NUC1 1H
P1 11.50 usec
PLW1 28.00000000 W

F2 - Processing parameters
SI 65536
SF 600.0000022 MHz
WDW EM
SSB 0
LB 0.30 Hz
GB 0
PC 1.00

28

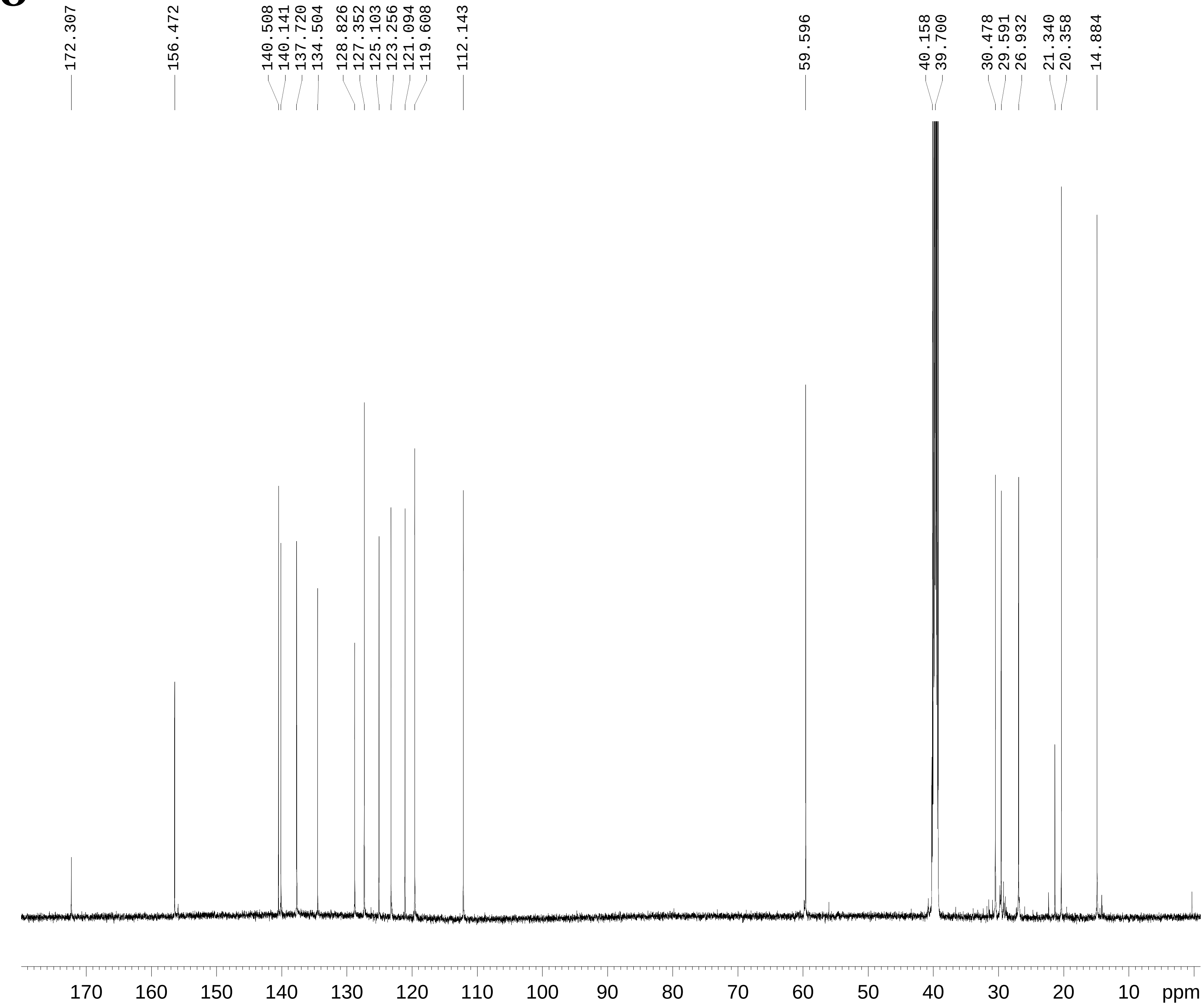

Standard 13C
142318
BAB0122_1
Pollak Patrik
2024.01.03. (KP)

Current Data Parameters

NAME	142318
EXPNO	12
PROCNO	1

F2 - Acquisition Parameters

Date_	20240103
Time	20.49 h
INSTRUM	spect
PROBHD	Z145856_0002 (
PULPROG	zgpg30
TD	65536
SOLVENT	DMSO
NS	2048
DS	4
SWH	36231.883 Hz
FIDRES	1.105709 Hz
AQ	0.9043968 sec
RG	196.07
DW	13.800 usec
DE	18.00 usec
TE	295.0 K
D1	1.00000000 sec
D11	0.03000000 sec
TD0	1
SFO1	150.8852070 MHz
NUC1	13C
P1	9.90 usec
PLW1	80.09999847 W
SFO2	600.0024000 MHz
NUC2	1H
CPDPRG[2	waltz16
PCPD2	80.00 usec
PLW2	35.00000000 W
PLW12	0.74861997 W
PLW13	0.37595001 W

F2 - Processing parameters

SI	32768
SF	150.8701591 MHz
WDW	EM
SSB	0
LB	1.00 Hz
GB	0
PC	1.40

28

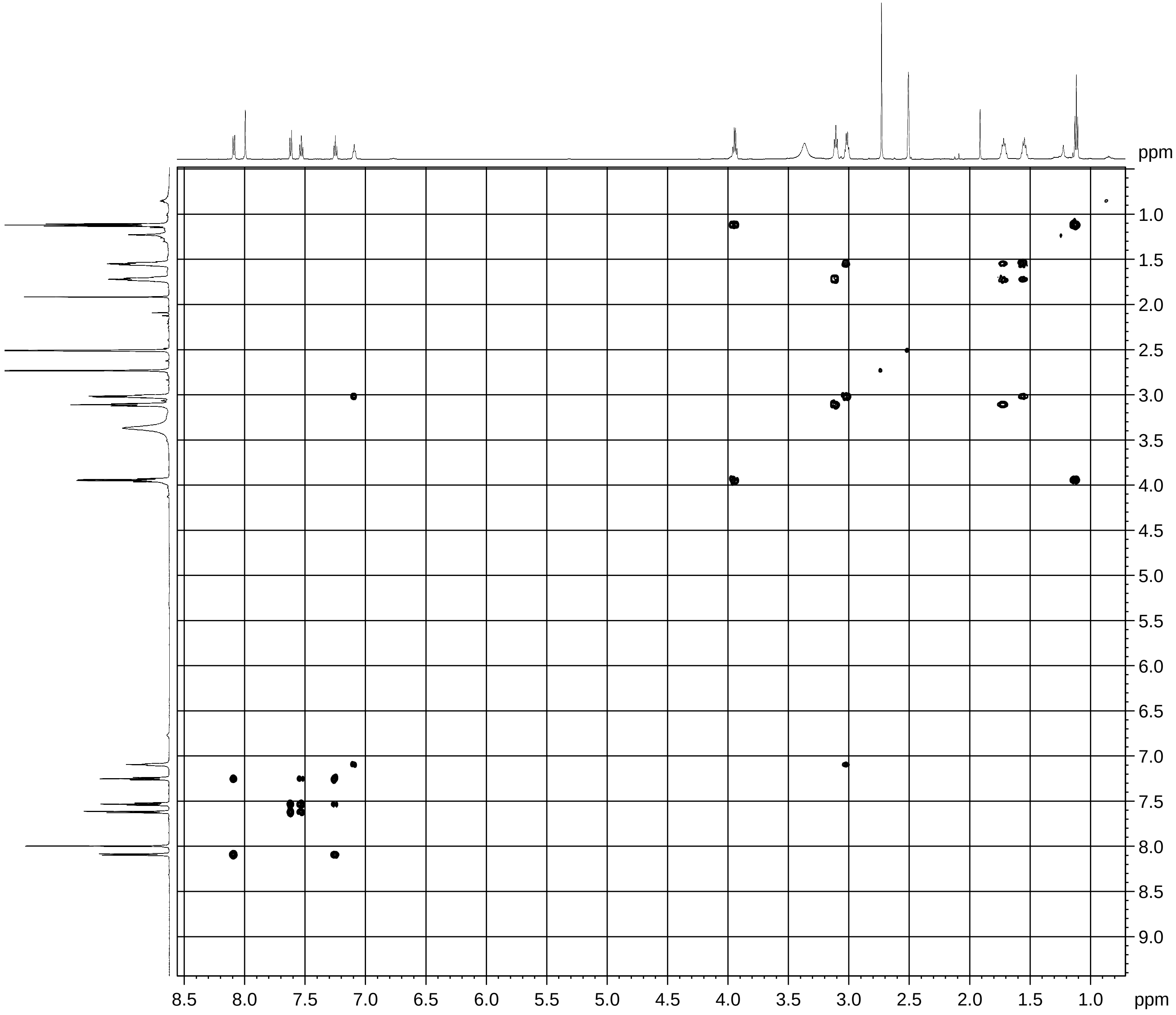

COSY
142318
BAB0122_1
Pollak Patrik
2024.01.03. (KP)

```
Current Data Parameters
NAME             142318
EXPNO                13
PROCNO                1

F2 - Acquisition Parameters
Date_          20240103
Time              20.51 h
INSTRUM           spect
PROBHD   Z145856_0002 (
PULPROG      cosygpmfqf
TD                 2048
SOLVENT            DMSO
NS                    2
DS                   16
SWH            7812.500 Hz
FIDRES         7.629395 Hz
AQ            0.1310720 sec
RG               196.07
DW               64.000 usec
DE                25.00 usec
TE                295.0 K
D0           0.00000300 sec
D1           2.00000000 sec
D13          0.00000400 sec
D16          0.00020000 sec
IN0          0.00012800 sec
TDav                  1
SFO1        600.0036000 MHz
NUC1                 1H
P1                11.50 usec
PLW1        28.00000000 W
GPNAM[1]     SMSQ10.100
GPZ1              16.00 %
GPNAM[2]     SMSQ10.100
GPZ2              12.00 %
GPNAM[3]     SMSQ10.100
GPZ3              40.00 %
P16             1000.00 usec

F1 - Acquisition parameters
TD                  256
SFO1           600.0036 MHz
FIDRES        61.035156 Hz
SW               13.021 ppm
FnMODE               QF

F2 - Processing parameters
SI                 1024
SF          600.0000022 MHz
WDW                SINE
SSB      0
LB       0 Hz
GB       0
PC                 1.40

F1 - Processing parameters
SI                 1024
MC2                  QF
SF          600.0000022 MHz
WDW                SINE
SSB      0
LB       0 Hz
GB       0
```

28

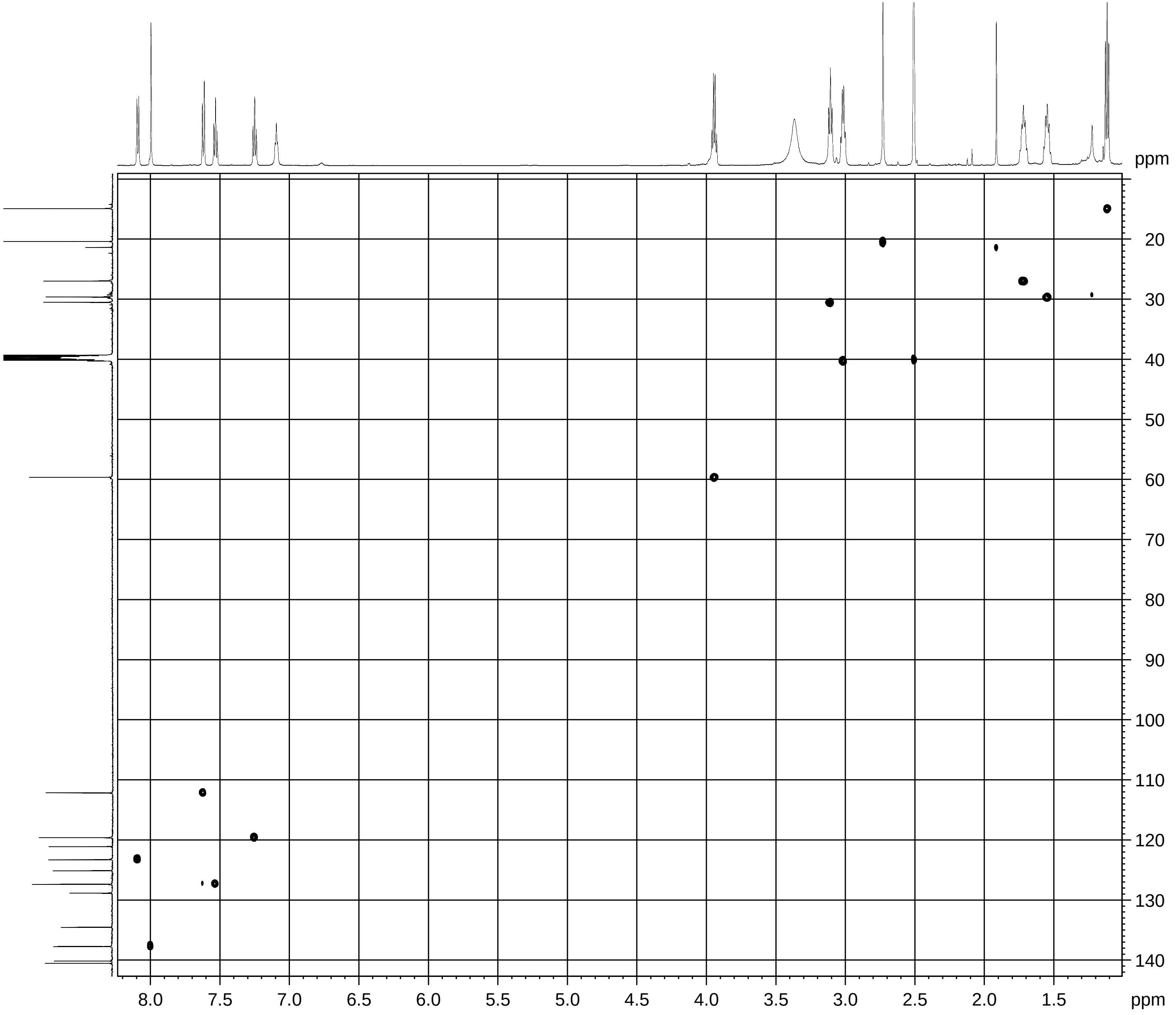

HSQC (140Hz)
142318
BAB0122_1
Pollak Patrik
2024.01.03. (KP)

Current Data Parameters
NAME 142318
EXPNO 14
PROCNO 1

F2 - Acquisition Parameters
Date_ 20240103
Time 21.11 h
INSTRUM spect
PROBHD Z145856_0002 (
PULPROG hsqcetgpsisp2.2
TD 2048
SOLVENT DMSO
NS 4
DS 32
SWH 7812.500 Hz
FIDRES 7.629395 Hz
AQ 0.1310720 sec
RG 196.07
DW 64.000 usec
DE 25.00 usec
TE 295.0 K
CNST2 140.0000000
CNST17 -0.5000000
D0 0.00000300 sec
D1 1.50000000 sec
D4 0.00178571 sec
D11 0.03000000 sec
D16 0.00020000 sec
D24 0.00089000 sec
IN0 0.00001510 sec
TDav 1
SFO1 600.0036000 MHz
NUC1 1H
P1 11.50 usec
P2 23.00 usec
P28 0 usec
PLW1 28.00000000 W
SFO2 150.8867157 MHz
NUC2 13C
CPDPRG[2 bi_p5m4sp_4sp.2
P3 9.90 usec
P14 500.00 usec
P24 2000.00 usec
P63 1500.00 usec
PLW0 0 W
PLW2 80.09999847 W
PLW12 2.59520006 W
SPNAM[3] Crp60,0.5,20.1
SPOAL3 0.500
SPOFFS3 0 Hz
SPW3 11.99499989 W
SPNAM[7] Crp60comp.4
SPOAL7 0.500
SPOFFS7 0 Hz
SPW7 11.99499989 W
SPNAM[14 Crp42,1.5,20.2
SPOAL14 0.500
SPOFFS14 0 Hz
SPW14 6.71710014 W
SPNAM[31 Crp42,1.5,20.2
SPOAL31 0.500
SPOFFS31 0 Hz
SPW31 1.67929995 W
GPNAM[1] SMSQ10.100
GPZ1 80.00 %
GPNAM[2] SMSQ10.100
GPZ2 20.10 %
GPNAM[3] SMSQ10.100
GPZ3 11.00 %
GPNAM[4] SMSQ10.100
GPZ4 -5.00 %
P16 1000.00 usec
P19 600.00 usec

F1 - Acquisition parameters
TD 256
SFO1 150.8867 MHz
FIDRES 258.692047 Hz
SW 219.453 ppm
FnMODE Echo-Antiecho

F2 - Processing parameters
SI 1024
SF 600.0000022 MHz
WDW QSINE

28

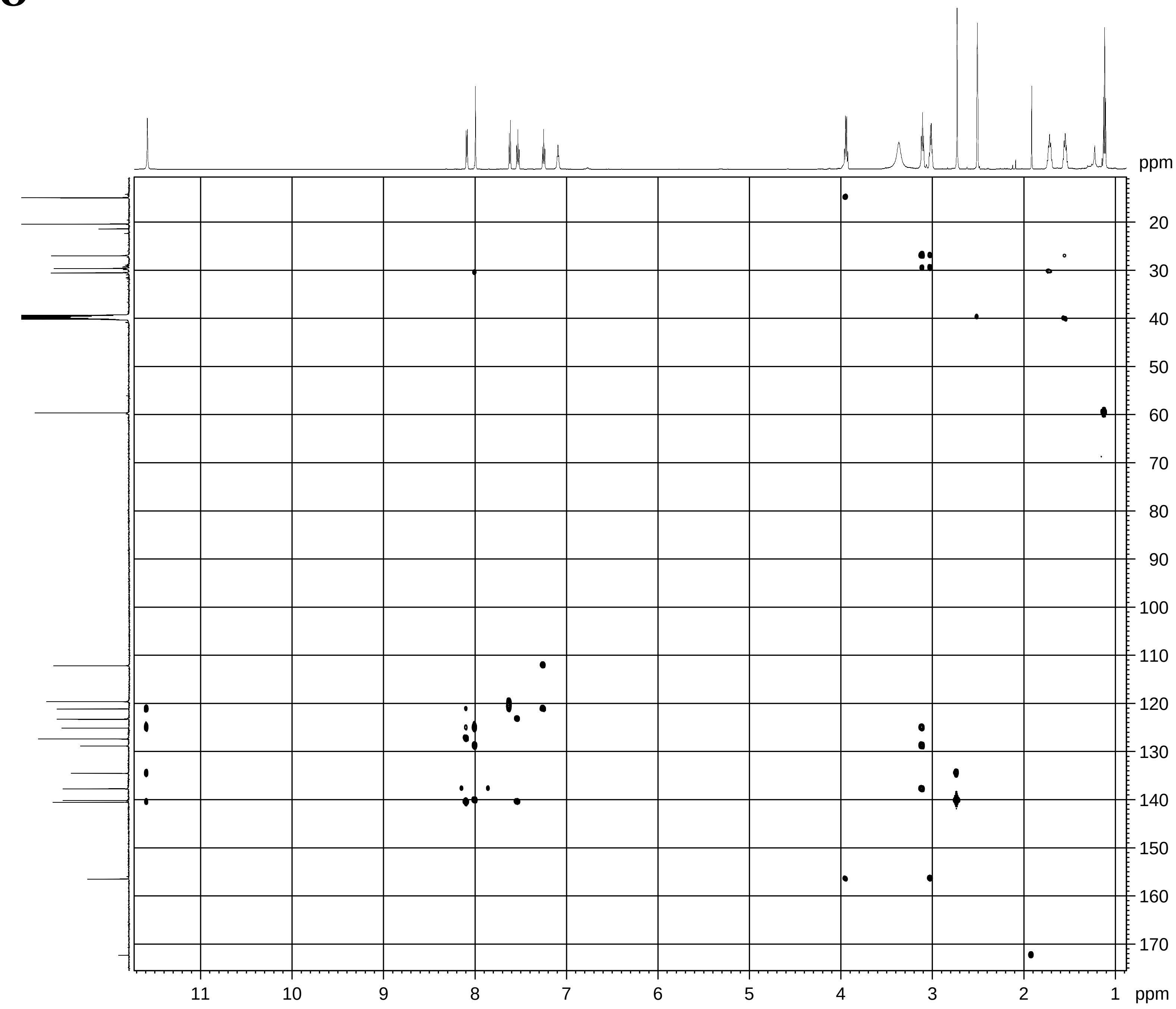

HMBC (8Hz, 140Hz)
142318
BAB0122_1
Pollak Patrik
2024.01.03. (KP)

```
Current Data Parameters
NAME              142318
EXPNO                 15
PROCNO                 1

F2 - Acquisition Parameters
Date_           20240103
Time               21.41 h
INSTRUM            spect
PROBHD   Z145856_0002 (
PULPROG    hmbcgplpndqf
TD                  2048
SOLVENT             DMSO
NS                     4
DS                    16
SWH             7812.500 Hz
FIDRES          7.629395 Hz
AQ             0.1310720 sec
RG                196.07
DW                64.000 usec
DE                 25.00 usec
TE                 295.0 K
CNST2        140.0000000
CNST13         8.0000000
D0           0.00000300 sec
D1           1.50000000 sec
D2           0.00357143 sec
D6           0.06250000 sec
D16          0.00020000 sec
IN0          0.00001510 sec
TDav                   1
SFO1        600.0037800 MHz
NUC1                  1H
P1                 11.50 usec
P2                 23.00 usec
PLW1         28.00000000 W
SFO2        150.8867157 MHz
NUC2                 13C
P3                  9.90 usec
PLW2         80.09999847 W
GPNAM[1]      SMSQ10.100
GPZ1               50.00 %
GPNAM[2]      SMSQ10.100
GPZ2               30.00 %
GPNAM[3]      SMSQ10.100
GPZ3               40.10 %
P16              1000.00 usec

F1 - Acquisition parameters
TD                   256
SFO1            150.8867 MHz
FIDRES        258.692047 Hz
SW               219.453 ppm
FnMODE                QF

F2 - Processing parameters
SI                  2048
SF           600.0000022 MHz
WDW                 SINE
SSB      0
LB       0 Hz
GB       0
PC                  1.40

F1 - Processing parameters
SI                  1024
MC2                   QF
SF           150.8701591 MHz
WDW                 SINE
SSB      0
LB       0 Hz
GB       0
```

28

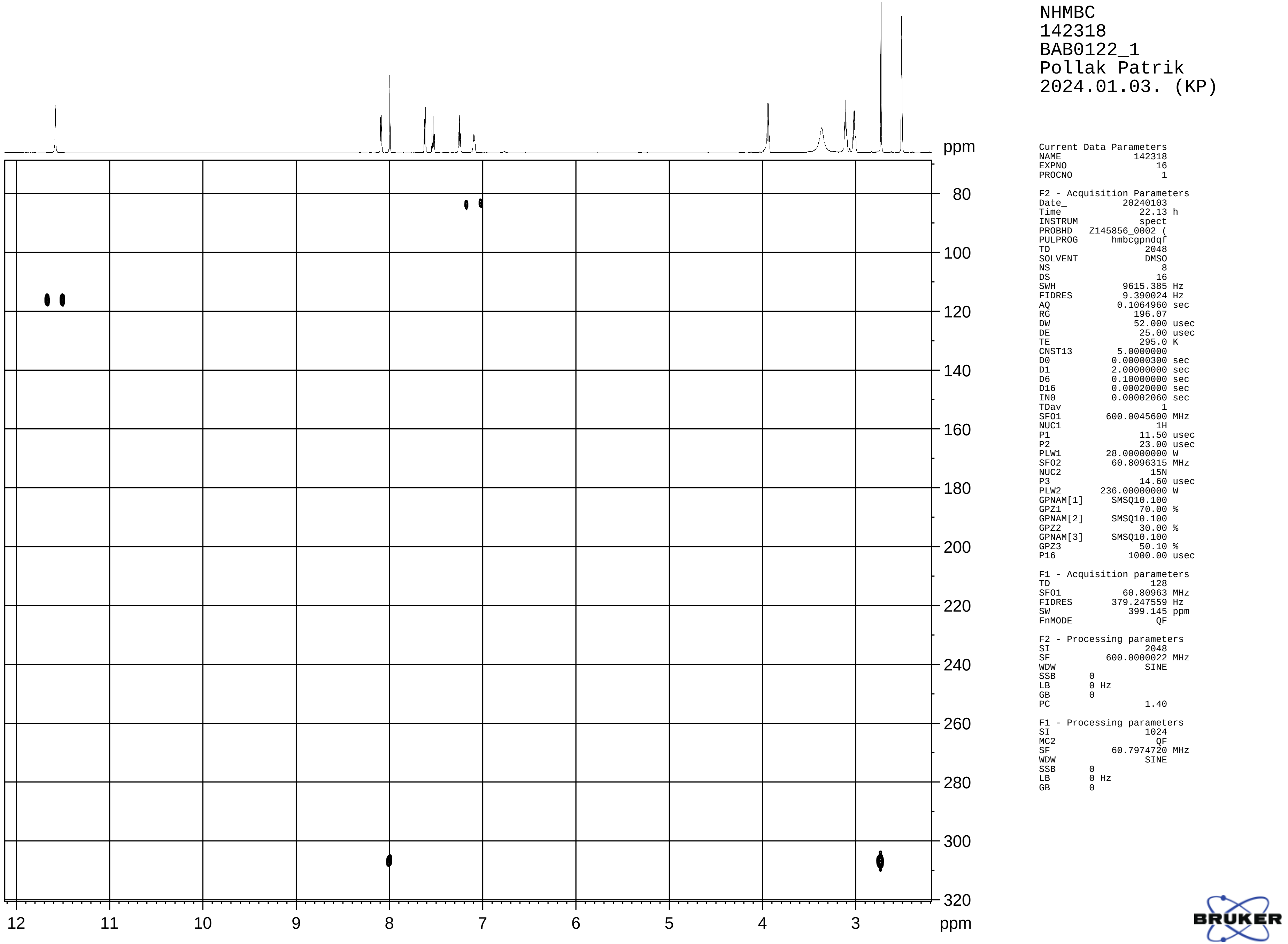
NHMBC
142318
BAB0122_1
Pollak Patrik
2024.01.03. (KP)
ppm
80
100
120
140
160
180
200
220
240
260
280
300
320
12
11
10
9
8
7
6
5
4
3
ppm
Current Data Parameters
NAME 142318
EXPNO 16
PROCNO 1
F2 - Acquisition Parameters
Date_ 20240103
Time 22.13 h
INSTRUM spect
PROBHD Z145856_0002 (
PULPROG hmbcgpndqf
TD 2048
SOLVENT DMSO
NS 8
DS 16
SWH 9615.385 Hz
FIDRES 9.390024 Hz
AQ 0.1064960 sec
RG 196.07
DW 52.000 usec
DE 25.00 usec
TE 295.0 K
CNST13 5.0000000
D0 0.00000300 sec
D1 2.00000000 sec
D6 0.10000000 sec
D16 0.00020000 sec
IN0 0.00002060 sec
TDav 1
SFO1 600.0045600 MHz
NUC1 1H
P1 11.50 usec
P2 23.00 usec
PLW1 28.00000000 W
SFO2 60.8096315 MHz
NUC2 15N
P3 14.60 usec
PLW2 236.00000000 W
GPNAM[1] SMSQ10.100
GPZ1 70.00 %
GPNAM[2] SMSQ10.100
GPZ2 30.00 %
GPNAM[3] SMSQ10.100
GPZ3 50.10 %
P16 1000.00 usec
F1 - Acquisition parameters
TD 128
SFO1 60.80963 MHz
FIDRES 379.247559 Hz
SW 399.145 ppm
FnMODE QF
F2 - Processing parameters
SI 2048
SF 600.0000022 MHz
WDW SINE
SSB 0
LB 0 Hz
GB 0
PC 1.40
F1 - Processing parameters
SI 1024
MC2 QF
SF 60.7974720 MHz
WDW SINE
SSB 0
LB 0 Hz
GB 0

30

Pollak Patrik	KP
KBr	2023.03.08.

BRUKER Alpha
Resolution: 2 cm-1
Number of Scans: 16

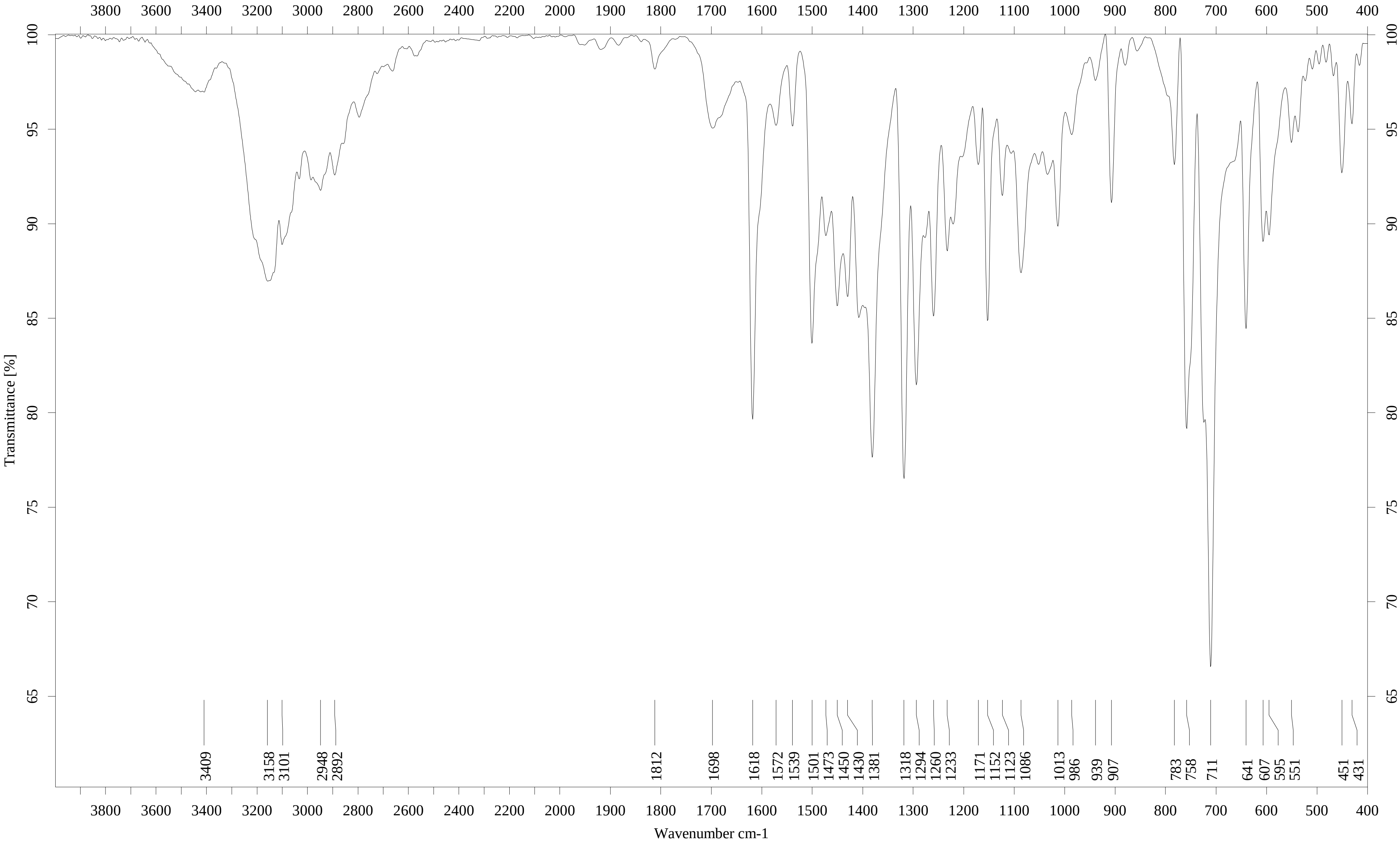

30

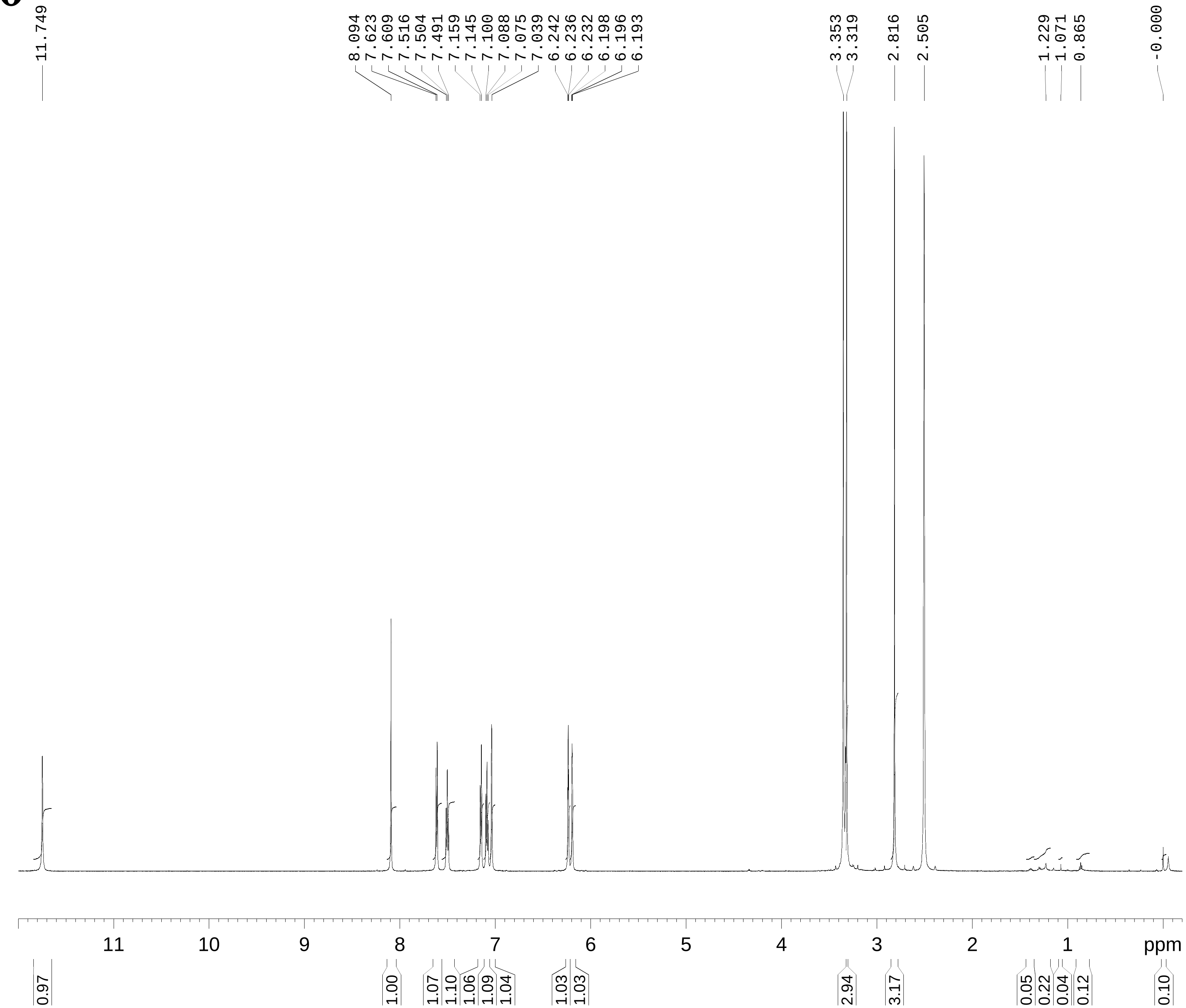

Standard 1H
140723
BAB0035_1
Pollak Patrik
2023.03.06. (DA)

Current Data Parameters

NAME	140723
EXPNO	11
PROCNO	1

F2 - Acquisition Parameters

Date_	20230307
Time	2.58 h
INSTRUM	spect
PROBHD	Z145856_0002 (
PULPROG	zg30
TD	65536
SOLVENT	DMSO
NS	16
DS	2
SWH	12019.230 Hz
FIDRES	0.366798 Hz
AQ	2.7262976 sec
RG	196.07
DW	41.600 usec
DE	25.00 usec
TE	295.0 K
D1	1.00000000 sec
TD0	1
SFO1	600.0537053 MHz
NUC1	1H
P1	11.50 usec
PLW1	28.00000000 W

F2 - Processing parameters

SI	65536
SF	600.0500011 MHz
WDW	no
SSB	0
LB	0 Hz
GB	0
PC	1.00

30

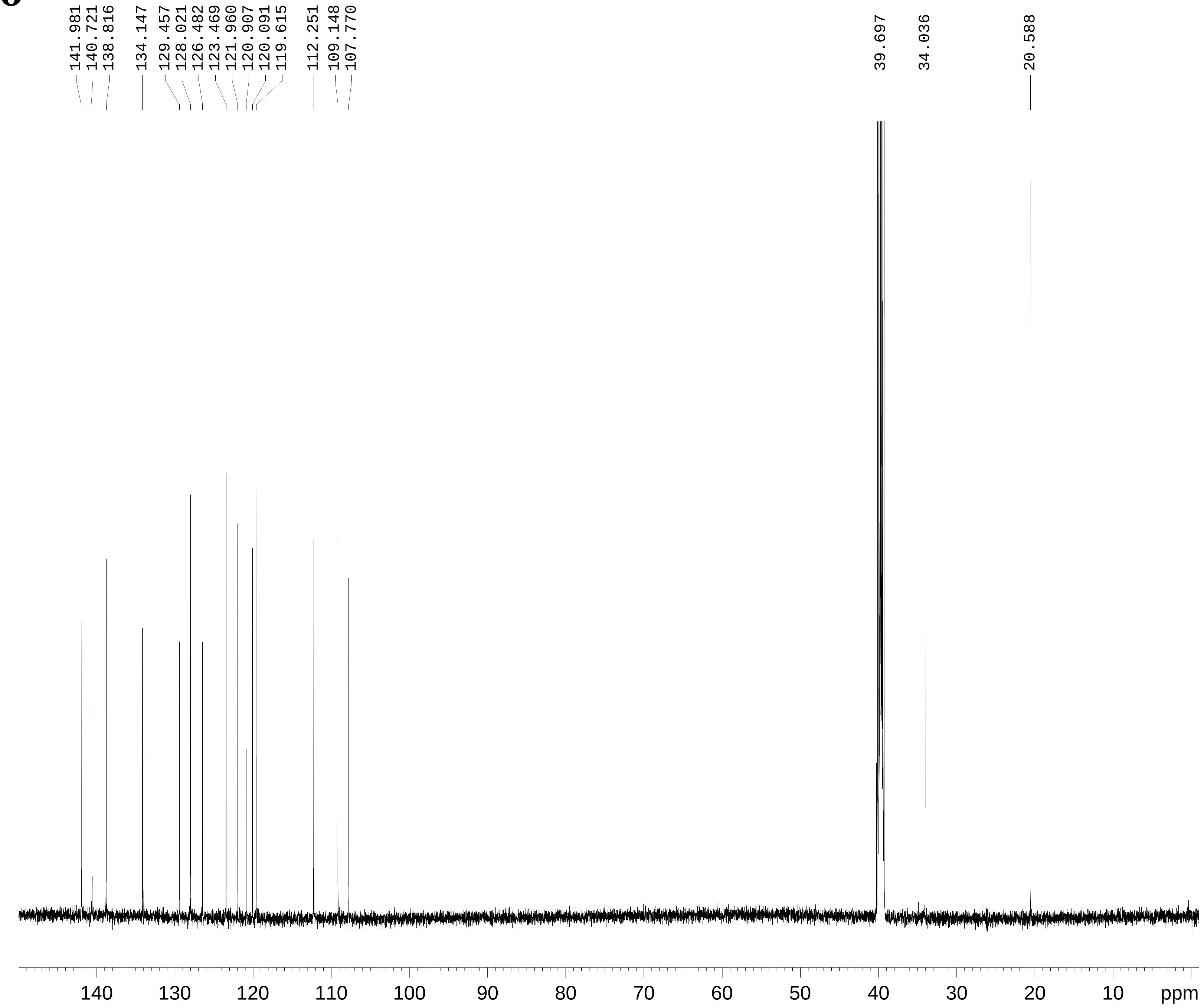

Standard 13C
140723
BAB0035_1
Pollak Patrik
2023.03.06. (DA)

Current Data Parameters	
NAME	140723
EXPNO	12
PROCNO	1
F2 - Acquisition Parameters	
Date_	20230307
Time	4.10 h
INSTRUM	spect
PROBHD	Z145856_0002 (
PULPROG	zgpg30
TD	65536
SOLVENT	DMSO
NS	2048
DS	4
SWH	36231.883 Hz
FIDRES	1.105709 Hz
AQ	0.9043968 sec
RG	196.07
DW	13.800 usec
DE	18.00 usec
TE	295.0 K
D1	1.00000000 sec
D11	0.03000000 sec
TD0	1
SFO1	150.8977808 MHz
NUC1	13C
P1	10.00 usec
PLW1	70.48699951 W
SFO2	600.0524002 MHz
NUC2	1H
CPDPRG[2	waltz16
PCPD2	80.00 usec
PLW2	29.46299934 W
PLW12	0.66293001 W
PLW13	0.33292001 W
F2 - Processing parameters	
SI	32768
SF	150.8827317 MHz
WDW	no
SSB	0
LB	0 Hz
GB	0
PC	1.40

30

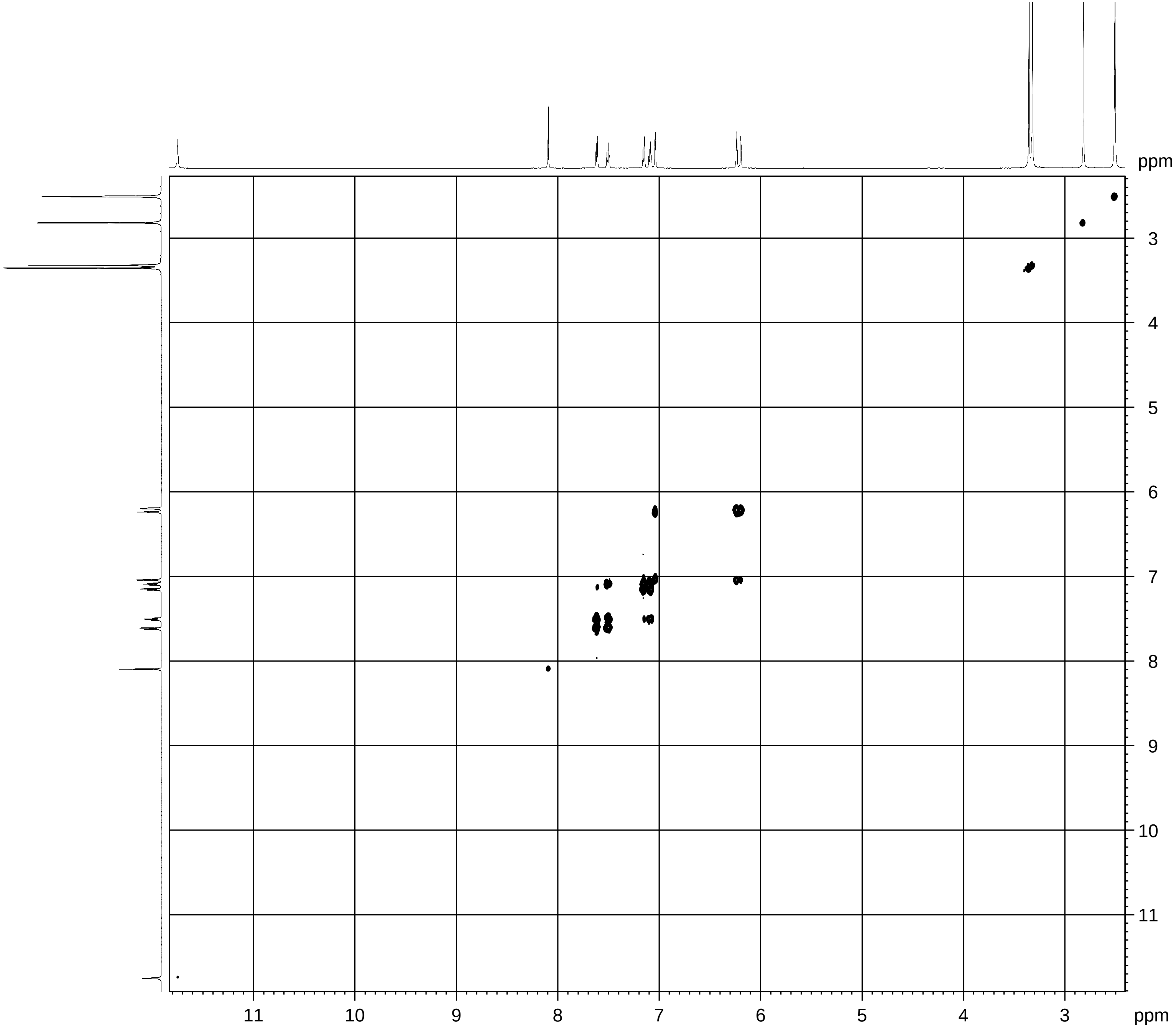

COSY
140723
BAB0035_1
Pollak Patrik
2023.03.06. (DA)

```
Current Data Parameters
NAME              140723
EXPNO                 13
PROCNO                 1

F2 - Acquisition Parameters
Date_           20230307
Time                4.23 h
INSTRUM            spect
PROBHD   Z145856_0002 (
PULPROG       cosygpmfqf
TD                  2048
SOLVENT             DMSO
NS                     2
DS                    16
SWH             7812.500 Hz
FIDRES          7.629395 Hz
AQ             0.1310720 sec
RG                196.07
DW                64.000 usec
DE                 25.00 usec
TE                 295.0 K
D0            0.00000300 sec
D1            2.00000000 sec
D13           0.00000400 sec
D16           0.00020000 sec
IN0           0.00012800 sec
TDav                   1
SFO1         600.0536003 MHz
NUC1                  1H
P1                 11.50 usec
PLW1         28.00000000 W
GPNAM[1]      SMSQ10.100
GPZ1               16.00 %
GPNAM[2]      SMSQ10.100
GPZ2               12.00 %
GPNAM[3]      SMSQ10.100
GPZ3               40.00 %
P16              1000.00 usec

F1 - Acquisition parameters
TD                   256
SFO1            600.0536 MHz
FIDRES         61.035156 Hz
SW                13.020 ppm
FnMODE                QF

F2 - Processing parameters
SI                  1024
SF           600.0500000 MHz
WDW                 SINE
SSB      0
LB       0 Hz
GB       0
PC                  1.40

F1 - Processing parameters
SI                  1024
MC2                   QF
SF           600.0500000 MHz
WDW                 SINE
SSB      0
LB       0 Hz
GB       0
```

30

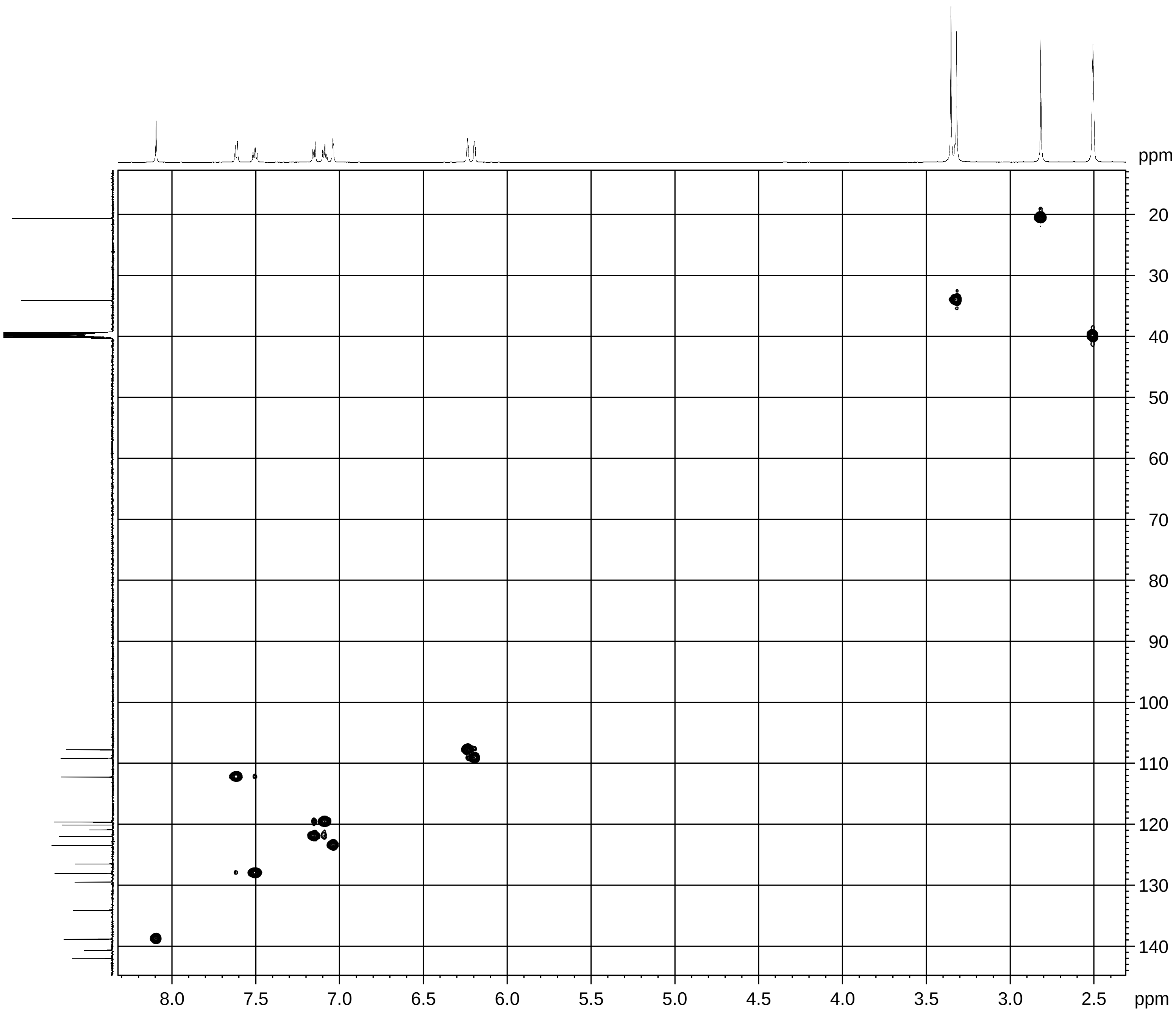

HSQC (140 Hz)
140723
BAB0035_1
Pollak Patrik
2023.03.06. (DA)

```
Current Data Parameters
NAME              140723
EXPNO                 14
PROCNO                 1

F2 - Acquisition Parameters
Date_           20230307
Time                4.49 h
INSTRUM            spect
PROBHD   Z145856_0002 (
PULPROG  hsqcetgpsisp2.2
TD                  1024
SOLVENT             DMSO
NS                     4
DS                    32
SWH             7812.500 Hz
FIDRES         15.258789 Hz
AQ             0.0655360 sec
RG                196.07
DW                64.000 usec
DE                 25.00 usec
TE                 295.0 K
CNST2        140.0000000
CNST17        -0.5000000
D0            0.00000300 sec
D1            1.50000000 sec
D4            0.00178571 sec
D11           0.03000000 sec
D16           0.00020000 sec
D24           0.00089000 sec
IN0           0.00001510 sec
TDav                   1
SFO1         600.0537803 MHz
NUC1                  1H
P1                 11.50 usec
P2                 23.00 usec
P28      0 usec
PLW1         28.00000000 W
SFO2         150.8985352 MHz
NUC2                 13C
CPDPRG[2 bi_p5m4sp_4sp.2
P3                 10.00 usec
P14               500.00 usec
P24              2000.00 usec
P63              1500.00 usec
PLW0     0 W
PLW2         70.48699951 W
PLW12         2.33019996 W
SPNAM[3] Crp60,0.5,20.1
SPOAL3             0.500
SPOFFS3  0 Hz
SPW3         10.77000046 W
SPNAM[7]    Crp60comp.4
SPOAL7             0.500
SPOFFS7  0 Hz
SPW7         10.77000046 W
SPNAM[14 Crp42,1.5,20.2
SPOAL14            0.500
SPOFFS14 0 Hz
SPW14         6.03100014 W
SPNAM[31 Crp42,1.5,20.2
SPOAL31            0.500
SPOFFS31 0 Hz
SPW31         1.50779998 W
GPNAM[1]     SMSQ10.100
GPZ1               80.00 %
GPNAM[2]     SMSQ10.100
GPZ2               20.10 %
GPNAM[3]     SMSQ10.100
GPZ3               11.00 %
GPNAM[4]     SMSQ10.100
GPZ4               -5.00 %
P16              1000.00 usec
P19               600.00 usec

F1 - Acquisition parameters
TD                   256
SFO1            150.8985 MHz
FIDRES        258.692047 Hz
SW               219.436 ppm
FnMODE    Echo-Antiecho

F2 - Processing parameters
SI                  1024
SF           600.0500010 MHz
WDW                QSINE
```

30

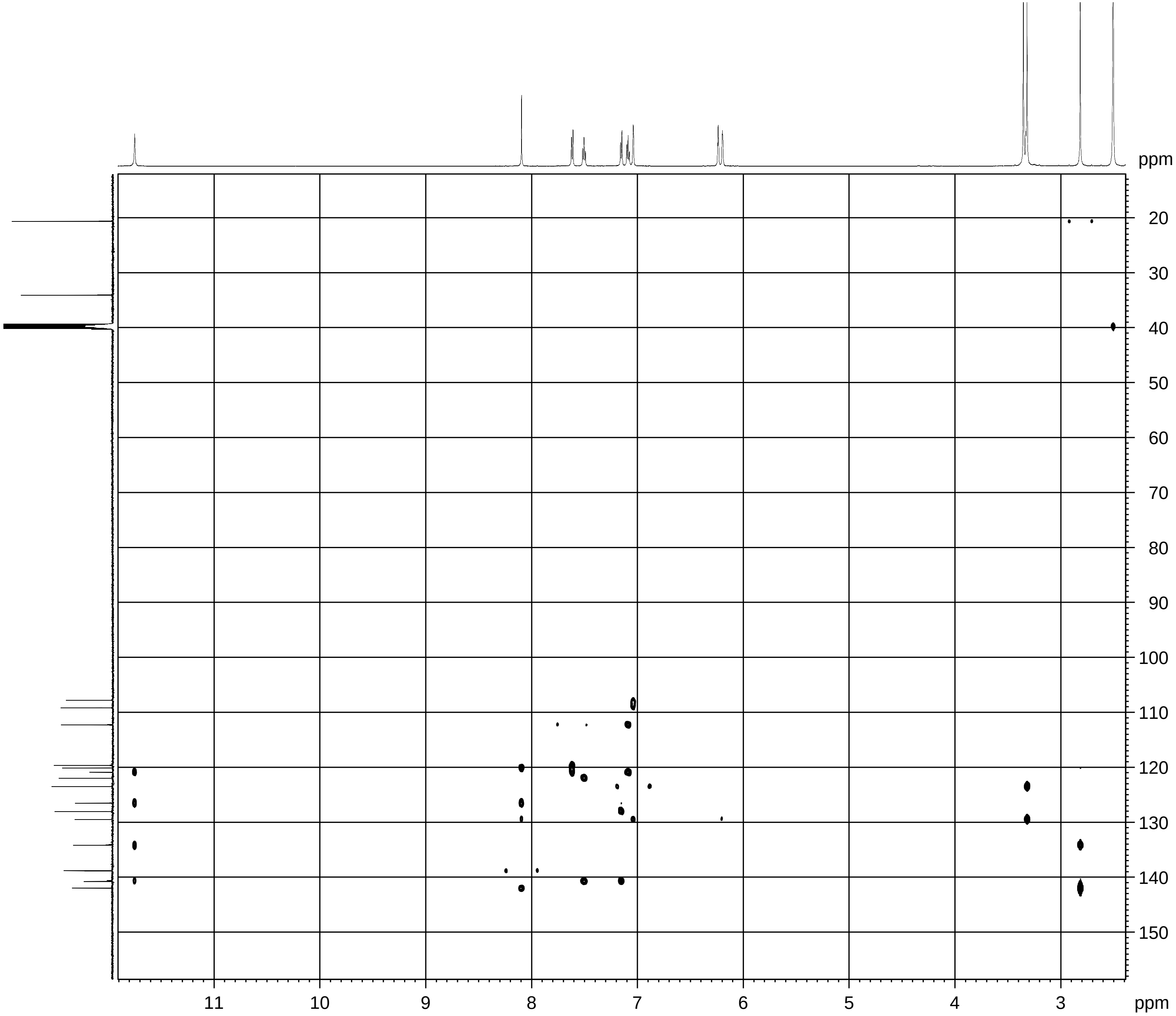

HMBC (8 Hz, 140 Hz)
140723
BAB0035_1
Pollak Patrik
2023.03.06. (DA)

```
Current Data Parameters
NAME              140723
EXPNO                 15
PROCNO                 1

F2 - Acquisition Parameters
Date_           20230307
Time                5.23 h
INSTRUM            spect
PROBHD   Z145856_0002 (
PULPROG    hmbcgplpndqf
TD                  2048
SOLVENT             DMSO
NS                     4
DS                    16
SWH             7812.500 Hz
FIDRES          7.629395 Hz
AQ             0.1310720 sec
RG                196.07
DW                64.000 usec
DE                 25.00 usec
TE                 295.0 K
CNST2        140.0000000
CNST13         8.0000000
D0            0.00000300 sec
D1            1.50000000 sec
D2            0.00357143 sec
D6            0.06250000 sec
D16           0.00020000 sec
IN0           0.00001510 sec
TDav                   1
SFO1         600.0537803 MHz
NUC1                  1H
P1                 11.50 usec
P2                 23.00 usec
PLW1         28.00000000 W
SFO2         150.8985352 MHz
NUC2                 13C
P3                 10.00 usec
PLW2         70.48699951 W
GPNAM[1]      SMSQ10.100
GPZ1               50.00 %
GPNAM[2]      SMSQ10.100
GPZ2               30.00 %
GPNAM[3]      SMSQ10.100
GPZ3               40.10 %
P16              1000.00 usec

F1 - Acquisition parameters
TD                   256
SFO1            150.8985 MHz
FIDRES        258.692047 Hz
SW               219.436 ppm
FnMODE                QF

F2 - Processing parameters
SI                  2048
SF           600.0500010 MHz
WDW                 SINE
SSB       0
LB        0 Hz
GB        0
PC                  1.40

F1 - Processing parameters
SI                  1024
MC2                   QF
SF           150.8827317 MHz
WDW                 SINE
SSB       0
LB        0 Hz
GB        0
```

31

Pollak Patrik	KP
KBr	2/20/2024

BRUKER Alpha
Resolution: 2 cm-1
Number of Scans: 16

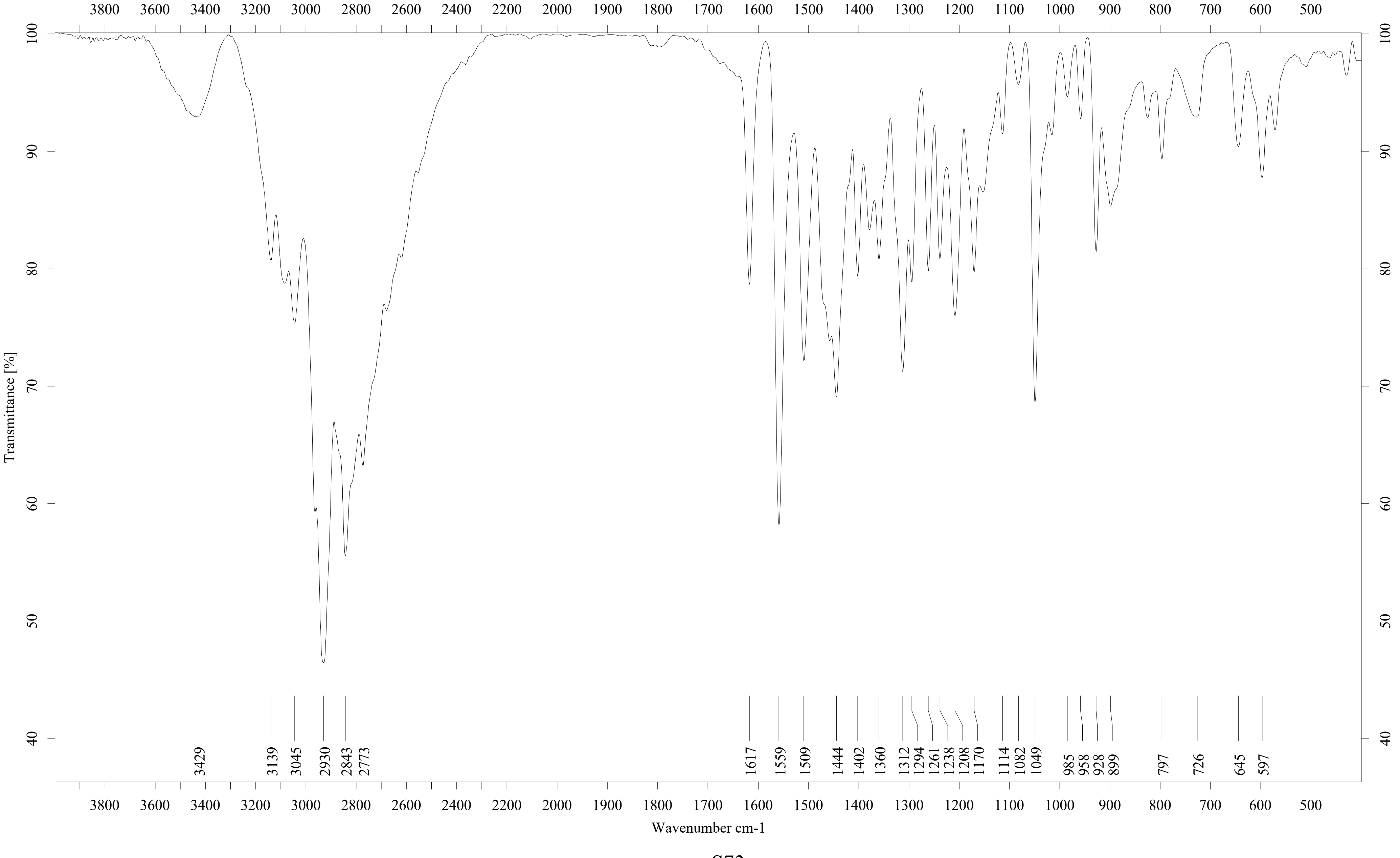

31

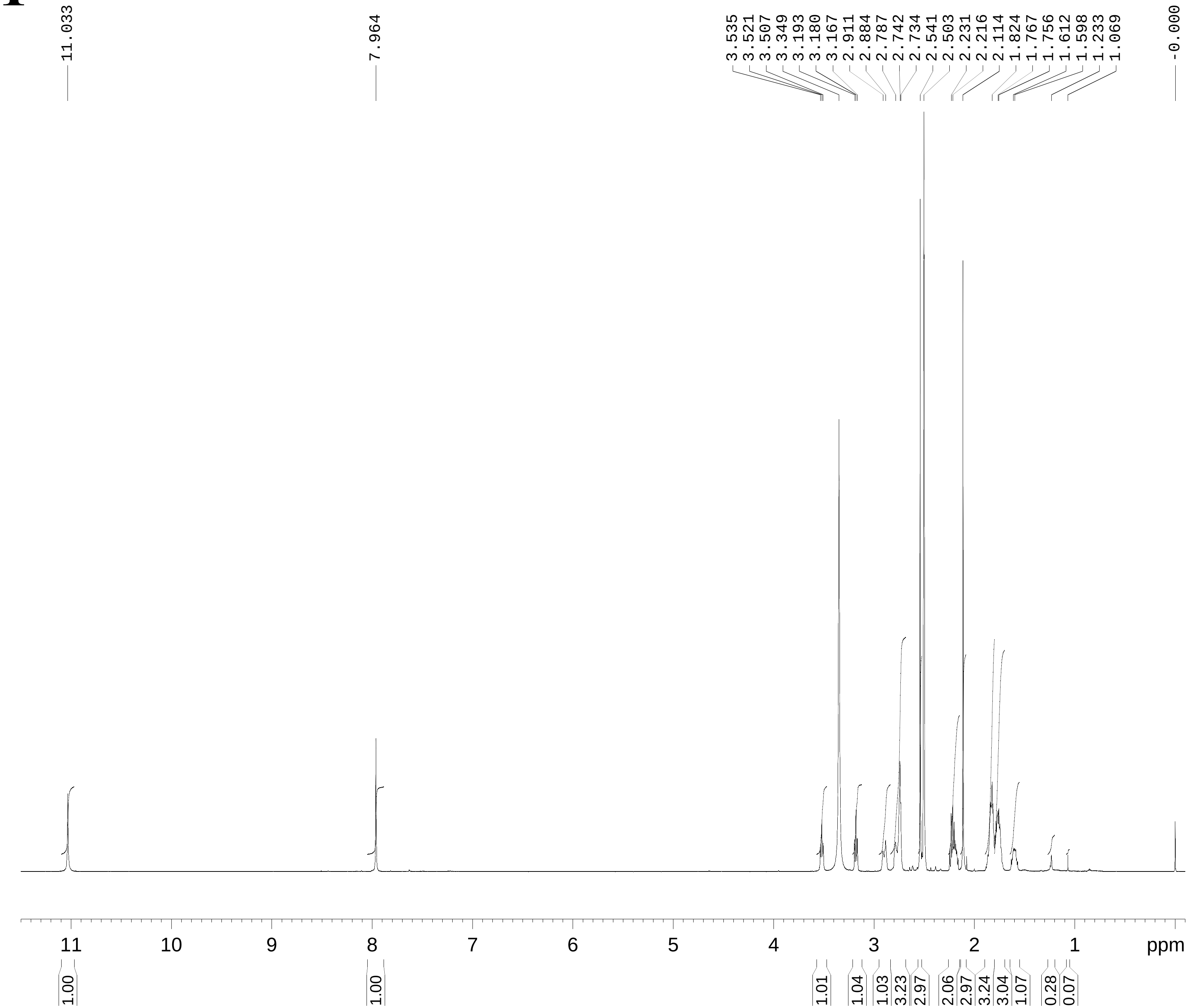

Standard 1H
142469
BAB0141_1
Pollak Patrik
2024.02.20. (KP)

Current Data Parameters

NAME	142469
EXPNO	11
PROCNO	1

F2 - Acquisition Parameters

Date_	20240220
Time	15.56 h
INSTRUM	spect
PROBHD	Z145856_0002 (
PULPROG	zg30
TD	65536
SOLVENT	DMSO
NS	16
DS	2
SWH	12019.230 Hz
FIDRES	0.366798 Hz
AQ	2.7262976 sec
RG	196.07
DW	41.600 usec
DE	25.00 usec
TE	295.0 K
D1	1.00000000 sec
TD0	1
SFO1	600.0037050 MHz
NUC1	1H
P1	11.50 usec
PLW1	28.00000000 W

F2 - Processing parameters

SI	65536
SF	600.0000029 MHz
WDW	EM
SSB	0
LB	0.30 Hz
GB	0
PC	1.00

31

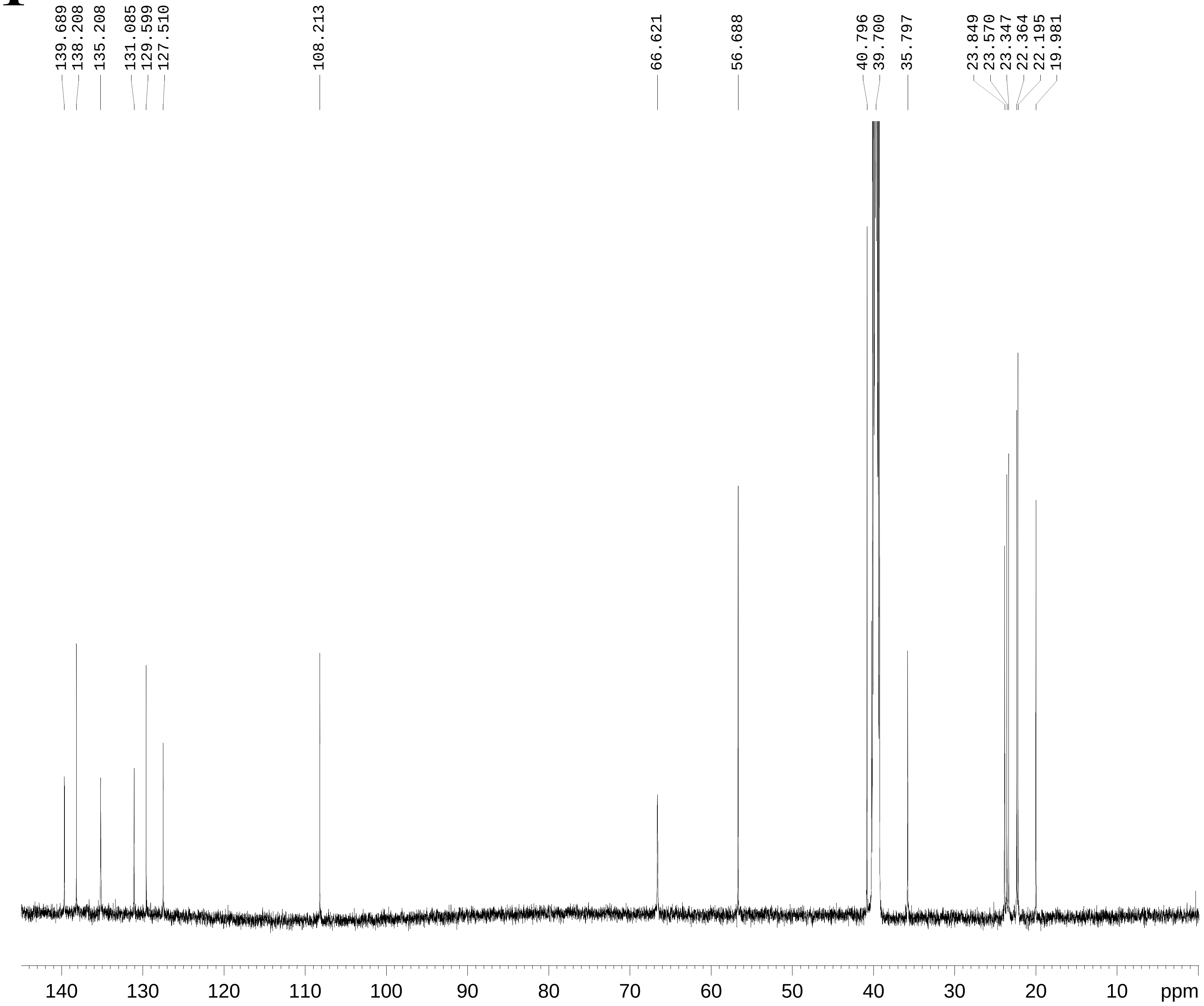

Standard 13C
142469
BAB0141_1
Pollak Patrik
2024.02.20. (KP)

Current Data Parameters	
NAME	142469
EXPNO	12
PROCNO	1

F2 - Acquisition Parameters	
Date_	20240220
Time	17.05 h
INSTRUM	spect
PROBHD	Z145856_0002 (
PULPROG	zgpg30
TD	65536
SOLVENT	DMSO
NS	2048
DS	4
SWH	36231.883 Hz
FIDRES	1.105709 Hz
AQ	0.9043968 sec
RG	196.07
DW	13.800 usec
DE	18.00 usec
TE	295.0 K
D1	1.00000000 sec
D11	0.03000000 sec
TD0	1
SFO1	150.8852070 MHz
NUC1	13C
P1	9.90 usec
PLW1	71.00000000 W
SFO2	600.0024000 MHz
NUC2	1H
CPDPRG[2	waltz16
PCPD2	80.00 usec
PLW2	32.90000153 W
PLW12	0.70370001 W
PLW13	0.35339001 W

F2 - Processing parameters	
SI	32768
SF	150.8701596 MHz
WDW	EM
SSB	0
LB	1.00 Hz
GB	0
PC	1.40

31

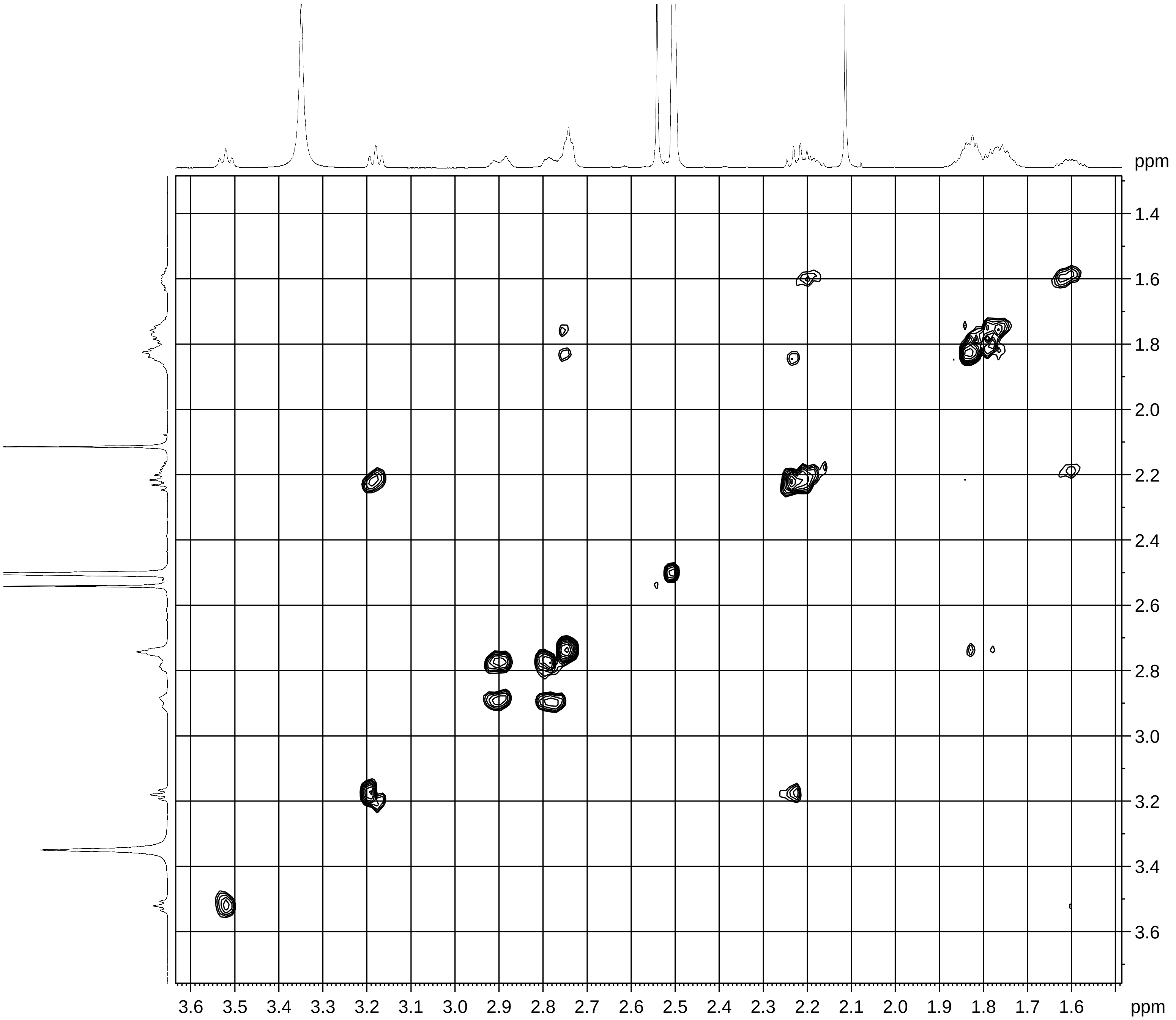

COSY
142469
BAB0141_1
Pollak Patrik
2024.02.20. (KP)

```
Current Data Parameters
NAME              142469
EXPNO                 13
PROCNO                 1

F2 - Acquisition Parameters
Date_           20240220
Time               17.07 h
INSTRUM            spect
PROBHD   Z145856_0002 (
PULPROG       cosygpmfqf
TD                  2048
SOLVENT             DMSO
NS                     2
DS                    16
SWH             7812.500 Hz
FIDRES          7.629395 Hz
AQ             0.1310720 sec
RG                196.07
DW                64.000 usec
DE                 25.00 usec
TE                 295.0 K
D0            0.00000300 sec
D1            2.00000000 sec
D13           0.00000400 sec
D16           0.00020000 sec
IN0           0.00012800 sec
TDav                   1
SFO1        600.0036000 MHz
NUC1                  1H
P1                 11.50 usec
PLW1         28.00000000 W
GPNAM[1]      SMSQ10.100
GPZ1               16.00 %
GPNAM[2]      SMSQ10.100
GPZ2               12.00 %
GPNAM[3]      SMSQ10.100
GPZ3               40.00 %
P16              1000.00 usec

F1 - Acquisition parameters
TD                   256
SFO1           600.0036 MHz
FIDRES        61.035156 Hz
SW                13.021 ppm
FnMODE                QF

F2 - Processing parameters
SI                  1024
SF          600.0000029 MHz
WDW                 SINE
SSB       0
LB        0 Hz
GB        0
PC                  1.40

F1 - Processing parameters
SI                  1024
MC2                   QF
SF          600.0000029 MHz
WDW                 SINE
SSB       0
LB        0 Hz
GB        0
```

31

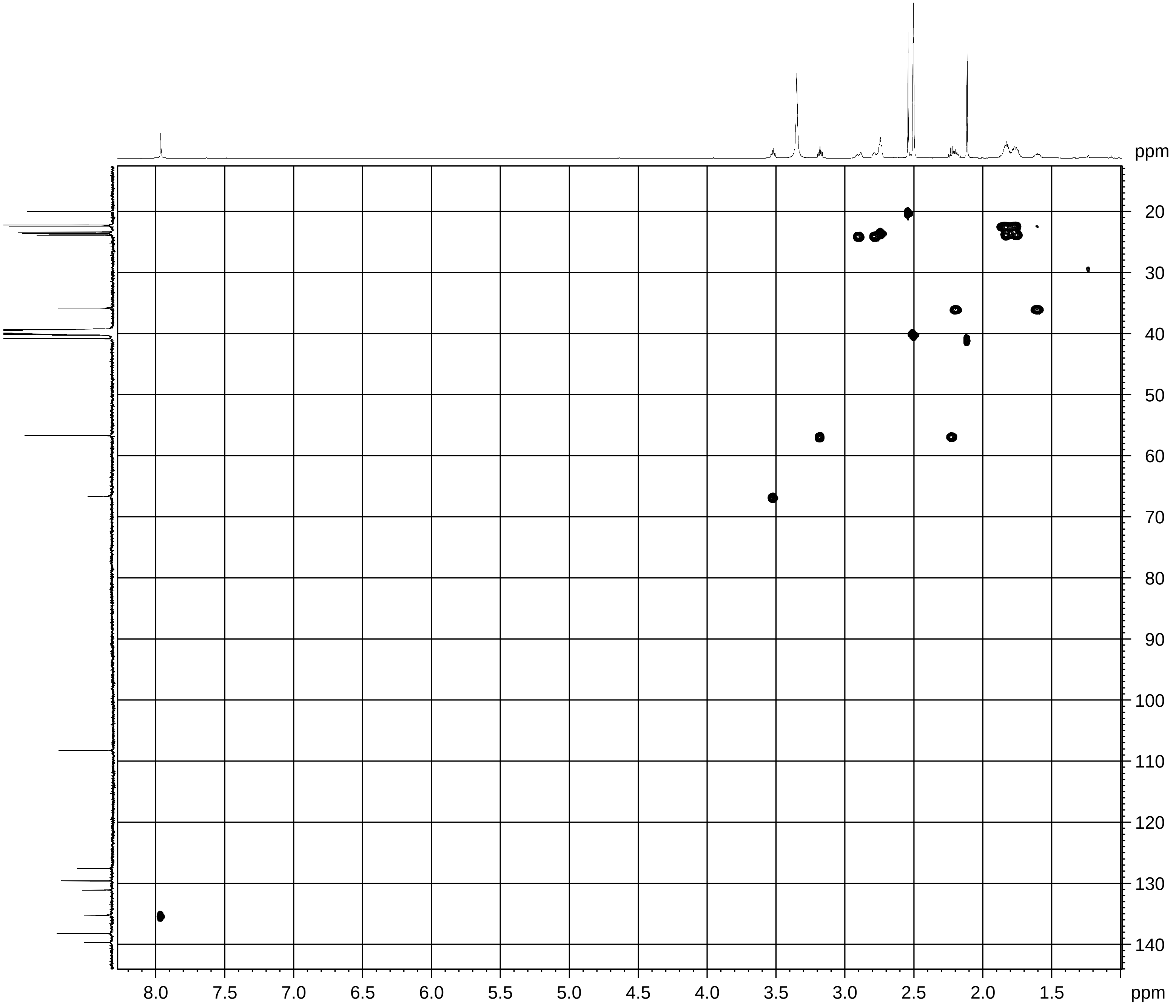

HSQC (140Hz)
142469
BAB0141_1
Pollak Patrik
2024.02.20. (KP)

```
Current Data Parameters
NAME              142469
EXPNO                 14
PROCNO                 1

F2 - Acquisition Parameters
Date_           20240220
Time               17.27 h
INSTRUM             spect
PROBHD   Z145856_0002 (
PULPROG  hsqcetgpsisp2.2
TD                  2048
SOLVENT             DMSO
NS                     4
DS                    32
SWH             7812.500 Hz
FIDRES          7.629395 Hz
AQ             0.1310720 sec
RG                196.07
DW                64.000 usec
DE                 25.00 usec
TE                 295.0 K
CNST2        140.0000000
CNST17        -0.5000000
D0            0.00000300 sec
D1            1.50000000 sec
D4            0.00178571 sec
D11           0.03000000 sec
D16           0.00020000 sec
D24           0.00089000 sec
IN0           0.00001510 sec
TDav                   1
SFO1         600.0036000 MHz
NUC1                  1H
P1                 11.50 usec
P2                 23.00 usec
P28      0 usec
PLW1         28.00000000 W
SFO2         150.8867157 MHz
NUC2                 13C
CPDPRG[2 bi_p5m4sp_4sp.2
P3                  9.90 usec
P14               500.00 usec
P24              2000.00 usec
P63              1500.00 usec
PLW0     0 W
PLW2         71.00000000 W
PLW12         2.30040002 W
SPNAM[3] Crp60,0.5,20.1
SPOAL3             0.500
SPOFFS3  0 Hz
SPW3         10.63199997 W
SPNAM[7]    Crp60comp.4
SPOAL7             0.500
SPOFFS7  0 Hz
SPW7         10.63199997 W
SPNAM[14 Crp42,1.5,20.2
SPOAL14            0.500
SPOFFS14 0 Hz
SPW14         5.95400000 W
SPNAM[31 Crp42,1.5,20.2
SPOAL31            0.500
SPOFFS31 0 Hz
SPW31         1.48850000 W
GPNAM[1]     SMSQ10.100
GPZ1               80.00 %
GPNAM[2]     SMSQ10.100
GPZ2               20.10 %
GPNAM[3]     SMSQ10.100
GPZ3               11.00 %
GPNAM[4]     SMSQ10.100
GPZ4               -5.00 %
P16              1000.00 usec
P19               600.00 usec

F1 - Acquisition parameters
TD                   256
SFO1            150.8867 MHz
FIDRES        258.692047 Hz
SW               219.453 ppm
FnMODE      Echo-Antiecho

F2 - Processing parameters
SI                  1024
SF           600.0000000 MHz
WDW                QSINE
```

31

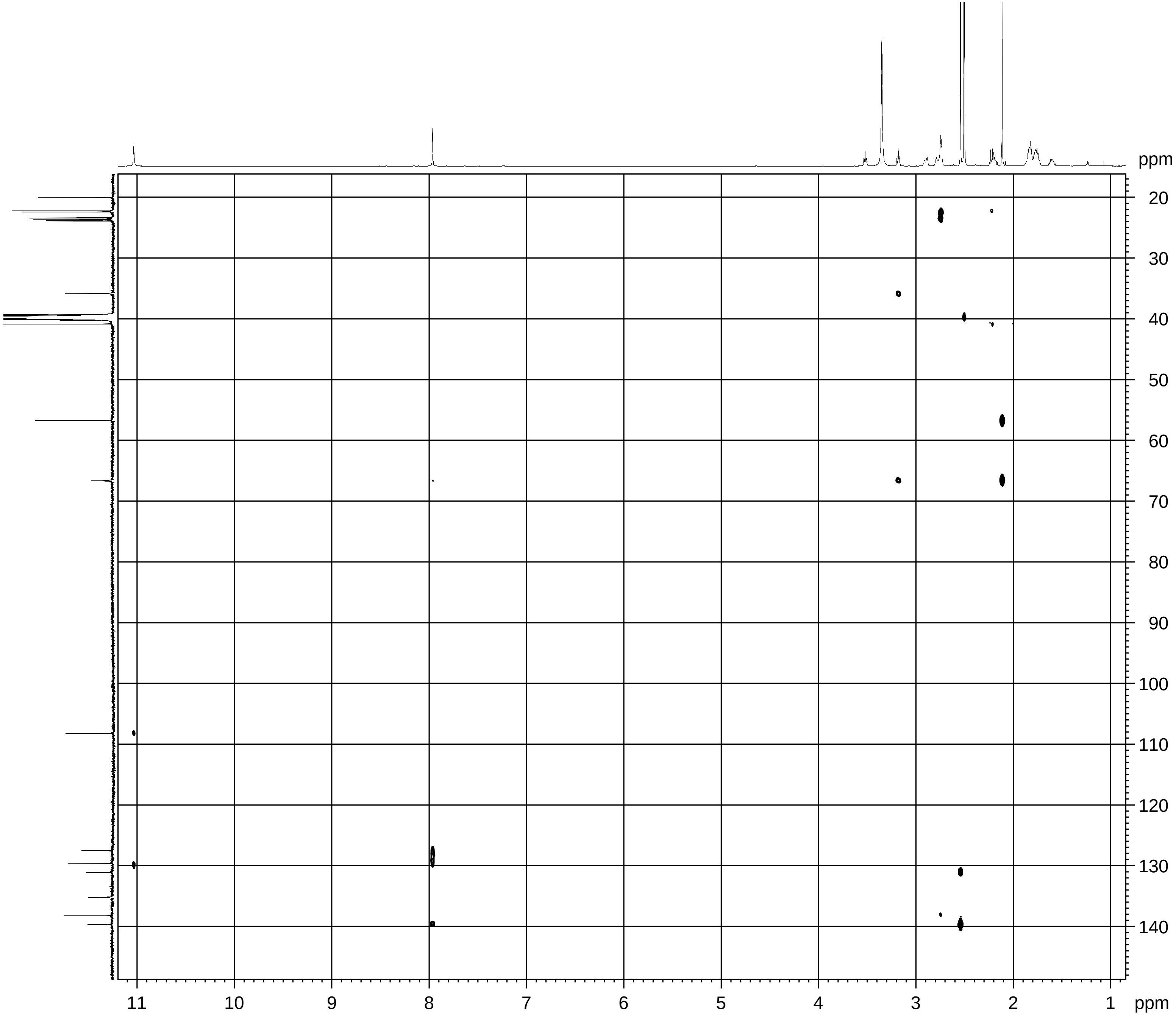

HMBC (8Hz, 140Hz)
142469
BAB0141_1
Pollak Patrik
2024.02.20. (KP)

```
Current Data Parameters
NAME             142469
EXPNO                15
PROCNO                1

F2 - Acquisition Parameters
Date_          20240220
Time              17.57 h
INSTRUM           spect
PROBHD   Z145856_0002 (
PULPROG    hmbcgplpndqf
TD                 2048
SOLVENT            DMSO
NS                    4
DS                   16
SWH            7812.500 Hz
FIDRES         7.629395 Hz
AQ            0.1310720 sec
RG               196.07
DW               64.000 usec
DE                25.00 usec
TE                295.0 K
CNST2       140.0000000
CNST13        8.0000000
D0           0.00000300 sec
D1           1.50000000 sec
D2           0.00357143 sec
D6           0.06250000 sec
D16          0.00020000 sec
IN0          0.00001510 sec
TDav                  1
SFO1        600.0037800 MHz
NUC1                 1H
P1                11.50 usec
P2                23.00 usec
PLW1        28.00000000 W
SFO2        150.8867157 MHz
NUC2                13C
P3                 9.90 usec
PLW2        71.00000000 W
GPNAM[1]     SMSQ10.100
GPZ1              50.00 %
GPNAM[2]     SMSQ10.100
GPZ2              30.00 %
GPNAM[3]     SMSQ10.100
GPZ3              40.10 %
P16             1000.00 usec

F1 - Acquisition parameters
TD                  256
SFO1           150.8867 MHz
FIDRES       258.692047 Hz
SW              219.453 ppm
FnMODE               QF

F2 - Processing parameters
SI                 2048
SF          600.0000029 MHz
WDW                SINE
SSB      0
LB       0 Hz
GB       0
PC                 1.40

F1 - Processing parameters
SI                 1024
MC2                  QF
SF          150.8701595 MHz
WDW                SINE
SSB      0
LB       0 Hz
GB       0
```

31

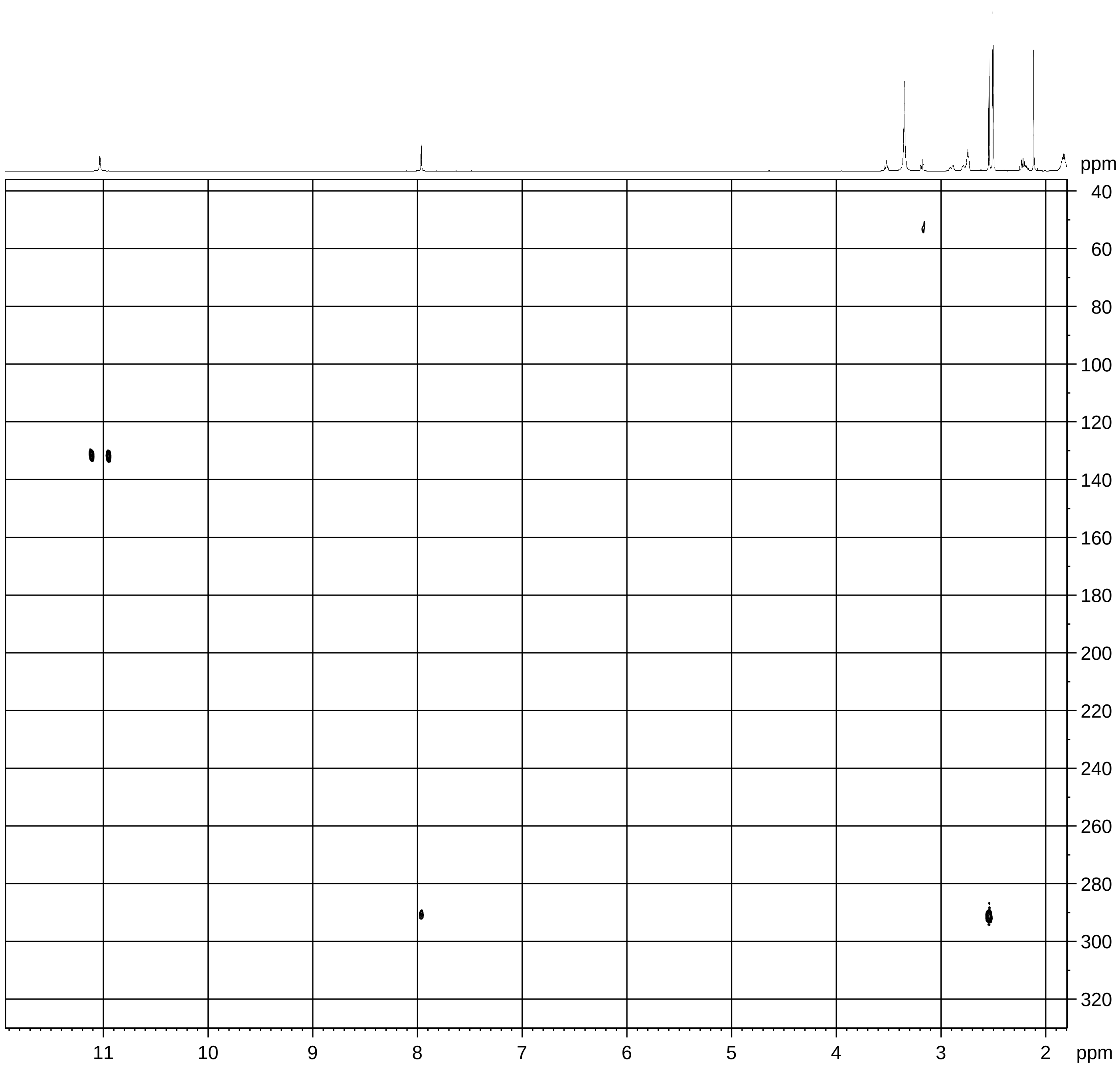

NHMBC
142469
BAB0141_1
Pollak Patrik
2024.02.20. (KP)

```
Current Data Parameters
NAME               142469
EXPNO                  16
PROCNO                  1

F2 - Acquisition Parameters
Date_            20240220
Time                18.29 h
INSTRUM             spect
PROBHD    Z145856_0002 (
PULPROG        hmbcgpndqf
TD                   2048
SOLVENT              DMSO
NS                      8
DS                     16
SWH              9615.385 Hz
FIDRES           9.390024 Hz
AQ              0.1064960 sec
RG                 196.07
DW                 52.000 usec
DE                  25.00 usec
TE                  295.0 K
CNST13          5.0000000
D0             0.00000300 sec
D1             2.00000000 sec
D6             0.10000000 sec
D16            0.00020000 sec
IN0            0.00002060 sec
TDav                    1
SFO1          600.0045600 MHz
NUC1                   1H
P1                  11.50 usec
P2                  23.00 usec
PLW1          28.00000000 W
SFO2           60.8096315 MHz
NUC2                  15N
P3                  15.00 usec
PLW2         210.13999939 W
GPNAM[1]       SMSQ10.100
GPZ1                70.00 %
GPNAM[2]       SMSQ10.100
GPZ2                30.00 %
GPNAM[3]       SMSQ10.100
GPZ3                50.10 %
P16               1000.00 usec

F1 - Acquisition parameters
TD                    128
SFO1             60.80963 MHz
FIDRES         379.247559 Hz
SW                399.145 ppm
FnMODE                 QF

F2 - Processing parameters
SI                   2048
SF            600.0000029 MHz
WDW                  SINE
SSB       0
LB        0 Hz
GB        0
PC                   1.40

F1 - Processing parameters
SI                   1024
MC2                    QF
SF             60.7974720 MHz
WDW                  SINE
SSB       0
LB        0 Hz
GB        0
```

BRUKER

32

Pollak Patrik	KP
Gyemant ATR	2023.05.09.

BRUKER Alpha
Resolution: 2 cm-1
Number of Scans: 32

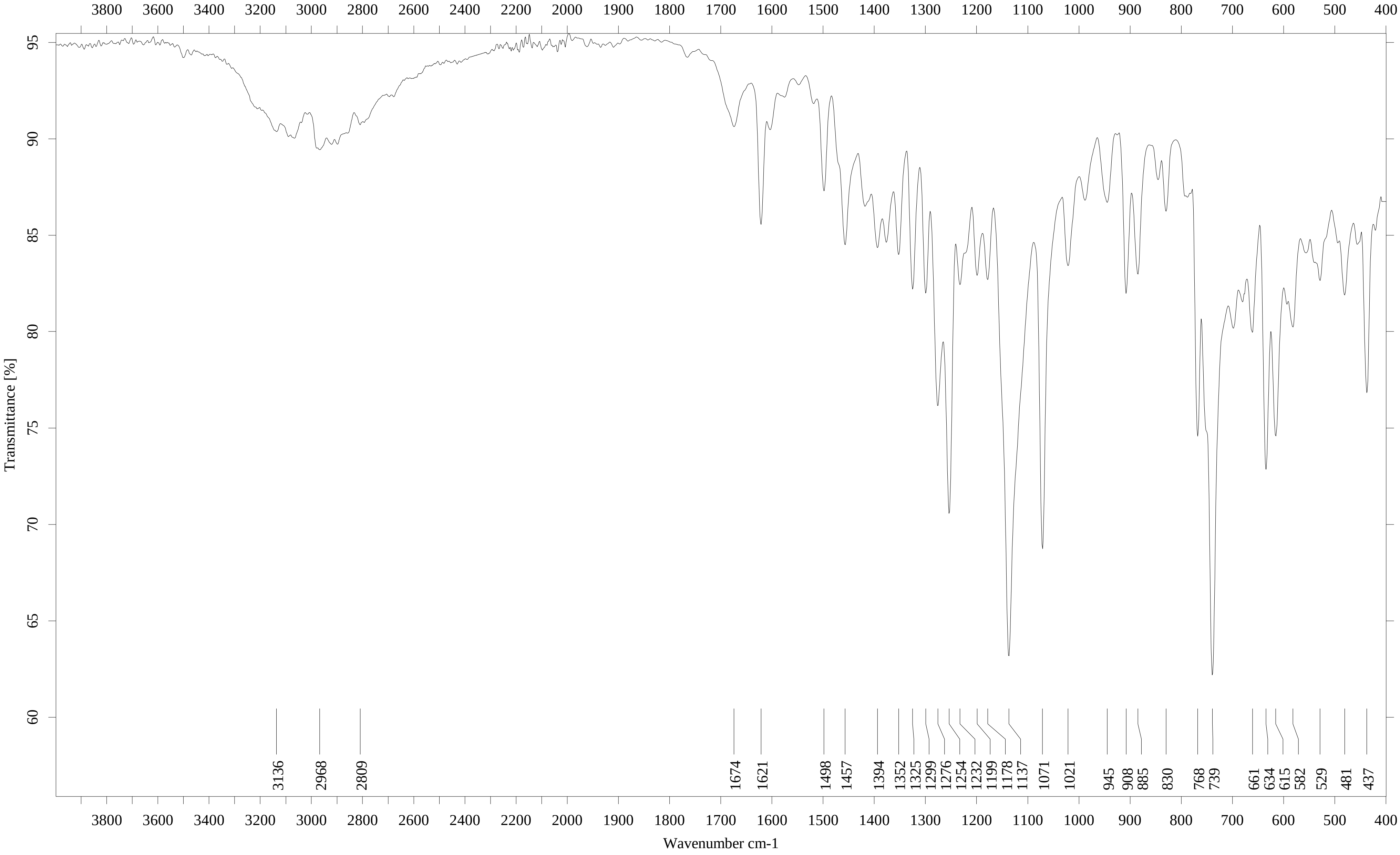

S80

32

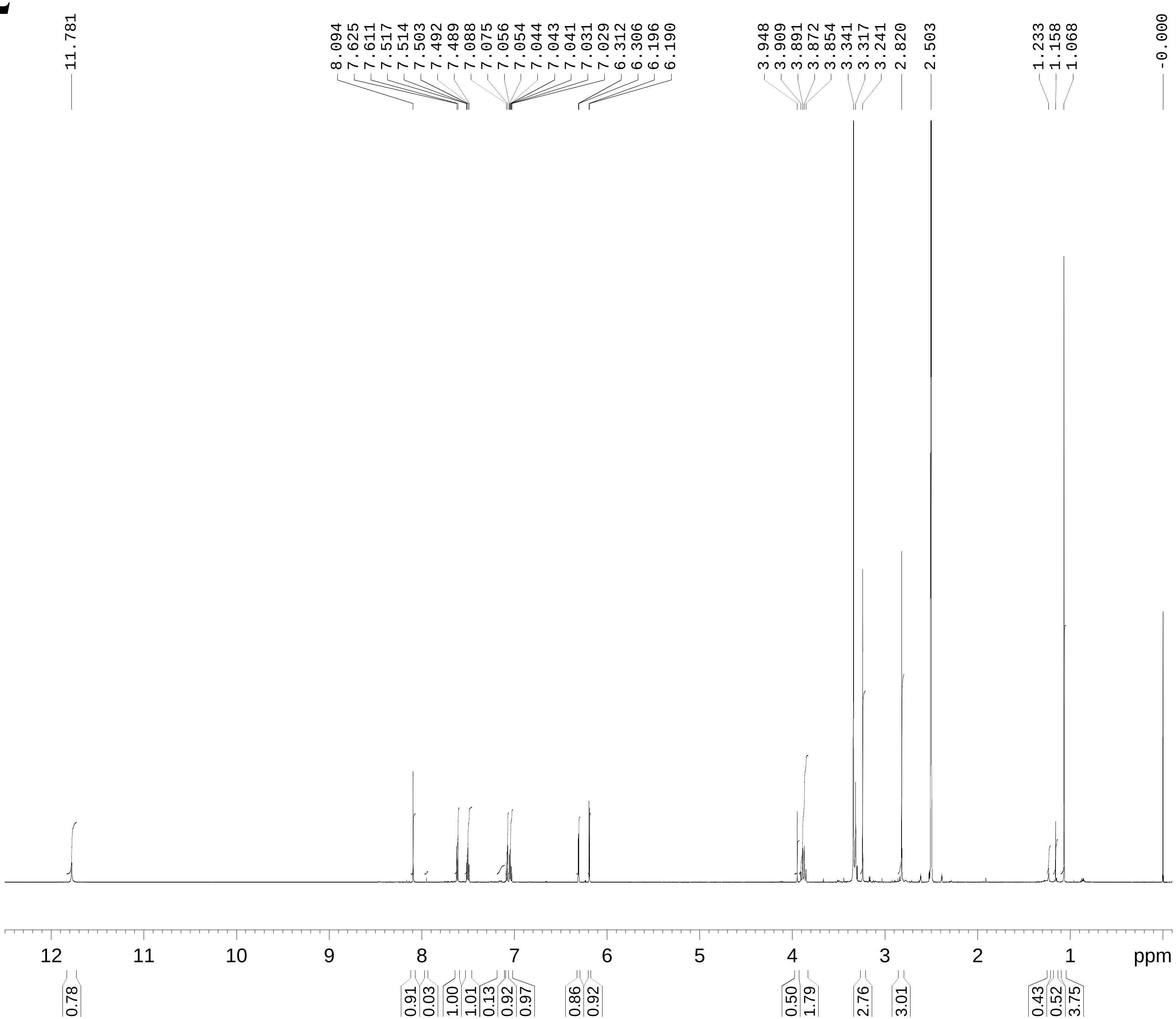

Standard 1H
141139
BAB0054_1
Pollak Patrik
2023.05.09. (KP)

Current Data Parameters
NAME 141139
EXPNO 11
PROCNO 1

F2 - Acquisition Parameters
Date_ 20230509
Time 23.18 h
INSTRUM spect
PROBHD Z145856_0002 (
PULPROG zg30
TD 65536
SOLVENT DMSO
NS 64
DS 2
SWH 12019.230 Hz
FIDRES 0.366798 Hz
AQ 2.7262976 sec
RG 196.07
DW 41.600 usec
DE 25.00 usec
TE 295.0 K
D1 1.00000000 sec
TD0 1
SFO1 600.0037050 MHz
NUC1 1H
P1 11.50 usec
PLW1 28.00000000 W

F2 - Processing parameters
SI 65536
SF 600.0000025 MHz
WDW no
SSB 0
LB 0 Hz
GB 0
PC 1.00

32

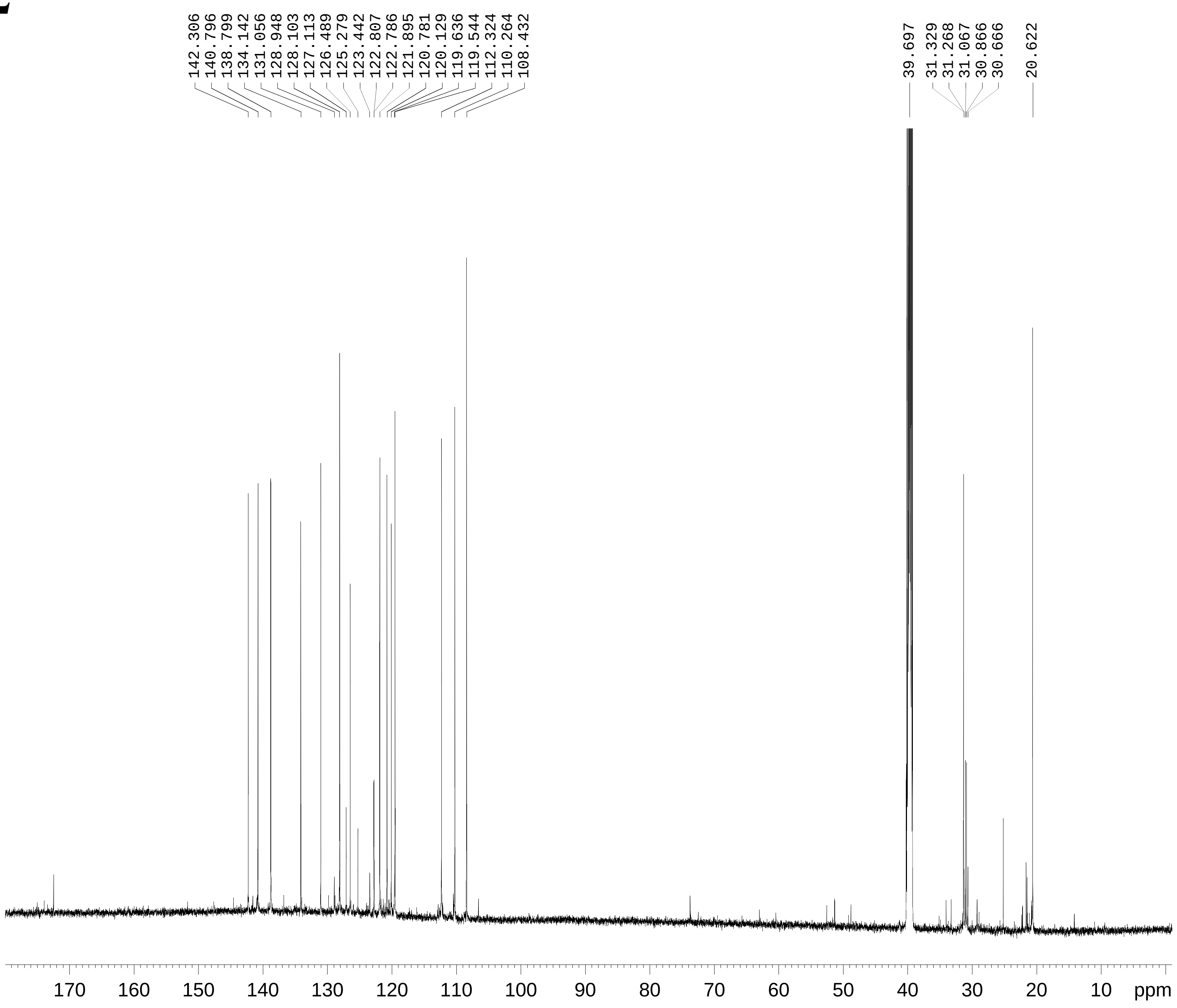

Standard 13C
141385
BAB0054_1B
Pollak Patrik
2023.06.20.(KP)

Current Data Parameters

NAME	141385
EXPNO	12
PROCNO	1

F2 - Acquisition Parameters

Date_	20230621
Time	2.31 h
INSTRUM	spect
PROBHD	Z145856_0002 (
PULPROG	zgpg30
TD	65536
SOLVENT	DMSO
NS	2048
DS	4
SWH	36231.883 Hz
FIDRES	1.105709 Hz
AQ	0.9043968 sec
RG	196.07
DW	13.800 usec
DE	18.00 usec
TE	295.0 K
D1	1.00000000 sec
D11	0.03000000 sec
TD0	1
SFO1	150.8852070 MHz
NUC1	13C
P1	9.90 usec
PLW1	80.09999847 W
SFO2	600.0024000 MHz
NUC2	1H
CPDPRG[2	waltz16
PCPD2	80.00 usec
PLW2	35.00000000 W
PLW12	0.74861997 W
PLW13	0.37595001 W

F2 - Processing parameters

SI	32768
SF	150.8701565 MHz
WDW	EM
SSB	0
LB	1.00 Hz
GB	0
PC	1.40

32

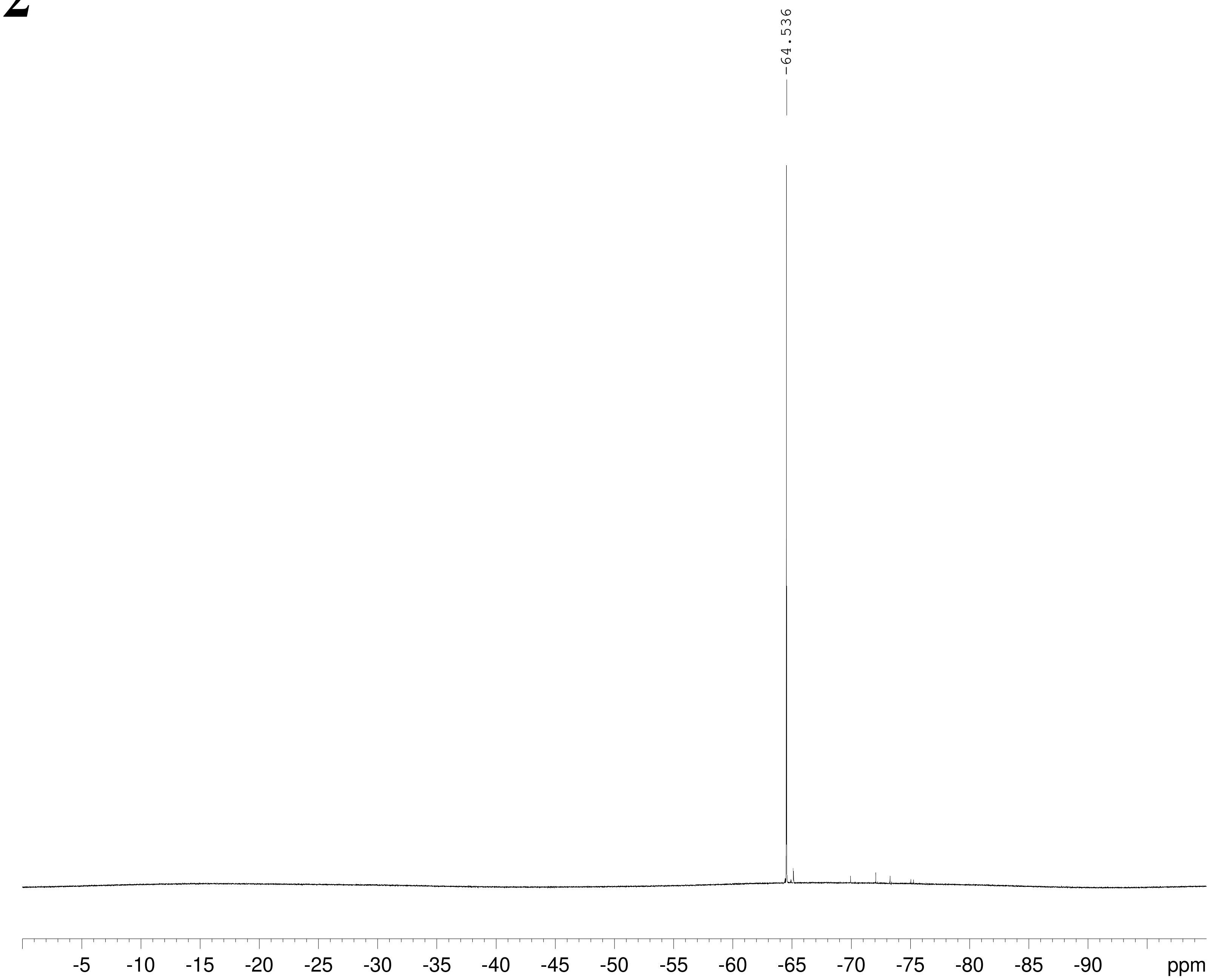

19F
141139
BAB0054_1
Pollak Patrik
2023.05.12. (DA)

Current Data Parameters

NAME	141139
EXPNO	20
PROCNO	1

F2 - Acquisition Parameters

Date_	20230512
Time	20.44 h
INSTRUM	spect
PROBHD	Z145856_0002 (
PULPROG	zgflqn
TD	131072
SOLVENT	DMSO
NS	16
DS	4
SWH	133928.578 Hz
FIDRES	2.043588 Hz
AQ	0.4893355 sec
RG	14.01
DW	3.733 usec
DE	18.00 usec
TE	295.0 K
D1	1.00000000 sec
TD0	1
SFO1	564.5076096 MHz
NUC1	19F
P1	14.70 usec
PLW1	23.70000076 W

F2 - Processing parameters

SI		65536
SF		564.5640660 MHz
WDW		EM
SSB	0	
LB		0.30 Hz
GB	0	
PC		1.00

32

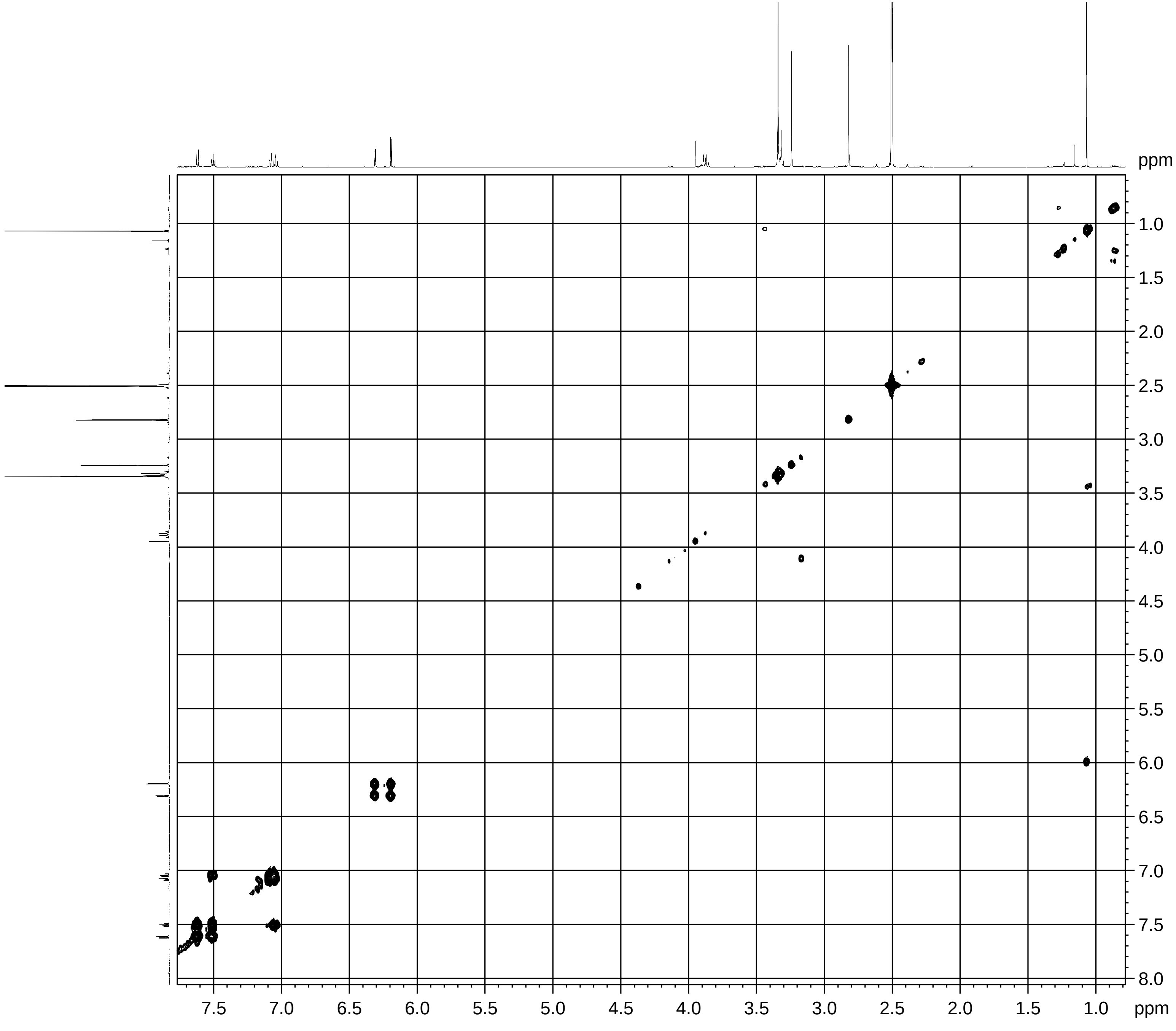

COSY
141139
BAB0054_1
Pollak Patrik
2023.05.09. (KP)

Current Data Parameters

NAME	141139
EXPNO	13
PROCNO	1

F2 - Acquisition Parameters

Date_	20230510
Time	3.49 h
INSTRUM	spect
PROBHD	Z145856_0002 (
PULPROG	cosygpmfqf
TD	2048
SOLVENT	DMSO
NS	16
DS	16
SWH	7812.500 Hz
FIDRES	7.629395 Hz
AQ	0.1310720 sec
RG	196.07
DW	64.000 usec
DE	25.00 usec
TE	295.0 K
D0	0.00000300 sec
D1	2.00000000 sec
D13	0.00000400 sec
D16	0.00020000 sec
IN0	0.00012800 sec
TDav	1
SFO1	600.0036000 MHz
NUC1	1H
P1	11.50 usec
PLW1	28.00000000 W
GPNAM[1]	SMSQ10.100
GPZ1	16.00 %
GPNAM[2]	SMSQ10.100
GPZ2	12.00 %
GPNAM[3]	SMSQ10.100
GPZ3	40.00 %
P16	1000.00 usec

F1 - Acquisition parameters

TD	256
SFO1	600.0036 MHz
FIDRES	61.035156 Hz
SW	13.021 ppm
FnMODE	QF

F2 - Processing parameters

SI	1024
SF	600.0000025 MHz
WDW	SINE
SSB	0
LB	0 Hz
GB	0
PC	1.40

F1 - Processing parameters

SI	1024
MC2	QF
SF	600.0000025 MHz
WDW	SINE
SSB	0
LB	0 Hz
GB	0

32

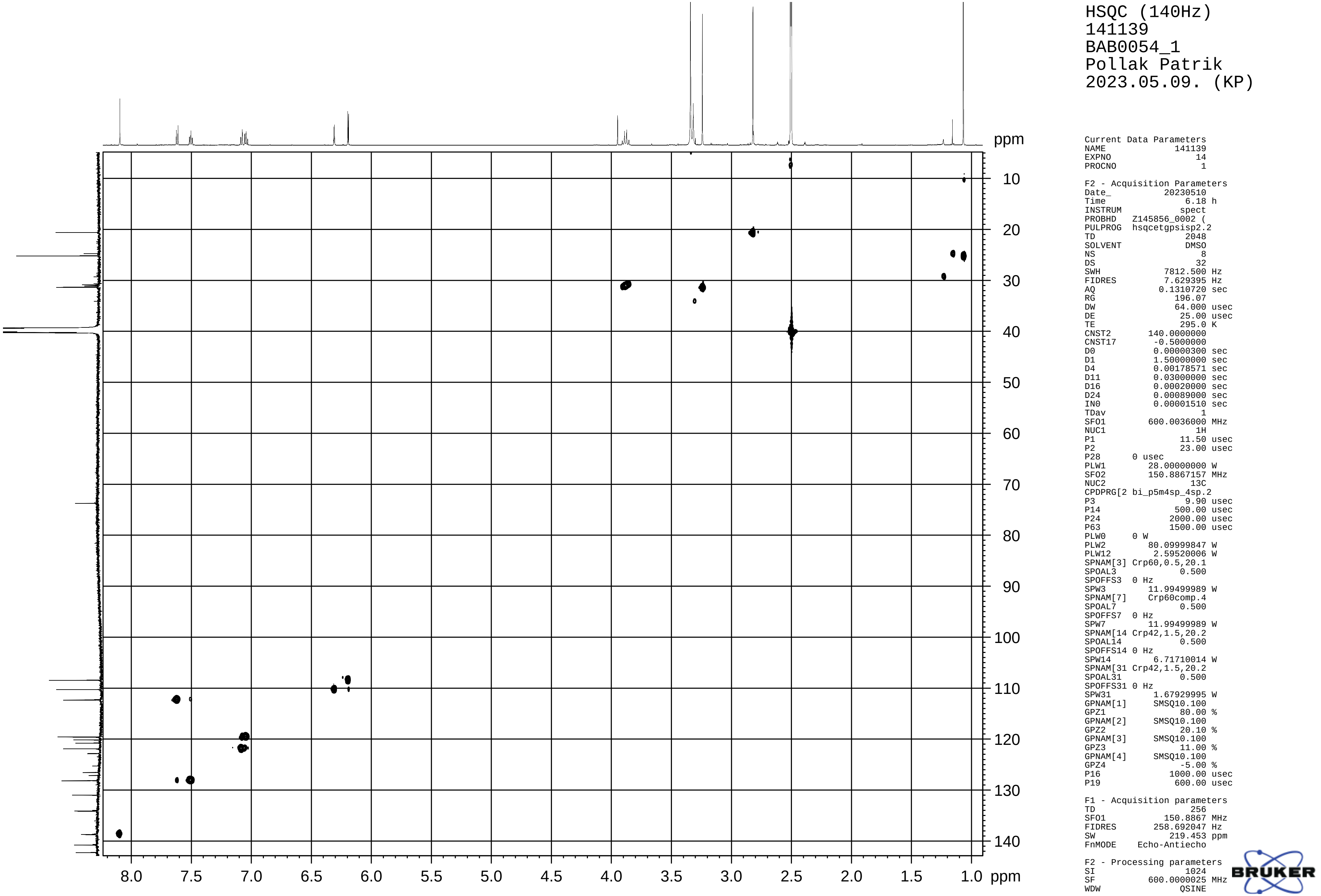
HSQC (140Hz)
141139
BAB0054_1
Pollak Patrik
2023.05.09. (KP)
Current Data Parameters
NAME 141139
EXPNO 14
PROCNO 1
F2 - Acquisition Parameters
Date_ 20230510
Time 6.18 h
INSTRUM spect
PROBHD Z145856_0002 (
PULPROG hsqcetgpsisp2.2
TD 2048
SOLVENT DMSO
NS 8
DS 32
SWH 7812.500 Hz
FIDRES 7.629395 Hz
AQ 0.1310720 sec
RG 196.07
DW 64.000 usec
DE 25.00 usec
TE 295.0 K
CNST2 140.0000000
CNST17 -0.5000000
D0 0.00000300 sec
D1 1.50000000 sec
D4 0.00178571 sec
D11 0.03000000 sec
D16 0.00020000 sec
D24 0.00089000 sec
IN0 0.00001510 sec
TDav 1
SFO1 600.0036000 MHz
NUC1 1H
P1 11.50 usec
P2 23.00 usec
P28 0 usec
PLW1 28.00000000 W
SFO2 150.8867157 MHz
NUC2 13C
CPDPRG[2 bi_p5m4sp_4sp.2
P3 9.90 usec
P14 500.00 usec
P24 2000.00 usec
P63 1500.00 usec
PLW0 0 W
PLW2 80.09999847 W
PLW12 2.59520006 W
SPNAM[3] Crp60,0.5,20.1
SPOAL3 0.500
SPOFFS3 0 Hz
SPW3 11.99499989 W
SPNAM[7] Crp60comp.4
SPOAL7 0.500
SPOFFS7 0 Hz
SPW7 11.99499989 W
SPNAM[14 Crp42,1.5,20.2
SPOAL14 0.500
SPOFFS14 0 Hz
SPW14 6.71710014 W
SPNAM[31 Crp42,1.5,20.2
SPOAL31 0.500
SPOFFS31 0 Hz
SPW31 1.67929995 W
GPNAM[1] SMSQ10.100
GPZ1 80.00 %
GPNAM[2] SMSQ10.100
GPZ2 20.10 %
GPNAM[3] SMSQ10.100
GPZ3 11.00 %
GPNAM[4] SMSQ10.100
GPZ4 -5.00 %
P16 1000.00 usec
P19 600.00 usec
F1 - Acquisition parameters
TD 256
SFO1 150.8867 MHz
FIDRES 258.692047 Hz
SW 219.453 ppm
FnMODE Echo-Antiecho
F2 - Processing parameters
SI 1024
SF 600.0000025 MHz
WDW QSINE
ppm
10
20
30
40
50
60
70
80
90
100
110
120
130
140
8.0
7.5
7.0
6.5
6.0
5.5
5.0
4.5
4.0
3.5
3.0
2.5
2.0
1.5
1.0
ppm

32

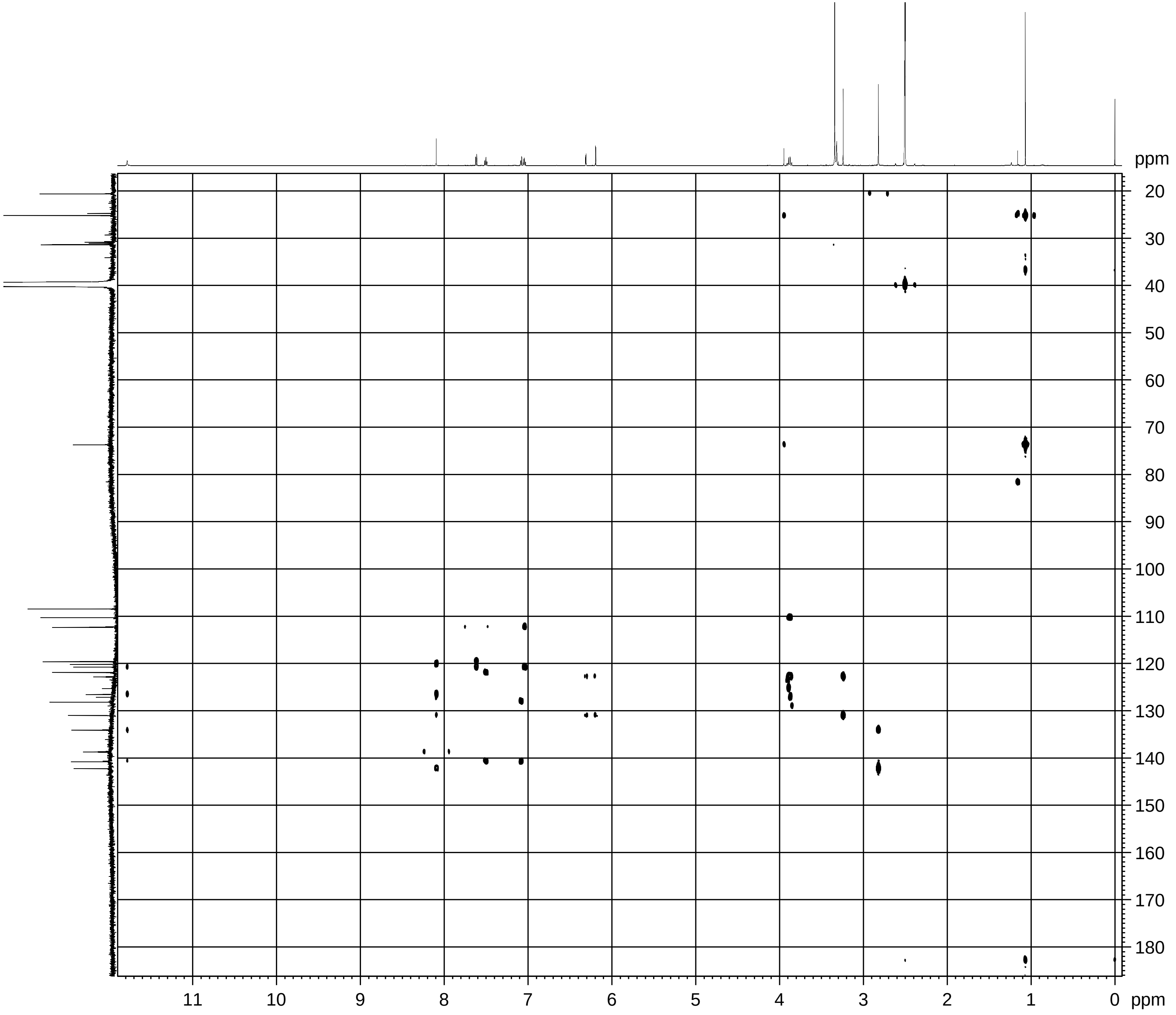

HMBC (8Hz, 140Hz)
141139
BAB0054_1
Pollak Patrik
2023.05.09. (KP)

```
Current Data Parameters
NAME              141139
EXPNO                 15
PROCNO                 1

F2 - Acquisition Parameters
Date_           20230510
Time                7.16 h
INSTRUM            spect
PROBHD    Z145856_0002 (
PULPROG    hmbcgplpndqf
TD                  2048
SOLVENT             DMSO
NS                     8
DS                    16
SWH             7812.500 Hz
FIDRES          7.629395 Hz
AQ             0.1310720 sec
RG                196.07
DW                64.000 usec
DE                 25.00 usec
TE                 295.0 K
CNST2        140.0000000
CNST13         8.0000000
D0            0.00000300 sec
D1            1.50000000 sec
D2            0.00357143 sec
D6            0.06250000 sec
D16           0.00020000 sec
IN0           0.00001510 sec
TDav                   1
SFO1        600.0037800 MHz
NUC1                  1H
P1                 11.50 usec
P2                 23.00 usec
PLW1         28.00000000 W
SFO2        150.8867157 MHz
NUC2                 13C
P3                  9.90 usec
PLW2         80.09999847 W
GPNAM[1]      SMSQ10.100
GPZ1               50.00 %
GPNAM[2]      SMSQ10.100
GPZ2               30.00 %
GPNAM[3]      SMSQ10.100
GPZ3               40.10 %
P16              1000.00 usec

F1 - Acquisition parameters
TD                   256
SFO1            150.8867 MHz
FIDRES        258.692047 Hz
SW               219.453 ppm
FnMODE                QF

F2 - Processing parameters
SI                  2048
SF           600.0000025 MHz
WDW                 SINE
SSB      0
LB       0 Hz
GB       0
PC                  1.40

F1 - Processing parameters
SI                  1024
MC2                   QF
SF           150.8701595 MHz
WDW                 SINE
SSB      0
LB       0 Hz
GB       0
```
