# Supplementary material for: Studies on the syntheses of β-carboline alkaloids brevicarine and brevicolline
Source: Beilstein J Org Chem. 2025 May 20;21:955–63. doi: 10.3762/bjoc.21.79 (PMC12117210; doi:10.3762/bjoc.21.79)

BEILSTEIN JOURNAL OF ORGANIC CHEMISTRY

# Supporting Information

for

# Studies on the syntheses of β-carboline alkaloids brevicarine and brevicolline

Benedek Batizi, Patrik Pollák, András Dancsó, Péter Keglevich, Gyula Simig, Balázs Volk and Mátyás Milen

*Beilstein J. Org. Chem.* **2025,** *21,* 955–963. doi:10.3762/bjoc.21.79

# Checkcif file for compound 31

License and Terms: This is a supporting information file under the terms of the Creative Commons Attribution License (https://creativecommons.org/licenses/by/4.0). Please note that the reuse, redistribution and reproduction in particular requires that the author(s) and source are credited and that individual graphics may be subject to special legal provisions.
The license is subject to the *Beilstein Journal of Organic Chemistry* terms and conditions: (https://www.beilstein-journals.org/bjoc/terms)

# checkCIF/PLATON report

You have not supplied any structure factors. As a result the full set of tests cannot be run.

THIS REPORT IS FOR GUIDANCE ONLY. IF USED AS PART OF A REVIEW PROCEDURE FOR PUBLICATION, IT SHOULD NOT REPLACE THE EXPERTISE OF AN EXPERIENCED CRYSTALLOGRAPHIC REFEREE.

No syntax errors found.    CIF dictionary    Interpreting this report

# Datablock: _142469_3

---

```
Bond precision:   C-C = 0.0049 A          Wavelength=1.54187

Cell:              a=10.1173(7)      b=20.5753(15)      c=30.337(2)
                   alpha=90          beta=90            gamma=90
Temperature:       293 K

                 Calculated                  Reported
Volume           6315.2(8)                   6315.2(8)
Space group      P b c a                     P b c a
Hall group       -P 2ac 2ab                  -P 2ac 2ab
Moiety formula   C17 H23 N3                  C17 H23 N3
Sum formula      C17 H23 N3                  C17 H23 N3
Mr               269.38                      269.39
Dx,g cm-3        1.133                       1.133
Z                16                          16
Mu (mm-1)        0.523                       0.523
F000             2336.0                      2336.0
F000'            2341.92
h,k,lmax         12,25,37                    12,24,37
Nref             6167                        6098
Tmin,Tmax        0.915,0.944
Tmin'            0.915

Correction method= Not given

Data completeness= 0.989          Theta(max)= 71.540

                                                      wR2(reflections)=
R(reflections)= 0.0748( 19904)                        0.0941( 20281)
S = 0.872                 Npar= 413
```

The following ALERTS were generated. Each ALERT has the format
**test-name_ALERT_alert-type_alert-level.**
Click on the hyperlinks for more details of the test.

### Alert level A

```
DIFMN02_ALERT_2_A  The minimum difference density is < -0.1*ZMAX*2.00
            _refine_diff_density_min given =    -24.200
            Test value =     -1.400
```

**Author Response: Altough the residual density is out of border but there is no doubt about the chemical structure taking into account other evidences, e.g. NMR. The error maybe due to crystal imperfections.**

```
PLAT097_ALERT_2_A Large Reported Max.  (Positive) Residual Density       9.52 eA-3
```

**Author Response: Altough the residual density is out of border but there is no doubt about the chemical structure taking into account other evidences, e.g. NMR. The error maybe due to crystal imperfections.**

```
PLAT098_ALERT_2_A Large Reported Min.  (Negative) Residual Density     -24.20 eA-3
```

**Author Response: Altough the residual density is out of border but there is no doubt about the chemical structure taking into account other evidences, e.g. NMR. The error maybe due to crystal imperfections.**

```
PLAT203_ALERT_2_A Negative Isotropic ADP for H1        ...........     -0.005 Report
```

**Author Response: Hydrogens give weak reflections. Crystal imperfections can not be excluded either.**

```
PLAT203_ALERT_2_A Negative Isotropic ADP for H2        ...........     -0.009 Report
```

**Author Response: Hydrogens give weak reflections. Crystal imperfections can not be excluded either.**

```
PLAT213_ALERT_2_A Atom C11             has ADP max/min Ratio .....        5.7 oblate
```

**Author Response: The structure is affected by some strain. Crystal imperfections can not be excluded either.**

```
PLAT703_ALERT_1_A Torsion Calc   -177.2(3), Rep   -178.2(4), Dev..       3.33 Sigma
              C(16-N(3)-C(17-C(19  1_555  1_555  1_555  1_555      #     5 Check
```

**Author Response: The structre is affected by some strain. Crystal imperfections can not be excluded either.**

```
PLAT703_ALERT_1_A Torsion Calc     -0.1(3), Rep      1.4(4), Dev..       5.00 Sigma
              C(17-N(3)-C(16-C(12  1_555  1_555  1_555  1_555      #    6 Check
```

**Author Response: The structre is affected by some strain. Crystal imperfections can not be excluded either.**

```
PLAT703_ALERT_1_A Torsion Calc   -178.3(3), Rep   -177.3(4), Dev..       3.33 Sigma
              C(7)-N(5)-C(1)-C(11  1_555  1_555  1_555  1_555      #   10 Check
```

**Author Response: The structre is affected by some strain. Crystal imperfections can not be excluded either.**

```
PLAT703_ALERT_1_A Torsion Calc     48.2(5), Rep     50.2(6), Dev..       4.00 Sigma
              C(30-N(7)-C(31-C(40  1_555  1_555  1_555  1_555      #   15 Check
```

**Author Response: The structre is affected by some strain. Crystal imperfections can not be excluded either.**

```
PLAT703_ALERT_1_A Torsion Calc    157.3(3), Rep    158.9(4), Dev..       5.33 Sigma
              C(39-N(7)-C(31-C(40  1_555  1_555  1_555  1_555      #   20 Check
```

**Author Response: The structre is affected by some strain. Crystal imperfections can not be excluded either.**

```
PLAT703_ALERT_1_A Torsion Calc   -154.1(3), Rep   -155.2(4), Dev..       3.67 Sigma
              C(32-N(8)-C(28-C(2)  1_555  1_555  1_555  1_555      #   22 Check
```

**Author Response: The structre is affected by some strain. Crystal imperfections can not be excluded either.**

```
PLAT703_ALERT_1_A Torsion Calc      0.4(3), Rep      2.1(4), Dev..       5.67 Sigma
              C(17-C(10-C(12-C(16  1_555  1_555  1_555  1_555      #   57 Check
```

**Author Response: The structre is affected by some strain. Crystal imperfections can not be excluded either.**

```
PLAT703_ALERT_1_A Torsion Calc     -0.2(3), Rep     -2.2(4), Dev..       6.67 Sigma
              C(10-C(12-C(16-N(3)  1_555  1_555  1_555  1_555      #   59 Check
```

**Author Response: The structre is affected by some strain. Crystal imperfections can not be excluded either.**

```
PLAT703_ALERT_1_A Torsion Calc   -169.2(3), Rep   -167.8(4), Dev..       4.67 Sigma
              C(10-C(12-C(21-C(29  1_555  1_555  1_555  1_555      #   61 Check
```

**Author Response: The structre is affected by some strain. Crystal imperfections can not be excluded either.**

```
PLAT703_ALERT_1_A Torsion Calc    179.4(2), Rep    178.4(3), Dev..       5.00 Sigma
              C(21-C(12-C(16-N(3)  1_555  1_555  1_555  1_555      #   63 Check
```

**Author Response: The structre is affected by some strain. Crystal imperfections can not be excluded either.**

```
PLAT703_ALERT_1_A Torsion Calc   -168.4(2), Rep   -167.3(3), Dev..       5.50 Sigma
              N(3)-C(16-C(18-C(25  1_555  1_555  1_555  1_555      #   66 Check
```

**Author Response: The structre is affected by some strain. Crystal imperfections can not be excluded either.**

```
PLAT703_ALERT_1_A Torsion Calc   -176.4(2), Rep   -177.4(3), Dev..       5.00 Sigma
              C(10-C(17-C(19-C(13  1_555  1_555  1_555  1_555      #   71 Check
```

**Author Response: The structre is affected by some strain. Crystal imperfections can not be excluded either.**

```
PLAT703_ALERT_1_A Torsion Calc    -35.6(3), Rep    -37.5(5), Dev..       6.33 Sigma
              C(12-C(21-C(29-C(25  1_555  1_555  1_555  1_555      #   79 Check
```

**Author Response: The structre is affected by some strain. Crystal imperfections can not be excluded either.**

```
PLAT703_ALERT_1_A Torsion Calc    -90.0(4), Rep    -91.3(5), Dev..       3.25 Sigma
              C(27-C(26-C(30-C(38  1_555  1_555  1_555  1_555      #   90 Check
```

**Author Response: The structre is affected by some strain. Crystal imperfections can not be excluded either.**

```
PLAT703_ALERT_1_A Torsion Calc     10.7(3), Rep     11.9(4), Dev..       4.00 Sigma
              N(8)-C(28-C(33-C(35  1_555  1_555  1_555  1_555      #   92 Check
```

**Author Response: The structre is affected by some strain. Crystal imperfections can not be excluded either.**

```
PLAT703_ALERT_1_A Torsion Calc     33.8(4), Rep     31.3(6), Dev..       6.25 Sigma
              N(7)-C(30-C(38-C(40  1_555  1_555  1_555  1_555      #   94 Check
```

**Author Response: The structre is affected by some strain. Crystal imperfections can not be excluded either.**

```
PLAT703_ALERT_1_A Torsion Calc    -20.6(5), Rep    -24.8(6), Dev..       8.40 Sigma
             N(7)-C(31-C(40-C(38  1_555  1_555  1_555  1_555      #   96 Check
```

**Author Response: The structre is affected by some strain. Crystal imperfections can not be excluded either.**

```
PLAT703_ALERT_1_A Torsion Calc    -35.5(3), Rep    -34.3(5), Dev..       4.00 Sigma
             N(8)-C(32-C(35-C(33  1_555  1_555  1_555  1_555      #   97 Check
```

**Author Response: The structre is affected by some strain. Crystal imperfections can not be excluded either.**

```
PLAT703_ALERT_1_A Torsion Calc     14.8(4), Rep     13.3(5), Dev..       3.75 Sigma
             C(28-C(33-C(35-C(32  1_555  1_555  1_555  1_555      #   98 Check
```

**Author Response: The structre is affected by some strain. Crystal imperfections can not be excluded either.**

```
PLAT703_ALERT_1_A Torsion Calc     -7.9(4), Rep     -3.5(6), Dev..      11.00 Sigma
             C(30-C(38-C(40-C(31  1_555  1_555  1_555  1_555      #  100 Check
```

**Author Response: The structre is affected by some strain. Crystal imperfections can not be excluded either.**

---

**Alert level B**

```
REFLT02_ALERT_1_B  The number of reflections greater than the sigma threshold
           cannot exceed the number of symmetry-independent reflections
           Number of symmetry-independent reflections =   6098
           Number of reflections greater than sigma threshold =  19904
PLAT213_ALERT_2_B Atom N7             has ADP max/min Ratio .....        4.9 prolat
```

**Author Response: The structure is affected by some strain. Crystal imperfections can not be excluded either.**

```
PLAT230_ALERT_2_B Hirshfeld Test Diff for    N3       --C16      .       10.0 s.u.
PLAT230_ALERT_2_B Hirshfeld Test Diff for    N8       --C32      .       12.6 s.u.
PLAT230_ALERT_2_B Hirshfeld Test Diff for    N8       --C36      .       33.0 s.u.
PLAT230_ALERT_2_B Hirshfeld Test Diff for    C12      --C16      .        8.3 s.u.
PLAT230_ALERT_2_B Hirshfeld Test Diff for    C13      --C19      .        8.0 s.u.
PLAT230_ALERT_2_B Hirshfeld Test Diff for    C25      --C29      .        7.5 s.u.
PLAT230_ALERT_2_B Hirshfeld Test Diff for    C28      --C33      .       14.7 s.u.
PLAT230_ALERT_2_B Hirshfeld Test Diff for    C32      --C35      .        7.4 s.u.
PLAT230_ALERT_2_B Hirshfeld Test Diff for    C33      --C35      .       11.0 s.u.
```

```
PLAT230_ALERT_2_B Hirshfeld Test Diff for    N7       --C30      .       11.0 s.u.
PLAT230_ALERT_2_B Hirshfeld Test Diff for    N7       --C31      .       17.6 s.u.
PLAT230_ALERT_2_B Hirshfeld Test Diff for    N7       --C39      .       42.9 s.u.
PLAT230_ALERT_2_B Hirshfeld Test Diff for    C1       --C11      .       15.3 s.u.
PLAT230_ALERT_2_B Hirshfeld Test Diff for    C20      --C23      .       14.3 s.u.
PLAT230_ALERT_2_B Hirshfeld Test Diff for    C31      --C40      .       10.9 s.u.
PLAT703_ALERT_1_B Torsion Calc     -2.4(4), Rep     -3.4(6), Dev..       2.50 Sigma
              C(3)-N(1)-C(19-C(17  1_555  1_555  1_555  1_555      #    2 Check
```

**Author Response: The structre is affected by some strain. Crystal imperfections can not be excluded either.**

```
PLAT703_ALERT_1_B Torsion Calc    -36.9(3), Rep    -37.8(5), Dev..       3.00 Sigma
              C(32-N(8)-C(28-C(33  1_555  1_555  1_555  1_555      #   23 Check
```

**Author Response: The structre is affected by some strain. Crystal imperfections can not be excluded either.**

```
PLAT703_ALERT_1_B Torsion Calc   -179.1(3), Rep   -179.9(3), Dev..       2.67 Sigma
              N(5)-C(1)-C(11-N(6)  1_555  1_555  1_555  1_555      #   27 Check
```

**Author Response: The structre is affected by some strain. Crystal imperfections can not be excluded either.**

```
PLAT703_ALERT_1_B Torsion Calc     -0.7(5), Rep     -2.2(6), Dev..       3.00 Sigma
              N(5)-C(1)-C(11-C(24  1_555  1_555  1_555  1_555      #   28 Check
```

**Author Response: The structre is affected by some strain. Crystal imperfections can not be excluded either.**

```
PLAT703_ALERT_1_B Torsion Calc      0.8(3), Rep      1.6(4), Dev..       2.67 Sigma
              N(5)-C(1)-C(22-C(20  1_555  1_555  1_555  1_555      #   29 Check
```

**Author Response: The structre is affected by some strain. Crystal imperfections can not be excluded either.**

```
PLAT703_ALERT_1_B Torsion Calc    177.6(3), Rep    176.9(4), Dev..       2.33 Sigma
              C(11-C(1)-C(22-C(20  1_555  1_555  1_555  1_555      #   31 Check
```

**Author Response: The structre is affected by some strain. Crystal imperfections can not be excluded either.**

```
PLAT703_ALERT_1_B Torsion Calc     -3.1(5), Rep     -4.5(6), Dev..       2.80 Sigma
              C(11-C(1)-C(22-C(26  1_555  1_555  1_555  1_555      #   32 Check
```

**Author Response: The structre is affected by some strain. Crystal imperfections can not be excluded either.**

```
PLAT703_ALERT_1_B Torsion Calc     -2.1(4), Rep     -3.0(5), Dev..       2.25 Sigma
            C(3)-C(2)-C(10-C(17  1_555  1_555  1_555  1_555      #   36 Check
```

**Author Response: The structre is affected by some strain. Crystal imperfections can not be excluded either.**

```
PLAT703_ALERT_1_B Torsion Calc   -176.3(3), Rep   -177.1(4), Dev..       2.67 Sigma
            C(28-C(2)-C(3)-N(1)  1_555  1_555  1_555  1_555      #   40 Check
```

**Author Response: The structre is affected by some strain. Crystal imperfections can not be excluded either.**

```
PLAT703_ALERT_1_B Torsion Calc     -0.1(5), Rep      1.3(7), Dev..       2.80 Sigma
            C(28-C(2)-C(10-C(12  1_555  1_555  1_555  1_555      #   43 Check
```

**Author Response: The structre is affected by some strain. Crystal imperfections can not be excluded either.**

```
PLAT703_ALERT_1_B Torsion Calc     -0.5(3), Rep     -1.2(4), Dev..       2.33 Sigma
            C(12-C(10-C(17-N(3)  1_555  1_555  1_555  1_555      #   55 Check
```

**Author Response: The structre is affected by some strain. Crystal imperfections can not be excluded either.**

```
PLAT703_ALERT_1_B Torsion Calc     -0.7(4), Rep      0.2(4), Dev..       2.25 Sigma
            C(21-C(12-C(16-C(18  1_555  1_555  1_555  1_555      #   64 Check
```

**Author Response: The structre is affected by some strain. Crystal imperfections can not be excluded either.**

```
PLAT703_ALERT_1_B Torsion Calc     11.7(4), Rep     10.8(6), Dev..       2.25 Sigma
            C(12-C(16-C(18-C(25  1_555  1_555  1_555  1_555      #   67 Check
```

**Author Response: The structre is affected by some strain. Crystal imperfections can not be excluded either.**

```
PLAT703_ALERT_1_B Torsion Calc    179.7(3), Rep   -179.5(4), Dev..       2.67 Sigma
            N(3)-C(17-C(19-N(1)  1_555  1_555  1_555  1_555      #   68 Check
```

**Author Response: The structre is affected by some strain. Crystal imperfections can not be excluded either.**

```
PLAT703_ALERT_1_B Torsion Calc      0.5(3), Rep     -0.3(4), Dev..       2.67 Sigma
              C(7)-C(20-C(22-C(1)  1_555  1_555  1_555  1_555      #   73 Check
```

**Author Response: The structre is affected by some strain. Crystal imperfections can not be excluded either.**

```
PLAT703_ALERT_1_B Torsion Calc    168.0(3), Rep    167.3(4), Dev..       2.33 Sigma
              C(22-C(20-C(23-C(37  1_555  1_555  1_555  1_555      #   76 Check
```

**Author Response: The structre is affected by some strain. Crystal imperfections can not be excluded either.**

```
PLAT703_ALERT_1_B Torsion Calc     50.2(4), Rep     51.4(5), Dev..       3.00 Sigma
              C(18-C(25-C(29-C(21  1_555  1_555  1_555  1_555      #   85 Check
```

**Author Response: The structre is affected by some strain. Crystal imperfections can not be excluded either.**

```
PLAT703_ALERT_1_B Torsion Calc     89.7(4), Rep     88.6(5), Dev..       2.75 Sigma
              C(22-C(26-C(30-C(38  1_555  1_555  1_555  1_555      #   88 Check
```

**Author Response: The structre is affected by some strain. Crystal imperfections can not be excluded either.**

```
PLAT703_ALERT_1_B Torsion Calc    131.3(3), Rep    132.2(4), Dev..       3.00 Sigma
              C(2)-C(28-C(33-C(35  1_555  1_555  1_555  1_555      #   93 Check
```

**Author Response: The structre is affected by some strain. Crystal imperfections can not be excluded either.**

```
PLAT703_ALERT_1_B Torsion Calc    152.9(4), Rep    151.8(5), Dev..       2.75 Sigma
              C(26-C(30-C(38-C(40  1_555  1_555  1_555  1_555      #   95 Check
```

**Author Response: The structre is affected by some strain. Crystal imperfections can not be excluded either.**

---

**Alert level C**

```
DIFMN03_ALERT_1_C  The minimum difference density is < -0.1*ZMAX*0.75
            The relevant atom site should be identified.
DIFMX02_ALERT_1_C  The maximum difference density is > 0.1*ZMAX*0.75
            The relevant atom site should be identified.
RINTA01_ALERT_3_C  The value of Rint is greater than 0.12
            Rint given   0.141
```

```
PLAT020_ALERT_3_C The Value of Rint is Greater Than 0.12 .........      0.141 Report
PLAT166_ALERT_4_C S.U's Given on Coordinates for Calc-flagged ....         H1 Note
PLAT166_ALERT_4_C S.U's Given on Coordinates for Calc-flagged ....         H2 Note
PLAT213_ALERT_2_C Atom C39            has ADP max/min Ratio .....        4.0 prolat
```

**Author Response: The structure is affected by some strain. Crystal imperfections can not be excluded either.**

```
PLAT220_ALERT_2_C NonSolvent   Resd 1  C   Ueq(max)/Ueq(min) Range        3.6 Ratio
PLAT220_ALERT_2_C NonSolvent   Resd 2  C   Ueq(max)/Ueq(min) Range        5.4 Ratio
PLAT220_ALERT_2_C NonSolvent   Resd 2  N   Ueq(max)/Ueq(min) Range        3.1 Ratio
PLAT222_ALERT_3_C NonSolvent Resd 2  H   Uiso(max)/Uiso(min) Range        4.4 Ratio
PLAT230_ALERT_2_C Hirshfeld Test Diff for    N3       --C17      .        6.3 s.u.
PLAT230_ALERT_2_C Hirshfeld Test Diff for    C2       --C3       .        5.7 s.u.
PLAT230_ALERT_2_C Hirshfeld Test Diff for    C2       --C10      .        5.3 s.u.
PLAT230_ALERT_2_C Hirshfeld Test Diff for    N6       --C11      .        7.0 s.u.
PLAT230_ALERT_2_C Hirshfeld Test Diff for    N6       --C27      .        6.2 s.u.
PLAT230_ALERT_2_C Hirshfeld Test Diff for    C7       --C14      .        5.2 s.u.
PLAT230_ALERT_2_C Hirshfeld Test Diff for    C7       --C20      .        5.3 s.u.
PLAT230_ALERT_2_C Hirshfeld Test Diff for    C22      --C26      .        5.2 s.u.
PLAT230_ALERT_2_C Hirshfeld Test Diff for    C34      --C37      .        5.6 s.u.
PLAT230_ALERT_2_C Hirshfeld Test Diff for    C38      --C40      .        5.1 s.u.
PLAT234_ALERT_4_C Large Hirshfeld Difference C26      --C30      .       0.16 Ang.
PLAT241_ALERT_2_C High   'MainMol' Ueq as Compared to Neighbors of        C25 Check
PLAT241_ALERT_2_C High   'MainMol' Ueq as Compared to Neighbors of        C33 Check
PLAT241_ALERT_2_C High   'MainMol' Ueq as Compared to Neighbors of        C34 Check
PLAT241_ALERT_2_C High   'MainMol' Ueq as Compared to Neighbors of        C37 Check
PLAT241_ALERT_2_C High   'MainMol' Ueq as Compared to Neighbors of        C38 Check
PLAT241_ALERT_2_C High   'MainMol' Ueq as Compared to Neighbors of        C40 Check
PLAT242_ALERT_2_C Low    'MainMol' Ueq as Compared to Neighbors of        C28 Check
PLAT242_ALERT_2_C Low    'MainMol' Ueq as Compared to Neighbors of        C11 Check
PLAT242_ALERT_2_C Low    'MainMol' Ueq as Compared to Neighbors of        C30 Check
PLAT242_ALERT_2_C Low    'MainMol' Ueq as Compared to Neighbors of        C31 Check
PLAT340_ALERT_3_C Low Bond Precision on  C-C Bonds ...............    0.00488 Ang.
PLAT361_ALERT_2_C Long   C(sp3)-C(sp3) Bond  C30      - C38      .       1.69 Ang.
PLAT703_ALERT_1_C Torsion Calc      0.1(5), Rep      1.1(6), Dev..       2.00 Sigma
              C(19-N(1)-C(3)-C(2)  1_555  1_555  1_555  1_555      #    3 Check
```

**Author Response: The structre is affected by some strain. Crystal imperfections can not be excluded either.**

```
PLAT703_ALERT_1_C Torsion Calc      0.4(3), Rep     -0.1(3), Dev..       1.67 Sigma
              C(16-N(3)-C(17-C(10  1_555  1_555  1_555  1_555      #    4 Check
```

**Author Response: The structre is affected by some strain. Crystal imperfections can not be excluded either.**

```
PLAT703_ALERT_1_C Torsion Calc   -178.2(3), Rep   -177.6(3), Dev..       2.00 Sigma
              C(1)-N(5)-C(7)-C(14  1_555  1_555  1_555  1_555      #    8 Check
```

**Author Response: The structre is affected by some strain. Crystal imperfections can not be excluded either.**

```
PLAT703_ALERT_1_C Torsion Calc     -1.8(3), Rep     -2.3(4), Dev..       1.67 Sigma
              C(7)-N(5)-C(1)-C(22  1_555  1_555  1_555  1_555      #   11 Check
```

**Author Response: The structre is affected by some strain. Crystal imperfections can not be excluded either.**

```
PLAT703_ALERT_1_C Torsion Calc    178.8(3), Rep    179.4(3), Dev..       2.00 Sigma
              C(27-N(6)-C(11-C(24  1_555  1_555  1_555  1_555      #   14 Check
```

**Author Response: The structre is affected by some strain. Crystal imperfections can not be excluded either.**

```
PLAT703_ALERT_1_C Torsion Calc     86.4(4), Rep     85.6(5), Dev..       2.00 Sigma
              C(39-N(7)-C(30-C(26  1_555  1_555  1_555  1_555      #   18 Check
```

**Author Response: The structre is affected by some strain. Crystal imperfections can not be excluded either.**

```
PLAT703_ALERT_1_C Torsion Calc   -159.6(3), Rep   -160.1(4), Dev..       1.67 Sigma
              C(39-N(7)-C(30-C(38  1_555  1_555  1_555  1_555      #   19 Check
```

**Author Response: The structre is affected by some strain. Crystal imperfections can not be excluded either.**

```
PLAT703_ALERT_1_C Torsion Calc   -153.9(3), Rep   -153.5(3), Dev..       1.33 Sigma
              C(36-N(8)-C(28-C(33  1_555  1_555  1_555  1_555      #   25 Check
```

**Author Response: The structre is affected by some strain. Crystal imperfections can not be excluded either.**

```
PLAT703_ALERT_1_C Torsion Calc      5.0(5), Rep      6.0(6), Dev..       2.00 Sigma
              C(22-C(1)-C(11-N(6)  1_555  1_555  1_555  1_555      #   33 Check
```

**Author Response: The structre is affected by some strain. Crystal imperfections can not be excluded either.**

```
PLAT703_ALERT_1_C Torsion Calc   -176.6(3), Rep   -176.2(3), Dev..       1.33 Sigma
              C(22-C(1)-C(11-C(24  1_555  1_555  1_555  1_555      #   34 Check
```

**Author Response: The structre is affected by some strain. Crystal imperfections can not be excluded either.**

```
PLAT703_ALERT_1_C Torsion Calc   -178.6(3), Rep   -178.0(4), Dev..       2.00 Sigma
            C(3)-C(2)-C(10-C(12  1_555  1_555  1_555  1_555      #   35 Check
```

**Author Response: The structre is affected by some strain. Crystal imperfections can not be excluded either.**

```
PLAT703_ALERT_1_C Torsion Calc   -163.7(3), Rep   -164.3(4), Dev..       2.00 Sigma
            C(10-C(2)-C(28-N(8)  1_555  1_555  1_555  1_555      #   41 Check
```

**Author Response: The structre is affected by some strain. Crystal imperfections can not be excluded either.**

```
PLAT703_ALERT_1_C Torsion Calc     86.0(3), Rep     85.5(4), Dev..       1.67 Sigma
            C(10-C(2)-C(28-C(33  1_555  1_555  1_555  1_555      #   42 Check
```

**Author Response: The structre is affected by some strain. Crystal imperfections can not be excluded either.**

```
PLAT703_ALERT_1_C Torsion Calc     -1.7(3), Rep     -1.2(4), Dev..       1.67 Sigma
            N(5)-C(7)-C(20-C(22  1_555  1_555  1_555  1_555      #   46 Check
```

**Author Response: The structre is affected by some strain. Crystal imperfections can not be excluded either.**

```
PLAT703_ALERT_1_C Torsion Calc     -1.7(5), Rep     -2.3(6), Dev..       1.20 Sigma
            C(14-C(7)-C(20-C(23  1_555  1_555  1_555  1_555      #   49 Check
```

**Author Response: The structre is affected by some strain. Crystal imperfections can not be excluded either.**

```
PLAT703_ALERT_1_C Torsion Calc    -12.7(4), Rep    -12.1(6), Dev..       1.50 Sigma
            C(20-C(7)-C(14-C(34  1_555  1_555  1_555  1_555      #   50 Check
```

**Author Response: The structre is affected by some strain. Crystal imperfections can not be excluded either.**

```
PLAT703_ALERT_1_C Torsion Calc     -2.4(5), Rep     -3.2(7), Dev..       1.60 Sigma
            C(2)-C(10-C(12-C(21  1_555  1_555  1_555  1_555      #   52 Check
```

**Author Response: The structre is affected by some strain. Crystal imperfections can not be excluded either.**

```
PLAT703_ALERT_1_C Torsion Calc   -177.9(2), Rep   -177.6(3), Dev..       1.50 Sigma
            C(2)-C(10-C(17-N(3)  1_555  1_555  1_555  1_555      #   53 Check
```

**Author Response: The structre is affected by some strain. Crystal imperfections can not be excluded either.**

```
PLAT703_ALERT_1_C Torsion Calc     -0.1(4), Rep      0.7(6), Dev..       2.00 Sigma
              C(2)-C(10-C(17-C(19  1_555  1_555  1_555  1_555      #   54 Check
```

**Author Response: The structre is affected by some strain. Crystal imperfections can not be excluded either.**

```
PLAT703_ALERT_1_C Torsion Calc   -179.0(2), Rep   -178.6(4), Dev..       2.00 Sigma
              C(17-C(10-C(12-C(21  1_555  1_555  1_555  1_555      #   58 Check
```

**Author Response: The structre is affected by some strain. Crystal imperfections can not be excluded either.**

```
PLAT703_ALERT_1_C Torsion Calc     41.6(4), Rep     41.1(5), Dev..       1.25 Sigma
              C(7)-C(14-C(34-C(37  1_555  1_555  1_555  1_555      #   65 Check
```

**Author Response: The structre is affected by some strain. Crystal imperfections can not be excluded either.**

```
PLAT703_ALERT_1_C Torsion Calc    -35.4(4), Rep    -34.9(5), Dev..       1.25 Sigma
              C(16-C(18-C(25-C(29  1_555  1_555  1_555  1_555      #   72 Check
```

**Author Response: The structre is affected by some strain. Crystal imperfections can not be excluded either.**

```
PLAT703_ALERT_1_C Torsion Calc    179.8(3), Rep   -179.8(3), Dev..       1.33 Sigma
              C(1)-C(22-C(26-C(30  1_555  1_555  1_555  1_555      #   81 Check
```

**Author Response: The structre is affected by some strain. Crystal imperfections can not be excluded either.**

```
PLAT703_ALERT_1_C Torsion Calc   -177.6(4), Rep   -177.1(4), Dev..       1.25 Sigma
              C(30-C(26-C(27-N(6)  1_555  1_555  1_555  1_555      #   91 Check
```

**Author Response: The structre is affected by some strain. Crystal imperfections can not be excluded either.**

---

## Alert level G

```
PLAT005_ALERT_5_G No Embedded Refinement Details Found  in the CIF     Please Do !
PLAT007_ALERT_5_G Number of Unrefined Donor-H Atoms .............          1 Report
              H3
PLAT199_ALERT_1_G Reported _cell_measurement_temperature ..... (K)        293 Check
```

```
PLAT200_ALERT_1_G Reported   _diffrn_ambient_temperature ..... (K)        293 Check
PLAT793_ALERT_4_G Model has Chirality at C28        (Centro SpGr)           R Verify
PLAT793_ALERT_4_G Model has Chirality at C30        (Centro SpGr)           R Verify
PLAT808_ALERT_5_G No Parseable SHELXL Style Weighting Scheme Found     Please Check
PLAT882_ALERT_1_G No Datum for _diffrn_reflns_av_unetI/netI ......     Please Do !
PLAT883_ALERT_1_G Absent Datum for _atom_sites_solution_primary ..     Please Do !
```

---

```
 26 ALERT level A = Most likely a serious problem - resolve or explain
 37 ALERT level B = A potentially serious problem, consider carefully
 58 ALERT level C = Check. Ensure it is not caused by an omission or oversight
  9 ALERT level G = General information/check it is not something unexpected

 71 ALERT type 1 CIF construction/syntax error, inconsistent or missing data
 47 ALERT type 2 Indicator that the structure model may be wrong or deficient
  4 ALERT type 3 Indicator that the structure quality may be low
  5 ALERT type 4 Improvement, methodology, query or suggestion
  3 ALERT type 5 Informative message, check
```

---

It is advisable to attempt to resolve as many as possible of the alerts in all categories. Often the minor alerts point to easily fixed oversights, errors and omissions in your CIF or refinement strategy, so attention to these fine details can be worthwhile. In order to resolve some of the more serious problems it may be necessary to carry out additional measurements or structure refinements. However, the purpose of your study may justify the reported deviations and the more serious of these should normally be commented upon in the discussion or experimental section of a paper or in the "special_details" fields of the CIF. checkCIF was carefully designed to identify outliers and unusual parameters, but every test has its limitations and alerts that are not important in a particular case may appear. Conversely, the absence of alerts does not guarantee there are no aspects of the results needing attention. It is up to the individual to critically assess their own results and, if necessary, seek expert advice.

### Publication of your CIF in IUCr journals

A basic structural check has been run on your CIF. These basic checks will be run on all CIFs submitted for publication in IUCr journals (*Acta Crystallographica*, *Journal of Applied Crystallography*, *Journal of Synchrotron Radiation*); however, if you intend to submit to *Acta Crystallographica Section C* or *E* or *IUCrData*, you should make sure that full publication checks are run on the final version of your CIF prior to submission.

### Publication of your CIF in other journals

Please refer to the *Notes for Authors* of the relevant journal for any special instructions relating to CIF submission.

## Validation response form

Please find below a validation response form (VRF) that can be filled in and pasted into your CIF.

```
# start Validation Reply Form
_vrf_REFLT02__142469_3
;
PROBLEM: The number of reflections greater than the sigma threshold
RESPONSE: ...
;
_vrf_DIFMN03__142469_3
;
PROBLEM: The minimum difference density is < -0.1*ZMAX*0.75
RESPONSE: ...
;
_vrf_DIFMX02__142469_3
;
PROBLEM: The maximum difference density is > 0.1*ZMAX*0.75
RESPONSE: ...
;
_vrf_RINTA01__142469_3
;
PROBLEM: The value of Rint is greater than 0.12
RESPONSE: ...
;
_vrf_PLAT230__142469_3
;
PROBLEM: Hirshfeld Test Diff for    N3       --C16      .       10.0 s.u.
RESPONSE: ...
;
_vrf_PLAT020__142469_3
;
PROBLEM: The Value of Rint is Greater Than 0.12 .........      0.141 Report
RESPONSE: ...
;
_vrf_PLAT166__142469_3
;
PROBLEM: S.U's Given on Coordinates for Calc-flagged ....         H1 Note
RESPONSE: ...
;
_vrf_PLAT220__142469_3
;
PROBLEM: NonSolvent   Resd 1  C   Ueq(max)/Ueq(min) Range        3.6 Ratio
RESPONSE: ...
;
_vrf_PLAT222__142469_3
;
PROBLEM: NonSolvent Resd 2  H   Uiso(max)/Uiso(min) Range        4.4 Ratio
RESPONSE: ...
;
_vrf_PLAT234__142469_3
;
PROBLEM: Large Hirshfeld Difference C26      --C30      .       0.16 Ang.
RESPONSE: ...
;
_vrf_PLAT241__142469_3
;
PROBLEM: High   'MainMol' Ueq as Compared to Neighbors of        C25 Check
RESPONSE: ...
;
_vrf_PLAT242__142469_3
```

```
;
PROBLEM: Low    'MainMol' Ueq as Compared to Neighbors of        C28 Check
RESPONSE: ...
;
_vrf_PLAT340__142469_3
;
PROBLEM: Low Bond Precision on  C-C Bonds ...............    0.00488 Ang.
RESPONSE: ...
;
_vrf_PLAT361__142469_3
;
PROBLEM: Long   C(sp3)-C(sp3) Bond  C30      - C38      .       1.69 Ang.
RESPONSE: ...
;
# end Validation Reply Form
```

---

## PLATON version of 11/11/2024; check.def file version of 11/11/2024

**Datablock _142469_3** - ellipsoid plot

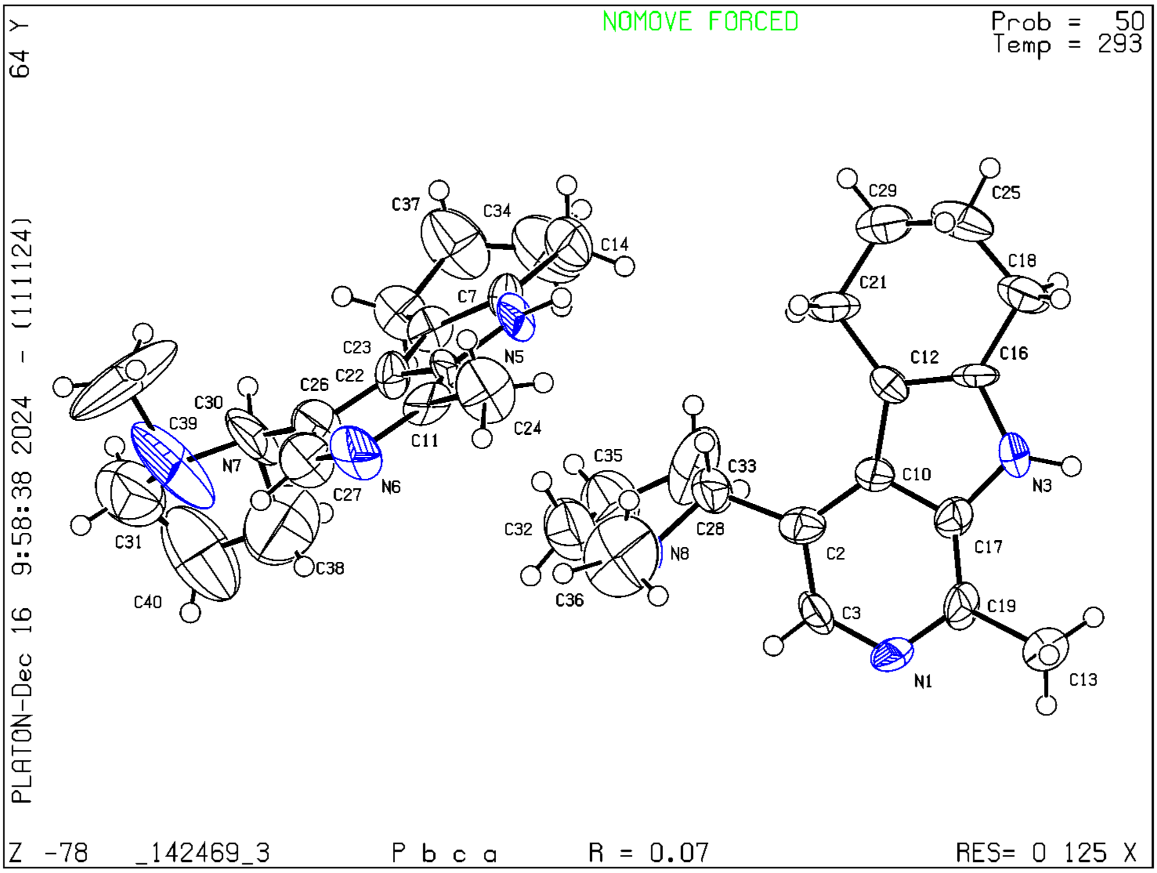

Supplement: File 3 — Checkcif file for compound 31. [file Beilstein_J_Org_Chem-21-955-s003.pdf]
